# Identification and Initial Characterization of the Effectors of an Anther Smut Fungus and Potential Host Target Proteins

**DOI:** 10.3390/ijms18112489

**Published:** 2017-11-22

**Authors:** Venkata S. Kuppireddy, Vladimir N. Uversky, Su San Toh, Ming-Chang Tsai, William C. Beckerson, Catarina Cahill, Brittany Carman, Michael H. Perlin

**Affiliations:** 1 Department of Biology, Program on Disease Evolution, University of Louisville, Louisville, KY 40208, USA; mail2swathi.k@gmail.com (V.S.K.); cloudysusan@gmail.com (S.S.T.); m0tsai02@louisville.edu (M.-C.T.); william.beckerson@louisville.edu (W.C.B.); catarina.cahill@louisville.edu (C.C.); brittany.carman@louisville.edu (B.C.); 2 Department of Molecular Biology and University of South Florida Health Byrd Alzheimer’s Research Institute, Morsani College of Medicine, University of South Florida, Tampa, FL 33612, USA; vuversky@health.usf.edu; 3 Laboratory of New Methods in Biology, Institute for Biological Instrumentation, Russian Academy of Sciences, Institutskaya Str., 7, Pushchino, Moscow Region 142290, Russia

**Keywords:** biotrophic pathogen, anther smut, fungal effectors, *Microbotryum violaceum*

## Abstract

(1) Background: Plant pathogenic fungi often display high levels of host specificity and biotrophic fungi; in particular, they must manipulate their hosts to avoid detection and to complete their obligate pathogenic lifecycles. One important strategy of such fungi is the secretion of small proteins that serve as effectors in this process. *Microbotryum violaceum* is a species complex whose members infect members of the Caryophyllaceae; *M. lychnidis-dioicae*, a parasite on *Silene latifolia*, is one of the best studied interactions. We are interested in identifying and characterizing effectors of the fungus and possible corresponding host targets; (2) Methods: In silico analysis of the *M. lychnidis-dioicae* genome and transcriptomes allowed us to predict a pool of small secreted proteins (SSPs) with the hallmarks of effectors, including a lack of conserved protein family (PFAM) domains and also localized regions of disorder. Putative SSPs were tested for secretion using a yeast secretion trap method. We then used yeast two-hybrid analyses for candidate-secreted effectors to probe a cDNA library from a range of growth conditions of the fungus, including infected plants; (3) Results: Roughly 50 SSPs were identified by in silico analysis. Of these, 4 were studied further and shown to be secreted, as well as examined for potential host interactors. One of the putative effectors, MVLG_01732, was found to interact with *Arabidopsis thaliana* calcium-dependent lipid binding protein (AtCLB) and with cellulose synthase interactive protein 1 orthologues; and (4) Conclusions: The identification of a pool of putative effectors provides a resource for functional characterization of fungal proteins that mediate the delicate interaction between pathogen and host. The candidate targets of effectors, e.g., AtCLB, involved in pollen germination suggest tantalizing insights that could drive future studies.

## 1. Introduction

During fungal infection of plants, a number of fungi secrete small proteins that serve to manipulate host responses and downstream events in host development during infection. Often such proteins allow biotrophic fungi to evade host defenses, but they can also redirect development so as to specifically benefit the fungus. Such proteins have been termed “effectors”, and many share common characteristics among different fungi [1,2]. For instance, for oomycete pathogens, such as *Phytophthera* species, or rust species (e.g., *Melampsora lini*), small secreted proteins (SSPs) are secreted from specialized structures called haustoria that penetrate host plant cells to draw nutrients from their hosts. Such fungal effectors are SSPs that bear an N-terminal signal peptide; the effectors are usually unique to the pathogen. Most effectors are cysteine-rich and share no sequence similarity with other known proteins, thus revealing the specialized arsenal that each pathogen possesses and, most likely, their association with the specificity of the pathogen for its host. Some effectors are translocated directly from the infection structures, i.e., haustoria or appressoria, into plant cells, while others interact with host cell receptors and get internalized into the cell [1]. Some studies suggest that secreted proteins can act as structural effectors that could accumulate at the host/pathogen interface and stabilize the fungal filaments [2]. However, the mechanism of how these effectors work in the entry into the plant cell or in the proliferation of the fungus inside the host has yet to be fully elucidated. *Microbotryum lychnidis-dioicae* is an obligate biotrophic basidiomycete smut fungus and is a member of the *Microbotryum violaceum* species complex that infects members of the Caryophyllaceae family. *M. lychnidis-dioicae* infects the dioecious host plant, *Silene latifolia.* The fungal life cycle begins when the fungal spores are disseminated by wind or pollinator species and land on a suitable host. The diploid teliospores then undergo meiosis to produce yeast-like haploid sporidia that reproduce by budding. Conjugation takes place between sporidia of opposite mating type, under suitable conditions, such as low nutrients and cool temperatures. Conjugation results in the formation of an infectious dikaryotic hypha that is stabilized by host cues, allowing the fungus to produce an appressorium and penetrate the host tissue. The fungus overwinters in the meristematic tissue; infection becomes systemic in the following year, producing diseased flowers, in which the pollen has been replaced with fungal spores, thus rendering the male plants sterile. It is thus commonly referred to as the “anther smut” [3]. Karyogamy occurs in the dikaryotic hyphae resulting in the formation of diploid spores, thus completing the life cycle. The fungal life cycle thus exhibits both a saprobic haploid phase and a parasitic dikaryotic/diploid phase. The disease also aborts the development of female organs in female host plants. Moreover, the female plants develop immature male reproductive anthers, making this one of the most interesting cases of parasitic modification of host floral organs. Linnaeus was the first to notice the smut-induced anthers in the female host plants [4]. Since pollination drives disease transmission, anther smut is considered as a plant sexually transmitted disease (STD) [5].

Recently, the genome sequence and transcriptomes of *M. lychnidis-dioicae* and its interaction with the host *S. latifolia* have been produced [6]. However, there have been no experimental data provided to explain how this fungus can divert the host resources for its own propagation and survival. Here, we provide the first study to examine the function of the candidate proteins, i.e., the putative effectors that might be involved in the pathogenicity of this group of fungi.

## 2. Results

### 2.1. In Silico Analyses to Identify Potential Effectors

To provide a conservative estimate of proteins secreted by *Microbotryum lychnidis-dioicae*, several bioinformatic tools were employed, and only those proteins that passed all measures used were retained in the list of predicted secreted proteins (Table S1). Out of 7364 proteins, 279 were identified to have a signal peptide; from this group, 71 predicted proteins were smaller than 250 amino acids (hereafter referred to as small secreted proteins, SSPs). Of these, 46 appeared to be unique to *M. lychnidis-dioicae* or to the *Microbotryum* complex, and 60 lacked identifiable PFAM domains. Among the SSPs, 19 were also significantly upregulated during plant infection, suggesting that these may play a role during those stages of the fungal lifecycle and in pathogenicity [7].

### 2.2. Intrinsic Disorder in Predicted Small Secreted Proteins (SSPs)

Intrinsic disorder is known to play an important role in protein-protein interactions [8,9,10,11,12,13,14]; intrinsically disordered proteins (IDPs), hybrid proteins containing ordered domains, and intrinsically disordered protein regions (IDPRs) are common among pathogenic microbes [15], and play a number of roles in pathogen-host interactions [16,17]. Accordingly, we analyzed the overall intrinsic disorder predisposition of the 49 predicted secreted proteins from *M. lychnidis-dioicae* upregulated during infection, using a set of established disorder predictors from the PONDR family (PONDR^®^ VSL2 [18], PONDR^®^ VLXT [19], PONDR^®^ VL3 [20], and PONDR^®^ FIT [21]). We also used the ANCHOR algorithm [22,23] to evaluate the presence of the disorder-based protein-protein interaction sites, molecular recognition features (MoRFs), i.e., regions that might undergo the binding-induced disorder-to-order transition. Results of these analyses are summarized in Supplementary Materials, Table S2. These results draw a picture of an impressive prevalence of intrinsic disorder in the *M. lychnidis-dioicae* SSPs. In fact, all putative effectors have regions of intrinsic disorder, and many of the effectors are very disordered. In particular, 11 effectors (22.4%) can be classified as mostly disordered, since they have >50% disordered residues; 19 effectors (38.8%) are highly disordered, possessing between 30 and 50% of disordered residues; 18 effectors (36.7%) are moderately disordered, since they have between 10% and 30% disordered residues; and just one protein (2.1%) has less than 10% disordered residues and therefore is mostly ordered. These values for disorder content are very high even for a eukaryotic organism and are rather atypical for groups of proteins that are not specifically selected for disorder. Furthermore, many effectors have disorder-based binding sites or MoRFs (i.e., sites that are disordered in the unbound state and undergo disorder-to-order transition at interaction with the binding partners). Finally, several effectors have more than one MoRF, suggesting that they can be engaged in interaction with multiple partners or, being engaged in interaction with one partner, utilize multivalent “wrapping around”-type binding mode. It is likely that the exceptionally high disorder levels and the presence of MoRFs can simplify interactions of these pathogenic effectors with host proteins or play some other role in regulation of the SSP functionality.

**Figure 1:**
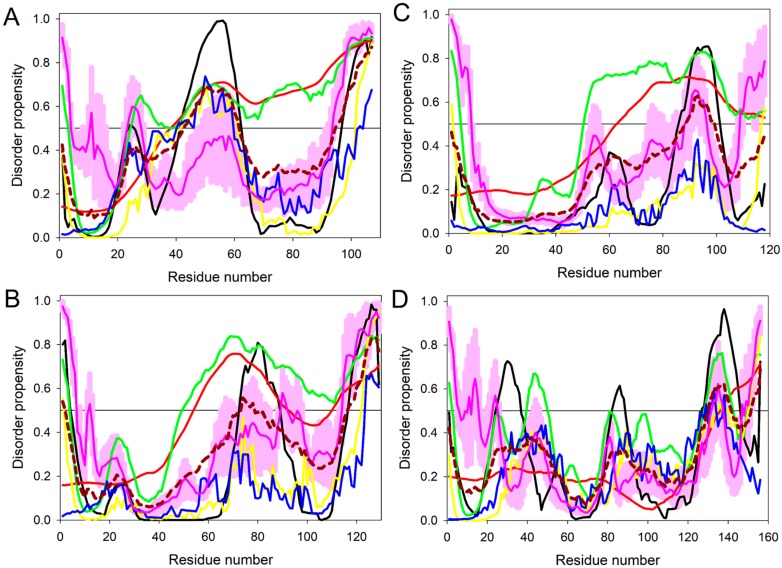
Evaluating intrinsic disorder propensity of protein effectors (**A**) MVLG_04106; (**B**) MVLG_05720; (**C**) MVLG_06175 and (**D**) MVLG_01732 by a series of per-residue disorder predictors. Disorder profiles generated by PONDR^®^ VLXT, PONDR^®^ VL3, PONDR^®^ VSL2, IUPred_short, IUPred_long, and PONDR^®^ FIT, are shown by black, red, green, yellow, blue, and pink lines, respectively. Dark red dashed line shows the mean disorder propensity calculated by averaging disorder profiles of individual predictors. Light pink shadow around the PONDR^®^ FIT shows error distribution. In these analyses, the predicted intrinsic disorder scores above 0.5 are considered to correspond to the disordered residues/regions, whereas regions with the disorder scores between 0.2 and 0.5 are considered flexible.

In line with the hypothesis that intrinsic disorder can be of functional importance for the SSPs from *M. lychnidis-dioicae*, Figure 1 represents in-depth analysis of the intrinsic disorder predisposition of four putative effector proteins that were up-regulated during infection and were shown to have important functions (Section 2.3 and Section 2.4 for the detailed functional characterization of these proteins).

The corresponding disorder profiles were by the overlay of the outputs of six commonly used disorder predictors, PONDR^®^ VSL2 [18], PONDR^®^ VLXT [19], PONDR^®^ VL3 [20], PONDR^®^ FIT [21], as well as IUPred_short and IUPred_long [24]. Furthermore, for each of these four proteins, mean per-residue disorder probability was calculated by averaging disorder profiles generated by the individual predictors. The use of consensus for evaluation of intrinsic disorder is motivated by empirical observations that this approach usually increases the predictive performance compared to using a single predictor [25,26,27]. Figure 1 clearly shows that these four proteins are characterized by high levels of predicted disorder that range (as per the outputs of PONDR^®^ VSL2 analysis) from 22.4% in MVLG_01732 to 61.0% in MVLG_06175, to 64.3% in MVLG_05720, and to 79.4% in MVLG_04106. According to the PONDR^®^ VSL2-based analysis, there are four IDPRs in MVLG_01732 (residues 1–2, 40–50, 128–142 and 150–156) and three IDPRs in MVLG_04106 (residues 1–3, 25–37, and 39–107), whereas MVLG_06175 and MVLG_05720 have two IDPRs each (residues 1–4 and 51–118 and residues 1–3 and 50–129, respectively). Furthermore, according to the ANCHOR analysis, each of these four SSPs might have at least one MoRF (residues 145–153 in MVLG_01732, residues 113–118 in MVLG_06175, residues 7–12 in MVLG_05720, and residues 3–12 in MVLG_04106). The presence of MoRFs in these proteins was also analyzed by MoRF_CHiBi_, which is a new computational approach for fast and accurate prediction of MoRFs in protein sequences. This analysis showed that although there is no MoRF_CHiBi_-identified MoRF in MVLG_01732, this protein has two regions with some potential to act as MoRFs (residues 29–39 and 139–156). Similarly, there are no MoRF_CHiBi_-identified MoRFs in MVLG_05720, which, however, have four regions with some potential to act as MoRFs (residues 1–14, 66–76, 97–104, and 120–129). On the other hand, MVLG_06175 has two MoRFs (residues 97–111 and 113–118), and almost the entire chain of MVLG_04106 can act as disorder-based binding region, since this protein has two MoRFs, residues 1–70 and 87–104, that cover almost 83% of its sequence.

### 2.3. Yeast Secretion Trap to Verify the Secretory Nature of Predicted Effectors

**Figure 2:**
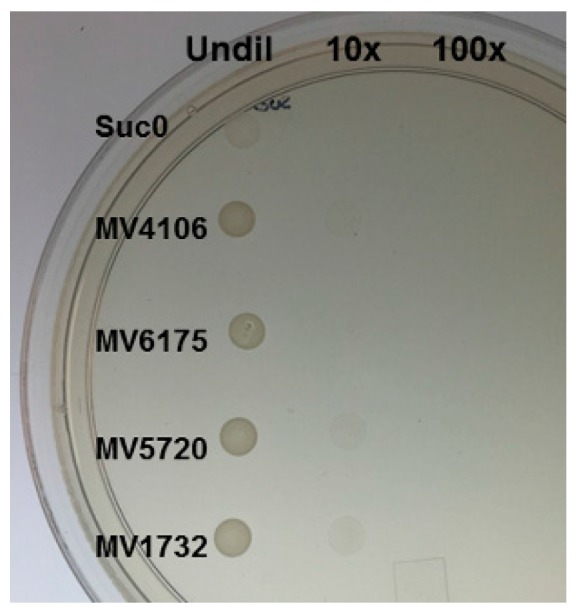
Results of secretion trap experiment with four *M. lychnidis-dioicae* predicted small secreted proteins (SSP) effectors. Suc0, yeast cells transformed with vector alone on sucrose, leu drop-out medium. Undil, undiluted; 10× and 100× dilutions.

We used Yeast Secretion Trap (YST) [28], a molecular genetic approach, to confirm the secretory nature of a small subset of the SSP putative effector proteins (MVLG_01732, MVLG_04106, MVLG_05720, and MVLG_06175; Table 1), each of which was also up-regulated during infection. Three of these proteins were also Cys-rich (MVLG_04106, MVLG_05720, and MVLG_06175), another hallmark of effectors in a number of fungal species [29]. YST employs a mutant strain of yeast, SEY 6210, that has a deletion in the *SUC2* locus encoding the enzyme, invertase. Invertase catalyzes hydrolysis of the disaccharide, sucrose, to glucose and fructose, so that the yeast cell can then take up glucose and metabolize this sugar. Thus, the SEY 6210 mutant yeast strain is normally unable to grow on media where sucrose is the sole carbon source. The method uses a vector, pYSTO-0, bearing the coding region of Suc2 invertase without its signal peptide and its start codon. The protein of interest can be cloned as a translational fusion protein with the invertase driven by a constitutive promoter from *ADH1*. If the protein of interest is secreted, this will result in the reconstituted functional activity of the invertase and enable the yeast cells to grow on sucrose medium. All four predicted effectors from *M. lychnidis-dioicae* examined experimentally with the yeast secretion trap assay indeed appeared to be secreted, since the signal peptide of each allowed Suc2p to be secreted and thus provide for growth of the yeast SEY 6210 mutant on sucrose medium (Figure 2). In contrast, SEY 6210 cells transformed with the vector only were unable to grow on such media.

### 2.4. Yeast Two-Hybrid Experiment

Our goal was to determine the function of these fungal proteins that are predicted, and now confirmed, to be secreted, as well as being highly expressed, during infection. We employed yeast two-hybrid genetic screening to identify the possible host interactors for these fungal proteins. As mentioned above, we chose a small subset of the SSPs that were also found to be induced in expression *in planta*.

#### 2.4.1. MVLG_04106 Autoactivates the Reporter Genes in Yeast Two-Hybrid Assay

**Figure 3:**
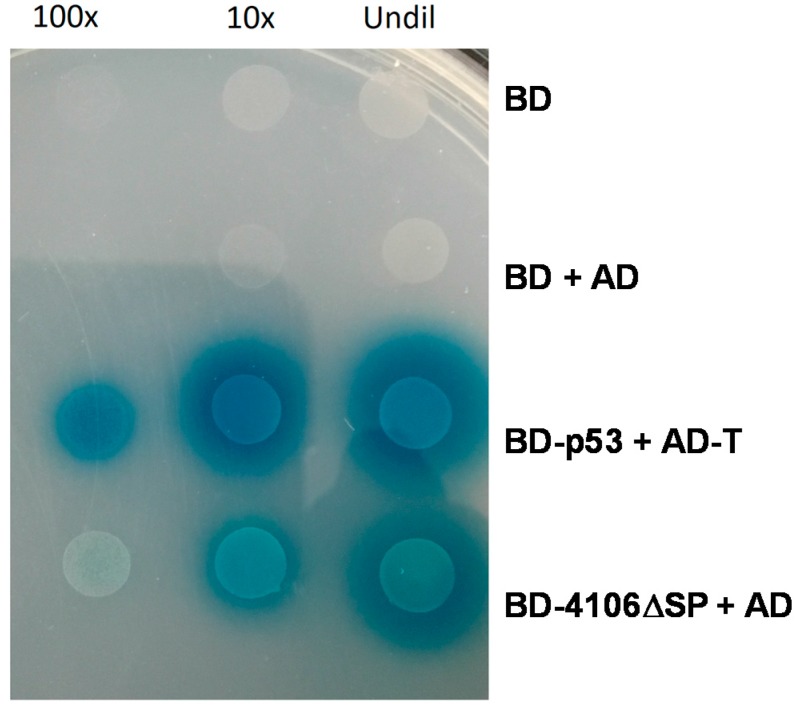
Autoactivation of three reporter genes by MVLG_04106 on QDO/X-α-gal + 3-AT (5 mM) plates. Undil, undiluted; 10× and 100× dilutions. QDO (Quadruple drop out media), 3-AT (3-Amino-1,2,4-triazole), BD (DNA binding domain in pGBKT7 vector), AD (Activation domain in pGADT7 vector), BD-p53 (pGBKT7-53 positive control plasmid), AD-T (pGADT7-T positive control plasmid), and BD-4106∆SP (MVLG_04106 lacking signal peptide).

We expressed MVLG_04106 lacking its signal peptide as a fusion protein to Gal4BD in the bait vector (pGBKT7-MVLG_04106∆SP) and tested its activity in expressing the reporter genes. It was found that the yeast strain transformed with this construct activated all three of the reporter genes-*HIS3*, *ADE2*, and *MEL1*, when mated with the opposite mating strain containing only the control prey vector (Figure 3). This indicates the cells’ ability to grow on media lacking the essential nutrients histidine and adenine because of the activation of the enzymes aminoimidazole ribonucleotide carboxylase 2 (*ADE2*) and imidazole glycerol phosphate dehydratase 3 (*HIS3*). Moreover, the cells were also able to express α-galactosidase, the gene product of the Melibiase 1 *(MEL1*) reporter gene that enables the yeast cells to turn blue-green in the presence of the chromogenic substrate X-α-gal. This was unexpected, so we generated the reciprocal set of constructs to further investigate possible transcriptional activation by MVLG_04106. In this case, a fusion protein was generated with MVLG_04106 and Gal4AD in the prey vector to test if the reporter genes could again be activated. Surprisingly, in this case the reporter genes were not activated. This suggests that MVLG_04106 could activate the transcription of the reporter genes only when attached to the corresponding DNA binding domain for those genes (i.e., Gal4BD). One possibility is that this fungal protein acts as a transcription factor in modulating the host gene expression during infection. In line with the known fact that transcription factors are typically characterized by high levels of intrinsic disorder [30,31,32], MVLG_04106 was predicted to possess 79.4% disordered residues (see Figure 1A) and is shown to contain long disorder-based interaction regions. The predicted protein contains 106 amino acid residues and is cysteine rich, with approximately 5% Cys residues. Further domain analysis using PROSITE did not yield any information, but prediction of post translational modification sites indicated proteolytic cleavage at residue D35, which could allow the mature protein to function as a transcriptional regulator [33] (Supplemental Table S2). Structural modelling using Swiss-Model yielded chorismite mutase for residues 30–65, for which there was 22.22% similarity in the 3-dimensional structure. When we compared the amino acid sequence of MVLG_04106 with predicted proteins of *M. silenes-dioicae* [34], there was 99.07% identity with the corresponding orthologue, whereas that for the *M. violaceum sensu lato* species [35], only had 63.04% identity.

#### 2.4.2. MVLG_05720 Fungal Protein Interacts with Fungal Proteins

Yeast two-hybrid screening with MVLG_05720 yielded 614 colonies after the initial stringent selection on QDO medium with 5 mM 3AT, along with screening for α-galactosidase expression on X-α-gal (as blue-green colonies). Further selection on 50 mM 3AT to reduce leaky *HIS* selection yielded 129 colonies for examination via sequence analysis. Of the 129 sequenced clones, we recovered only fungal interactors: 99 of the clones represented MVLG_07305, 27 of the clones were found to be MVLG_04206, and 3 of the clones matched MVLG_04267. Figure 1B illustrates that there are 64.3% disordered residues in MVLG_05720, and this protein has several MoRFs. It contains 129 amino acids and is highly cysteine rich with roughly 9% Cys residues. When the amino acid sequence of MVLG_05720 was compared to the genomes of *M. silenes-dioicae* and *M. violaceum sensu lato*, the corresponding orthologues showed 96.9% identity and 85.93% identity, respectively.

#### 2.4.3. MVLG_06175 Interacts with a Host Protein and a Fungal Protein

Yeast two-hybrid screening with MVLG_06175 initially yielded 1000 colonies after the stringent selection on QDO/X-α-gal + 3AT (5 mM) medium. Further selection to reduce leaky *HIS* selection yielded 201 colonies for examination via sequence analysis. Of the 39 sequenced clones that we recovered, 4 of them were full length clones that encode CASPL2C1, 1 was a fungal protein encoded by MVLG_06379, and the rest of the sequenced clones were for the fungal protein encoded by MVLG_07305 mentioned above. The *S. latifolia* genomic region matching the sequence for CASPL2C1 is found on contig m.88187 (GenBank: FMHP01040264.1) in NCBI for the *Silene latifolia* genome assembly (taxid:37657). It also corresponded to c93454_g1 RNA detected in RNA-Seq experiments [6,7]. According to Figure 1C, 61.0% of residues in MVLG_06175 are predicted to be intrinsically disordered and this protein can be engaged in disorder-based protein-protein interactions. It contains 118 amino acids and is Cys-rich (roughly 8% Cys residues). When the amino acid sequence of MVLG_6175 was compared to the genomes of *M. silenes-dioicae* and *M. violaceum sensu lato*, there was 95.76%, but only 59.83% identity, respectively, with the corresponding orthologues.

#### 2.4.4. MVLG_01732 Interacts with Host Proteins

Yeast two-hybrid screening with MVLG_01732 yielded 401 colonies after the initial stringent selection on QDO/X-α-gal + 3AT (5 mM) medium. Further selection to reduce leaky *HIS* selection yielded 65 colonies for examination via sequence analysis. From the 65 sequenced clones, yeast two-hybrid screening of MVLG_01732 revealed interesting host plant interactors. One of the interactors, represented by 52 clones, was found from blastp searches of the *Arabidopsis thaliana* genome (TAIR; https://www.arabidopsis.org/) as an orthologue of the AT3G61050.2 gene, which encodes a calcium-dependent lipid binding protein (AtCLB). AtCLB has both coiled coil regions and C2 domain similar to synaptotagmins, and synaptotagmins were also identified as hits in blastp searches of the ncbi database. Synaptotagmins are class of proteins with an N terminal transmembrane and two cytoplasmic C_2_ domains (Figure S1). The *S. latifolia* genomic region matching the AtCLB sequence is found on contig m.108787 (GenBank: FMHP01019528.1) in the *S. latifolia* genome assembly. It also corresponded to c85332_g3 RNA detected in RNA-Seq experiments [6,7].

The other interactor identified by yeast two-hybrid was cellulose synthase Interactive protein 1 (CSI1; Figure S2), represented by 13 clones. The *S. latifolia* genomic region matching this sequence is found on contig m.23209 (GenBank: FMHP01009449.1) in the *S. latifolia* genome assembly. It also corresponded to c93789_g3 RNA detected in RNA-Seq experiments [6,7]. Figure 1D shows that with 22.4% disordered residues, MVLG_01732 is the least disordered protein analyzed in this study. However, despite relatively low disorder content, MVLG_01732 contains MoRFs and, therefore, is expected to use intrinsic disorder for protein-protein interactions. The protein is 156 amino acids long and is not rich in Cys residues. When the amino acid sequence of MVLG_1732 was compared to the genome of *M. silenes-dioicae*, a 94.23% identity match was found in the corresponding orthologue, whereas only a 48.99% identity match was observed for the orthologue from *M. violaceum sensu lato*.

## 3. Discussion

**Figure 4:**
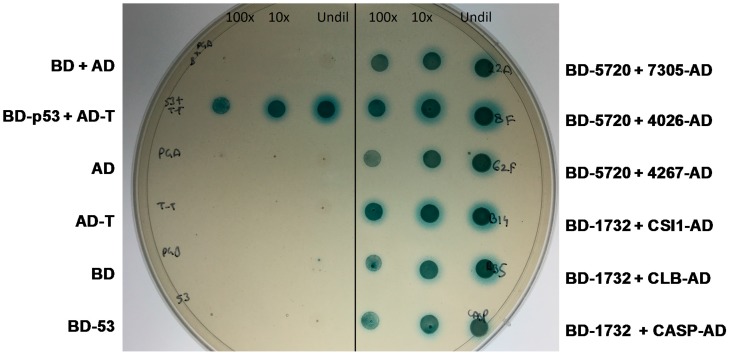
Yeast two-hybrid spot test results for all four proteins and positive and negative controls on quadruple drop-out medium (QDO)/X-α-gal + 3AT (5 mM) plates. For the spot test, each strain bearing the plasmid was grown in 3 ml of appropriate drop out liquid media at 30 °C and shaken at 280 rpm for 2 days. To reconfirm the interaction, 10 ΜL of each culture (washed and resuspended in 0.9% *w*/*v* NaCl) was mixed and spotted on QDO plates containing X-α-gal at the indicated dilutions and incubated at 30 °C for 3–5 days. Undil, undiluted; 10× and 100× dilutions.

In this study, we were able to predict from in silico analyses a conservative estimate of the secretome of *M. lychnidis-dioicae*. Furthermore, among this group, we identified candidate effectors as SSPs that were also highly expressed during plant infection. For the four putative effectors examined in greater detail in this study, amino acid sequence comparisons between *M. lychnidis-dioicae* and *M. silenes-dioicae* [34] revealed that these two organisms share close similarity in their predicted SSPs. In contrast, comparisons of most of the orthologues identified in *M. violaceum sensu lato* [35], the species that infects *Silene paradoxa*, had significantly lower amino acid similarities to those of the other species. These findings suggest that the latter organism has diverged substantially from the other two species, a finding supported by the phylogenetic relationships of the three respective fungal species [36] and the lack of cross-infectivity for *M. violaceum sensu lato* on either *S. latifolia* or *S. dioicae*; similarly, neither *M. lychnidis-dioicae* nor *M. silenes-dioicae* have been found to infect *S. paradoxa*. Of note, all SSPs were shown to contain IDPRs, and the vast majority of these proteins (>61.0%) were classified as mostly or highly disordered. Many SSPs were also predicted to have at least one MoRF, with some of the putative effectors possessing multiple MoRFs that can be utilized in promiscuous interactions with the fungal and host proteins. To test some of these predictions, a subset of the SSPs predicted in silico were confirmed to be secreted by YST experiment. We conducted yeast two-hybrid analysis for these SSPs to identify their host interactors and hence to understand their role in the mechanism of the infection (Figure 4).

Studies suggest that the proteins that undergo post translational modifications (PTMs) are considered to interact more with other proteins by engaging in more physical contacts and are known to be in the central network pathways more than non-PTM proteins. For example, all but four of the 49 secreted proteins we examined in detail were predicted to be targets for amidation. C-terminal amidation has been shown to be involved in membrane interactions for some proteins. In one case, an antimicrobial peptide, maximin H5, was able to penetrate and lyse erythrocyte membranes when amidated, but the ability to penetrate lipid membranes was severely reduced with deamidated peptide [37]. For the 4 SSPs, we examined in detail by yeast two-hybrid analysis, MVLG_04106 and MVLG_05720, which were predicted to have amidation targets (but, not at the C-terminus), while MVLG_06175 and MVLG_01732 each have a predicted target closer to the C terminus. If amidation plays a similar role for these SSPs as it does for maximin H5, this could indicate that these effectors penetrate host cells as part of their normal function.

### 3.1. MVLG_04106 Could Serve as a Transcriptional Regulator

The finding that MVLG_04106 was able to autoactivate all the reporter genes—*HIS3*, *ADE2*, and *MEL1*—in the yeast two-hybrid screen suggests its role as a transcriptional regulator. However, analysis by structural modelling reveals that a portion of this protein is similar to chorismate mutase, a vital enzyme that catalyzes the conversion of chorismate to prephenate in the shikimate pathway, leading to the production of aromatic amino acids, phenylalanine, and tyrosine, and regulating their balance. Chorismate also serves as a substrate for the production of salicylic acid (SA), which is a major signaling defense molecule in plants. This fungal protein chorismate mutase could deviate the flow of available chorismate for the production of prephenate and hence channel down its availability for SA production. In fact, studies show that *Ustilago maydis*, an obligate biotrophic pathogen that causes corn smut, also secretes an effector called Cmu1, a chorismate mutase taken up by plant cells and spread to adjacent cells causing metabolic priming in the infected cells [38]. However, if MVLG_04106 is a chorismate mutase, this still begs the question of how it autoactivates the reporter genes in yeast two-hybrid assay. Transcriptome analysis revealed that it is highly expressed during infection but not under in vitro conditions, all of which suggests its role in pathogenicity. Thus, further investigation is requited to better define its true role during infection.

### 3.2. MVLG_05720 Possibly Regulated by Additional Fungal Proteins

Three fungal proteins were identified as interactors with MVLG_05720: MVLG_07305, MVLG_04026, and MVLG_04267. None of the three were predicted via bioinformatic tools to be secreted. The differential expression data [6,7] indicated that MVLG_07305 was downregulated in late infection stages *in planta* and upregulated in mated conditions in vitro (while MVLG_05720 was downregulated during mating) [6]. Thus, MVLG_07305 may play some role in mating or in the transition to dikaryotic filaments. The gene is located on the mating-type chromosome, but its expression is similar in both a1 and a2 mating-type strains on either rich or nutrient-limited media [7]. Blastp predicted its function as a putative fimbrial outer membrane usher protein, containing a mannose binding domain. Of note, fimbrial appendages were first observed serendipitously on the haploid cells of an anther smut fungus [39]. They are involved in cell-to-cell communication and adhesion during mating before pathogenesis, as enzymatic and mechanical removal of these structures were shown to delay mating until the regeneration of fimbriae occurred [40,41].

The second fungal interactor, MVLG_04026, followed the same expression pattern as that of MVLG_07305; its predicted function was as a Fibrillin-like protein. Fibrillins are secreted proteins that constitute the backbone of extracellular macromolecular microfibrils [42]. The C terminus of fibrillins can undergo multimerization as a consequence of intermolecular disulfide bonding with itself or other proteins soon after secretion [43]. However, MVLG_04026 was predicted via bioinformatic tools not to be secreted. Its transcription was also upregulated during mating and downregulated during infection.

MVLG_04267 was not found to be differentially expressed under any of the conditions examined. It belongs to the DUF1212 superfamily, a class of membrane proteins with unknown function. Perhaps this protein plays a role in transport of MVLG_05720. If the MVLG_07305 and 4026 proteins are translated during mating and persist during infection, they may interact with MVLG_05720, to sequester it until it is needed for manipulation of the host. However, the mechanism of their action remains a mystery and requires further investigation.

### 3.3. MVLG_06175 Role in Host Entry During the Infection and in Reproduction

From yeast two-hybrid screening, the protein product of fungal gene *MVLG_06175* interacts with a host CASP-like protein 2C1 orthologue of *Spinacia oleracea* (LOC110788005), transcript mRNA (XM_021992637.1); it also matched CASP-like protein 2C1, AT4G25830.1 of *A. thaliana*. The corresponding transcript from *Silene* expression data (Toh et al. submitted) similarly matched the same *S. oleracea* protein. CASP-like proteins (CASPLs) are homologues of Casparian strip membrane domain proteins (CASPs). With respect to the functions of CASPLs, previous research showed CASPLs might function as protein barriers on the cell membrane of the endodermis and form protein scaffolds for the synthesis of the Casparian strip. Some CASPLs were shown to be expressed in the root endodermis, peripheral root cap, root meristem zone, trichomes, lateral root primordia, young leaves, and the floral organ abscission zone in *Arabidopsis thaliana* [44]. This last role, in floral organs, would be an appropriate target for a fungal effector from an anther smut. Alternatively, since CASPLs are orthologous with MARVEL domain proteins associated with the function of epithelial tight junctions [45], CASPLs might be related to tight junction functions in plant cells as well. Thus, the interaction between MVLG_06175 and CASPL2C1 of *Silene* could indicate that *Microbotryum* alters the functions of tight junctions to enter the host tissues during infection.

MVLG_06379 was also found as a fungal interactor of MVLG_06175. MVLG_06379 contains a PFAM domain (PF03328.7) for ATP citrate lyase (ACL) β subunit. The enzyme converts cytosolic citrate into acetyl-CoA for further fatty acid synthesis, but in the parasitic fungi the transcription and translation of *ACL* appears to be associated with infection and reproduction. *Cryptococcus neoformans* increased transcriptional level of *ACL1* within macrophages. Additionally, mutants lacking *ACL1* showed higher susceptibility to antifungal drugs, a lower survival rate within macrophages, and defects in expression of virulence factors [46].

### 3.4. MVLG_01732 Role in Altering the Vesicular Traffic in the Host and Male Sterility

In blastp analyses searching the *Arabidopsis* genome we found this interactor as the orthologue of the AT3G61050.2 gene. This encodes a calcium-dependent lipid binding protein (AtCLB) that has both coiled coil regions and C2 domain (see Figure S1) similar to synaptotagmins. Many coiled coil proteins are involved in regulating gene expression as transcription factors. The motif is present in the nucleotide binding site leucine rich repeat (NBS-LRR) proteins of R genes. *Arabidopsis* encodes 150 NBS-LRR-type proteins and they are either the coiled coil (CC) type or the TIR type [47]. C2 domains in animal cells are involved in signal transduction and vesicle trafficking, but in plant cells they are not well characterized. They could be involved in plant stress signal transduction as positive or negative regulators of stress signaling cascades (Figure S3 for predicted interaction partners of AtCLB). AtCLB expression is highly detected in rosette leaves and flowers and low in roots, stems, and cauline leaves. Transcriptome analyses studies on pollen germination and tube growth shows its expression in mature pollen, hydrated pollen, and pollen tube growth, which suggest its role in the development of the male gametophyte [48]. Studies show that AtCLB acts as a DNA-binding protein, and binds specifically to the promoter sequence of Thalional synthase 1 (*THAS1*), a key enzyme in the synthesis of the triterpenoid, thalionol. AtCLB negatively regulates *THAS1* transcription, as part of a response involved in drought and stress tolerance. AtCLB becomes localized on the nuclear membrane and can bind to ceramides, a glycolipid present in cellular membranes that acts as a second messenger in cell signaling, cell differentiation, and apoptosis [45]. Since it is a membrane protein, its activation by membrane lipid ceramide could result in a proteolytic cleavage and translocate it to nucleus to activate transcription of a different set of genes [49]. Interestingly, analysis of the amino acid sequence showed that there are two proteolytic cleavages at positions 28 (after the transmembrane region (1–22)) and 383 (close to where the C2 domain (264–361) ends and the coiled coil region (390–417) begins). Although this is purely speculative at this point, if the MVLG_01732 effector were to become intracellular, upon Ca^2+^ triggering due to conformational changes, the AtCLB protein could interact with the effector at the coiled coil region to mediate AtCLB activation by the membrane lipid ceramide resulting in proteolytic cleavage after the TM to yield mature protein and translocation to the nucleus to regulate transcription of target genes.

In blastp analyses against the NCBI database, the same host interactor for MVLG_01732 matched a portion of the C_2_ domain and mostly the C-terminal coiled coil region of synatotagmin-5 of *Beta vulgaris* subsp. *vulgaris* (LOC104905441), transcript variant X2, mRNA (XM_010693990.2); the corresponding transcript from *Silene* expression data (Toh et al. submitted) similarly matched the same *B. vulgaris* protein. Synaptotagmins are a family of membrane proteins concentrated on secreted vesicles, including synaptic vesicles. They are composed of a short uncleaved *N*-terminal signal peptide that overlaps a transmembrane (TM) domain, a synaptotagmin-like mitochondrial and lipid-binding protein (SMP) domain, and two tandem cytosolic calcium binding domains (C_2_A and C_2_B) at the C-terminus required to bind to phospholipids or different ligands in response to calcium signals [50]. Ca^2+^ plays an important role as a second messenger in response to variety of stimuli like cold, drought, salt, oxidative, and biotic stress. Ca^2+^ binding confers two roles in membrane targeting process. One is to provide a bridge between C_2_ domain and anionic phospholipids, and the second is to induce intra or inter domain conformational changes, which further triggers membrane protein interactions.

The second host interactor for MVLG_01732 matched the C_2_ domain of Cellulose synthase interactive protein 1 (CSI1) of *Spinach oleracea* (Accession number: XP_021846375) and *Beta vulgaris* (Accession no: XP_010680591), orthologues of *Arabidopsis* AT2G22125.1 gene. Again, the C_2_ domain (Figures S1 and S2) is a Ca^2+^ binding motif originally identified in Protein kinase C [51]. However, not all C_2_ domains are regulated by Ca^2+^, with some functioning in a Ca^2+^-independent manner and others having mainly a structural role. C_2_ domains interact with cellular membranes and mediate key intracellular processes like insulin secretion and neurotransmitter release in eukaryotic cells. It binds to a multitude of different ligands and substrates that include Ca^2+^, inositol polyphosphates, intracellular proteins, and phospholipids.

Mutant analyses found that CSI physically interacts with microtubules and plays a crucial role in anther dehiscence. This is interesting because the events leading to anther dehiscence are coordinated with pollen differentiation, flower development, and opening for successful pollination. CSI 1 disruption mutants exhibited complete sterility and defective anther dehiscence, with crumpled pollen and defective pollen release from the anther. Moreover, such mutants had morphological changes in the epidermal and endothecial cell length and width necessary for anther maturation, indicating the reason for defective dehiscence may be due to unstable microtubules. CSI mutants also exhibited altered sensitivity to exogenous Ca^2+^ levels, which indicates that there is Ca^2+^-mediated regulation in microtubule stability and anther dehiscence [52]. Of note, CSI 1 mutants also exhibited decreased number of ovules per gynoecium but were viable, indicating an additional effect of CSI in early gynoecial development.

We hypothesize that the fungal effector MVLG_01732 modulates the function of CSI1 by interacting with the C_2_ domain (suggested by our yeast two-hybrid results), thereby altering the stability of microtubules, resulting in delayed anther development and dehiscence. This could provide the fungus an opportunity to hijack anther development, replacing the pollen grains with its teliospores. Studies show that calcium binding proteins and calcium dependent signaling are involved in both the development of embryo sacs and during the development of pollen [53]. In both the host interactors we identified, the MVLG_01732 effector binding could modulate C_2_ domains and their interaction with Ca^2+^, triggering several signaling pathways for the benefit of the fungus.

In sum, in silico analyses predicted a number of fungal small secreted proteins that could serve as effectors to modulate the plant host. For a subset of these, we identified the host interactors that are candidates for targets of these effectors. Recognizing that, even with appropriate controls, yeast two-hybrid analysis can give false positive results, we are planning to further verify the predicted interactions in future experiments using co-immunoprecipitation from infected plants. There is also a need to characterize the function of these interactors and their roles in the plant. Future experiments to express the fungal effectors in transgenic plants might recapitulate phenotypes observed during infection. Although the natural host, *S. latifolia*, currently lacks a transformation system, heterologous plant systems like *A. thaliana* are amenable for such experiments and should help in the characterization of these fungal proteins. Moreover, expressing the fungal proteins with a fluorescent marker like GFP or mCherry would help determine the localization of these fungal proteins inside the host. Pull down assays can also be conducted to identify additional host proteins, if any, that may have been missed by yeast two-hybrid analysis. This experimental model could then be expanded in the future to provide mechanistic insights into the interplay of this biotrophic pathogen with its host.

## 4. Materials and Methods

### 4.1. Plant and Fungal Growth

*Silene latifolia* seeds that were used in this study were originally collected from a field population in Clover Hollow near Mountain Lake Biological Station, Virginia. Sterilized seeds were plated on sterile 0.3% phytagar (Life Technologies/Thermo Fisher, Waltham, MA, USA), one half strength Murashige and Skoog salts (Sigma Aldrich, St. Louis, MO, USA), and 0.05% MES (2-(*N*-morpholino) ethanesulphonic acid) buffer (Sigma-Aldrich). Seeds were kept at 4 °C for 5 days to encourage germination and then were transferred to a 20 °C growth chamber with 13 h of fluorescent light. Humidity was kept high initially by using dome covers and flood trays, and was gradually decreased to lower levels. Seedlings were transplanted to bigger pots for the emerging new roots to provide hydration requirement when the volume of soil was not sufficient. Plants were grown in Sunshine MVP professional growing mix (Sun gro Horticulture Canada Ltd, cat no: 02392868, Agawam, MA, USA) and were watered every other day with 100-ppm fertilizer (Peters Professional 15-16-17 Peat-Lite Special, Formula no: S12893, JR Peters, Inc. Allentown, PA, USA) [7].

Fungal strains of *M. lychnidis dioiceae*, p1A1 and p1A2, were axenically grown separately on nutrient rich media (yeast peptone dextrose media (YPD); 1% yeast extract, 10% dextrose, 2% peptone, and 2% agar) at 28 °C for 5 days and nutrient-free water agar media for 2 days (2% water agar).

Plant infection employed haploid *M. lychnidis-dioicae* p1A1 and p1A2 cells that were grown on nutrient rich media (YPD; 1% yeast extract, 10% dextrose, 2% peptone, and 2% agar) at 28 °C; these were harvested and adjusted to a concentration of 1x10^9^ cells/mL in equal proportion before being spotted onto nutrient free media (2% agar). Once conjugation tubes were identified microscopically, the cells were resuspended to a concentration of 1 × 10^6^ in distilled water. Then, 5 µL of this was dropped onto the floral meristem of 11–12 day old *S. latifolia* seedlings [7].

### 4.2. In Silico Analyses

#### 4.2.1. Prediction of Small Secreted Proteins (SSPs)

**Figure 5:**
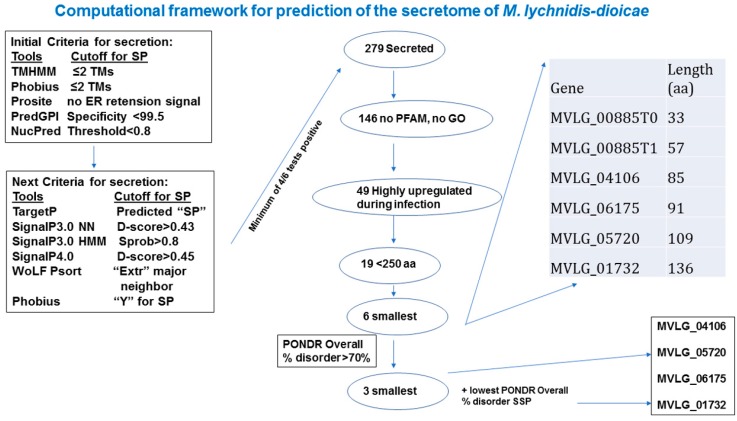
Computational framework for prediction of secretome for *M. lychnidis-dioicae* and selection of candidate effectors for further analyses. Detailed description of tools and cut-off criteria for secretome prediction and prediction of disorder are provided in Supplementary Methods. Numbers tally for proteins at each stage of secretome prediction are provided in tab 3 of Table S1. TM, trans-membrane domain; ER, endoplasmic reticulum’ SP, secreted protein; PFAM, protein family; aa, amino acid; GO, gene ontology; MVLG designations refer to specific *Microbotryum lychnidis-dioicae* proteins.

Prediction of the secretome used a pipeline of software packages (TargetP1.1, SignalP3.0, SignalP4.0 (http://www.cbs.dtu.dk/services/SignalP/), TMHMM2.0, PredGPI, Phobius, NucPred, Prosite, and WoLF PSORT) to provide a stringent determination of likely secretion [7] (Table S1 and Figure 5).

#### 4.2.2. Prediction of Intrinsic Disorder

In order to analyse the residue level of disorder propensity of 49 putative effector proteins, four intrinsic disorder predictors were used: PONDR^®^ VSL2 [18], PONDR^®^ VLXT [19], PONDR^®^ VL3 [20], and PONDR^®^ FIT [21]. While evaluating the intrinsic disorder predisposition of four SSPs targeted for functional analysis (MVLG_01732, MVLG_04106, MVLG_05720, and MVLG_06175), in addition to the members of the PONDR family, IUPred_short and IUPred_long were used [24].

Molecular recognition features (MoRFs) are short segments with increased order propensity located within longer disordered regions. MoRFs bind to globular protein domains and undergo disorder-to-order transition. These disorder-based binding sites are categorized into three types: α-MoRFs (form α-helices upon binding), β-MoRFs (form β-strands), and ι-MoRFs (form irregular structures). For all 49 predicted secreted proteins whose transcription was upregulated during infection, the ANCHOR algorithm (http://anchor.enzim.hu/) was used to predict such protein binding regions that are disordered in isolation but can undergo disorder-to-order transition upon binding [22]. This computational tool finds segments within disorder regions that cannot form stable intra-chain interactions to fold on their own, but are likely to gain stabilizing energy by interacting with a globular protein partner [22]. Furthermore, the presence of MoRFs in MVLG_01732, MVLG_04106, MVLG_05720, and MVLG_06175 was further evaluated by another computational tool, MoRF_chibi_ [54].

#### 4.2.3. Additional Bioinformatic Analyses

Alignment of nucleotide and/or amino acid sequences to find regions of similarity between such biological sequences employed Basic Local Alignment Search Tool (BLAST; https://blast.ncbi.nlm.nih.gov/Blast.cgi). Further domain analysis for prediction of post translational modification sites used ModPred [33]. Structural modelling of predicted proteins utilized Swiss-Model [55,56]. Results of these analyses are found in Table S2. Additional analysis methods are provided in Supplementary Materials and associated references [57,58,59,60].

### 4.3. Yeast Secretion Trap (YST) Experiment

For each candidate effector, validation of secretion employed a yeast-based secretion trap method [28]. Putative secretion signals for each fungal gene were cloned into the pYSTO-0 vector. In such analyses, if the putative signal peptide from a protein provides for secretion of the Suc2p invertase, *S. cerevisiae* cells will be able to grow on sucrose as a sole carbon source; inability to promote growth would indicate that the fungal protein of interest is not normally secreted.

The signal peptide sequence of each fungal protein was determined by Signal P software and amplified by PCR. Standard PCR cycle was used with initial denaturation set at 94 °C for 4 min and 35 cycles of 94 °C for 30 s, 60 °C for 30 s, and 72 °C for 30 s, with a final extension time of 5 min at 72 °C. The product was held at 4 °C at the end of the cycle.

The PCR products were separated by gel electrophoresis through 1.8% agarose (Agarose LE; USB Corp., Cleveland, OH, USA). The fragments were excised from the gel and purified using the Zymo Gel DNA recovery kit (Orange, CA, USA). The purified fragments were subjected to restriction digestion with *Eco*RI and *Not*I enzymes. The digested fragment was purified and cloned into the pYST-0 vector to obtain a translational fusion with the invertase expressed from the *ADH1* promoter and transformed into *Escherichia coli* DH5 α cells. Cells were plated on LB plates with ampicillin (100 mg·L^−1^) and incubated at 37 °C overnight. *E. coli* strain, DH5 α (Bethesda research Laboratories, Bethesda, MD, USA), was utilized for all cloning purposes. *E. coli* strains were grown at 37 °C in Circle Grow media (MP Biomedicals, LLC, Solon, OH, USA) and plasmid DNA was isolated from potential clones using the alkaline lysis procedure [61]. The presence of each signal peptide encoded in-frame with the *SUC2* coding region was confirmed by DNA sequencing at the Nucleic Acids Core Facility (Center for Genetics and Molecular Medicine, University of Louisville, Louisville, KY, USA).

Invertase-deficient (suc2^−^) *S. cerevisiae* strain (SEY 6210 (MATαleu2-3, 112 ura3-52 his-Δ200 trp1-Δ901 lys2-801 suc2^−^ Δ9 GAL)) [62] cells were transformed with the constructs using the lithium acetate/single-stranded carrier DNA/PEG method [63]. Selection was on Synthetic Dropout medium, with SD/-Leu (Clontech, Mountain View, CA, USA) selection plates containing glucose as the sole carbon source. The dropout medium contained glucose (20 g·L^−1^), yeast nitrogen base (6.7 g·L^−1^), dropout mix minus leucine (2 g·L^−1^), agar (15 g·L^−1^), and water. The plates were incubated at 30 °C for 6–10 days. The colonies were restreaked for purification onto SD/-Leu drop out selection plates with sucrose as the sole carbon source to select the positive clones that were able to utilize sucrose by secreting invertase enzyme. Such strains were grown overnight in 3 mL of SD/-Leu broth with sucrose, and 10-fold dilutions were spotted onto SD/-Leu with glucose or sucrose as the carbon source and incubated for 5 days at 30 °C. Clones harboring functional signal peptides with reconstituted invertase activity were able to grow on sucrose as the sole carbon source. Untransformed mutant yeast strain SEY 6210 and the same strain, transformed with empty pYST-0 vector, were used as negative controls. Plasmid DNA was extracted from the positive clones and used to retransform *E. coli*. The constructs were again checked for the presence of signal peptide sequence by DNA sequencing.

### 4.4. RNA Extraction and cDNA Library Construction

RNA for generating the cDNA library was obtained from the axenically grown cultures of p1A1 and p1A2 haploid strains [7] on nutrient rich media for 5 days (YPD) at 28 °C, nutrient-free water agar media (2% water agar) for 2 days, and the fungal infected *Silene latifolia* tissue [7]. This latter set of RNAs was extracted from floral stem (pedicle, and remaining cluster and sepals), floral buds (male and female) at different stages (male: 2–6 mm buds, 8 mm to fully opened smutted flowers; female: 3–6 mm, 7–14 mm, and 15–24 mm). The quality of the RNA was checked by Agilent Bioanalyzer and all the samples indicated highly intact RNA with the RNA integrity scores of at least 7.8. The total samples were pooled equally based on Bioanalyzer quantification to generate a normalized cDNA library. The cDNA library was constructed in a Gal4 based prey vector, pGADT7 (Clontech), by CD Genomics (Shirley, NY, USA) for yeast two-hybrid screening.

### 4.5. Yeast Two Hybrid Screen

The yeast two-hybrid system allows for an initial screening of possible protein-protein interactions [64,65]. A “bait” protein of interest is expressed from a yeast (*Saccharomyces cerevisiae*) expression vector as a fusion with the Gal4 DNA binding domain (BD). Interactors with bait are identified by screening “prey” expressed from a yeast vector where the fusion is with the Gal4 transcriptional activation domain (AD). pGBKT7 was used as a “bait” vector with the GAL4 DNA-binding domain and pGADT7 was used as a “prey” vector with the GAL4 DNA activation domain. While neither the BD, nor AD alone, can activate transcription of the reporter genes used in this system, if two proteins physically interact (i.e., if prey Y interacts with bait X), then the BD and AD are brought together and reporter genes will be expressed. In our studies, the prey proteins were all expressed from normalized cDNA libraries of the different stages of *M. lychnidis-dioicae,* including in association with its host, *S. latifolia*. Initial selection of interactors involves ability to grow on increasingly more stringent auxotrophic media, since the yeast strains have auxotrophic mutations that require them to either be provided with the missing nutrients or to have a functional interaction that activates transcription of reporter genes whose read-out is complementation of the growth defect. Additionally, an α-galactosidase gene serves as a reporter, whereby color change to blue-green occurs via cleavage of 5-bromo-4-chloro-3-indolyl α-d-galactopyranoside (X-α-gal) in the medium. In order to avoid false positives, a number of controls were employed, including comparisons using (1) vectors alone/without bait or prey (i.e., pGBKT7 or pGADT7, respectively); (2) bait in BD vector alone; (3) prey in AD vector alone; (4) re-transformation of yeast strains with identified interactors and bait; (5) repetition of the experiment with vectors, in which the bait has been fused to AD and the identified prey interactor has been fused to the BD, so as to avoid artifacts associated with the particular fusion used originally. The interaction of pGBKT7-53 (containing p53 coding sequence) and pGADT7-T (containing T antigen coding sequence) was used as a positive control. Only those candidate interactors that passed these stringent tests were considered worthy of further investigation.

The coding sequences of each of the effector candidates, lacking signal peptides and stop codon, were PCR amplified using cDNA as template, generated from fungal infected *S. latifolia* floral buds, using the primer pairs described in Table S3. The effector candidates tested in this study were MVLG_004106, MVLG_005720, MVLG_06175, and MVLG_001732, for which sequences are available in the JGI Fungal Genome database [66]. The PCR products were cloned into the pCR 2.1 TOPO entry vector (Invitrogen/Thermo Fisher, Waltham, MA, USA). *Escherichia coli* strains, DH5 α (Bethesda research Laboratories, Bethesda, MD, USA), were utilized for all cloning purposes. Plasmid DNA was isolated and the inserts were digested out of this vector with *Eco*RI and *Bam*HI. Purified fragments were subsequently cloned into a pGBKT7 destination vector (Clontech) where transcription of the cloned gene would be driven by an *ADH1* promoter, producing fusion proteins at their N termini with the DNA binding domain of the Gal 4 transcription factor.

*S. cerevisiae* strain Y187 (Library host strain) (MATα, ura3-52, his3-200, ade2-101, trp1-901, leu2-3, 112, gal4Δ, met–, gal80Δ, URA3 : : GAL1_UAS_-GAL1_TATA_-lacZ) [67], containing the *MEL1*/*lacz* reporter gene, was transformed with the prey vector containing the cDNA library using the Frozen-EZ Yeast Transformation II kit (Zymo Research) and selected on SD drop out medium lacking Leucine (SD/-Leu). MELIBIASE1 *(MEL1*) reporter gene encodes α-galactosidase and enables yeast cells to turn blue-green in the presence of the chromogenic substrate, 5-bromo-4-chloro-3-indolyl α-d-galactopyranoside (X-α-gal). Cell density of the library was calculated by tittering 10^−4^, 10^−5^, 10^−6^, and 10^−7^ dilutions on SD/-Leu plates.

The AH109 yeast strain (Mating partner) (MATa, trp1-901, leu2-3, 112, ura3-52, his3-200, gal4Δ, gal80Δ, LYS2::GAL1_UAS_-GAL1_TATA_-HIS3, GAL2_UAS_-GAL2_TATA_-ADE2, URA3::MEL1_UAS_-MEL1_TATA_-lacZ) [68], containing *HIS3*, *ADE2*, and *MEL1/lacz*) reporter genes, was used as the host for the bait constructs. The *HIS3*, *ADE2* reporter gene products enable the cells to biosynthesize required nutrients to grow on plates lacking histidine and adenine. The three reporter genes are under the control of distinct *GAL4* upstream sequences and promoter elements *GAL1*, *GAL2,* and *MEL1,* respectively, yielding strong and specific responses. In AH109, the entire *HIS3* promoter (including both TATA boxes) was replaced by the entire *GAL1* promoter, leading to tight regulation of the *HIS3* reporter gene in this strain. The bait constructs were transformed into AH109 by the lithium acetate/single-stranded carrier DNA/PEG method [63] and selected on SD drop out medium lacking Trp.

The yeast two-hybrid screening was conducted following the Matchmaker Library Construction and Screening Kits User manual (Clontech). Initial screening was conducted on high stringent quadruple drop out media (QDO) SD/-Ade/-His/-Leu/-Trp plates with X-α-gal and 5 mM 3AT. Subsequently, colonies were restreaked onto QDO/X-α-gal plates with 5 mM 3AT initially, and then on to QDO/x-α-gal plates containing 50 mM 3AT to select strong interactors. 3AT was used to inhibit the leaky expression that reduces the effectiveness of histidine selection, and to inhibit X-α-gal to allow detection of the *MEL1*/*lacz* reporter. 3-AT is a competitive inhibitor of the yeast HIS3 protein (His3p), blocking low levels of His3p expression, and thus suppressing background growth on SD medium lacking His. Only the positive blue-green clones (indicating α-galactosidase activity) that survived on the highest 3AT levels were used for further screening. To estimate the mating efficiency and to calculate the total number of screened colonies dilution serials were prepared and 100 µL of each dilution was spread on SD/-Trp, SD/-leu, and DDO plates. Plasmids were isolated from the surviving colonies and were individually used to transform *E. coli*. The prey plasmids were isolated from *E. coli*, and sequenced and analyzed by BLAST screens against the NCBI database [69]. The dropout medium contained Glucose (20 g·L^−1^), yeast nitrogen base (6.7 g·L^−1^), appropriate dropout mix (2 g·L^−1^), agar (15 g·L^−1^), and water.

## 5. Conclusions

In this paper we identified, for the first time, the interactors of the putative effectors of *M*. *lychnidis*-*dioicae*. We believe that the protein product of MVLG_04106 codes for a transcriptional regulator/activator of host responses to allow successful infection. The fungal interactors of MVLG_05720 protein product, MVLG_07305 and MVLG_04026, could potentially sequester this effector until it is required during infection. MVLG_06175 appears to interact with a CASP-like homologue and may be involved in cell-cell junctions. The identification of two host interactors of MVLG_01732-AtCLB and CSI I, which play roles in anther/pollen development and dehiscence, provides exciting targets for future studies, as we hypothesize this effector may be crucial in redirecting anther and pollen development in such a way as to benefit the reproductive program of the fungus. Plant infection studies with knockouts or over expression of these effector genes will further our understanding in characterizing the function of these key players in the infection. This strongly suggests the need to also characterize the remaining candidate effector proteins for a more complete understanding of the mechanisms of infection and development of this fascinating plant parasite.

## Figures and Tables

**Table 1:** Candidate SSPs chosen for further analyses.

Predicted Protein	Expression ^a^	Size (Amino Acids)	No. of Cys	Function
MVLG_01732	144 rsem vs. 0	156	1	Candidate effector
MVLG_04106	86 rsem vs. 0	107	6	Candidate effector
MVLG_05720	1164 rsem vs. 0	129	12	Candidate effector
MVLG_06175	127 rsem vs. 0	118	10	Candidate effector

^a^ rsem normalized counts for infected male *S. latifolia* vs. expression in YPD or nutrient-limited agar [7].

## Supplementary Materials

Supplementary materials can be found at www.mdpi.com/1422-0067/18/11/2489/s1.
