# Supplementary material for: Identification and Initial Characterization of the Effectors of an Anther Smut Fungus and Potential Host Target Proteins
Source: Int J Mol Sci. 2017 Nov 22;18(11):2489. doi: 10.3390/ijms18112489 (PMC5713455; doi:10.3390/ijms18112489)
Supplement: Supplementary file 1 [file ijms-18-02489-s001.zip › ijms-240699-proof 2 done suppl. .pdf]

Supplementary Methods

Prediction of secreted proteins

Prediction of secreted proteins was performed using a combination of online tools: TargetP1.1, SignalP3.0, SignalP4.0, TMHMM2.0, PredGPI, Phobius, NucPred, Prosite and WoLF PSORT.

The initial absolute cutoffs for excluding non-secreted proteins are listed in the following table.

Criteria for secretome discovery.

**Tools**	**Cutoff for non-SP**	**Remain in pool of probable secretome**
TMHMM	>2 TMs	≤2 TMs
Phobius	>2TMs	≤2 TMs
Prosite	ER retention signal: 00014	-
PredGPI	Specificity of >99.5% using the General Model	Specificity of <99.5, where 99.0-99.5 is characterized as lowly probably for GPI linkage
NucPred	Threshold>0.8	Threshold<0.8

The following guidelines were then used in determining whether the protein was secreted or not. Out of the 6 tests for indicating SP localization, at least 4 tests should be positive for SP in order to remain in the probable SP pool.

Further guidelines for secretome discovery on the remaining pool of probable secretome.

**Tools**	**Guideline for SP**
TargetP	Predicted localization to “SP”
SignalP3.0 NN	D-score>0.43
SignalP3.0 HMM	Sprob>0.8
SignalP4.0	D-score>0.45
WoLF Psort	“Extr” listed as major neighbor
Phobius	“Y” for SP

Where there are ambiguous predictions, TMHMM and Phobius have to agree on the existence of 1-2 TM(s) on the protein to exclude it from the probable secretome pool. In addition, if a protein is predicted with “lowly probable” GPI linkage, but has TM predicted by TMHMM and/or Phobius around the same region, they serve as corroborating evidence for anchorage to the membrane

Where there were contractions or insufficient evidence for determining secretome status, BLASTp and Pfam domains is used to establish probable orthologs, followed by referencing the UniProtKB and FunSecKB database for confirmation of localization of the orthologs, where available.

<u>Analysis of intrinsic disorder predisposition and presence of functional sites in proteins encoded by 49 MVLG genes</u>

For each protein, we present:

1) MVLG ID and disorder content evaluated by PONDR VSL2 [67].
2) Amino acid sequence. Position of secretion signal peptide found by SignalP4.1 [68] are shown by red bold font.
3) Results of intrinsic disorder predisposition analysis by four predictors of PONDR family, PONDR® VSL2 [67], PONDR® VLXT [69], PONDR® VL3 [70], and PONDR® FIT [71].
4) PONDR® VSL2 disorder prediction statistics including location of predicted disordered regions.
5) Results of evaluation of the presence and localization of disorder-based binding sites conducted using the ANCHOR algorithm [72, 73].
6) Presence of possible post-translational modification sites found by ModPred [74]. Prediction results with High Confidence are listed.
7) Presence of possible functions found by PROSITE [75, 76].
8) Molecular models built using SWISS-MODEL [56, 57].

References

18. Peng, K.; Radivojac, P.; Vucetic, S.; Dunker, A.K.; Obradovic, Z. Length-dependent prediction of protein intrinsic disorder. *Bmc Bioinformatics* **2006**, *7*.
19. Romero, P.; Obradovic, Z.; Li, X.H.; Garner, E.C.; Brown, C.J.; Dunker, A.K. Sequence complexity of disordered protein. *Proteins-Structure Function and Genetics* **2001**, *42*, 38-48.
20. Peng, K.; Vucetic, S.; Radivojac, P.; Brown, C.J.; Dunker, A.K.; Obradovic, Z. Optimizing long intrinsic disorder predictors with protein evolutionary information. *Journal of bioinformatics and computational biology* **2005**, *3*, 35-60.
21. Xue, B.; Dunbrack, R.L.; Williams, R.W.; Dunker, A.K.; Uversky, V.N. Pondr-fit: A meta-predictor of intrinsically disordered amino acids. *Bba-Proteins Proteom* **2010**, *1804*, 996-1010.
22. Dosztanyi, Z.; Meszaros, B.; Simon, I. Anchor: Web server for predicting protein binding regions in disordered proteins. *Bioinformatics* **2009**, *25*, 2745-2746.
23. Meszaros, B.; Simon, I.; Dosztanyi, Z. Prediction of protein binding regions in disordered proteins. *Plos Computational Biology* **2009**, *5*.
33. Pejaver, V.; Hsu, W.L.; Xin, F.; Dunker, A.K.; Uversky, V.N.; Radivojac, P. The structural and functional signatures of proteins that undergo multiple events of post-translational modification. *Protein Sci* **2014**, *23*, 1077-1093.
57. Petersen, T.N.; Brunak, S.; von Heijne, G.; Nielsen, H. Signalp 4.0: Discriminating signal peptides from transmembrane regions. *Nat Methods* **2011**, *8*, 785-786.
58. Sigrist, C.J.; de Castro, E.; Cerutti, L.; Cuche, B.A.; Hulo, N.; Bridge, A.; Bougueleret, L.; Xenarios, I. New and continuing developments at prosite. *Nucleic Acids Res* **2013**, *41*, D344-347.
59. de Castro, E.; Sigrist, C.J.; Gattiker, A.; Bulliard, V.; Langendijk-Genevaux, P.S.; Gasteiger, E.; Bairoch, A.; Hulo, N. Scanprosite: Detection of prosite signature matches and prorule-associated functional and structural residues in proteins. *Nucleic Acids Res* **2006**, *34*, W362-365.
60. Berardini, T. Z., Reiser, L., Li, D., Mezheritsky, Y., Muller, R., Strait, E., and Huala, E. (2015) The Arabidopsis information resource: Making and mining the "gold standard" annotated reference plant genome. *Genesis (New York, N.Y. : 2000)* **53**, 474-485

Table S1: List of Predicted Secreted Proteins (see separate Excel File)

Table S2: Mostly disordered proteins (>50% disordered residues by PONDR® VSL2 analysis; percent disordered indicated in parentheses after the protein name; amino acids shown in red indicate the predicted signal peptide)

> MVLG_01284T0 (94.01%)- 217aa

MLMLKSLSVLIVAASAAHALQSPAAASNLGERGLTDGTTGLLDNLPGLPGLGDLLGGGGKTTTGK
VKRELDDFVSDTNSATPVKSPPGTDSALNDVTSDHNVLPQDAGLESLIPGLHKRQDEDEVDQDEDD
VDSNSGLDAGAEVDADFDLVRRGYRKASEVKFKVNEPKPATFVKKSKKHPKTADKEHGKKHDGV
HKKPDEEHKKHDEVPKKHHPVA

PONDR:

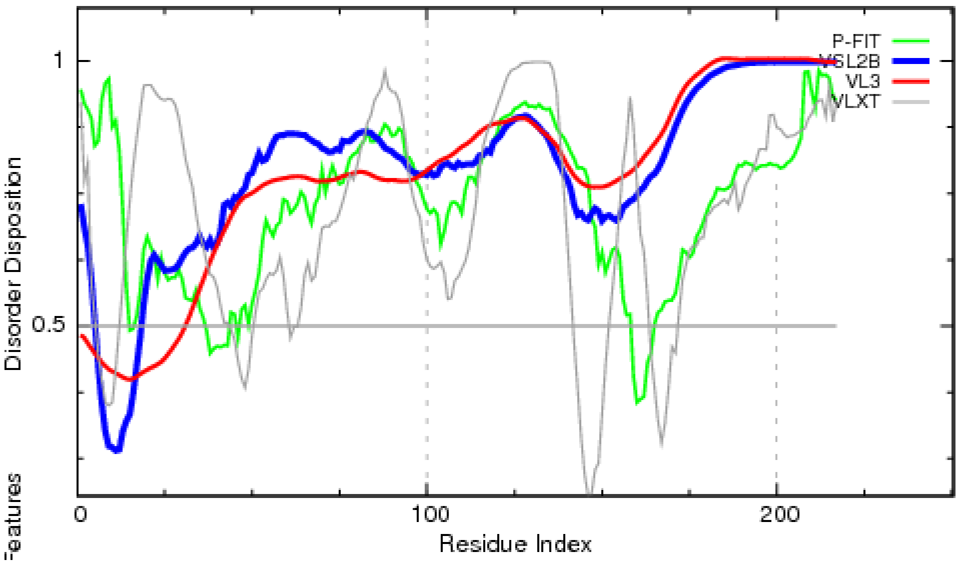

===============================PONDR VSL2 STATISTICS===============================

Predicted residues: 217
Number residues disordered: 204
Overall percent disordered: 94.01
Predicted disorder segment [1]-[5]
Predicted disorder segment [19]-[217]

Number Disordered Regions: 2
Longest Disordered Region: 199
Average Prediction Score: 0.7951
Average Strength= 0.6332
Average Strength= 0.8290

ANCHOR:

Predicted Disordered Binding Regions			
	From	To	Length
1	41	56	16
2	68	78	11
3	91	118	28
4	136	178	43
5	209	217	9
**Filtered Regions**			
	From	To	Length

1	1	14	14
2	192	192	1

ModPred and PROSITE:

ModPred: Sumoylation (K5), Amidation (G37, F73, P86, D128, F148, E160, E208), ADP-ribosylation (R68), Phosphorylation (T81), Proteolytic cleavage (D133, R152, K162, T172, H192, D193).

PROSITE: No identified domain recognition sites.

Structural modelling:

	Name	Title	Identity	Method	Oligo State	Ligands
☐	5bs7.1.F	Protein SPT2 homolog	26.83	X-ray, 3.3Å	hetero-oligomer	None
☐	5g1k.1.B	THIOL DISULFIDE INTERCHANGE PROTEIN DSBG	30.30	X-ray, 2.0Å	homo-dimer	None
☐	1ywf.1.A	PHOSPHOTYROSINE PROTEIN PHOSPHATASE PTPB	23.08	X-ray, 1.7Å	monomer	None

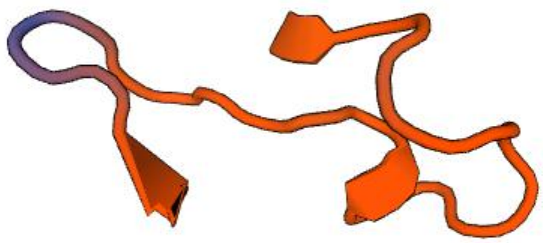

Model #1: Residues 43-68 of MVLG_01284T0 with 1ywf.1.A (23.08 % sequence identity) as a template

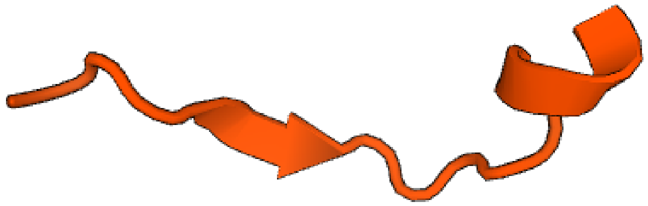

Model #2: Residues 138-153 of MVLG_01284T0 with 5bs7.1.F (26.83 % sequence identity) as a template

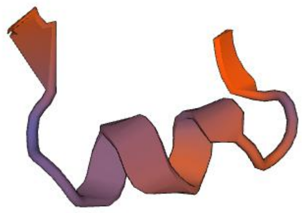

Model #3: Residues 21-35 of MVLG_01284T0 with 5g1k.1.B (30.30 % sequence identity) as a template

> MVLG_00385T0 (90.32%)

MHIHFFTVLSASALLALSHAAPTPGGPSSVHNVSPKYGPDSKCFHYYFDHLDEYKPKYGCQDYEQ
YKCSYADAYYVKKKAEQDKKEAECKKQDYDFKKHTWHYTSVKNSFEEEKKKYNALLKQYNLET
TRYEEHKKNYEAFKRAREEENRKNEETKKYCEKVFEEVKEYFKPKQSYHIDGHQNSHGGGNLKGD
GKGFAGKKDEDGEKEGGHGGVKNVGGKDGGKGRAKDGKDGKLDDKDEHDGKDGHDGKASKIH
GKKDEKCHDEKKDFSSGGNPKSGW

PONDR:

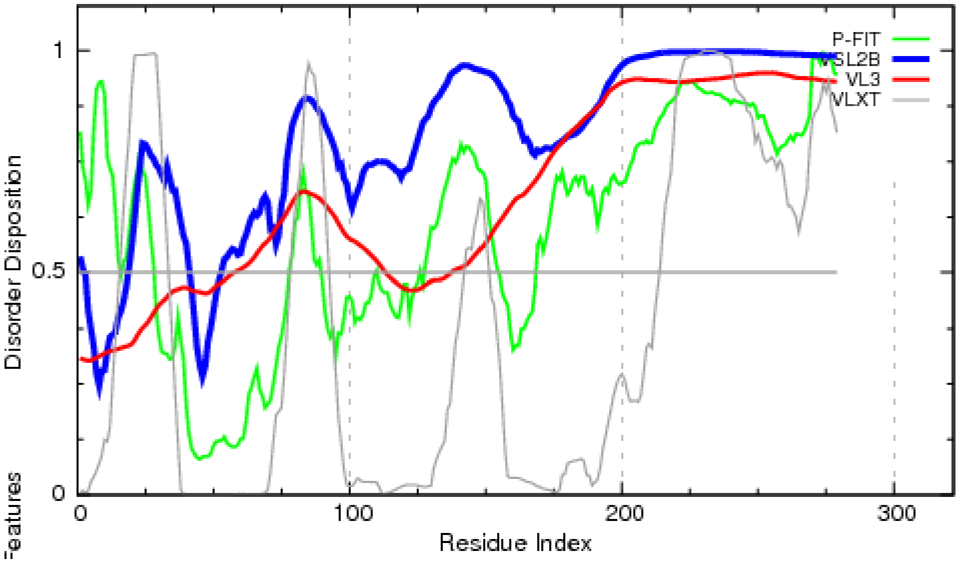

===============================PONDR VSL2 STATISTICS===============================

Predicted residues: 279
Number residues disordered: 252
Overall percent disordered: 90.32
Predicted disorder segment [1]-[2]
Predicted disorder segment [19]-[41]
Predicted disorder segment [53]-[279]

Number Disordered Regions: 3
Longest Disordered Region: 227
Average Prediction Score: 0.7983
Average Strength= 0.5224
Average Strength= 0.6797
Average Strength= 0.8639

ANCHOR:

Predicted Disordered Binding Regions			
	From	To	Length
1	119	125	7
2	158	180	23
3	250	257	8
4	267	279	13

ModPred and PROSITE:

ModPred:Proteolytic cleavage (Y75, K228, D229, D232, D242, D248, K267), Amidation (Q92), Carboxylation (E127, E133, E146, E147, E154, E160, E164, E165), Amidation (R145, Y176, L190), Acetylation (K195, K200, K220, K224), ADP-ribosylation (R226).

PROSITE: No identified domain recognition sites.

Structural modelling:

	Name	Title	Identity	Method	Oligo State	Ligands
☐	3etz.1.A	Adhesin A	20.63	X-ray, 2.0Å	monomer	None
☐	2dod.1.A	Transcription elongation regulator 1	11.67	NMR	monomer	None

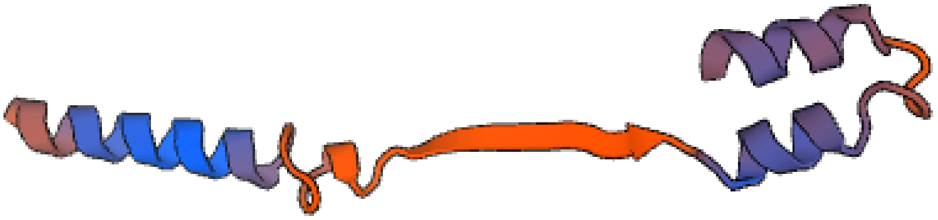

Model #1: Residues 104-169 of MVLG_00385T0 with 3etz.1.A (20.63 % sequence identity) as a template

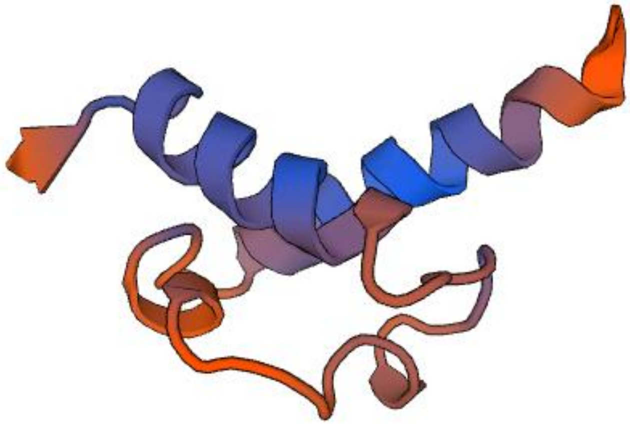

Model #2: Residues 107-168 of MVLG_00385T0 with 2dod.1.A (11.67 % sequence identity) as a template

>MVLG_06764T0 (80.27%)- 603 aa

MKLIRSARGAFLGAIGLSCLAVRLVRAQAGDEGNSYYADDTNSWIKFDGSWKEVVSDQFHQYTS
MLTSDKGATATFKFIGESFKILGISDQANFSVTVDGQDADVPSVSSDPDKTSALFSQTQLRPGTQHTV
ILEVISSGNLSFDAVVIGGGPASKAALCPVKGHQPCTSVNFQSIPKAHQSKPSTDRGGLFTTSLLHAGI
KLKLRKPDPDPSTPKDNSGDKSGNPLRHPKGAVGRKQKVDNTDKQDEKMGKDDVDQDGGKHQH
QNEGKGAPKDQPTNDSADKEYDPGMKIGKTSEHHSKEDPAGADTTKSEKAGLEGHELKPSKQNKP
DSPKTENPAPDDPKEPKETSAPHNQTDGAGGDSGLLEGLLGGAVGSSGSNEHESHHGGDHREGDSA
SQTPKKSSLIPAIVKLVGSDGGHKPSKSKASSKPTDAGTLGNSSDPHKPSSSDHSTTLVGLSRPLGDG
TGEEHHSNSKDSGIEQPEPKTLVDIKGGGDKHPDDPAADKPPPTPKKLVSIKGGPTDTGLQAGIDLPK
IVLPGSPATKLPDGQDVLGKKNVTGPSKDPGGKDHDQDHPIVPVVIKFSDSHDDPGSVATVVTVSQE
PVPKP

PONDR:

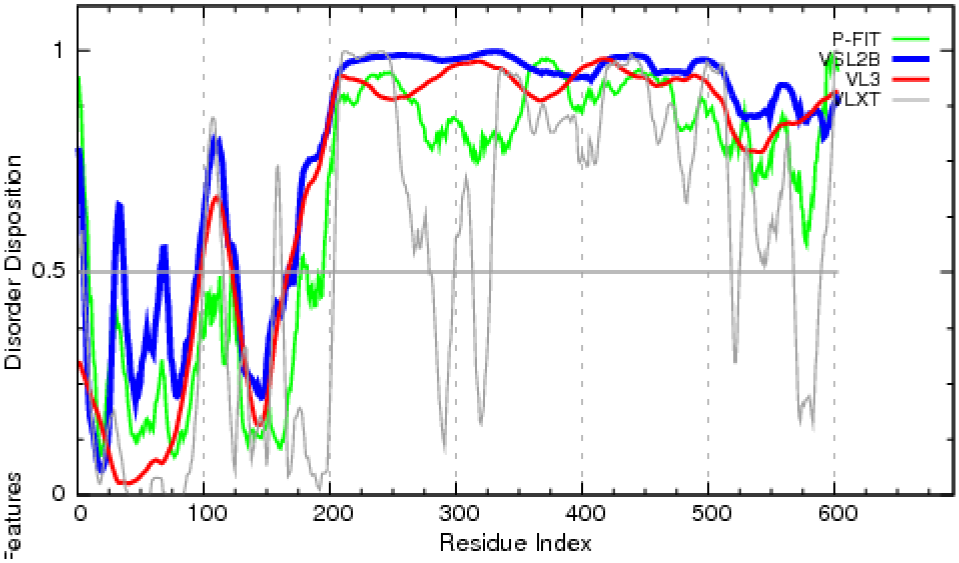

=============================PONDR VSL2 STATISTICS==============================

Predicted residues: 603	Number Disordered Regions: 6
Number residues disordered: 484	Longest Disordered Region: 432
Overall percent disordered: 80.27	Average Prediction Score: 0.7831
Predicted disorder segment [1]-[5]	Average Strength= 0.6747
Predicted disorder segment [30]-[37]	Average Strength= 0.5884
Predicted disorder segment [66]-[70]	Average Strength= 0.5322
Predicted disorder segment [96]-[126]	Average Strength= 0.6613
Predicted disorder segment [168]-[170]	Average Strength= 0.5116
Predicted disorder segment [172]-[603]	Average Strength= 0.9337

ANCHOR:

Predicted Disordered Binding Regions			
	From	To	Length
1	141	149	9
2	156	165	10
3	168	176	9
4	189	207	19
5	223	242	20
6	249	271	23
7	278	326	49
8	350	420	71
9	424	440	17
10	446	464	19
11	471	494	24
12	507	555	49
13	568	597	30
**Filtered Regions**			
	From	To	Length
1	134	138	5

ModPred and PROSITE:

ModPred: Sumoylation (K2), Amidation (I79, K410, T591), Phosphorylation (T214, Y285, S331, T398, T507, S537), Proteolytic cleavage (K231, D253, D393, K410, D547), ADP-ribosylation (R458), Ubiquitination (K494), Hydroxylation (P505, P506).

PROSITE: PROKAR_LIPOPROTEIN (Prokaryotic membrane lipoprotein lipid attachment site profile, 1-19, PROSITE entry PS51257). Signal (1-18), N-palmitoyl cysteine (19), S-diacylglycerol cysteine (19)

In prokaryotes, membrane lipoproteins are synthesized with a precursor signal peptide, which is cleaved by a specific lipoprotein signal peptidase (signal peptidase II). The peptidase recognizes a conserved sequence and cuts upstream of a cysteine residue to which a glyceride-fatty acid lipid is attached.

PROSITE: TONB_DEPENDENT_REC_1 (TonB-dependent receptor proteins signature 1, 1-101, PROSITE entry PS00430)

In *Escherichia coli*, the tonB protein interacts with outer membrane receptor proteins that carry out high-affinity binding and energy-dependent uptake of specific substrates into the periplasmic space. These substrates are either poorly permeable through the porin channels or are encountered at very low concentrations. In the absence of tonB these receptors bind their substrates but do not carry out active transport. The tonB protein also interacts with some colicins.

Structural modelling:

Name	Title	Identity	Method	Oligo State	Ligands
2wab.1.A	ENDOGLUCANASE E	24.04	X-ray, 1.9Å	monomer	None
5x7o.1.A	Glycoside hydrolase family 31 alpha-glucosidase	14.63	X-ray, 2.0Å	homo-dimer	2 x NI, 6 x CA, 9 x MG, 4 x MES
1fv3.1.A	TETANUS TOXIN HEAVY CHAIN	16.25	X-ray, 2.3Å	monomer	None
2vyu.1.A	CHOLINE BINDING PROTEIN F	24.59	X-ray, 2.5Å	monomer	None
3ron.1.A	Type-1Aa cytolytic delta-endotoxin	17.95	X-ray, 2.2Å	monomer	None

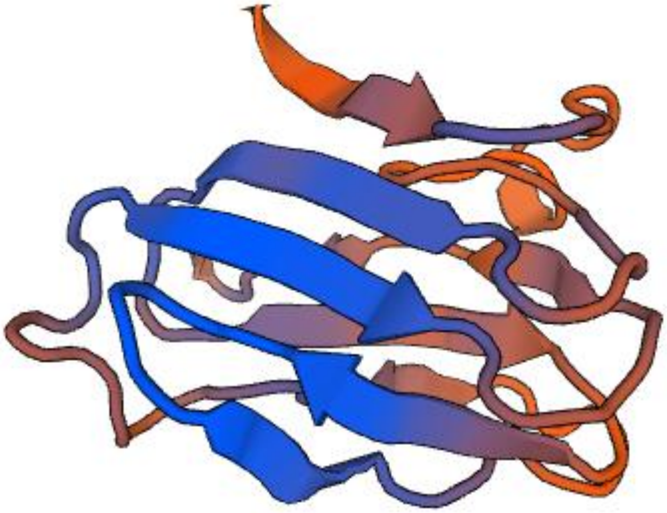

Model #1: Residues 42-152 of MVLG_06764T0 with 2wab.1.A (24.04 % sequence identity) as a template

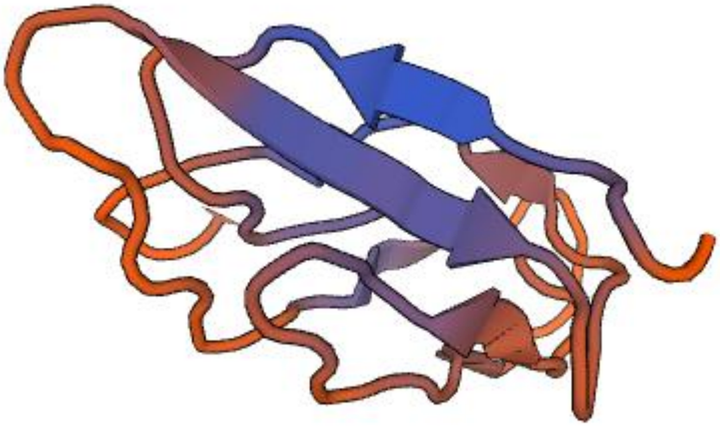

Model #2: Residues 69-154 of MVLG_06764T0 with 5x7o.1.A (19.05 % sequence identity) as a template

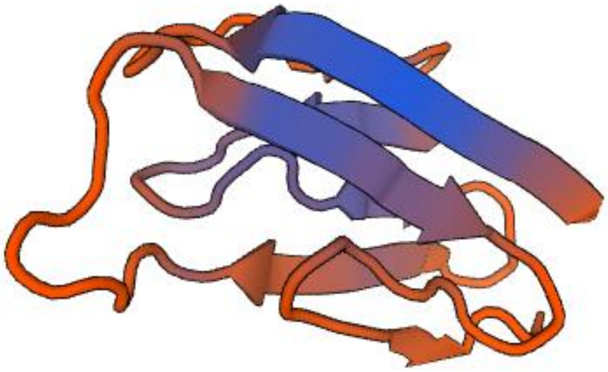

Model #3: Residues 71-151 of MVLG_06764T0 with 1fv3.1.A (16.25 % sequence identity) as a template

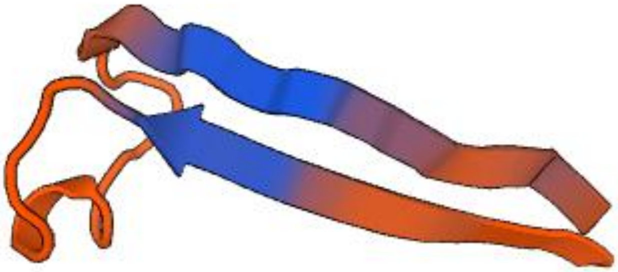

Model #4: Residues 65-103 of MVLG_06764T0 with 3ron.1.A (17.95 % sequence identity) as a template

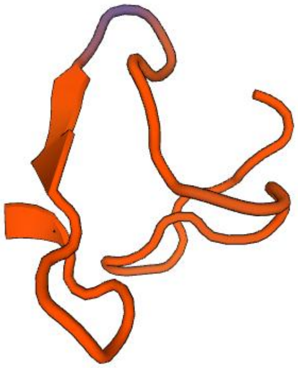

Model #5: Residues 29-61 of MVLG_06764T0 with 2vyu.1.A (24.59 % sequence identity) as a template

>MVLG_00398T0 (80.00%)- 205 aa

MPSMRSLTSFAFSALVAVSSSAPTVSSMPSLIERHDSPLSPPSLPPPPSPSVKSSLPTFSPPPPTYENQT
MCINYYFEHLPEYKDLYDCTVYDATKSFYDARYYQKKAIEKLEEEAQCAKDQADFADRVNQFAQA
ELAYAAEQKRFEWASKKFEHEKASLETSKKAFQALINQKVLEASQAMEIKHTCEKVFSEHTNEYIPK
HSI

PONDR:

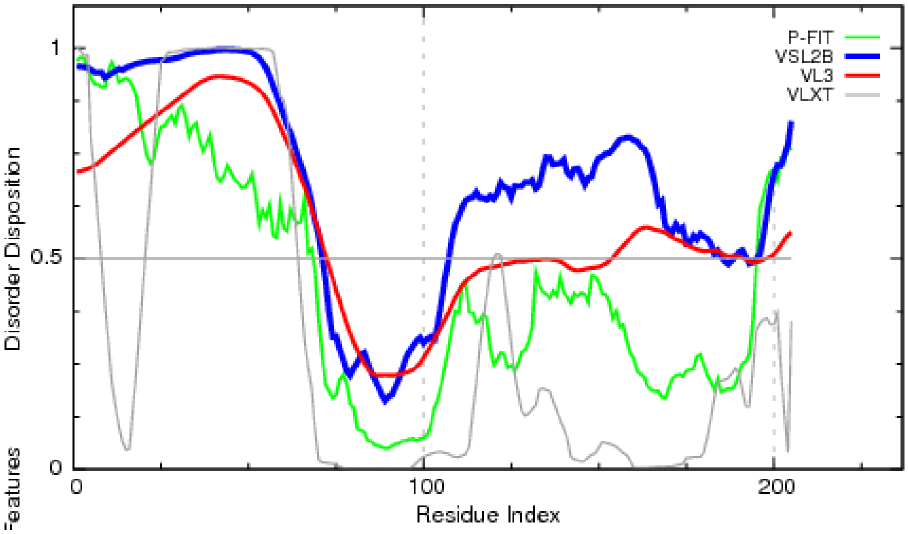

================================PONDR VSL2 STATISTICS================================

Predicted residues: 205
Number residues disordered: 164
Overall percent disordered: 80.00
Predicted disorder segment [1]-[71]
Predicted disorder segment [108]-[185]
Predicted disorder segment [189]-[192]
Predicted disorder segment [195]-[205]

Number Disordered Regions: 4
Longest Disordered Region: 78
Average Prediction Score: 0.6783
Average Strength= 0.9207
Average Strength= 0.6637
Average Strength= 0.5114
Average Strength= 0.6655

ANCHOR:

Predicted Disordered Binding Regions			
	From	To	Length
1	7	18	12
Filtered Regions			
	From	To	Length
1	71	79	9
2	86	89	4

ModPred and PROSITE:

ModPred: Phosphorylation (S37, S40, S49), Hydroxylation (P47), O-linked glycosylation (T58), Amidation (Y199).

PROSITE: No identified domain recognition sites.

Structural modelling:

	Name	Title	Identity	Method	Oligo State	Ligands
	2ap3.1.A	conserved hypothetical protein	20.00	X-ray, 1.6Å	monomer	None
	4bne.1.A	PROTEIN KINASE C AND CASEIN KINASE SUBSTRATE IN NEURONS PROTEIN 2	18.33	X-ray, 2.6Å	homo-dimer	None
	5cx2.1.B	Coronin	26.83	X-ray, 2.2Å	hetero-oligomer	None

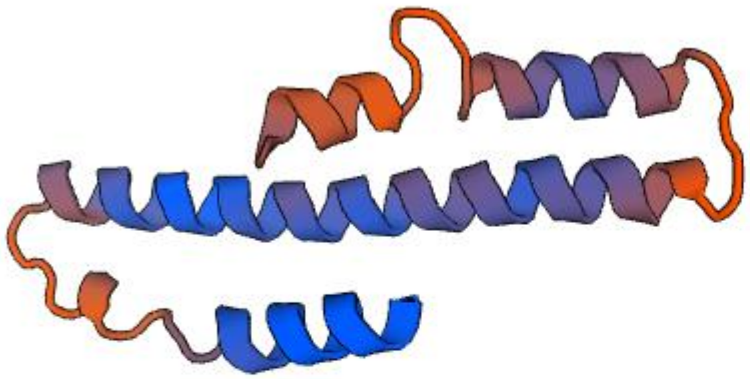

Model #1: Residues 106-199 of MVLG_00398T0 with 2ap3.1.A (19.78 % sequence identity) as a template

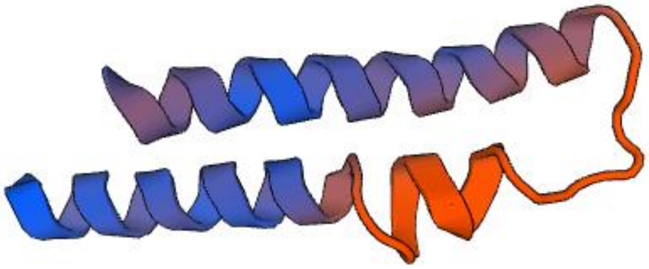

Model #2: Residues 125-187 of MVLG_00398T0 with 4bne.1.A (20.00 % sequence identity) as a template

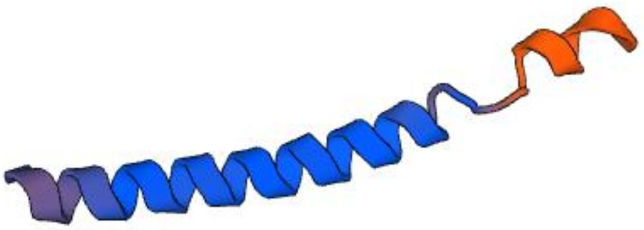

Model #3: Residues 142-182 of MVLG_00398T0 with 5cx2.1.B (26.83 % sequence identity) as a template

> MVLG_04106T0 (79.44%)- 107aa

MKYSLVFVALVVIATRIVSALAADATKQASTSEVDYPYFPEEHAATVSQGPPTRPITHPVASTLNES
LVNCKAEKCTTCKGEARGTCIEQCASWMAHQASQPEPEGC

PONDR:

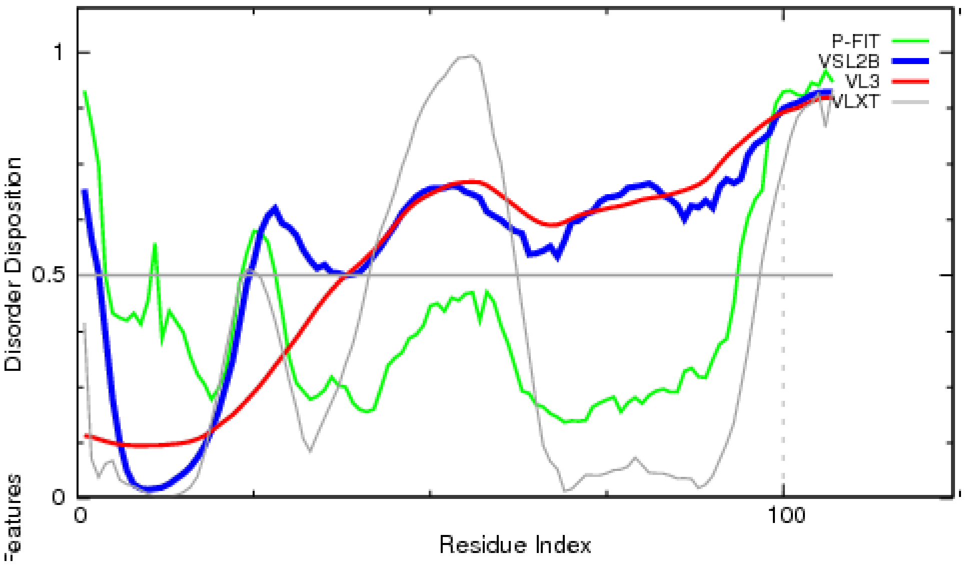

==============================PONDR VSL2 STATISTICS==============================

Predicted residues: 107
Number residues disordered: 85
Overall percent disordered: 79.44
Predicted disorder segment [1]-[3]
Predicted disorder segment [25]-[37]
Predicted disorder segment [39]-[107]

Number Disordered Regions: 3
Longest Disordered Region: 69
Average Prediction Score: 0.5594
Average Strength= 0.5938
Average Strength= 0.5667
Average Strength= 0.6827

ANCHOR:

Predicted Disordered Binding Regions			
	From	To	Length
None			

Filtered Regions			
	From	To	Length
1	3	14	12

ModPred and PROSITE:

ModPred: Post translational modification sites include proteolytic cleavage (D35), sulfation (Y36), Amidation (S48).

No identified domain recognition sites (PROSITE)

Structural modelling:

Name	Title	Identity	Method	Oligo State	Ligands
1csm.1.A	CHORISMATE MUTASE	22.22	X-ray, 2.2Å	homo-dimer	2 x TRP

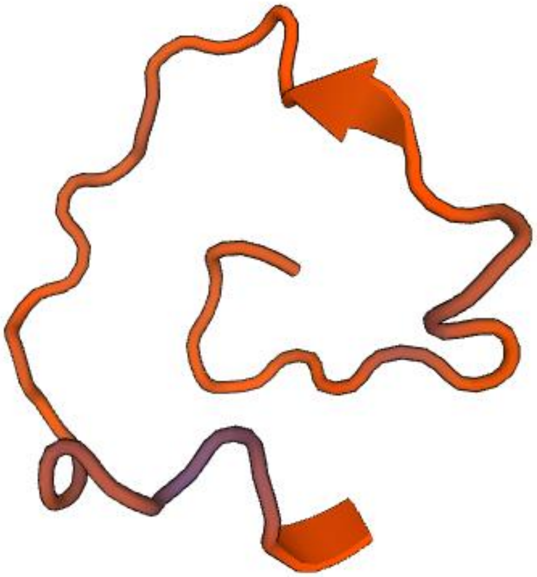

Model: Residues 30-65 of MVLG_04106T0 with 1csm.1.A (22.22% sequence identity) as a template

> MVLG_05720T0 (64.34%)- 129aa

MMRSLIKLLVLFTAVSVALANPWPPSVQDSCNWLKAWCTDCQTSFCGNITSHKQHKLCFKTHCES
HHPRDYPRPCKQMQMADKCMRSCQWKRSHNLTLSWNPFINHDKCRHCCDMQGGPTEKRMRRSG
Y

PONDR:

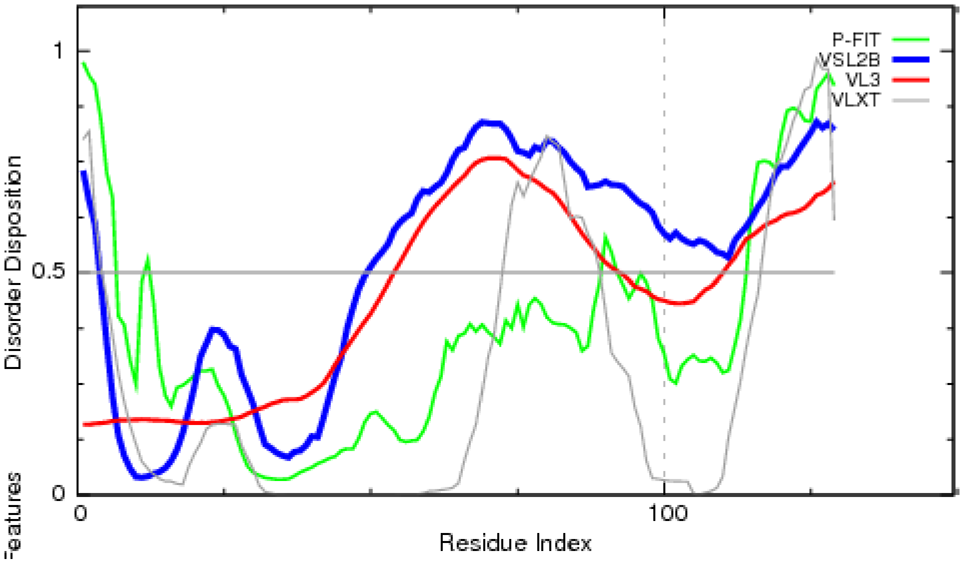

==============================PONDR VSL2 STATISTICS==============================

Predicted residues: 129
Number residues disordered: 83
Overall percent disordered: 64.34
Predicted disorder segment [1]-[3]
Predicted disorder segment [50]-[129]

Number Disordered Regions: 2
Longest Disordered Region: 80
Average Prediction Score: 0.5194
Average Strength= 0.6671
Average Strength= 0.6944

ANCHOR:

Predicted Disordered Binding Regions			
	From	To	Length
None			

Filtered Regions			
	From	To	Length
1	7	12	6

ModPred and PROSITE:

ModPred: SUMOylation (k7), Amidation (A14), Hydroxylation (P119), Proteolytic cleavage (R123, R126, S127)

PROSITE: No identified domain recognition sites.

Structural modelling:
No templates were found matching target sequence

> MVLG_06175T0 (61.02%)- 118aa

MWTSSIVQAALLFAVIVLYSSPVVAWAFCPFGKTAEHMAICSSLCRMRCYDPNSGTSNSTCRNAC
TGQYHVSRSLNAADQCMQQCDRFTKDKKKQGEGKLEHKRCLHKCTDWFFPLNL

PONDR:

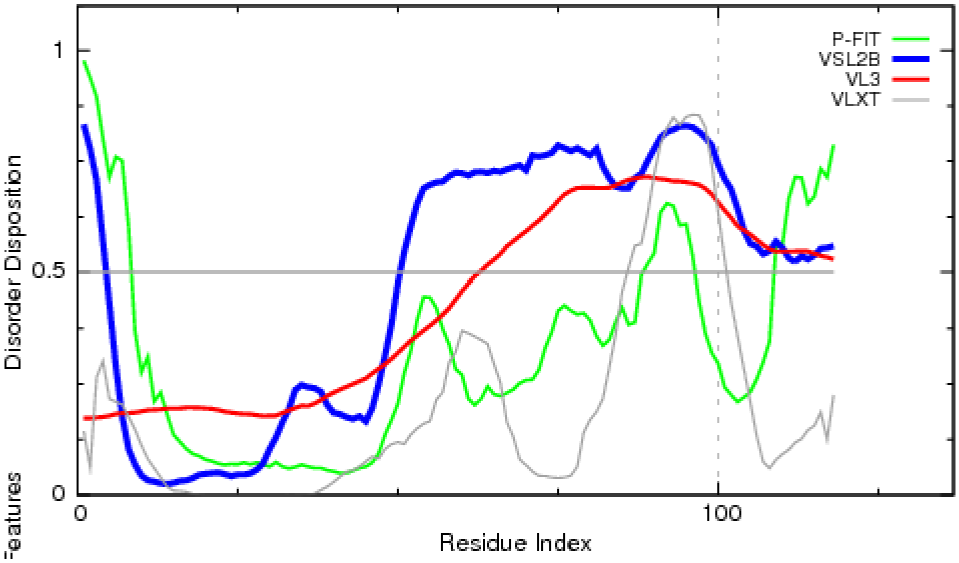

===============================PONDR VSL2 STATISTICS===============================

Predicted residues: 118
Number residues disordered: 72
Overall percent disordered: 61.02
Predicted disorder segment [1]-[4]
Predicted disorder segment [51]-[118]

Number Disordered Regions: 2
Longest Disordered Region: 68
Average Prediction Score: 0.4819
Average Strength= 0.7216
Average Strength= 0.6969

ANCHOR:

Predicted Disordered Binding Regions			
	From	To	Length
None			

Filtered Regions			
	From	To	Length
1	113	118	6

ModPred and PROSITE:

ModPred: Amidation (A10, S59, Y69), GPI anchor amidation (N53).

No identified domain recognition sites (PROSITE)

Structural modelling:

	Name	Title	Identity	Method	Oligo State	Ligands
☐	2v79.1.B	DNA REPLICATION PROTEIN DNAD	20.59	X-ray, 2.0Å	homo-tetramer	6 x NA, 22 x CL
☐	2ahx.1.A	Receptor tyrosine-protein kinase erbB-4	27.08	X-ray, 2.4Å	monomer	5 x NAG, 3 x NDG

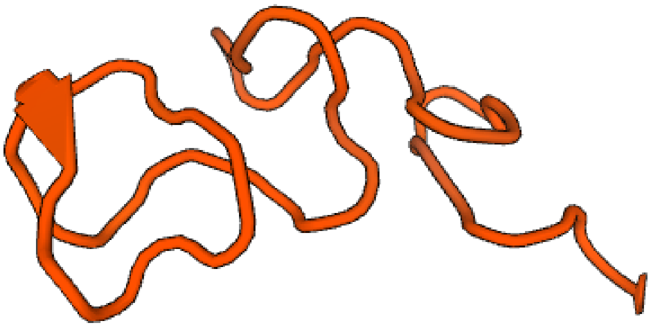

Model #1: Residues 40-87 of MVLG_06175T0 with 2ahx.1.A (27.08% sequence identity) as a template

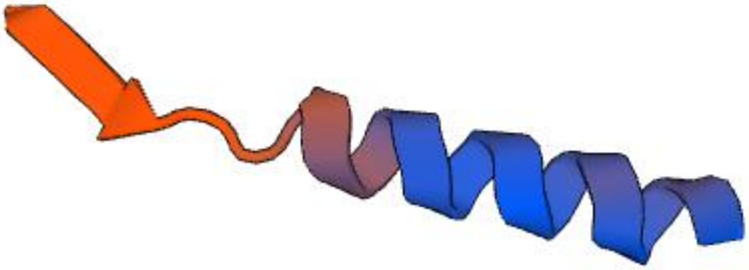

Model #2: Residues 66-88 of MVLG_06175T0 with 2v79.1.B (20.59% sequence identity) as a template

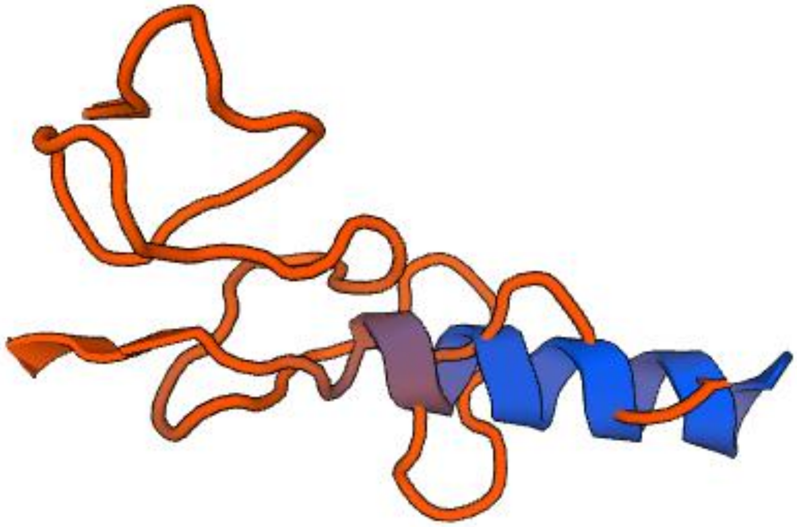

Overlap of models 1 and 2 for MVLG_06175T0

> MVLG_01591T0 (59.82%)- 448 aa

MRSLQLLSVLVTTCLPAAFAEIIDNTDAKKVITESGADISGNYTIQNAEGDYMHFQRDGTPGNSHV
SLSFVPQYSTVEVVSRTIYGASGRMHGRFTGVSLSGANKCAATQFNSNEGRDYDVVAYGCTFNRN
HTGAKMIFNVLPCGNTEDALSLAQKIRGVSKKEDFKFKKANPKSSPSRKSSGKSGAHRNTPHRPQSD
LSSSGQPGRHHVGGYRGKRHSGHGRRRGGHGGHGGHGHEGGNHHGGGHGHKGGNHHGGGHGH
KGGNHHGGGHPQHHHVRSLCTGNSLACQRRRHYLAKRDSRSQMLVSPQGPSPQGPSPQGPVSPSG
TPKQSASGASGGAGSAAGDHGPGPQSTAKKTQDGAVSQQASKDPNPASEADKSNSEIADHLRKNL
MSGKAQTVCIVGQDHLSDMQTAGLTGKETVGAGGVPGLMYDLFDASNDAFWLTMTRVN

PONDR:

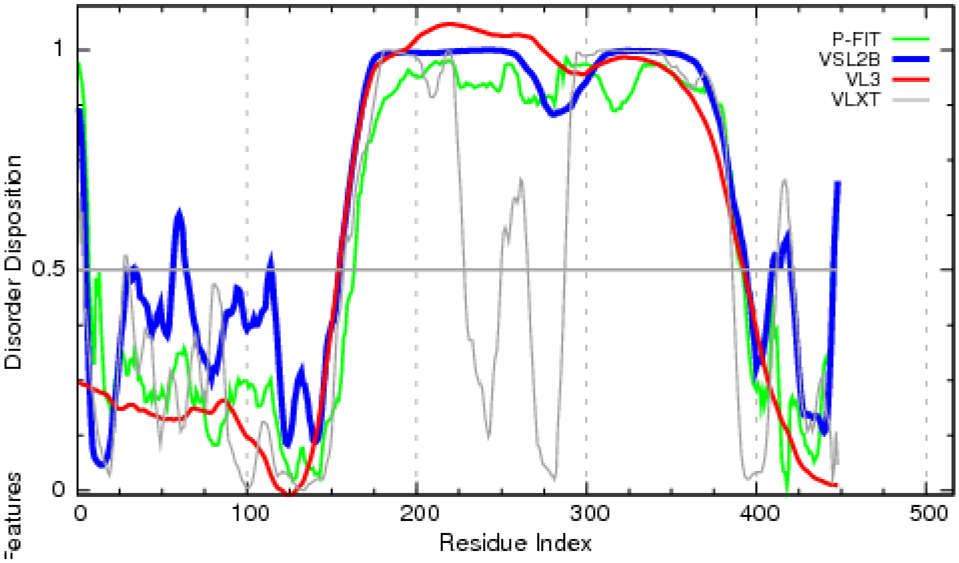

================================PONDR VSL2 STATISTICS================================

Predicted residues: 448	Number Disordered Regions: 7
Number residues disordered: 268	Longest Disordered Region: 240
Overall percent disordered: 59.82	Average Prediction Score: 0.6601
Predicted disorder segment [1]-[5]	Average Strength= 0.7239
Predicted disorder segment [33]-[33]	Average Strength= 0.5020
Predicted disorder segment [57]-[63]	Average Strength= 0.5857
Predicted disorder segment [114]-[114]	Average Strength= 0.5102
Predicted disorder segment [155]-[394]	Average Strength= 0.9325
Predicted disorder segment [410]-[420]	Average Strength= 0.5355
Predicted disorder segment [446]-[448]	Average Strength= 0.6314

ANCHOR:

Predicted Disordered Binding Regions			
	From	To	Length
1	136	144	9
2	151	174	24
3	181	311	131
4	318	349	32
5	352	367	16
6	378	407	30

	From	To	Length
7	426	441	16
**Filtered Regions**			
	**From**	**To**	**Length**
1	122	126	5

ModPred and PROSITE:

ModPred: Amidation (M53, G59, N104, R290, K354, M429, T445), Proteolytic cleavage (Y119, D120, K180, K184, R189, Q302, M303), Acetylation (K162, K216), Sumoylation (K167), Phosphorylation (S176, S322), Methylation (R225).

PROSITE: No identified domain recognition sites.

Structural modelling:

Name	Title	Identity	Method	Oligo State	Ligands
4y3k.1.A	Serpin A12	17.14	X-ray, 2.2Å	monomer	None
4k07.1.A	Amyloidogenic immunoglobulin light chain protein AL-103	5.71	X-ray, 2.8Å	homo-dimer	None

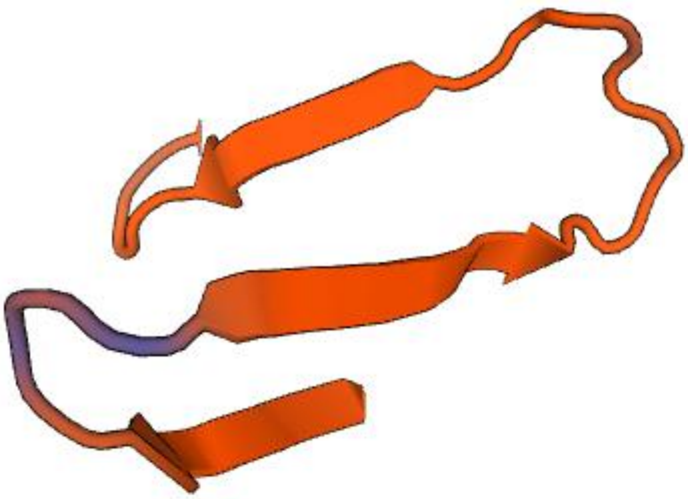

Model #1: Residues 29-63 of MVLG_01591T0 with 4k07.1.A (5.71% sequence identity) as a template

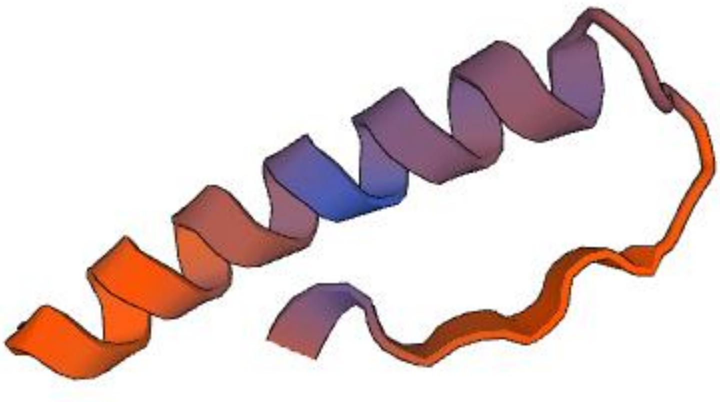

Model #2: Residues 274-308 of MVLG_01591T0 with 4y3k.1.A (17.14% sequence identity) as a template

> MVLG_04168T0 (58.93%)- 319 aa

MHLVTVLPFALVAFLGSTGVQALIKTANATLEALYLVEYDTKAYGTPETGAVKHFGTNFHQACV
AAAIQKIPIMHCRVADIPFNPLIIPQDGTWDNHGPVTDLMDLAMNNTLFLGNARIVSGPDGKPYAPA
YPPPTKPTPETRDADAASHVPDSTGLVPGTTLPGPSTIPGPGTTPPGPGTTLPGPSTIPGPGTTPPGPGT
TLPGPSTIPGPGTTPGPGTTLPGPSTIPGPSTIPGPGTTPPGPGPTLPGPGPTLVPVSRPTTPLTGGHGRK
GRKHRNGRKGGFKRVQVTVDDTVDDFLMNGQVQPPDSVDTLLGGIAF

PONDR:

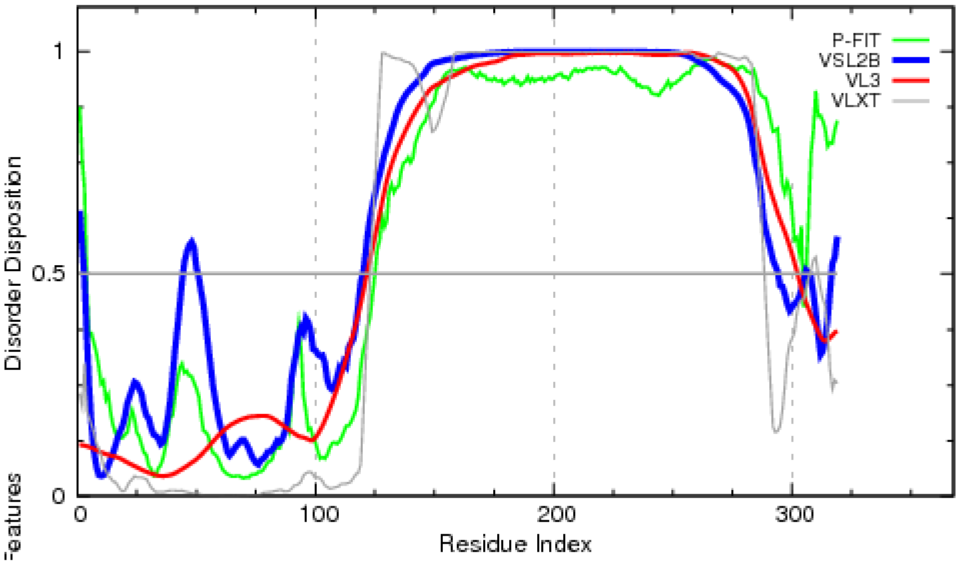

================================PONDR VSL2 STATISTICS================================

Predicted residues: 319
Number residues disordered: 188
Overall percent disordered: 58.93
Predicted disorder segment [1]-[2]
Predicted disorder segment [45]-[50]
Predicted disorder segment [120]-[294]
Predicted disorder segment [306]-[307]
Predicted disorder segment [317]-[319]

Number Disordered Regions: 5
Longest Disordered Region: 175
Average Prediction Score: 0.6365
Average Strength= 0.6015
Average Strength= 0.5467
Average Strength= 0.9317
Average Strength= 0.5051
Average Strength= 0.5479

ANCHOR:

Predicted Disordered Binding Regions			
	From	To	Length
1	105	121	17
2	144	171	28
3	180	193	14
4	198	238	41
5	246	273	28
6	277	306	30
7	309	319	11

ModPred and PROSITE:

ModPred: Proteolytic cleavage (R118, S121, R143, G156, R277, R280, K281, K285, R286, D311), Hydroxylation (P133, P134, P135, P237, P244), Amidation (P134, P204, P257), Phosphorylation (T139, T175, T195, T240, T263), O-linked glycosylation (S167, S187, S207, T215), ADP-ribosylation (R260).

PROSITE: No identified domain recognition sites.

Structural modelling:

Name	Title	Identity	Method	Oligo State	Ligands
1z5z.2.A	Helicase of the snf2/rad54 family	11.11	X-ray, 2.0Å	monomer	None

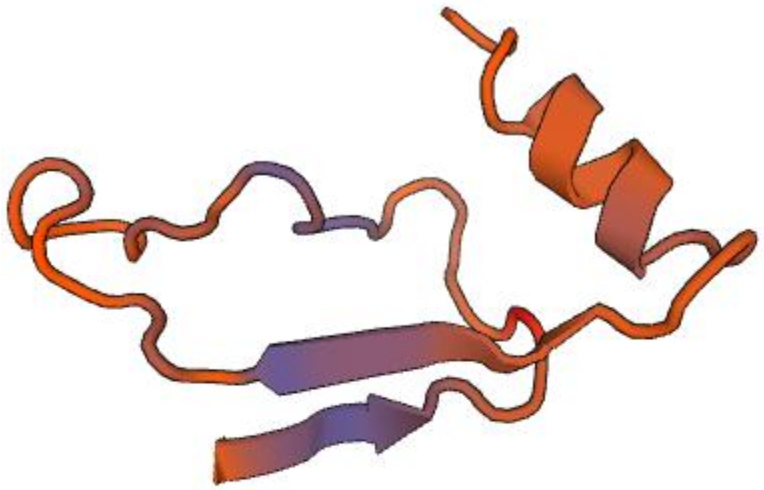

Model: Residues 253-307 of MVLG_04168T0 with 1z5z.2.A (11.11% sequence identity) as a template

> MVLG_02288T0 (55.25%)- 324 aa

MRFLTSQITLCLLVLVTSTLAFHVANLFPDLSVDAHRTVPSSNRRHHRCLAHSVRHNHHKKCRHS
RKTGLKHFDQEKSAHTHGHLAHRNRRKKPAIKRLGKKRPVSHVDPNHKRPEHDQSNPPTILTGPTL
QQPEPSGVHVSKTPAGKTAPERQIPDGKDQVSEIQALALEEINAFRALHNAPPLQTSPELVQNAVVW
TSKCHYGHTRGAFTGEYGEIIARTSGSWGNNMSKAIELWTVDEENDFNPRKPQTTHFTQAVWKSSR
LLGCASSDKCNDPADNSTTVTGDDIPPDEHNSVLYICRFLPAGNLNDKDVDIIMLKGFAD

PONDR:

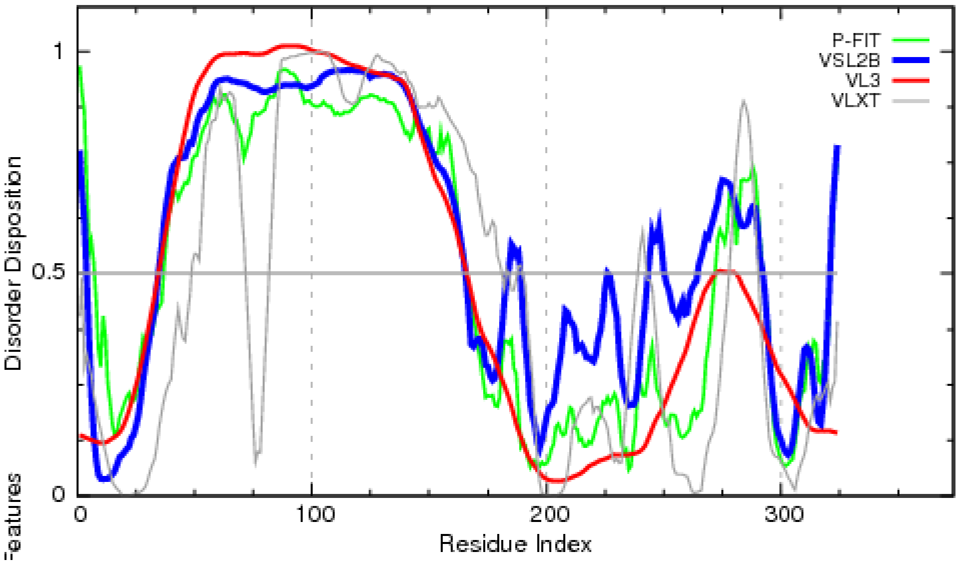

================================PONDR VSL2 STATISTICS=================================

Predicted residues: 324
Number residues disordered: 179
Overall percent disordered: 55.25
Predicted disorder segment [1]-[3]
Predicted disorder segment [35]-[165]
Predicted disorder segment [184]-[188]
Predicted disorder segment [226]-[226]
Predicted disorder segment [244]-[250]
Predicted disorder segment [265]-[292]
Predicted disorder segment [321]-[324]

Number Disordered Regions: 7
Longest Disordered Region: 131
Average Prediction Score: 0.5690
Average Strength= 0.6941
Average Strength= 0.8695
Average Strength= 0.5446
Average Strength= 0.5008
Average Strength= 0.5636
Average Strength= 0.6244
Average Strength= 0.6675

ANCHOR:

Predicted Disordered Binding Regions			
	From	To	Length
1	46	112	67
2	122	147	26
3	161	180	20
4	192	205	14
5	232	237	6
6	259	268	10
7	297	305	9

Filtered Regions			
	From	To	Length
1	10	27	18
2	218	218	1
3	318	319	2

ModPred and PROSITE:

ModPred: Amidation (A21), Proteolytic cleavage (D34, H36, R37, H52, R88, R98), Acetylation (K71, K97).

PROSITE: No identified domain recognition sites.

Structural modelling:

Name	Title	Identity	Method	Oligo State	Ligands
5jys.1.A	Protein PRY1	25.81	X-ray, 1.9Å	monomer	1 x MG
2giz.1.A	Natrin-1	26.62	X-ray, 1.7Å	monomer	None
4kt0.1.I	Photosystem I reaction center subunit XII	30.77	X-ray, 2.8Å	hetero-oligomer	4 x LHG, 3 x SF4, 90 x CLA, 2 x PQN, 2 x LMU, 2 x CL0, 17 x BCR, 1 x LMG

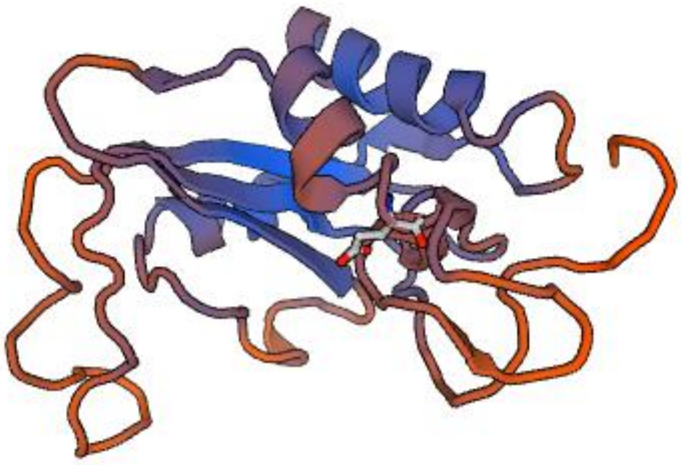

Model #1: Residues 163-321 of MVLG_02288T0 with 2giz.1.A (26.62% sequence identity) as a template

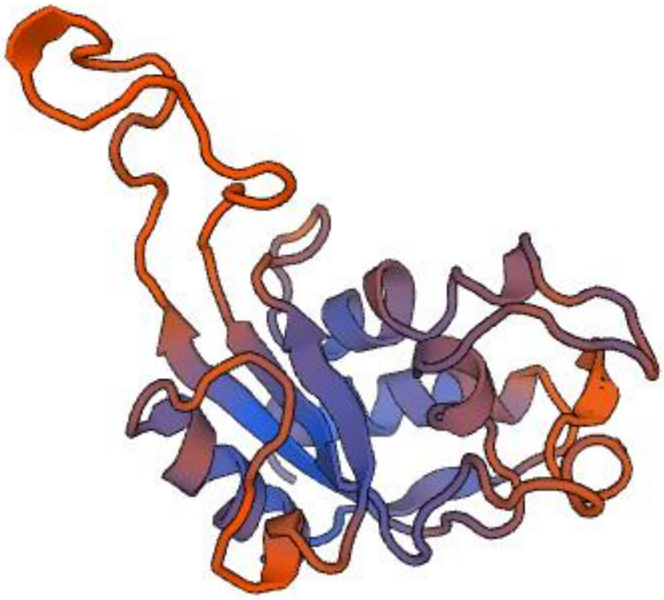

Model #2: Residues 162-320 of MVLG_02288T0 with 5jys.1.A (25.81% sequence identity) as a template

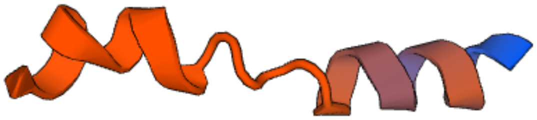

Model #3: Residues 3-26 of MVLG_02288T0 with 4kt0.1.I (30.77% sequence identity) as a template

> MVLG_00566T0 (55.00%)- 80 aa

MRTCSIVFALGTLLTLSLTQVVVAAPKAADSTDFTKGMSCNSCVKTCNQKHLATGSADMEAGTS
LVDCMDSCISVYNCES

PONDR:

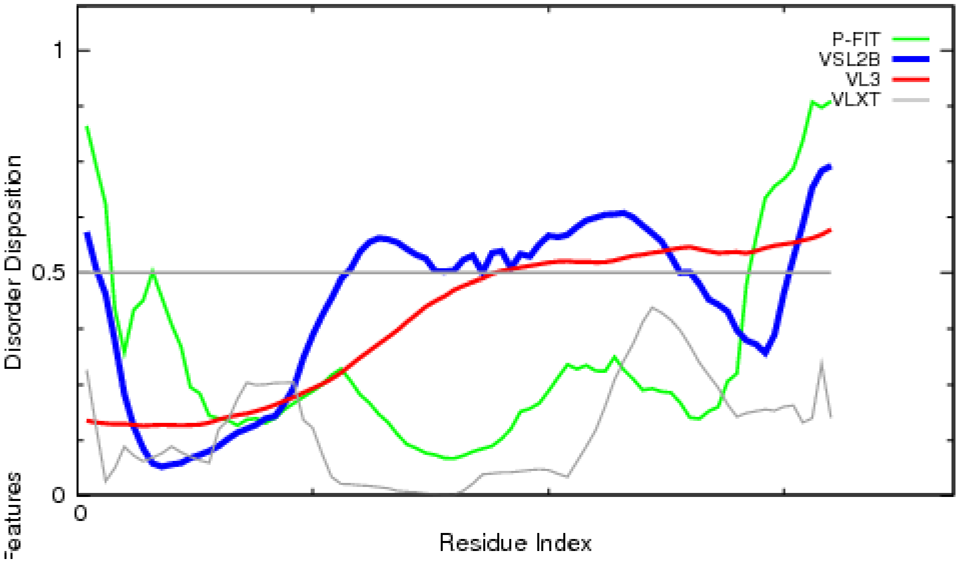

===============================PONDR VSL2 STATISTICS================================

Predicted residues: 80	Number Disordered Regions: 3
Number residues disordered: 44	Longest Disordered Region: 37
Overall percent disordered: 55.00	Average Prediction Score: 0.4306
Predicted disorder segment [1]-[2]	Average Strength= 0.5504
Predicted disorder segment [29]-[65]	Average Strength= 0.5601
Predicted disorder segment [76]-[80]	Average Strength= 0.6607

ANCHOR:

Predicted Disordered Binding Regions			
	From	To	Length
None			

ModPred and PROSITE:

ModPred: Proteolytic cleavage (D33), Carboxylation (E79).

PROSITE: No identified domain recognition sites.

Structural modelling:

Name	Title	Identity	Method	Oligo State	Ligands
5d6s.1.A	Epoxyqueuosine reductase	15.15	X-ray, 2.6Å	monomer	2 x SF4, 1 x B12

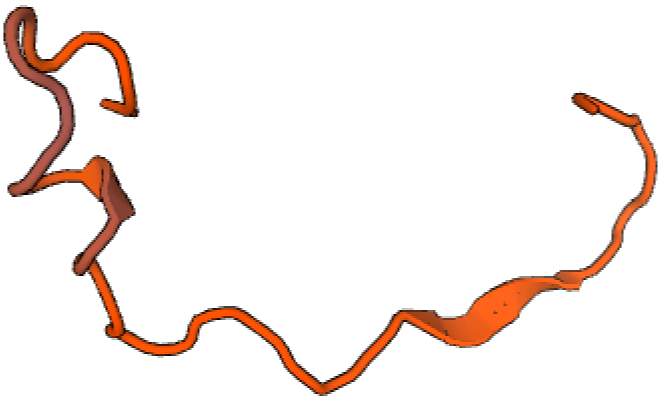

Model: Residues 21-53 of MVLG_00566T0 with 5d6s.1.A (15.15% sequence identity) as a template

Highly disordered proteins (30-50% disordered residues by PONDR® VSL2 analysis)
> MVLG_05122T0 (43.93%)- 173 aa
MLFKVSAALVLAGLSLGASALPSMSTESRAQPSPSSNKSPYGRTGYIDSPADRKTTTYKVGDKIHF
VYTSAPATYFVDVSLMLANGSQSFQLANRLTGSSMISNDANARAYFRMPENLKTIATELLAASQDE
HSGAMKNNNCILAYLIAKETQNGQYGLVGNLETKQAIAISM

PONDR:

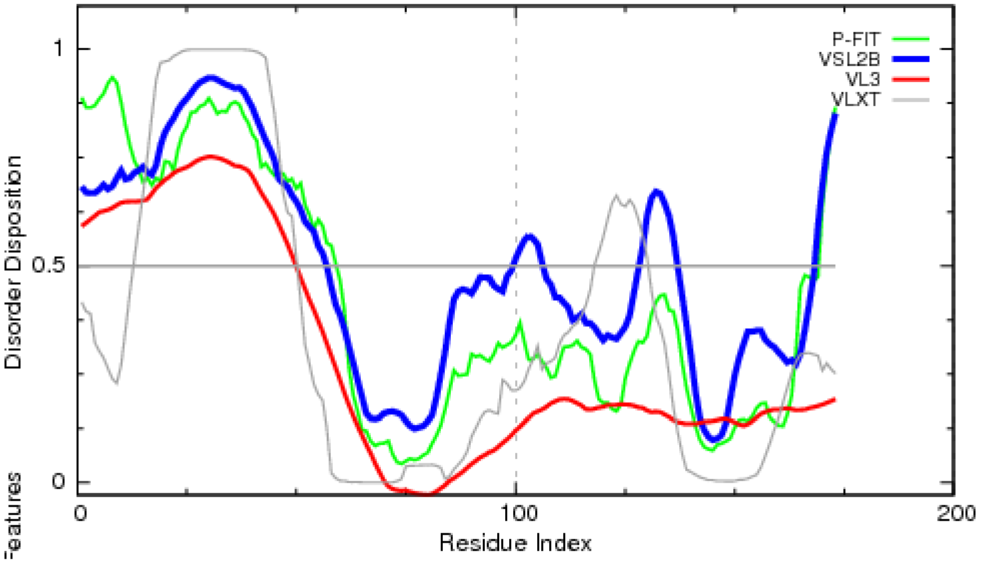

================================PONDR VSL2 STATISTICS================================

Predicted residues: 173
Number residues disordered: 76
Overall percent disordered: 43.93
Predicted disorder segment [1]-[56]
Predicted disorder segment [100]-[106]
Predicted disorder segment [129]-[136]
Predicted disorder segment [169]-[173]

Number Disordered Regions: 4
Longest Disordered Region: 56
Average Prediction Score: 0.4965
Average Strength= 0.7669
Average Strength= 0.5439
Average Strength= 0.6229
Average Strength= 0.7406

ANCHOR:

Predicted Disordered Binding Regions			
	From	To	Length
1	142	147	6
**Filtered Regions**			
	From	To	Length
1	1	16	16
2	75	81	7

ModPred and PROSITE:

ModPred: Proteolytic cleavage (E27), Phosphorylation (S49), GPI anchor amidation (N104), Amidation (Y157).

PROSITE: No identified domain recognition sites.

Structural modelling:

Name	Title	Identity	Method	Oligo State	Ligands
2o2o.1.A	SH3-domain kinase-binding protein 1	15.91	NMR	monomer	None

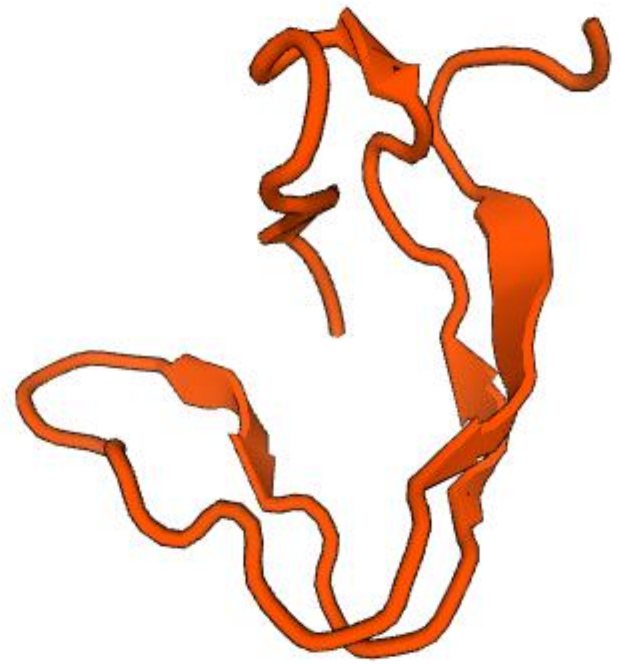

Model: Residues 35-78 of MVLG_05122T0 with 2o2o.1A (15.91 % sequence identity) as a template

> MVLG_03398T0 (43.07%)- 202 aa

MLTLPLLVASLGFCHSVVGSRHKNQDAHQVQGSTGKEPLIDADKMYLIRVNPQITGLQSACTFLS
DRCEKYVKRGPNVKQLDVSCSSAGQAVTSTSPYLWASCFETGTNEKDGRARDVSFNAFAGSDHAI
VFLRGDEVFREVEIDEDLLKTESKKWKPTVNHQPQNRSPRQAGHRQINHETTGPKTHSGHNGDRPK
RHKQET

PONDR:

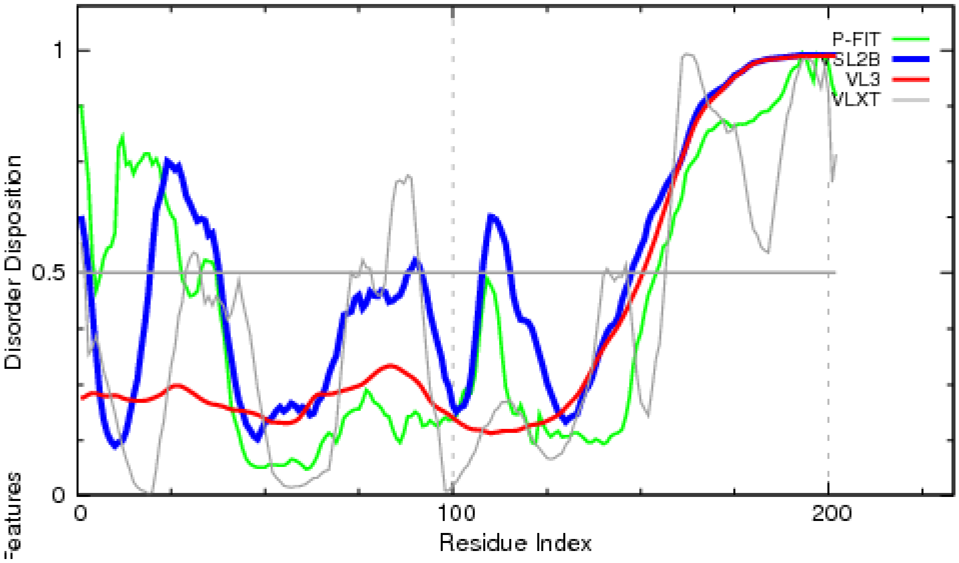

==============================PONDR VSL2 STATISTICS===============================

Predicted residues: 202
Number residues disordered: 87
Overall percent disordered: 43.07
Predicted disorder segment [1]-[3]
Predicted disorder segment [20]-[37]
Predicted disorder segment [89]-[91]
Predicted disorder segment [108]-[115]
Predicted disorder segment [148]-[202]

Number Disordered Regions: 5
Longest Disordered Region: 55
Average Prediction Score: 0.5044
Average Strength= 0.5705
Average Strength= 0.6548
Average Strength= 0.5176
Average Strength= 0.5787
Average Strength= 0.8722

ANCHOR:

Predicted Disordered Binding Regions			
	From	To	Length
1	98	103	6
2	120	146	27
3	172	179	8

Filtered Regions			
	From	To	Length
1	6	13	8
2	148	149	2

ModPred and PROSITE:

ModPred: Proteolytic cleavage (R116, D117, N121).

PROSITE: No identified domain recognition sites.

Structural modelling:

	Name	Title	Identity	Method	Oligo State	Ligands
☐	2myn.1.A	Glutaredoxin arsenate reductase	17.91	NMR	monomer	None
☐	1ybx.1.A	Conserved hypothetical protein	19.44	X-ray, 1.8Å	homo-dimer	None

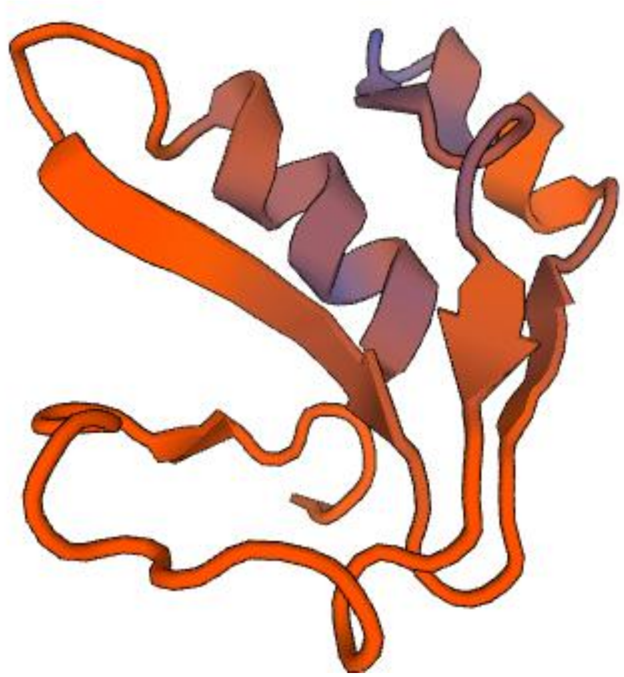

Model #1: Residues 35-78 of MVLG_03398T0 with 2myn.1.A (17.91 % sequence identity) as a template

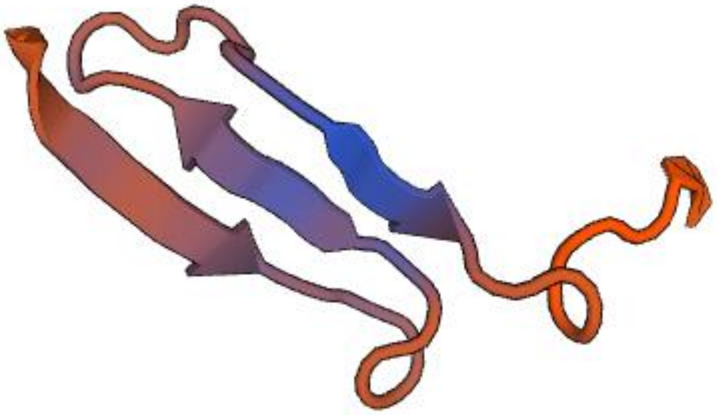

Model #2: Residues 117-152 of MVLG_03398T0 with 1ybx.1.A (19.44 % sequence identity) as a template

> MVLG_05716T0 (41.84%)- 141 aa

MMYSSLFIFAFTVVGAIVNAKMAKVATNSQTTSLGPVAGVEKFHQPYWKNGTAAPAACVAVSQA
CFECLSKCYQHHNQWGFGNKTDCYYGQCNNTRETRYKESCAIENNAKTCSDGLPKAQQTGGPMLE
NCCKQANGTSLY

PONDR:

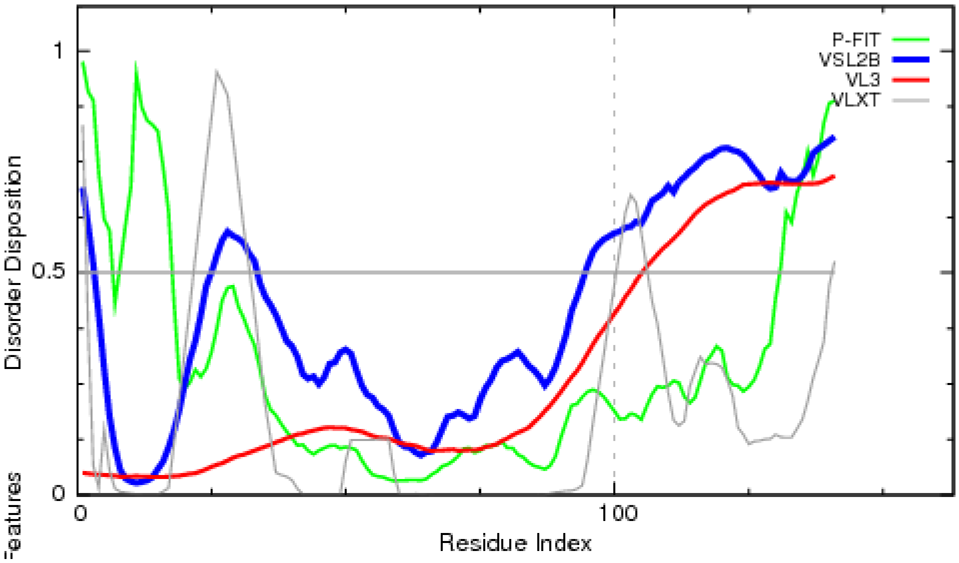

==============================PONDR VSL2 STATISTICS==============================

Predicted residues: 141	Number Disordered Regions: 3
Number residues disordered: 59	Longest Disordered Region: 47
Overall percent disordered: 41.84	Average Prediction Score: 0.4194
Predicted disorder segment [1]-[3]	Average Strength= 0.6057
Predicted disorder segment [25]-[33]	Average Strength= 0.5559
Predicted disorder segment [95]-[141]	Average Strength= 0.6970

ANCHOR:

Predicted Disordered Binding Regions			
	From	To	Length
None			

ModPred and PROSITE:

ModPred: Disulphide linkage (C87, C92), Carboxylation (E102).

PROSITE: No identified domain recognition sites.

Structural modelling:

	Name	Title	Identity	Method	Oligo State	Ligands
☐	1mm9.1.A	Streptavidin	15.56	X-ray, 1.7Å	homo-tetramer	8 x MRD

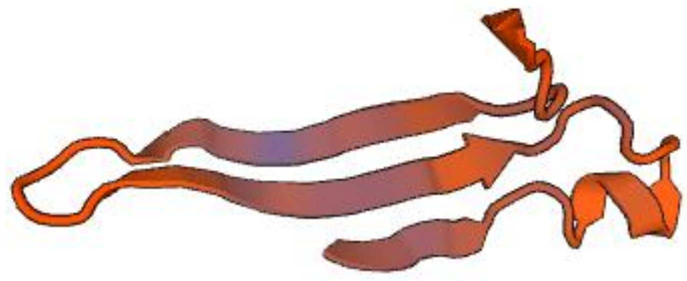

Model: Residues 79-125 of MVLG_05716T0 with 1mm9.1.A (15.56 % sequence identity) as a template

> MVLG_04806T0 (40.65%)—647 aa

MPDLSWFHSALLLSCLGTLALSQPINLTTYHLYDSSHAAQGLVHPDALRGELVQTPSALLNALGL
RDAELFVWASPKGTRGTSQVAIMQMETHEKQKVLNMQRFSQLLAAVKCKPSQVTIQFVTKAAFEA
ASQLWSAVNSDRIWHLQLFTSWKGCYTDGGNLKPFHLTEVSFDSEKLAATLTGNETDWKTAAHTFI
MSSGEHFDETPPTADGSSRPLLTRSSIFTKAGKEFAKEFKSVAKSIDKVENKFLKKIRKELHATLANK
HKVLRIAFDKSYTGTKTFSTPGKAASLNGSVTCTGCGPTGSLVLHTVIKVTLGEEPTVKLTMKPQNL
GVSLGLAMSAKSDFPESFGIETPLLEQTIPAAGFKIPEIASVGLVASLGYGISVSNFKGNLNVAKNVSV
SIPDGAKLNLLVNPSNDEQSKLGGSWAPVAQSTPMHISGFGSGEFAVGLGISLSLKMEVLSFEITPAK
ITLSGPSIGFEFGVSSKECGPYTSVGSSHDPTYYITPKLGFSLSVGSDLNVDSGTWGLGIPIPDSGGPNV
NSSHGTGGSGGGGGGTNPPYPSSTHTNPDQKDSSQPAVKTRSVNKRHNHDPIGASKISISASKTLYE
QSFRLHSPICFMSGLHQRRPLSNHGRKDEPDGGTRRLSNSSE

PONDR:

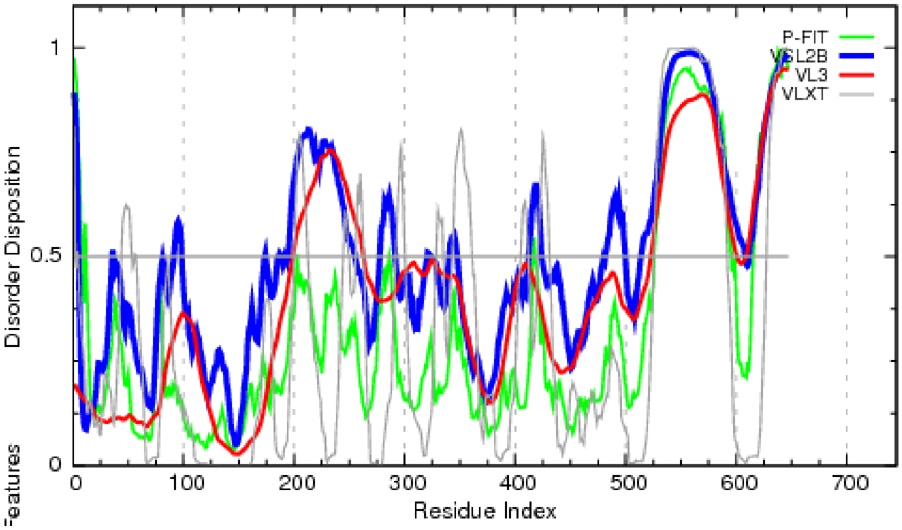

===============================PONDR VSL2 STATISTICS===============================

Predicted residues: 647	Number Disordered Regions: 14
Number residues disordered: 263	Longest Disordered Region: 94
Overall percent disordered: 40.65	Average Prediction Score: 0.4942
Predicted disorder segment [1]-[5]	Average Strength= 0.7473
Predicted disorder segment [36]-[36]	Average Strength= 0.5165
Predicted disorder segment [92]-[98]	Average Strength= 0.5592
Predicted disorder segment [174]-[174]	Average Strength= 0.5079
Predicted disorder segment [186]-[188]	Average Strength= 0.5043
Predicted disorder segment [192]-[192]	Average Strength= 0.5019
Predicted disorder segment [195]-[255]	Average Strength= 0.6964
Predicted disorder segment [278]-[291]	Average Strength= 0.5958
Predicted disorder segment [321]-[322]	Average Strength= 0.5085
Predicted disorder segment [341]-[347]	Average Strength= 0.5378
Predicted disorder segment [412]-[424]	Average Strength= 0.5981
Predicted disorder segment [482]-[499]	Average Strength= 0.5869
Predicted disorder segment [514]-[607]	Average Strength= 0.8037
Predicted disorder segment [612]-[647]	Average Strength= 0.8180

ANCHOR:

Predicted Disordered Binding Regions			
	From	To	Length
1	503	531	29
2	573	584	12
3	587	626	40
Filtered Regions			
	From	To	Length
1	268	270	3
2	642	642	1
3	644	647	4

ModPred and PROSITE:

ModPred: Proteolytic cleavage (R49, D570, R579), Amidation (T82, T119, L217, I351, S461, S521, K583), ADP-ribosylation (R215, R579), Acetylation (K233, K244, K248), O-linked glycosylation (S533, S547), Phosphorylation (S643, S645, S646).

PROSITE: No identified domain recognition sites.

Structural modelling:

No templates were found matching target sequence.

> MVLG_03092T0 (40.02%)- 812 aa

MKFSNSIIACALLASGWADLARTHGVDAGGGSIGVSLPPTTPSDPSCTGSNEWFSPYRSTFEAHER
GENLDQLKQPGSIDDSEYLLNPNFSINNCQTTRYYYLDIHETRAAPDGFEREMFLFNGRINGPLIEAN
QGDTIVVYVHNYLDIGTTVHWHGLAQNGSGWADGPLGVTQCPIPPGTTFIYKYTLSRFDQCGTYW
YHAHRLAHYSDGLVAPLVIHCPNDPLKRGDLYDIDQVVVVRDHYHPLSTRIISALLVNGSFQGSSAT
PSPNAGLINGRGRYNCSFAPEGSVCTDDAPLTEFEFPKGSRVRLRLINPSAHAQFLVSVDEHPLNVVE
ADDTPVWQTTVHRIPINVGQRYSAILNTADNNEGDSFWMRADINTACFGANFTDLNPEVKAIIRIGP
ASSSPSSSVSSSASSENGSNPPSQGASNGSSDSSGDHSSSGNPSDLQQSSNNNGTSGDAPDGSDQSDN
SGDGNASGDGRGDWGNQEDSDGSGDSGDQQGSQRGSRWKRAAGLRKRNGNNNDNNDNNDNND
NNDSNDNNDNNDNNDNNNSGSNTNTNQNLPTSTDWSDAVNGSCHDLAESTLVPRVPFNPPGASIS
HEFRATILTTPSGAFGFAANNVSFESFVDDPFLFRVNRGDDIPLGLSASIVLDDKSLAHDIVINNANPI
DHPFHLHGVQMHLIARGAGSVSADNISSVALNLNNPIRRDTISVTGNTFAIVRVVADNAGVWAIHC
HILPHQVTGLMGVVVIRPDLIRKMEIPQHARDLCTLGSSLSSAQGQDPQPNIEPGRRIRRSINPLLPSK
DFIRKRVLLNQD

PONDR:

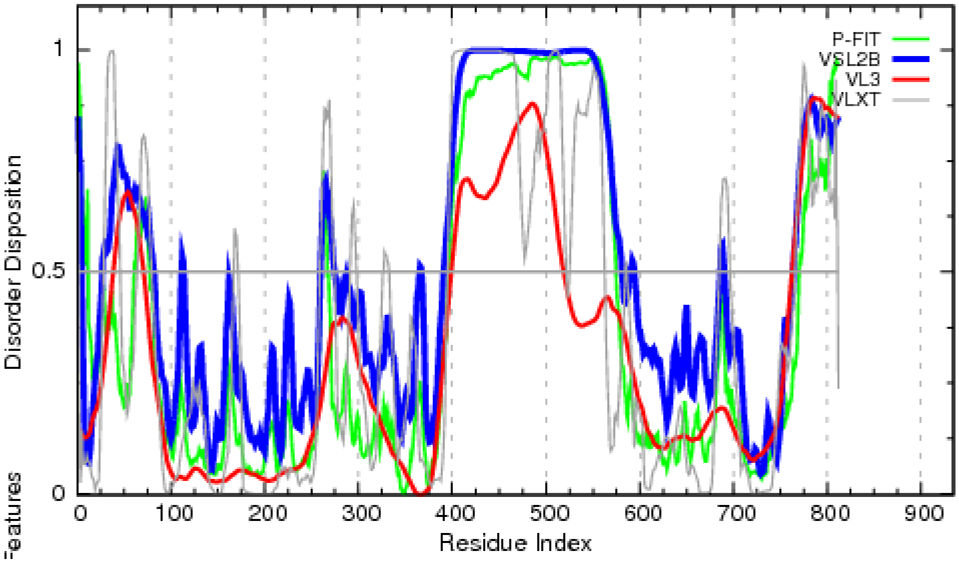

==============================PONDR VSL2 STATISTICS===============================

Predicted residues: 812	Number Disordered Regions: 9
Number residues disordered: 325	Longest Disordered Region: 189
Overall percent disordered: 40.02	Average Prediction Score: 0.4951
Predicted disorder segment [1]-[4]	Average Strength= 0.7432
Predicted disorder segment [27]-[81]	Average Strength= 0.6519
Predicted disorder segment [112]-[113]	Average Strength= 0.5300
Predicted disorder segment [260]-[274]	Average Strength= 0.6220
Predicted disorder segment [366]-[367]	Average Strength= 0.5105
Predicted disorder segment [392]-[580]	Average Strength= 0.9414
Predicted disorder segment [590]-[594]	Average Strength= 0.5136
Predicted disorder segment [686]-[690]	Average Strength= 0.5364
Predicted disorder segment [765]-[812]	Average Strength= 0.8098

ANCHOR:

Predicted Disordered Binding Regions			
	From	To	Length
1	98	103	6
2	371	403	33
3	440	452	13
4	459	465	7
5	471	488	18
6	498	516	19
7	563	588	26
8	801	809	9
Filtered Regions			
	From	To	Length
1	6	17	12

ModPred and PROSITE:

ModPred: Amidation (S4, H346, E579, V586, L797), Proteolytic cleavage (R22, Q235, R509, R791), Sumoylation (K394), Phosphorylation (S405), GPI anchor amidation (N418), ADP-ribosylation (R514)

PROSITE: Multicopper_oxidase1 (725-745, PROSITE entry PS00079), Multicopper_oxidase2 (730-741, PROSITE entry PS00080).

Structural modelling:

	Name	Title	Identity	Method	Oligo State	Ligands
☐	3sqr.1.A	laccase	31.29	X-ray, 1.7Å	monomer	5 x NAG, 3 x CU, 1 x MAN
☐	3gyr.1.A	Phenoxazinone synthase	17.43	X-ray, 2.3Å	homo-hexamer	6 x C2O, 18 x CU

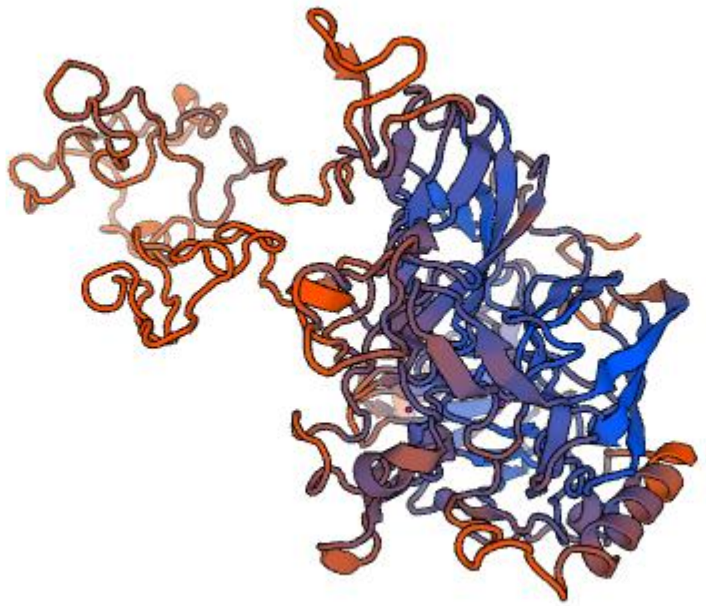

Model #1: Residues 83-787 of MVLG_03092T0 with 3sqr.1.A (31.29 % sequence identity) as a template

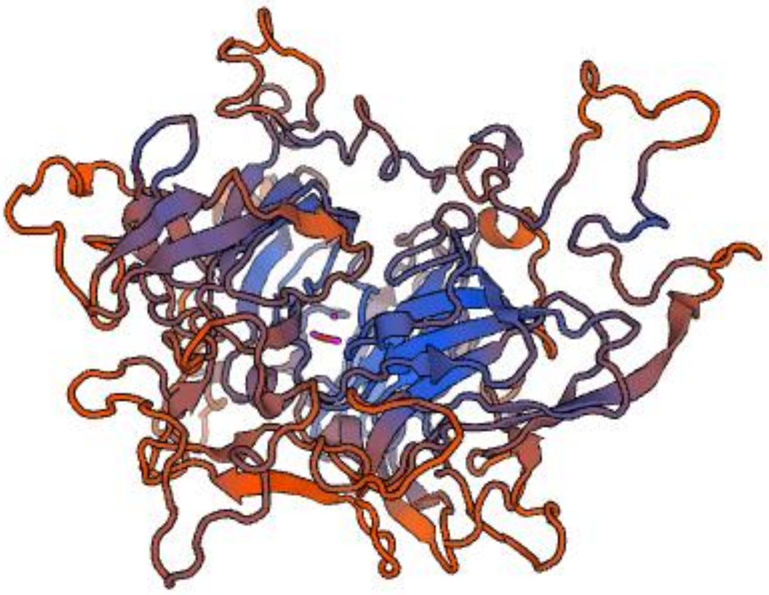

Model #2: Residues 70-756 of MVLG_03092T0 with 3gyr.1.A (17.43 % sequence identity) as a template

> MVLG_01652T0 (38.13%)- 237 aa

MFRTLYRLACLSLVPALLQAAPAPVDSSLGQGVSALKTRGSQSCRRVAQCTQAAPRNAVQTCNG
GKCGFACKSGYTWKDKKCQAASSGQATSGGTLLAAVSGHMVDAQLASNGITGFRAQSNGWNTNA
IASWFRTDSIQDSTNGHSWCYNEYDDSLPGFAPDVSVMLANFGGSNVRAGQAYCGLEAEVVTADG
RTVNLIIMDGFDSKWVRTPASIDVIYNAFGLLHGSTTNDKNTVESGVKWRLTGRRDSRYTFNSS

PONDR:

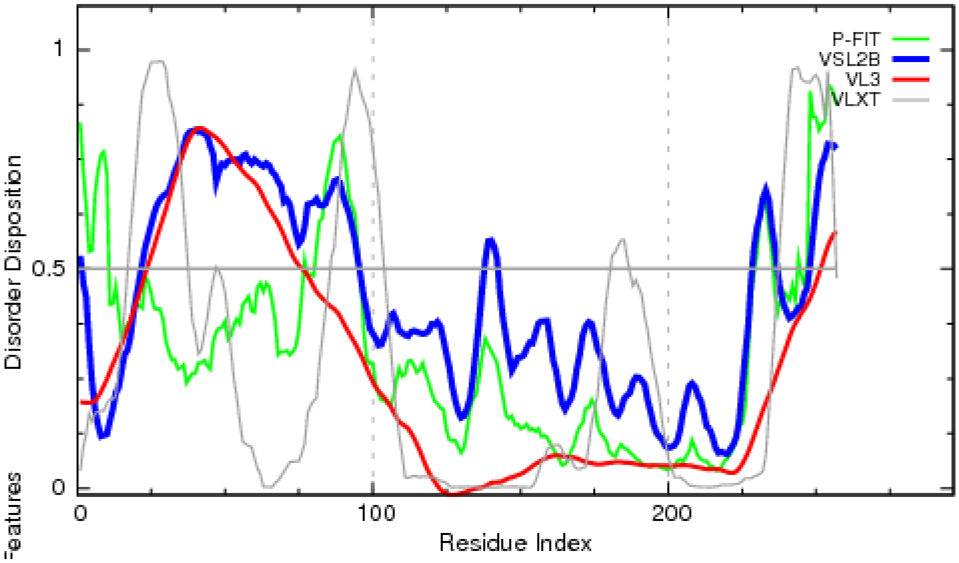

===============================PONDR VSL2 STATISTICS===============================

Predicted residues: 257	Number Disordered Regions: 4
Number residues disordered: 98	Longest Disordered Region: 74
Overall percent disordered: 38.13	Average Prediction Score: 0.4307
Predicted disorder segment [22]-[95]	Average Strength= 0.6931
Predicted disorder segment [138]-[142]	Average Strength= 0.5438
Predicted disorder segment [229]-[236]	Average Strength= 0.6128
Predicted disorder segment [248]-[257]	Average Strength= 0.7063

ANCHOR:

Predicted Disordered Binding Regions			
	From	To	Length
None			

Filtered Regions			
	From	To	Length
1	1	8	8

ModPred and PROSITE:

ModPred: Amidation (A9, G32, F222), Pyrrolidone carboxylic acid (Q19), Disulphide linkage (C67, C148), O-linked glycosylation (S92), Proteolytic cleavage (D136).
PROSITE: No identified domain recognition sites.

Structural modelling:

	Name	Title	Identity	Method	Oligo State	Ligands
☐	2hcz.1.A	Beta-expansin 1a	19.82	X-ray, 2.8Å	monomer	None
☐	1k4r.1.A	MAJOR ENVELOPE PROTEIN E	17.65	EM, 24.0Å	homo-trimer	None

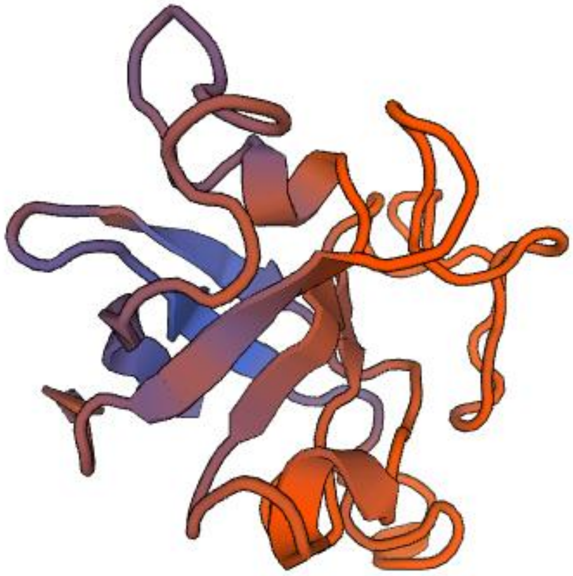

Model #1: Residues 123-244 of MVLG_01652T0 with 2hcz.1.A (19.82 % sequence identity) as a template

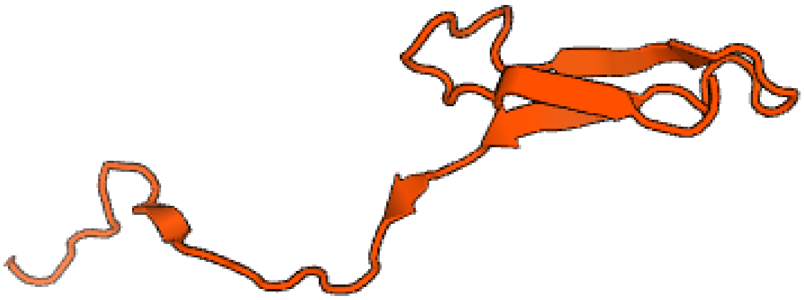

Model #2: Residues 130-191 of MVLG_01652T0 with 1k4r.1.A (17.65 % sequence identity) as a template

> MVLG_02018T0 (37.58%)- 165 aa

MLLLWLRVAALALAATGGPVSASPLKALDNALSSLNSTGPASKLTPIPTPPTISLLSKSKTYYPGDT
VFFKWDRAAPTMQSADLFIAYSGPLATVPICVTRDMLLQPDRGSMVLHSAYVIPWKELLGQKRATV
EGFIYFVVYSPTHYRFDTGVTGGKSDSFTIQHR

PONDR:

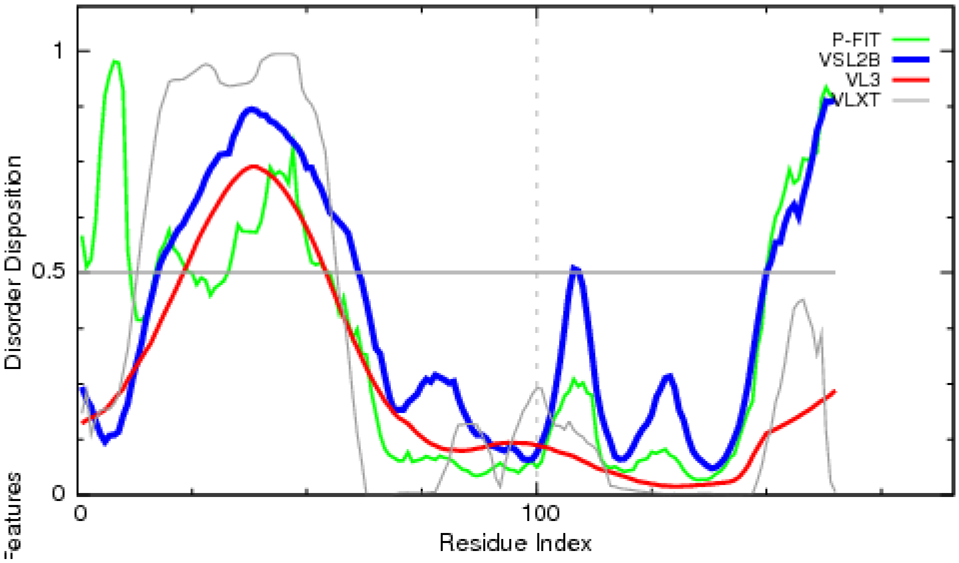

==============================PONDR VSL2 STATISTICS==============================

Predicted residues: 165
Number residues disordered: 62
Overall percent disordered: 37.58
Predicted disorder segment [18]-[61]
Predicted disorder segment [108]-[109]
Predicted disorder segment [150]-[165]

Number Disordered Regions: 3
Longest Disordered Region: 44
Average Prediction Score: 0.3924
Average Strength= 0.7153
Average Strength= 0.5065
Average Strength= 0.6979

ANCHOR:

Predicted Disordered Binding Regions			
	From	To	Length
None			

Filtered Regions			
	From	To	Length
1	1	8	8

ModPred and PROSITE:

ModPred: O-linked glycosylation (T49), Amidation (V140).

PROSITE: No identified domain recognition sites.

Structural modelling:

Name	Title	Coverage	Identity	Method	Oligo State	Ligands
2cp5.1.A	Restin	Created with Raphaël 2.2.0	23.44	NMR	monomer	None
1bcp.1.D	PERTUSSIS TOXIN	Created with Raphaël 2.2.0	35.19	X-ray, 2.7Å	hetero-oligomer	1 x ATP
2jgx.1.A	COMPLEMENT FACTOR H	Created with Raphaël 2.2.0	9.80	NMR	monomer	None
1tnr.1.A	TUMOR NECROSIS FACTOR BETA	Created with Raphaël 2.2.0	28.57	X-ray, 2.8Å	hetero-oligomer	None

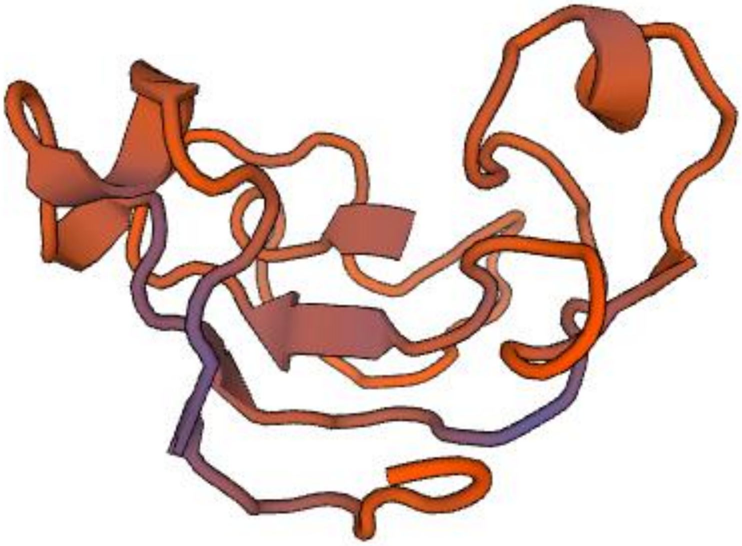

Model #1: Residues 123-244 of MVLG_02018T0 with 2cp5.1.A (23.44 % sequence identity) as a template

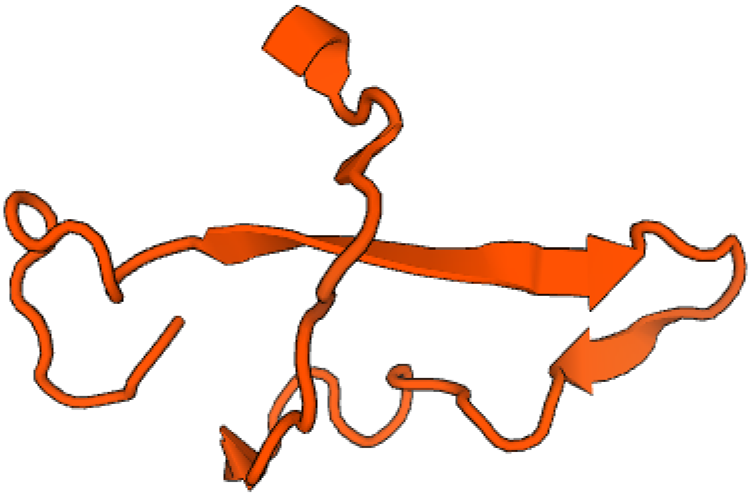

Model #2: Residues 123-244 of MVLG_02018T0 with 1bcp.1.D (35.19 % sequence identity) as a template

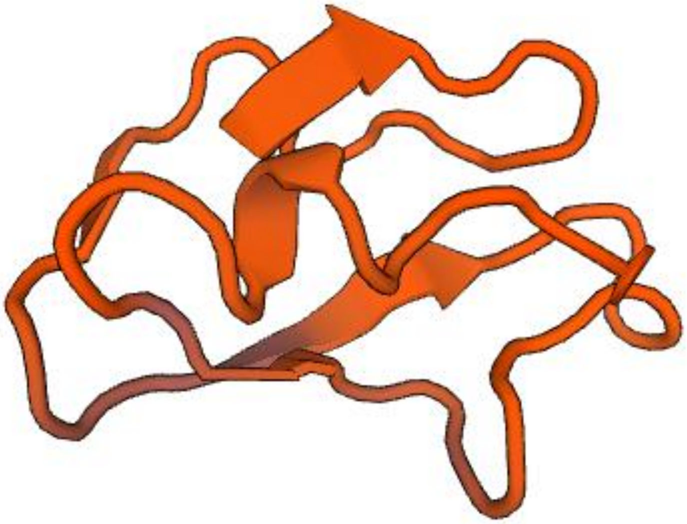

Model #3: Residues 123-244 of MVLG_02018T0 with 2jgx.1.A (9.80 % sequence identity) as a template

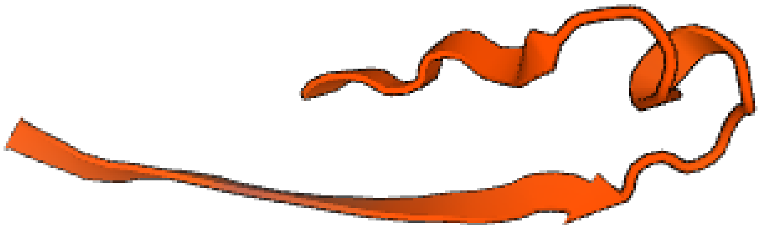

Model #4: Residues 123-244 of MVLG_02018T0 with 1tnr.1.A (28.57 % sequence identity) as a template

> MVLG_01824T0 (34.74%)- 639 aa

MVYTRPCRARAAVFASIVLCLIVVVVPTSVLALETAIVAPPDGNSSKGPDTETIPTLAGADDGETLF
QLNSTEMSPEEPLLEEPHWLPLLKRARRRKPPSEDAWSGETTTRIIGKLGIGAMQAIQSSDDELLVRH
RAAFGAKLRPRILTQDITETSLIGSSSLSNDPYLGIGRKCDFCAGGTFLSNGDIISVGGQPSEHTELGKP
GFAEDGFTGLRIFQPTSHRLLDNPKKVHIQSARWYASVVRVTDGSALIMGGSKKGQYNNDPKVDNP
TMEFFPSKGPQFYSKFLQDALDSNLFPLAFLLSGSGNIFVVANHVAMIYDWKHNREHRVKGVPGGI
VATYPGSGTAVLLPLTIKNNWISEVLICGGVFNTVNLTNPGFNVRADEPVSDQCARTSFPRGNSMSG
WEVEHMLSPRIMGDPVITPDGQVLIVGGAKTGTAGYGNAIGMDAAVPNLVPTLYNPDAPRGQRFSE
EFPPAKIERMYHSTSLLTTEGSVLTMGSSPNPRILTRLTYKSRFEVELIAPPYMTKKRPAILNYPQQIK
YNGRYTLTMSNPMGCDNVRVVLIDGGYATHALHMNQRSVELLVTSSNQSTITFQSPHDGTIWPPGP
AFLWITVCEGKIPSKGHKIMVGDGSNPPNYKAPF

PONDR:

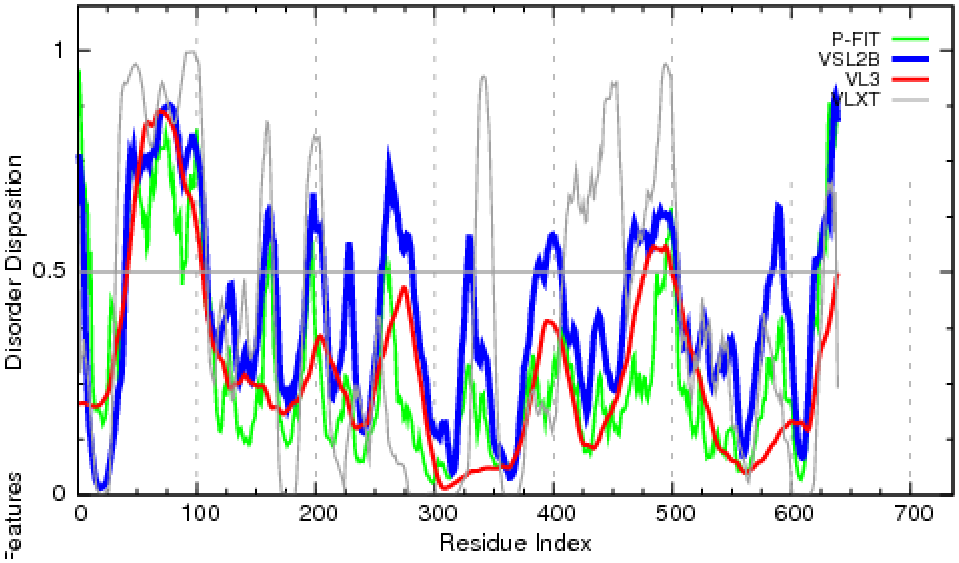

================================PONDR VSL2 STATISTICS================================

Predicted residues: 639	Number Disordered Regions: 12
Number residues disordered: 222	Longest Disordered Region: 69
Overall percent disordered: 34.74	Average Prediction Score: 0.4069
Predicted disorder segment [1]-[5]	Average Strength= 0.6753
Predicted disorder segment [40]-[108]	Average Strength= 0.7657
Predicted disorder segment [156]-[165]	Average Strength= 0.5868
Predicted disorder segment [193]-[206]	Average Strength= 0.5926
Predicted disorder segment [227]-[229]	Average Strength= 0.5466
Predicted disorder segment [256]-[281]	Average Strength= 0.6077
Predicted disorder segment [328]-[331]	Average Strength= 0.5577
Predicted disorder segment [386]-[390]	Average Strength= 0.5129
Predicted disorder segment [393]-[406]	Average Strength= 0.5531
Predicted disorder segment [463]-[504]	Average Strength= 0.5905
Predicted disorder segment [584]-[593]	Average Strength= 0.5737
Predicted disorder segment [620]-[639]	Average Strength= 0.6653

ANCHOR:

Predicted Disordered Binding Regions			
	From	To	Length
1	85	90	6
2	113	121	9
3	173	183	11
4	606	614	9
**Filtered Regions**			
	From	To	Length
1	14	27	14
2	66	69	4
3	363	366	4
4	427	430	4
5	510	511	2
6	560	560	1

ModPred and PROSITE:

ModPred: Amidation (A12, F276, Y635), Proteolytic cleavage (R92, R95, R136, R329, K331, R464, R467), Ubiquitination (K323), Methylation (R464).

PROSITE: No identified domain recognition sites.

Structural modelling:

	Name	Title	Identity	Method	Oligo State	Ligands
☐	5lxz.1.A	Secreted protein	21.57	X-ray, 1.5Å	monomer	2 x CU

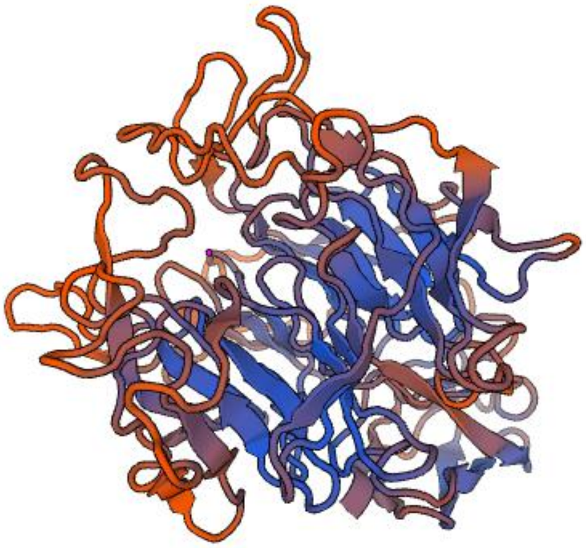

Model: Residues 99-627 of MVLG_01824T0 with 5lxz.1.A (21.57 % sequence identity) as a template

> MVLG_00243T0 (33.33%)- 528 aa

MSRMNTRAVLALVLVASLQALASPITGITFAAGPEQALAVRAPAPAAAATARSRVQQLGADRYV
RLATLHTRDDEHLLEARLLNHVMIEPGLAIRAADESESEDTEEAETDDEAEFEETVEEVIERRGNSRV
FATSARPILAGMRPPHVIPNPKDPDFGKLTATPVRTTTTTASAKSTATTTPRATTAPSTTMTTTTTTTT
TTTSTRDAAAASIPTGLGCFPSNVKSIPTGVNYTATDLASSWWCADSSEYAFIGFSYSVDECQSPSTL
LASFTRMRKQFGARDVRLYGACDATWFNDALVDAAASANLDVYHLIWFGFDGDDQRKSRYSAFV
KTMRTNPKAPFVFKNVAIGSEPLYDGVLSATNLVTEIFSMKSKMAPYGTKATFSEMPYGLQINNGA
PSTMAAADFVEGNVLPFFDSQATTGANAWGVVSWSLSYFASLAPGKIIRMTQTGWPSDQSVWKAN
TPTAVSSILSQASYYALLDSKCSWFNANGGIGWFAHIYSDDSLPGWGLLNNGNLKFPFAPKSSC

PONDR:

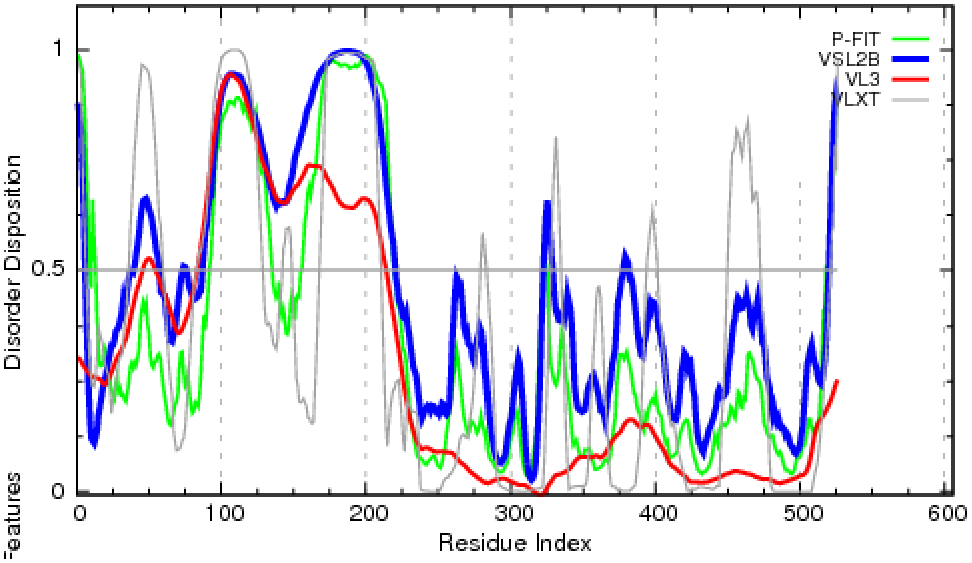

================================PONDR VSL2 STATISTICS==================================

Predicted residues: 528
Number residues disordered: 176
Overall percent disordered: 33.33
Predicted disorder segment [1]-[5] Average Strength= 0.7728
Predicted disorder segment [40]-[57] Average Strength= 0.5946
Predicted disorder segment [74]-[76] Average Strength= 0.5057
Predicted disorder segment [88]-[220] Average Strength= 0.8449
Predicted disorder segment [323]-[328] Average Strength= 0.5981
Predicted disorder segment [378]-[381] Average Strength= 0.5212
Predicted disorder segment [522]-[528] Average Strength= 0.7649

Number Disordered Regions: 7
Longest Disordered Region: 133
Average Prediction Score: 0.4540

ANCHOR:

Predicted Disordered Binding Regions			
	From	To	Length
1	79	96	18
2	116	151	36
3	155	166	12
4	214	221	8

ModPred and PROSITE:

ModPred: Amidation (A8, G359, W454, G496, Y502), Proteolytic cleavage (R62, K160, R283, R286, D322, K326, Y329, S330, D407), Carboxylation (E99, E106, E107, E109, E113,E117, E118, E121, E122), O-linked glycosylation (T169, T170, T171, T178, T180, T182, T186, S190, T196, T197, T198, T199, T200, T201, T202, T203, T204, T205, T206), Hydroxylation (P189), N-linked glycosylation (N397).

PROSITE: No identified domain recognition sites.

Structural modelling:

	Name	Title	Identity	Method	Oligo State	Ligands
	4wtr.1.A	beta-1,3-glucanosyltransferase	20.54	X-ray, 2.3Å	monomer	4 x BGC
	3pz9.1.A	Mannan endo-1,4-beta-mannosidase. Glycosyl Hydrolase family 5	16.02	X-ray, 1.4Å	monomer	None

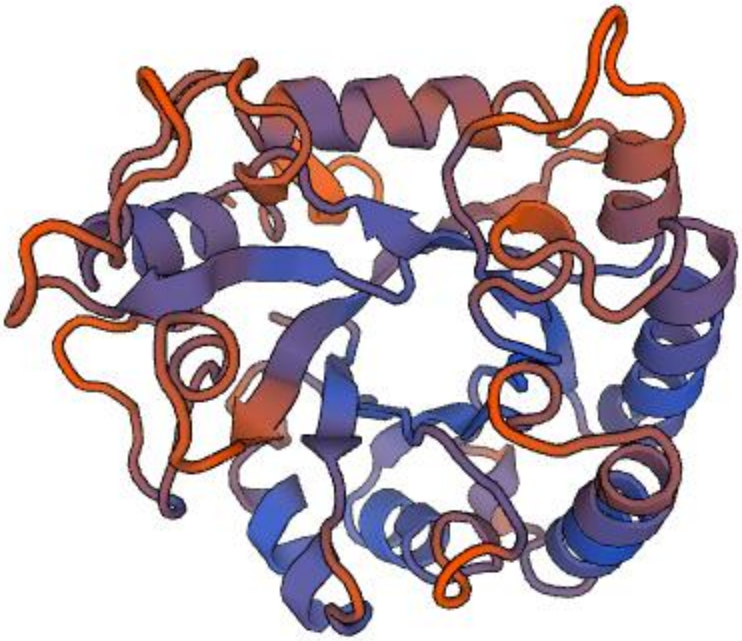

Model #1: Residues 250-528 of MVLG_00243T0 with 4wtr.1.A (20.54 % sequence identity) as a template

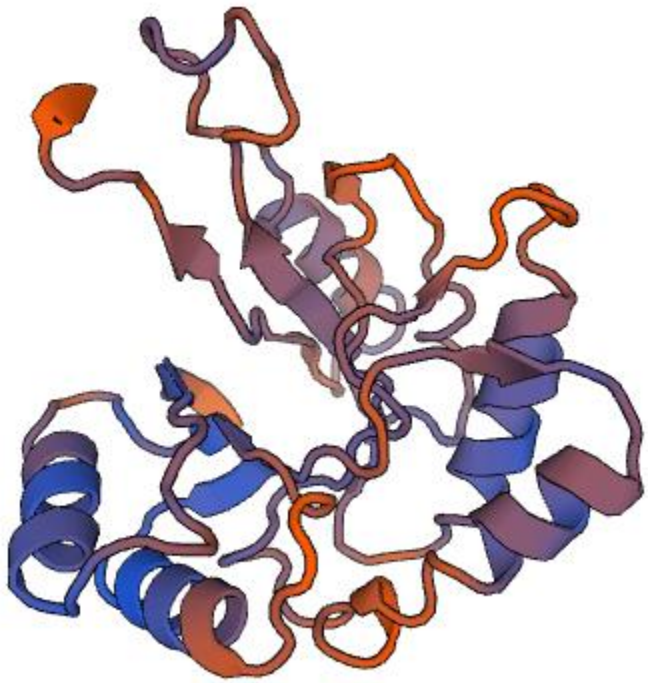

Model #2: Residues 251-458 of MVLG_00243T0 with 3pz9.1.A (16.02 % sequence identity) as a template

> MVLG_02872T0 (33.33%)- 441 aa

MFFRASTVFTSLLLVAPALAEAPSPDKRGFKLELHSRASHNPQHAGKAPKSGHAQPYAKRLKHGP
AHPRRGGAKAHQGHPPFLAATKAGSRQSFANLANVENIDWSVEVTFGSPPQRVPLFLSMGSSLSSV
ADQNIKSDAKTRYNPSKSLTARNMTKAQVDPNTGVTFITYKDKISIGGFEVSDQTFAVMTSTPNGDP
LERVYDSPVPWAGALALGRTSKGTPSLSFLENLIRSKVIDNAVCGISLTVEGGALFFGGIDSHSFKGKI
VWSPVETHYMEGFWTIKTGGWGWKGKVATGTAGLLQFAPENTYTYISAILGNKLFAGIKHHVDSK
TQRYLLPCNSNASDTIGFFIHNRMFPVPIPDLILFPSDSDPTMCHTALLQVTNKHILDDYTVVMGALH
MRSFYTILSYEKEHGGPAIGLAESSIKVMGGDPGPGGHSEK

PONDR:

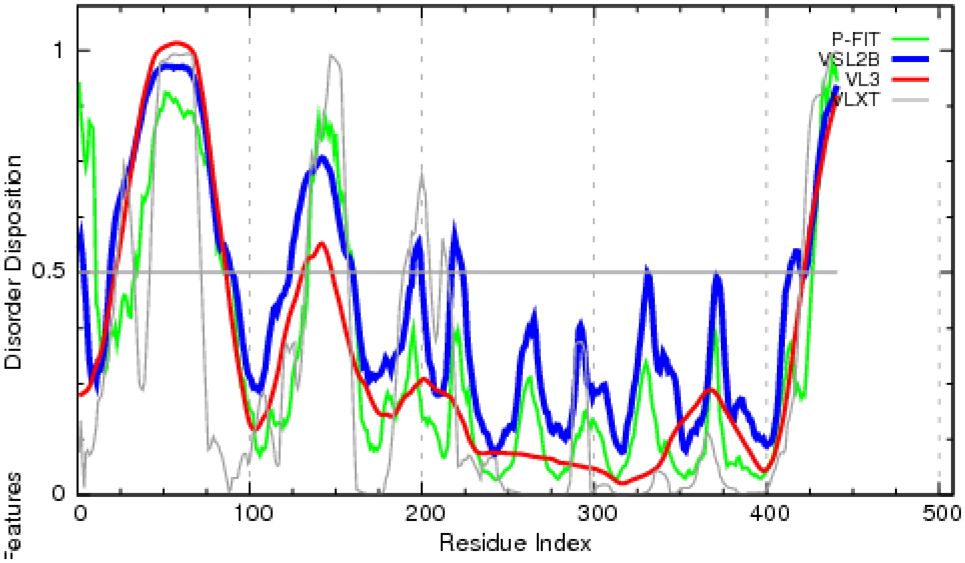

================================PONDR VSL2 STATISTICS================================

Predicted residues: 441
Number residues disordered: 147
Overall percent disordered: 33.33
Predicted disorder segment [1]-[3]
Predicted disorder segment [19]-[89]
Predicted disorder segment [124]-[159]
Predicted disorder segment [195]-[199]
Predicted disorder segment [218]-[223]
Predicted disorder segment [414]-[420]
Predicted disorder segment [423]-[441]

Number Disordered Regions: 7
Longest Disordered Region: 71
Average Prediction Score: 0.4227
Average Strength= 0.5550
Average Strength= 0.8052
Average Strength= 0.6611
Average Strength= 0.5464
Average Strength= 0.5458
Average Strength= 0.5277
Average Strength= 0.7575

ANCHOR:

Predicted Disordered Binding Regions			
	From	To	Length
1	29	38	10
2	50	65	16
3	73	108	36
4	118	128	11
5	169	175	7

Filtered Regions			
	From	To	Length
1	1	19	19

ModPred and PROSITE:

ModPred: Amidation (A16, D139, P271, P362), Proteolytic cleavage (K31, H35, D204), Acetylation (K59), Ubiquitination (K74), Methylation (K427)

PROSITE: Peptidase_A1 domain (105-422, PROSITE entry PS51767), Disulphide bridge (340-376)
Eukaryotic Aspartyl proteases (Aps) form peptidase family A1.
Known eukaryotic Aps in Fungi:

- Fungal proteases such as aspergillopepsin A (EC 3.4.23.18), candidapepsin (EC 3.4.23.24), mucoropepsin (EC 3.4.23.23) (mucor rennin), endothiapepsin (EC 3.4.23.22), polyporopepsin (EC 3.4.23.29), and rhizopuspepsin (EC 3.4.23.21).
- Yeast saccharopepsin (EC 3.4.23.25) (proteinase A) (gene PEP4). PEP4 is implicated in posttranslational regulation of vacuolar hydrolases.
- Yeast barrierpepsin (EC 3.4.23.35) (gene BAR1); a protease that cleaves α-factor and thus acts as an antagonist of the mating pheromone.
- Fission yeast sxa1 which is involved in degrading or processing the mating pheromones.

Structural modelling:

	Name	Title	Identity	Method	Oligo State	Ligands
☐	2psg.1.A	PEPSINOGEN	22.58	X-ray, 1.8Å	homo-dimer	None
☐	3zkm.1.A	BETA-SECRETASE 2	24.17	X-ray, 1.8Å	hetero-oligomer	None

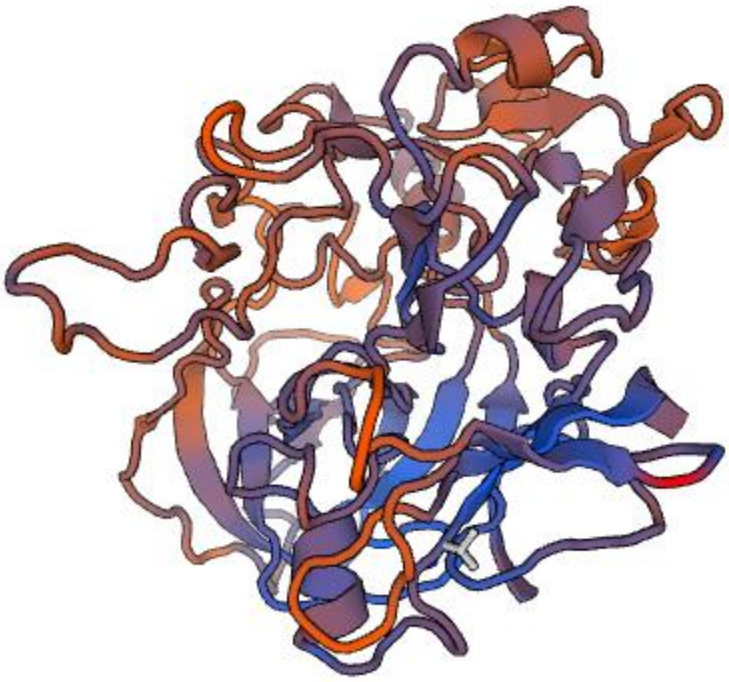

Model #1: Residues 29-425 of MVLG_02872T0 with 2psg.1.A (22.58 % sequence identity) as a template

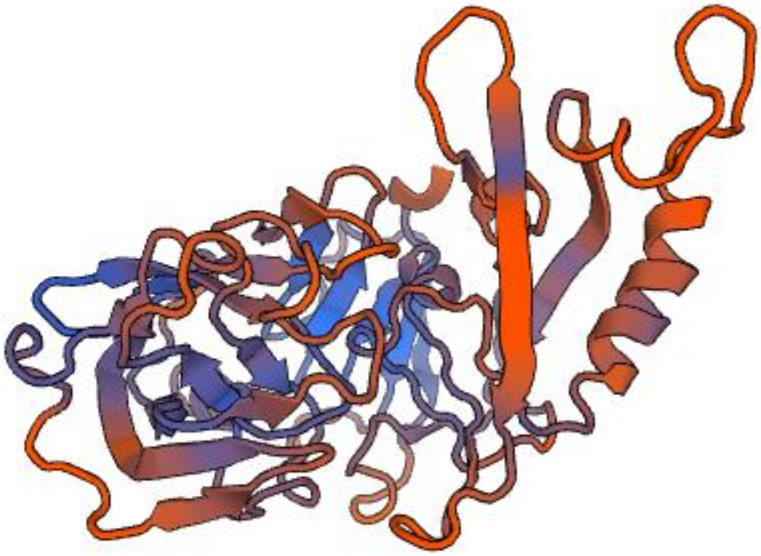

Model #2: Residues 96-428 of MVLG_02872T0 with 3zkm.1.A (24.17 % sequence identity) as a template

> MVLG_05120T0 (32.92%)

MLLKLTFTLALTLVSLGVSASQSNDTVEGRAMSPSSTKSTVTSPAYGKALKTDEAFSFVYYPAEG
DRQDSFFQITKLSLKAVEKDQFPTFDFANDLSSAQTEPVSVDFRLPPLEYFNANTVKTAKTGDSIEA
MLEISEQNFQKQVQTINVPLTITLHAHSQ

PONDR:

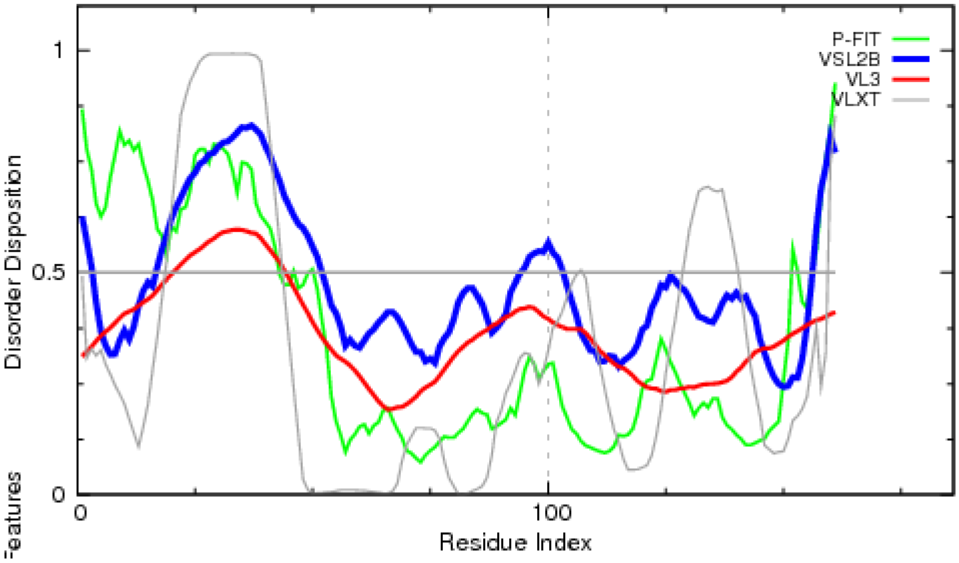

================================PONDR VSL2 STATISTICS================================

Predicted residues: 161
Number residues disordered: 53
Overall percent disordered: 32.92
Predicted disorder segment [1]-[3]
Predicted disorder segment [17]-[52]
Predicted disorder segment [95]-[103]
Predicted disorder segment [157]-[161]

Number Disordered Regions: 4
Longest Disordered Region: 36
Average Prediction Score: 0.4757
Average Strength= 0.5702
Average Strength= 0.6993
Average Strength= 0.5370
Average Strength= 0.7201

ANCHOR:

Predicted Disordered Binding Regions			
	From	To	Length
None			

Filtered Regions			
	From	To	Length
1	1	13	13

ModPred and PROSITE:

ModPred: Amidation (A20, Y115), Proteolytic cleavage (E28, R30, F56, D69, D107), Phosphorylation (S33), ADP-ribosylation (R109).

PROSITE: No identified domain recognition sites.

> MVLG_00885T0 (32.89%)- 76 aa

MRFSLAFFAVPFLVGQVVASVSDWSAKNGSFKCTSNEAGKGGKCMVCVHSNLDIFNTSLSQACG
NCGEFCTSNVHA

PONDR:

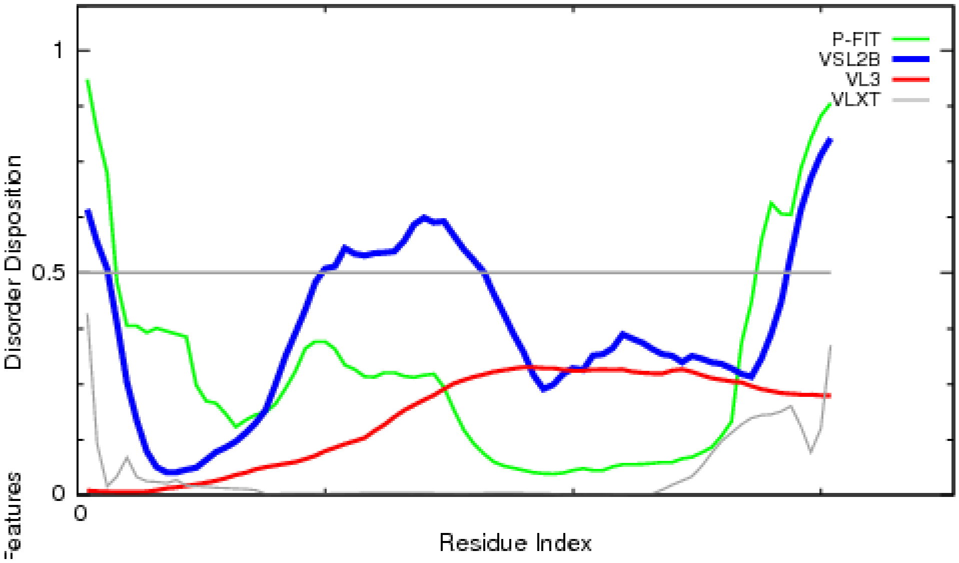

=============================PONDR VSL2 STATISTICS=============================

Predicted residues: 76	Number Disordered Regions: 3
Number residues disordered: 25	Longest Disordered Region: 17
Overall percent disordered: 32.89	Average Prediction Score: 0.3704
Predicted disorder segment [1]-[3]	Average Strength= 0.5726
Predicted disorder segment [25]-[41]	Average Strength= 0.5583
Predicted disorder segment [72]-[76]	Average Strength= 0.6922

ANCHOR:

Predicted Disordered Binding Regions			
	From	To	Length
None			

ModPred and PROSITE:

ModPred: Amidation (A6, L52).

PROSITE: No identified domain recognition sites.

Structural modelling:

No templates were found matching target sequence.

> MVLG_00677T0 (32.03%)- 215 aa

MRASVILPLCLGLLSYCASAAPSLEPPFTYQIPSPERIAELAQPYLINPENYSKTVYYRHEPRDHISSY
VMYAFQSMTNTSKWASAVVRHTYGHPKKDSNFEPTTTDIALSMPHIIPAPIPEGTPEGEGQPVQVEY
VRNRNVYRKVFLWHQPRSAVLGLEEKLQLGTLIDLGNIDPTTQPVMREYSEDMAKELGEGCPVSG
AMFERHMKDVQMYQ

PONDR:

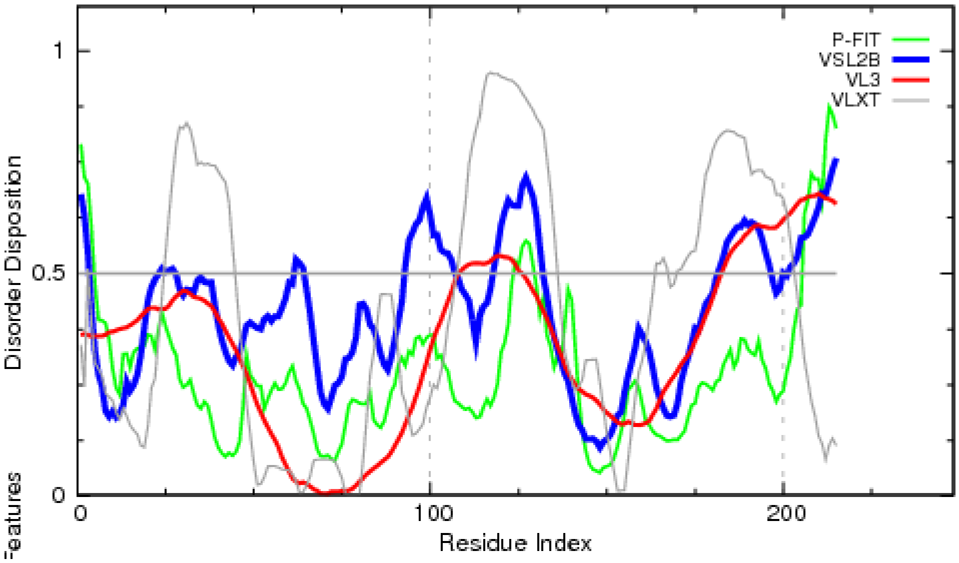

===============================PONDR VSL2 STATISTICS================================

Predicted residues: 215
Number residues disordered: 69
Overall percent disordered: 32.09
Number Disordered Regions: 8
Longest Disordered Region: 15
Average Prediction Score: 0.4189

Predicted disorder segment [1]-[3]	Average Strength= 0.6180
Predicted disorder segment [24]-[27]	Average Strength= 0.5074
Predicted disorder segment [62]-[64]	Average Strength= 0.5218
Predicted disorder segment [93]-[107]	Average Strength= 0.5807
Predicted disorder segment [118]-[131]	Average Strength= 0.6365
Predicted disorder segment [182]-[196]	Average Strength= 0.5799
Predicted disorder segment [200]-[200]	Average Strength= 0.5007
Predicted disorder segment [202]-[215]	Average Strength= 0.6257

ANCHOR:

Predicted Disordered Binding Regions			
	From	To	Length
1	142	150	9
2	163	171	9

Filtered Regions			
	From	To	Length
1	10	13	4
2	70	74	5

ModPred and PROSITE:

ModPred: Proteolytic cleavage (E135), ubiquitination (K191).

PROSITE: No identified domain recognition sites.

Structural modelling:

Name	Title	Identity	Method	Oligo State	Ligands
4x3i.1.A	Activity-regulated cytoskeleton-associated protein	27.91	X-ray, 1.8Å	monomer	1 x ALA-THR-ARG-ASN-PHE-SER-GLY
2d9d.1.A	BAG family molecular chaperone regulator 5	23.68	NMR	monomer	None
1rl2.1.A	PROTEIN (RIBOSOMAL PROTEIN L2)	20.00	X-ray, 2.3Å	monomer	None
1m6x.1.I	Flp recombinase	33.33	X-ray, 2.8Å	homo-tetramer	None

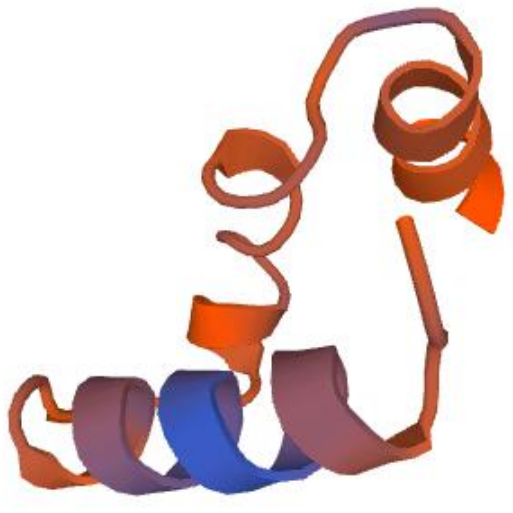

Model #1: Residues 60-102 of MVLG_00677T0 with 4x3i.1.A (27.91% sequence identity) as a template

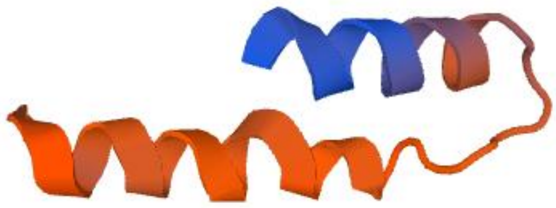

Model #2: Residues 155-193 of MVLG_00677T0 with 2d9d.1.A (23.68% sequence identity) as a template

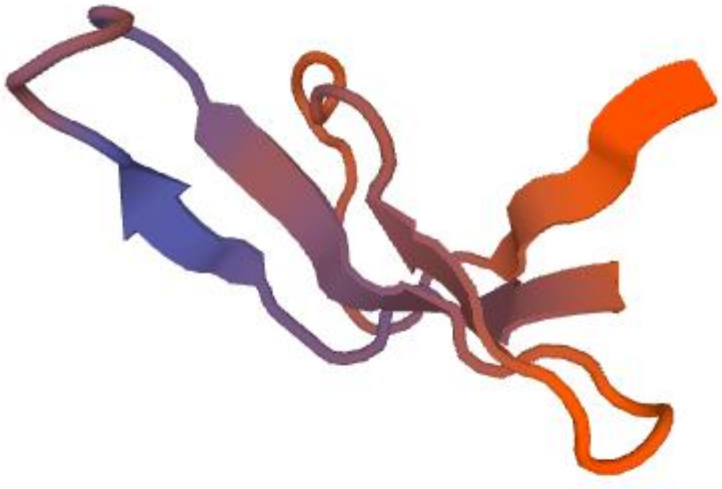

Model #3: Residues 128-172 of MVLG_00677T0 with 1rl2.1.A (20.00% sequence identity) as a template

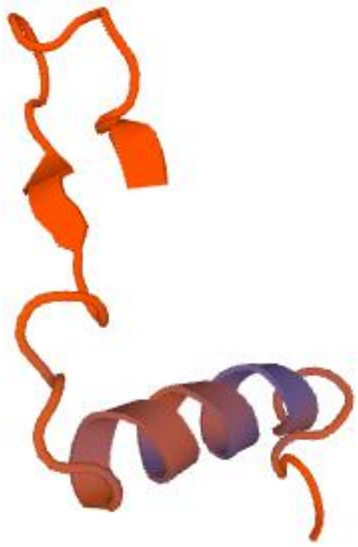

Model #4: Residues 58-99 of MVLG_00677T0 with 1m6x.1.I (33.33% sequence identity) as a template

> MVLG_02682T0 (31.70%)- 511 aa

MVQVHHRPTGIKRLFCWVLVLQLASLSAFAALDTAKREACQVFTNSPDLSKCPVDTIYVSARDP
KAKFKSIQQAINYLKATTANTPATILIGSGVYQEQLVVDGFASITLLGQTTSPRSSYAHNTVDINHNR
VLQKSDGYQNWLTSTLLVNGCADFKAYNLNLRQTAPVGIALAVAVMSSSGSFYACAIEGYQDTLF
LGPNKTRGYLYGCYVSGVVDFIYGWATLVVKDSQIMLLGEGTAYVAWRGAETTTSGAYFFGSTFD
AAQNSFGKIYPRTVAVGRAWNDKARIVILDCYLGSMIVPGIFAPWSYNPKDTRLSNEVFFGEYNSQG
PGSEAKSKIVDSRTGKVDVDHLLHVLDTKSAAPYYSLSTIFGQDILWIDGNFNVKAVSLASGGVGGS
STPGALAAAPALAASLPPPAPSAGKGTPLTKQDPKKPTQDPKKTEHNPTKQKSKHHSHPKRKKKAT
STKPQVDSGAGASSNHVSGGRPNLASLPKKRGHGSGGHPHHHHHHHHHG

PONDR:

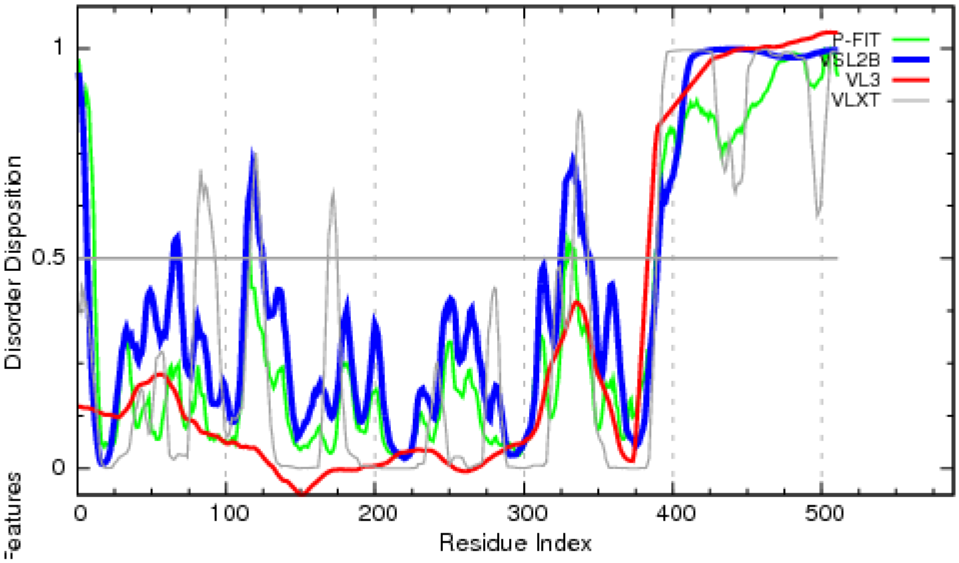

===============================PONDR VSL2 STATISTICS===============================

Predicted residues: 511	Number Disordered Regions: 6
Number residues disordered: 162	Longest Disordered Region: 122
Overall percent disordered: 31.70	Average Prediction Score: 0.4298
Predicted disorder segment [1]-[6]	Average Strength= 0.8039
Predicted disorder segment [65]-[68]	Average Strength= 0.5361
Predicted disorder segment [114]-[124]	Average Strength= 0.6219
Predicted disorder segment [325]-[342]	Average Strength= 0.6352
Predicted disorder segment [344]-[344]	Average Strength= 0.5044
Predicted disorder segment [390]-[511]	Average Strength= 0.9446

ANCHOR:

Predicted Disordered Binding Regions

	From	To	Length
1	400	415	16
2	444	511	68

Filtered Regions

	From	To	Length
1	368	389	22
2	422	426	5

ModPred and PROSITE:

ModPred: Amidation (N45, L111, Y363, Y364), N-linked glycosylation (N151), O-linked glycosylation (S252), Proteolytic cleavage (D340, K421, D469), Hydroxylation (P414, P415), ADP-ribosylation (R483), Methylation (K491).

PROSITE: No identified domain recognition sites.

Structural modelling:

	Name	Title	Identity	Method	Oligo State	Ligands
☐	1xg2.1.A	Pectinesterase 1	21.12	X-ray, 1.9Å	hetero-oligomer	None
☐	4pmh.1.A	Pectinesterase	22.18	X-ray, 1.8Å	monomer	None
☐	4pew.1.A	Putative secreted protein	17.53	X-ray, 1.5Å	monomer	1 x MG
☐	4xr6.1.A	Tail spike protein	12.24	X-ray, 1.8Å	homo-trimer	6 x GLC, 3 x GLA, 3 x RAM, 6 x NAG, 3 x NDG
☐	4xop.1.A	Tail spike protein	10.34	X-ray, 1.6Å	homo-trimer	6 x GLC, 3 x GLA, 3 x RAM, 6 x NAG, 3 x NDG

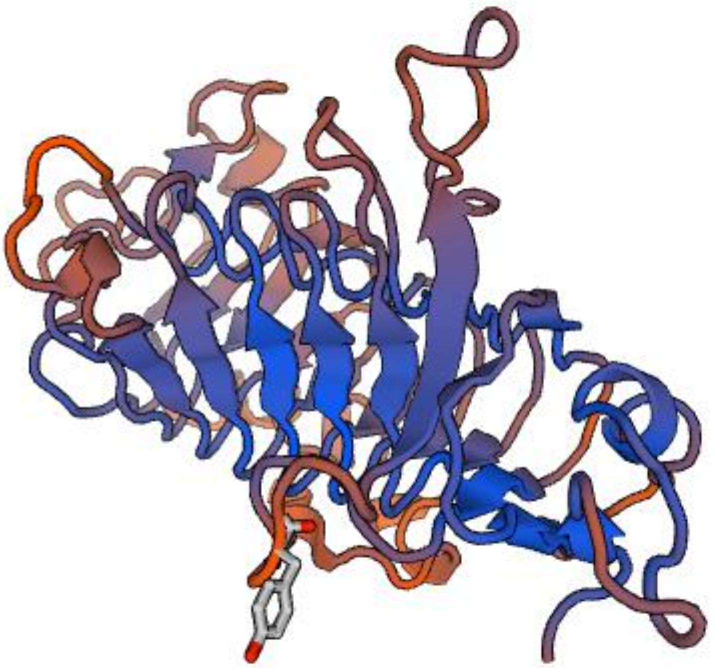

Model #1: Residues 54-387 of MVLG_02682T0 with 1xg2.1.A (21.12% sequence identity) as a template

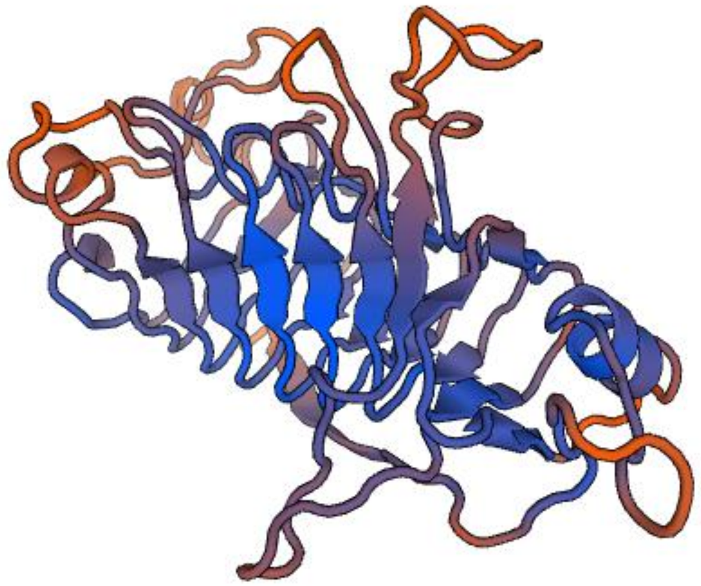

Model #2: Residues 41-332 of MVLG_02682T0 with 4pmh.1.A (22.18% sequence identity) as a template

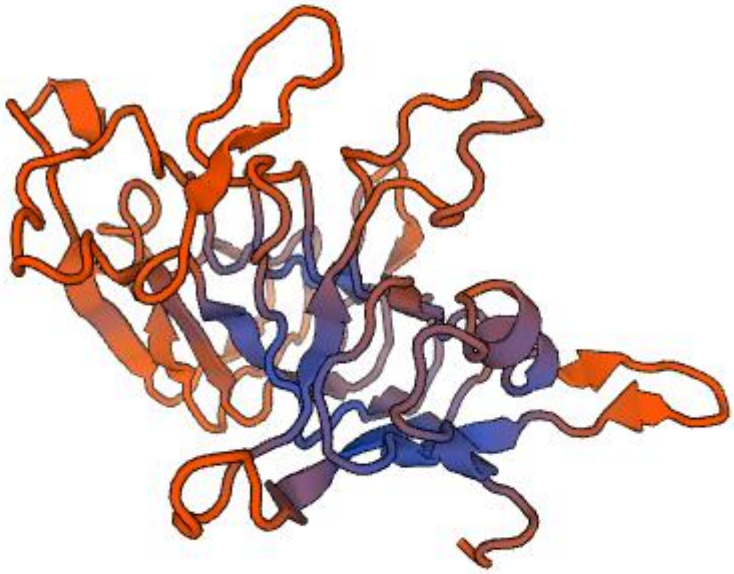

Model #3: Residues 52-305 of MVLG_02682T0 with 4pew.1.A (17.53% sequence identity) as a template

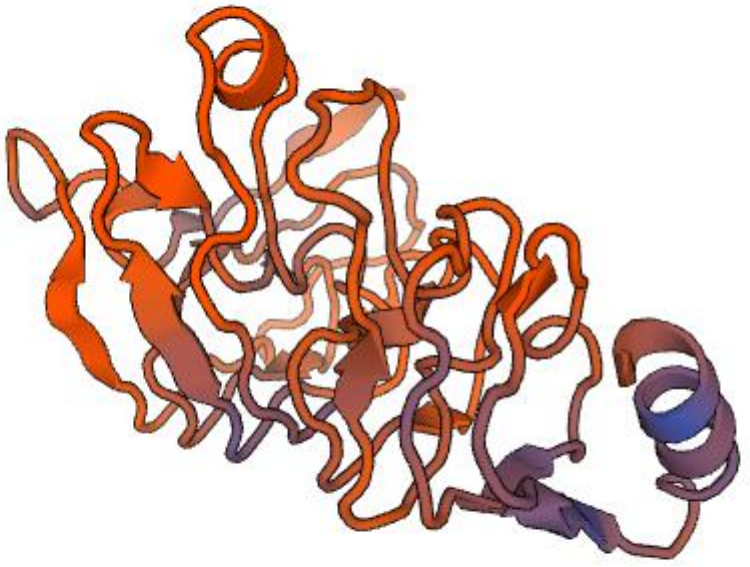

Model #4: Residues 71-284 of MVLG_02682T0 with 4xr6.1.A (12.24% sequence identity) as a template

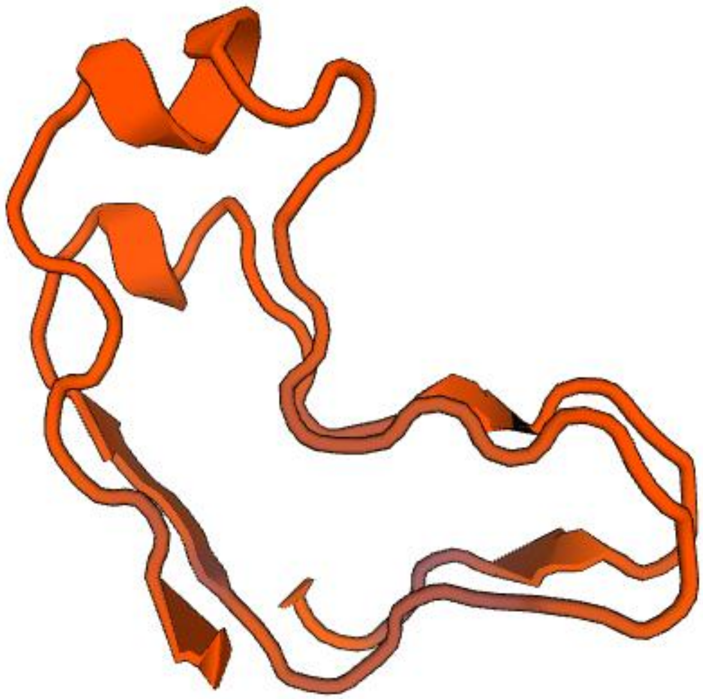

Model #5: Residues 153-215 of MVLG_02682T0 with 4xop.1.A (10.34% sequence identity) as a template

> MVLG_03747T0 (31.36%)- 456 aa

MKIILAALPLSLAALAGAHKHSSGHSSHRYRHHRASGVLQASSGTTTCIVDESGVGQDSTPKIMDA
FTKCQKNAKIVLNGNYLVKSLLYTPMLYNVEIELTGTLTYSDDIAYWSKPTTDTHGDGSYELYYQN
VTTFFFLQGEKIWLHGSPTSKTSKAEKQSTFNGNGQKWWDQFVKDKKAGNLHGIESTEYARPILLTI
GNAKNVRVEYINFLNGPFWNIFITHSKQVTMSNINIDAVSKSDSLPYNTDGVDTYNSDDVTLLDFNV
NNADDCVSLKPNSTNVEVGRVNCNGSHGISVGSLGQYVDSYDIVENVYIHDISMSNAQAGARIKAW
PDRNGTAKDAGGGSGYVKNITFQNFVNKNVDEPLLITSCYMNSNEYCTKFPSKMTVSDVHYINVTG
TSSGKYKDVVALLDCSKECTGITAIGTHLSLPTPSTPPVYNCHNVDSEKQLDFHCTEL

PONDR:

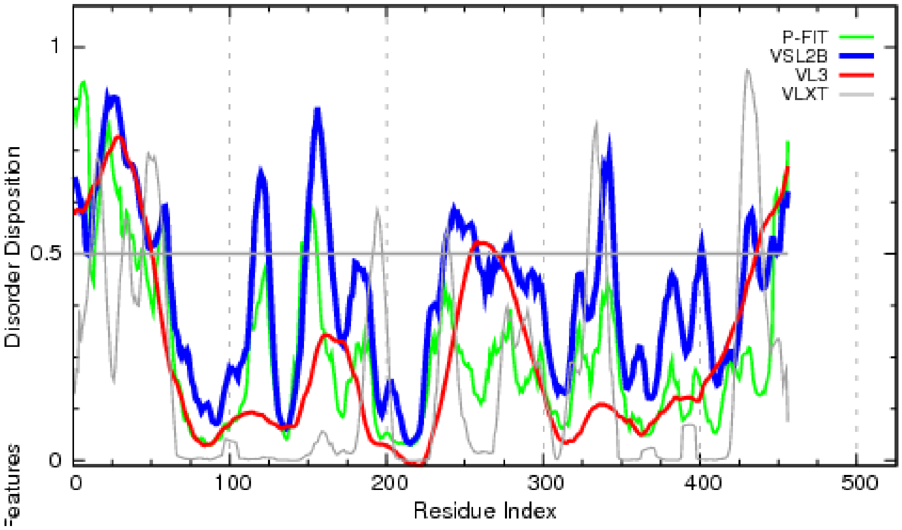

==============================PONDR VSL2 STATISTICS==============================

Predicted residues: 456	Number Disordered Regions: 10
Number residues disordered: 143	Longest Disordered Region: 51
Overall percent disordered: 31.36	Average Prediction Score: 0.3970
Predicted disorder segment [1]-[9]	Average Strength= 0.6124
Predicted disorder segment [11]-[61]	Average Strength= 0.6787
Predicted disorder segment [116]-[124]	Average Strength= 0.6396
Predicted disorder segment [149]-[164]	Average Strength= 0.6972
Predicted disorder segment [237]-[257]	Average Strength= 0.5645
Predicted disorder segment [275]-[280]	Average Strength= 0.5112
Predicted disorder segment [336]-[345]	Average Strength= 0.6419
Predicted disorder segment [401]-[401]	Average Strength= 0.5162
Predicted disorder segment [429]-[436]	Average Strength= 0.5581
Predicted disorder segment [445]-[456]	Average Strength= 0.5574

ANCHOR:

Predicted Disordered Binding Regions

	From	To	Length
1	307	314	8

Filtered Regions

	From	To	Length
1	1	13	13
2	136	139	4
3	213	213	1
4	215	218	4

ModPred and PROSITE:

ModPred: Proteolytic cleavage (R34, K340), Ubiquitination (K340), Amidation (K415).

PROSITE: POLYGALACTURONASE (Polygalacturonase active site, 286-299, PROSITE entry PS00502) Polygalacturonase (EC 3.2.1.15) (PG) (pectinase) catalyzes the random hydrolysis of 1,4-α-D-galactosiduronic linkages in pectate and other galacturonans. In plant bacterial pathogens such as Erwinia carotovora or Pseudomonas solanacearum and fungal pathogens such as Aspergillus niger, polygalacturonase is involved in maceration and soft-rotting of plant tissue.

Structural modelling:

Name	Title	Identity	Method	Oligo State	Ligands
4c2l.1.A	ENDO-XYLOGALACTURONAN HYDROLASE A	37.40	X-ray, 1.8Å	monomer	1 x NAG-NAG, 1 x NAG, 1 x MAN
3lmw.1.A	Iota-carrageenase, CgiA	14.05	X-ray, 2.6Å	monomer	1 x NI, 1 x CA
4xr6.1.A	Tail spike protein	17.69	X-ray, 1.8Å	homo-trimer	6 x GLC, 3 x GLA, 3 x RAM, 6 x NAG, 3 x NDG
3lmw.1.A	Iota-carrageenase, CgiA	10.56	X-ray, 2.6Å	monomer	1 x NI, 1 x CA
5gai.1.Z	Tail fiber protein	17.82	EM	hetero-oligomer	None

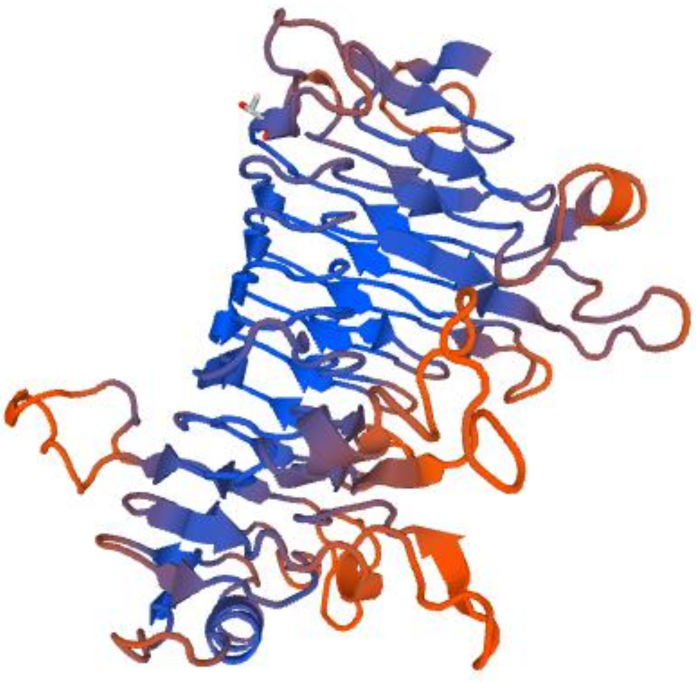

Model #1: Residues 46-455 of MVLG_03747T0 with 4c2l.1.A (37.40% sequence identity) as a template

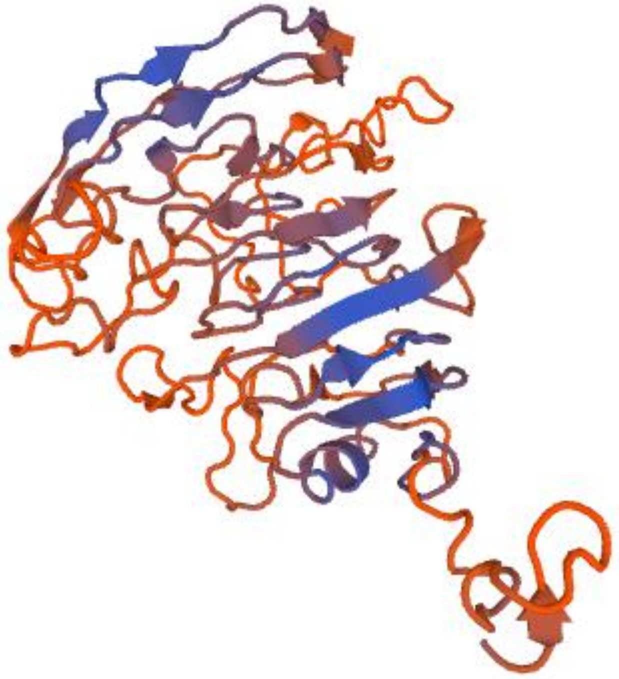

Model #2: Residues 24-401 of MVLG_03747T0 with 4xr6.1.A (17.69% sequence identity) as a template

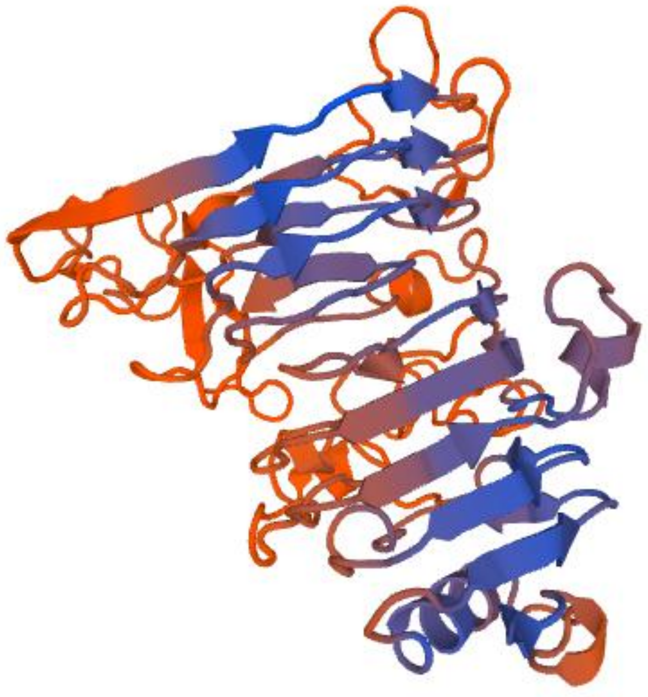

Model #3: Residues 46-422 of MVLG_03747T0 with 3lmw.1.A (14.05% sequence identity) as a template

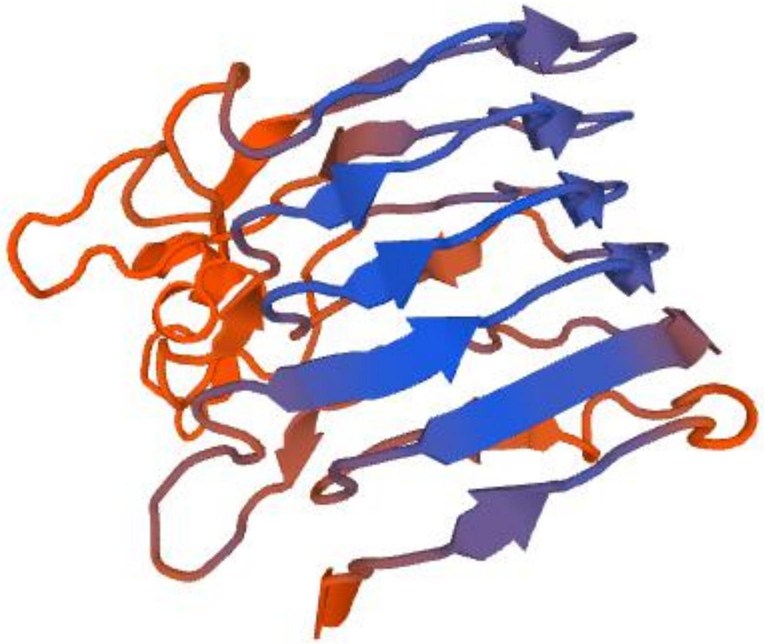

Model #4: Residues 199-397 of MVLG_03747T0 with 3lmw.1.A (10.56% sequence identity) as a template

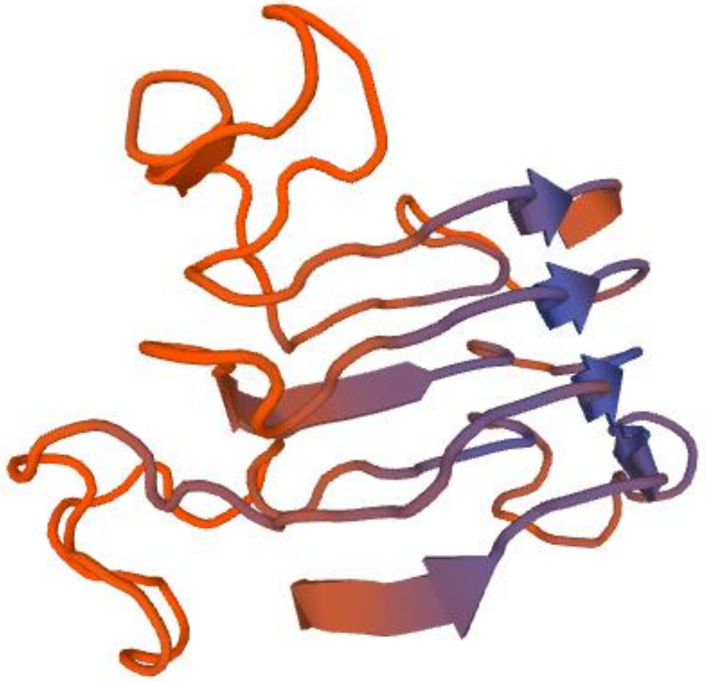

Model #5: Residues 226-362 of MVLG_03747T0 with 5gai.1.Z (17.82% sequence identity) as a template

> MVLG_05737T0 (31.08%)- 473 aa

MMTPLSTLVVAAATLSSLLQVAATQAATTAPMLSSYFPAYTEGATVAWNQTKLAMYFVDITTKD
GFEIGPNQPLDGIKKFTSQAYANGAKPMVTLGSWNGSLYFSKQLSTPEGRTKLASQLQNYLYYKEF
KGVDVSWLYPAQQGIGCNTVSPKDTDNFLKFLKTLRGWLGMGYLISIAAPPGGFLTGNGTEHVKD
YSEWATVLDHINVMTYDYTGPWSSKTGPLSPMHSCASGGGVTAAVKYWTSSGFPAEKIFISIPSYAI
SFTLKSSTLEKTYMTDGDGGTFNYSSLIYQSFSSIPKGEAADSNEPTTDGCGVVTANYTGQWHYTSL
IKEGLLAHDGSKGLKGYARYMDGCSQTPFLFNPTNKHFIAYEDAASASIKAGFARDNGLKGVTVFT
SEGFDDTVYDAIVTDLNRPKKELESGGATGKSDTPQAQAGGKTTKPSTPPPSSKQPQDMPKKASHG
AGILGKMNLRAR

PONDR:

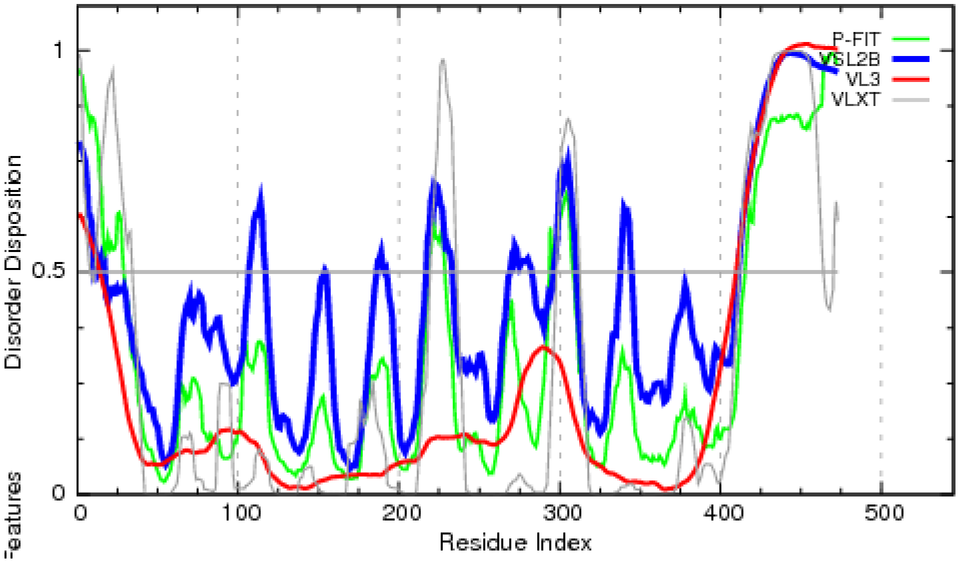

==============================PONDR VSL2 STATISTICS==============================

Predicted residues: 473	Number Disordered Regions: 11
Number residues disordered: 147	Longest Disordered Region: 62
Overall percent disordered: 31.08	Average Prediction Score: 0.4255
Predicted disorder segment [1]-[12]	Average Strength= 0.6728
Predicted disorder segment [14]-[15]	Average Strength= 0.5140
Predicted disorder segment [17]-[17]	Average Strength= 0.5080
Predicted disorder segment [107]-[117]	Average Strength= 0.5893
Predicted disorder segment [154]-[154]	Average Strength= 0.5021
Predicted disorder segment [187]-[192]	Average Strength= 0.5213
Predicted disorder segment [218]-[233]	Average Strength= 0.6169
Predicted disorder segment [269]-[282]	Average Strength= 0.5254
Predicted disorder segment [296]-[310]	Average Strength= 0.6447
Predicted disorder segment [338]-[344]	Average Strength= 0.5982
Predicted disorder segment [412]-[473]	Average Strength= 0.9104

ANCHOR:

Predicted Disordered Binding Regions			
	From	To	Length
1	399	411	13
2	455	473	19
**Filtered Regions**			
	From	To	Length
1	290	290	1
2	393	394	2

ModPred and PROSITE:

ModPred: Acetylation (K160, K467), Amidation (Y196, S197, V240, Y275, Y370, Y404), Ubiquitination (K220, K467), Proteolytic cleavage (D405, T409, D410, E419), Phosphorylation (T429, T443), Hydroxylation (P446), ADP-ribosylation (R471).

PROSITE: No identified domain recognition sites.

Structural modelling:

	Name	Title	Identity	Method	Oligo State	Ligands
☐	4txg.1.A	Chitinase	22.56	X-ray, 1.8Å	monomer	11 x CS

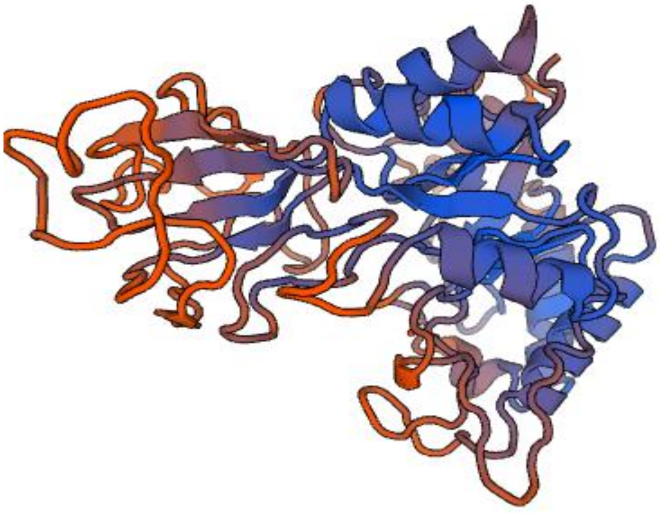

Model #1: Residues 28-413 of MVLG_057377T0 with 4txg.1.A (22.56% sequence identity) as a template

> MVLG_01520T0 (31.00%)- 958 aa

MRVGNSLAVLAAAAAIVPAAFAQKTQVFGRYIIVYPESNKEGKALHARDHLSNLRAKSGVEPLEV
VQEYHMPGVLVGQSVNAPGVTKEQLEKMPGVKAVYPVFDYSFAAVQQQKEEPPSQSFQQHHNHR
KVKESGAAPPHLELRDQQFLGQKNMPNTTGGFSPHRMTSIDILHKRGFFGQGVKSCFIEGHTQYTHP
LLGKRGCFGSKNCAIQFGADLVGTDPNHPQPGPDPHADCDVRSTHILGQMVAPENRFDFVGAIPQA
EIGWYSIFPCGGGGATGDIIIGAFLKAADDGCKVISNSLISSVGWNDNDLGPITLNKLAEEKGVFAVS
AWGVSRDEGLFYPAGPATGTEGVGAAYVDLNQYPFAYTLTFENGEATLPYISVYPIPYDDSFEVYFL
STSSTDTAATGCDDLPHDTPDLTNRAVVVQRASCGFETQMANVRKFGARVLLVVNYPASVGWPAP
YFDGIAPSVPFPVGMIHSDGAKLLEYYRKNSNGLKLNFKDRTLIHPVNADTGGKISFYSSYGPDNSLT
TGPTFGVPANQIAGIRPNGSVGTIDATSSPITNAIATLVLGARKNDNLKPDELRSLLATTAKPISIHPRA
DGEPLETTTLAGSGLVNALRAVEAQTLVTPFSFKINDTAHVQKEQQLTLKNMGHASITYTFDSTAA
QTKMTYDGGAKQDIVPSSLPTVLQEAEAKVSFDKTSITIEAGQTATVKVTITPPQLTAREKDYFPVYS
GFINIHASNKQEFHVSYFGLAADIVDMPIIDVSTSFASAFRSGLPQGLTYPYLLDNSPNSVKVPTQLTT
FDRSIGVGVFIRFAQATRHVTVDVIAGNSTFKGTLPSHEGRNHRRSLNAADENHLVARRLARQSRA
DPNQLYTDVQVLGRIYEKKNQARDDKGSPDALVVFKGSMHKDLSMDGQASDLPDGMPYRVLVRA
LKTTADPSLEASWESWVSPPVQFKS

PONDR:

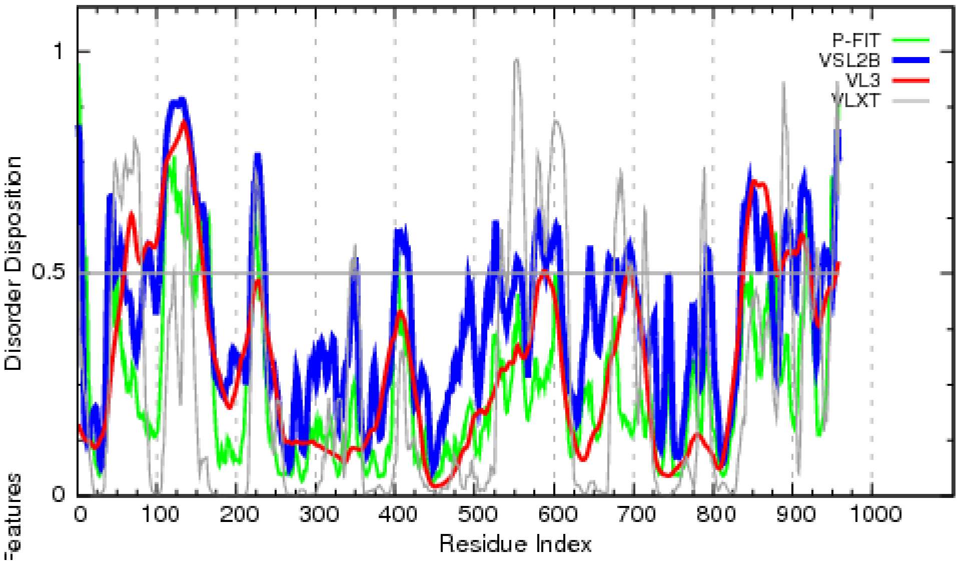

================================PONDR VSL2 STATISTICS================================

Predicted residues: 958	Number Disordered Regions: 21
Number residues disordered: 297	Longest Disordered Region: 60
Overall percent disordered: 31.00	Average Prediction Score: 0.3976
Predicted disorder segment [1]-[5]	Average Strength= 0.7563
Predicted disorder segment [39]-[47]	Average Strength= 0.6197
Predicted disorder segment [50]-[58]	Average Strength= 0.5426
Predicted disorder segment [85]-[93]	Average Strength= 0.5318
Predicted disorder segment [104]-[163]	Average Strength= 0.7643
Predicted disorder segment [219]-[235]	Average Strength= 0.6649
Predicted disorder segment [349]-[351]	Average Strength= 0.5175
Predicted disorder segment [401]-[417]	Average Strength= 0.5639
Predicted disorder segment [524]-[530]	Average Strength= 0.5664
Predicted disorder segment [551]-[558]	Average Strength= 0.5182

Predicted disorder segment [574]-[607]	Average Strength= 0.5686
Predicted disorder segment [643]-[646]	Average Strength= 0.5319
Predicted disorder segment [668]-[679]	Average Strength= 0.5201
Predicted disorder segment [689]-[699]	Average Strength= 0.5432
Predicted disorder segment [792]-[796]	Average Strength= 0.5282
Predicted disorder segment [835]-[857]	Average Strength= 0.6365
Predicted disorder segment [859]-[873]	Average Strength= 0.5794
Predicted disorder segment [887]-[900]	Average Strength= 0.5821
Predicted disorder segment [906]-[924]	Average Strength= 0.6381
Predicted disorder segment [938]-[946]	Average Strength= 0.5367
Predicted disorder segment [952]-[958]	Average Strength= 0.7087

ANCHOR:

Predicted Disordered Binding Regions			
	From	To	Length
1	96	110	15
2	140	150	11
3	166	187	22
4	204	214	11
5	565	570	6
6	615	621	7
7	627	634	8
8	875	885	11
9	900	908	9
10	927	933	7
**Filtered Regions**			
	From	To	Length
1	266	269	4
2	586	588	3
3	812	815	4
4	859	860	2

ModPred and PROSITE:

ModPred: Amidation (A12, I259, F392), Proteolytic cleavage(R30, Y31, D216, D254, W332, L340, Y395, R584, Y733, D923, R928), Ubiquitination (K40, K57), ADP-ribosylation (R546), O-linked glycosylation (T716), Phosphorylation (S897).

PROSITE: No identified domain recognition sites.

Structural modelling:

Name	Title	Identity	Method	Oligo State	Ligands
3eif.1.A	C5a peptidase	19.82	X-ray, 1.9Å	monomer	1 x CA, 1 x MLA
4yn3.1.A	Cucumisin	19.24	X-ray, 2.0Å	monomer	1 x MAN, 1 x BMA, 4 x NAG, 1 x FUC
4i0w.1.B	Protease CspB	14.39	X-ray, 1.6Å	hetero-oligomer	None
3lxu.1.A	Tripeptidyl-peptidase 2	15.80	X-ray, 3.1Å	homo-dimer	None
1r6v.1.A	subtilisin-like serine protease	19.59	X-ray, 1.7Å	monomer	1 x CA
1y9z.1.A	alkaline serine protease	20.21	X-ray, 1.4Å	monomer	2 x CA, 1 x PMS
4mzd.1.A	Nisin leader peptide-processing serine protease NisP	18.84	X-ray, 1.1Å	monomer	None

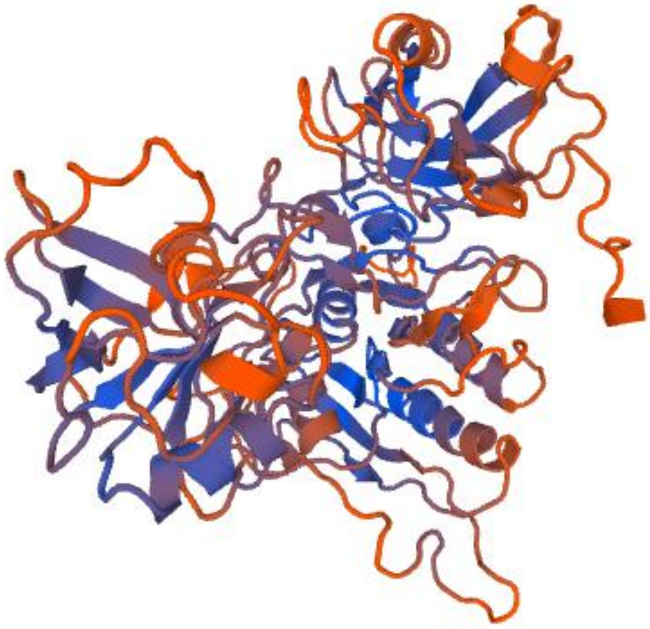

Model #1: Residues 163-766 of MVLG_01520T0 with 3eif.1.A (19.82% sequence identity) as a template

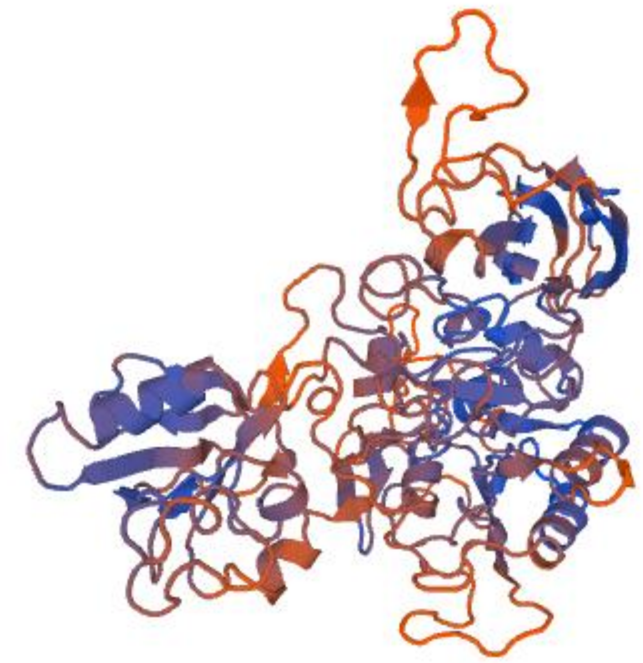

Model #2: Residues 144-754 of MVLG_01520T0 with 4yn3.1.A (19.24% sequence identity) as a template

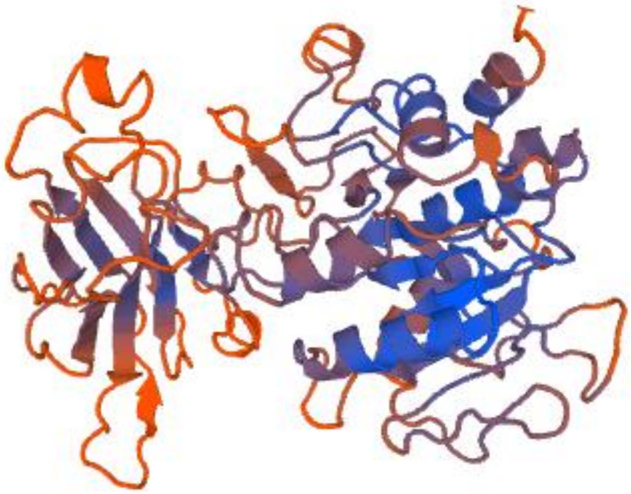

Model #3: Residues 167-629 of MVLG_01520T0 with 4i0w.1.B (14.39% sequence identity) as a template

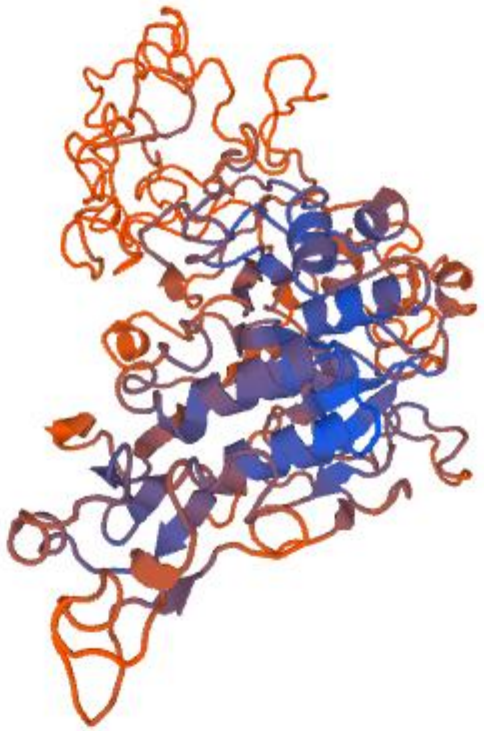

Model #4: Residues 19-626 of MVLG_01520T0 with 1r6v.1.A (19.59% sequence identity) as a template

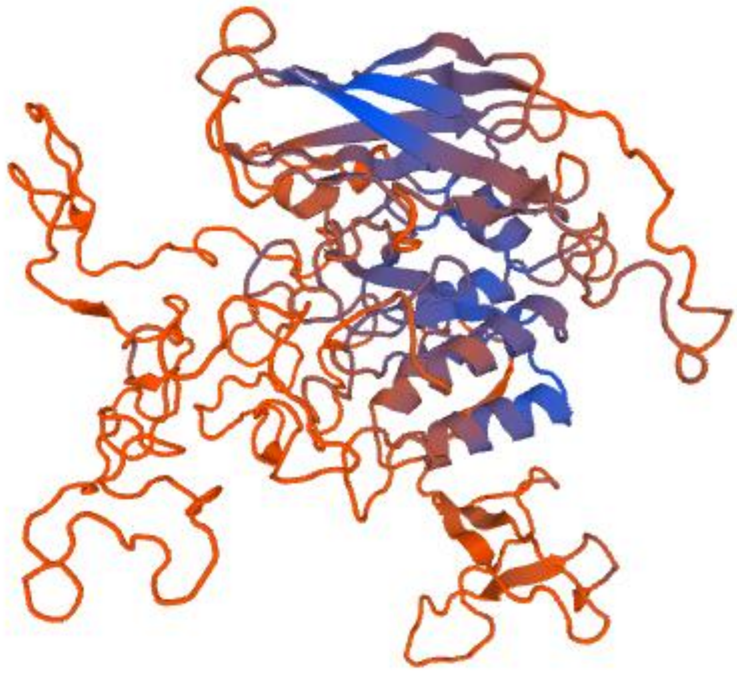

Model #5: Residues 177-755 of MVLG_01520T0 with 3lxu.1.A (15.80% sequence identity) as a template

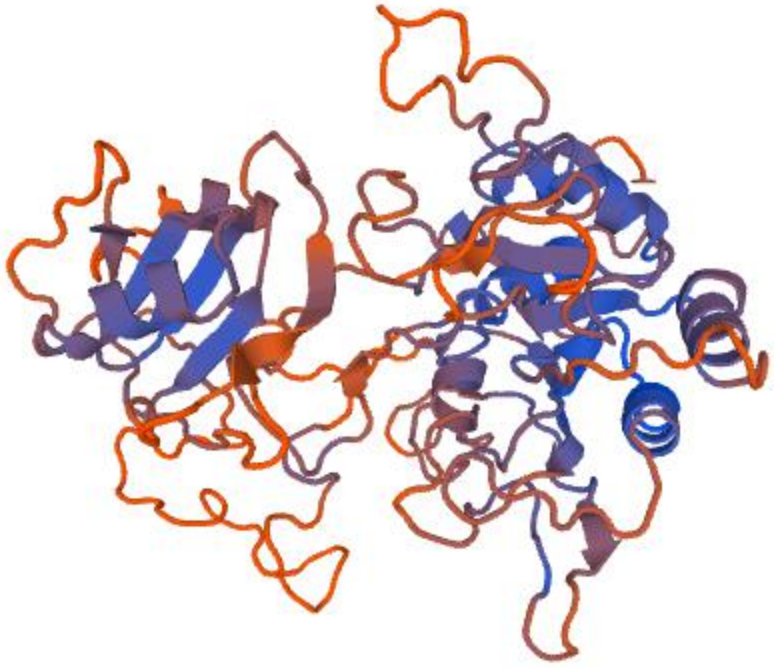

Model #6: Residues 168-629 of MVLG_01520T0 with 1y9z.1.A (20.21% sequence identity) as a template

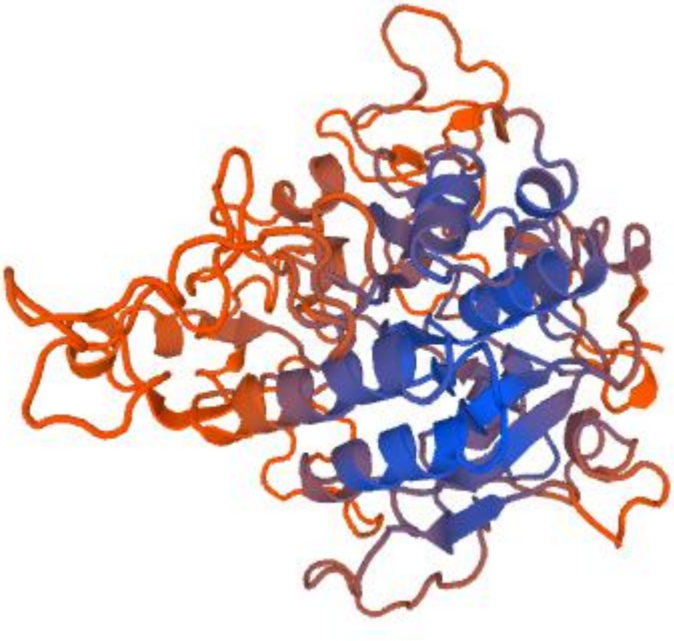

Model #7: Residues 145-627 of MVLG_01520T0 with 4mzd.1.A (18.84% sequence identity) as a template

> MVLG_04107T0 (30.00%)- 100 aa

MKYSLVFVTLVLMAAINVSAIPADLTKPTSTSSEVDKVHDPKKYAPPAVISFISKANATVARQTKD
CCNYCLKRRRDGVKLNSCYAICLWSSGKWTTKCP

PONDR:

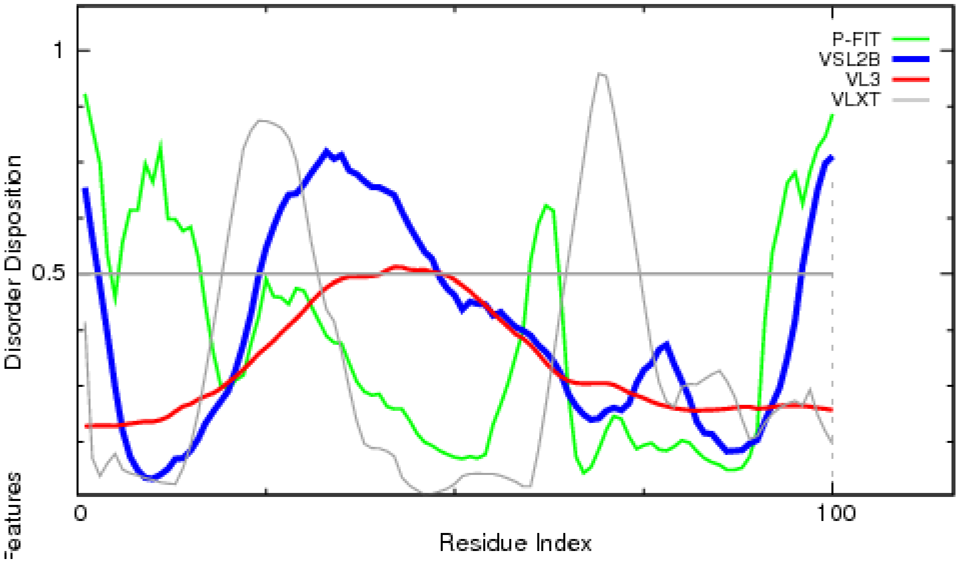

================================PONDR VSL2 STATISTICS================================

Predicted residues: 100	Number Disordered Regions: 3
Number residues disordered: 30	Longest Disordered Region: 23
Overall percent disordered: 30.00	Average Prediction Score: 0.3768
Predicted disorder segment [1]-[2]	Average Strength= 0.6372
Predicted disorder segment [25]-[47]	Average Strength= 0.6721
Predicted disorder segment [96]-[100]	Average Strength= 0.6643

ANCHOR:

Predicted Disordered Binding Regions			
	From	To	Length
None			
Filtered Regions			
	From	To	Length
1	6	12	7

ModPred and PROSITE:

ModPred: Proteolytic cleavage (D36, R76, D77), Disulphide linkage (C67, C71, C99), Amidation (Y70), Hydroxylation (P100).

PROSITE: No identified domain recognition sites.

Structural modelling:

Name	Title	Identity	Method	Oligo State	Ligands
3hwe.1.A	Neutrophil gelatinase-associated lipocalin	15.22	X-ray, 2.8Å	monomer	2 x RKS
5ool.1.2	39S ribosomal protein L36, mitochondrial	16.22	EM	hetero-oligomer	1 x PNS, 12 x MG, 3 x ZN
2yeu.2.B	DR2231	23.53	X-ray, 2.0Å	homo-dimer	2 x GD

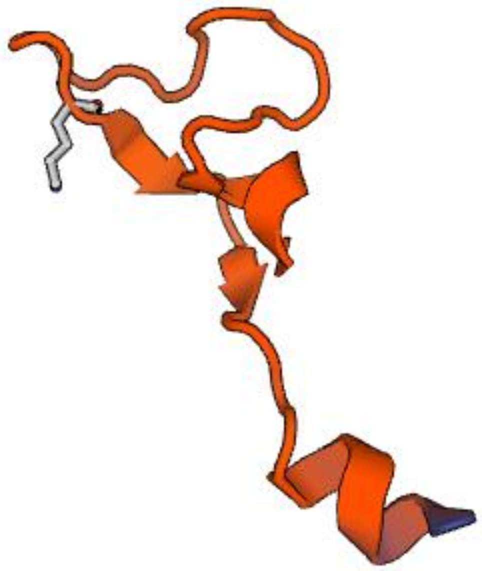

Model #1: Residues 16-56 of MVLG_04107T0 with 2yeu.2.B (23.53% sequence identity) as a template

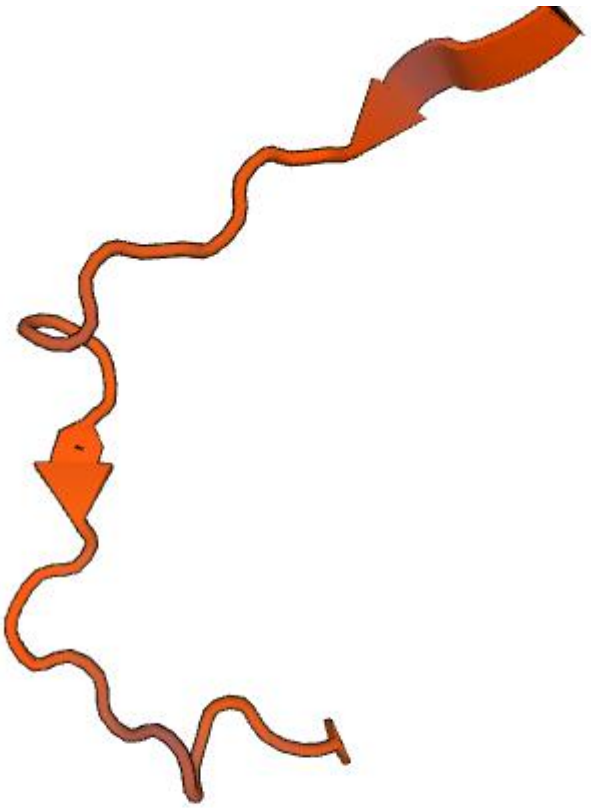

Model #2: Residues 21-46 of MVLG_04107T0 with 3hwe.1.A (15.22% sequence identity) as a template

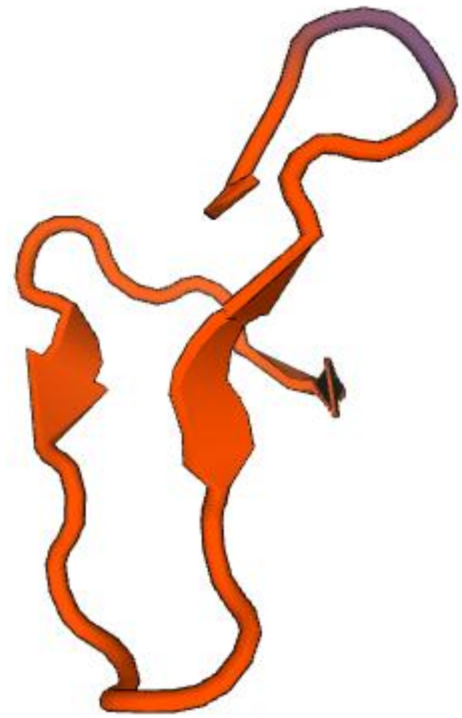

Model #3: Residues 58-80 of MVLG_04107T0 with 5ool.1.2 (16.22% sequence identity) as a template

Moderately disordered proteins (10-30% disordered residues by PONDR® VSL2 analysis)

> MVLG_02763T0 (29.30%)- 976 aa

MLVGKVSLVILWTATMALASPSRRGNHKSTTGKRSSFGSGRYLVKLSTQTDMKGSKVPVEQHRQ
DCNDQIKNLSSDFFGAIKVHNTFNFAYQCSMSVEAMEGFSPIDLADYLGVTGVDVIKVVVHGRGLS
FPMPPPVSKLQPKEGFATPWRKNHHPLLLSPSVYRNNSFAPHVESEVHLMHNLGLLGDSNVSVCLV
DTGVDYTNRRLGEGFGKGFKIVLGHDFVGNDGKHPGPSPYTNCTDHGTHVTGIVGANFDPDFKFSG
AAPEVTLGHYRAFACTGQSTEDTIAAALLRAHADGCKVITLSLGGPSAWEDGLVADAASHVTNQG
SLVVSSAGNFGTQGLFYGDVPGELPEVLGTAATDLREYPVGYLLDFVDHSFQPIPYFAVYPVKINET
LDVCYIPPSITDDPKCNLSTIVLPKGDLKNSLLVLELGQCPHSLVAKWAVANKLRVAMVSFKPEDAQ
SPLNYYSNHFARGIDYFLIVPHSWVETLIRYYTASRGKLQVSFAAGKRAPVEALANHESGGNMAFY
SSYGPTATLEGFGNTLAAPGTNILSTVTVAQGGVGVMSGTSMACPLAAGIAALLFSHRKADNLTPR
QVKSLMATTAQPVRISQKPKDAFATVVQQGAGIVSAYRAYIAKTLIEPHSIALGDLEHFKNSHSITLK
NTNKFAVTYTLSSTSSQTVTTYDKSASIDINPSGIPRPGIAGAATVAFTPRSLRIPPGQSATFTATFTLP
NFSKIDFFRVPVVSGWLLIDSAGDPVPTYRIAYAGVAAGLQIMPVLDSTDVASQSYGIKGLRHPFIM
VGNSLDPDALPSTAADVLSDPNKVTSVSRKDGIFVFLRFAMATPYVQVDLVDANTTFIPTIPSNNNL
HLAENNSLTGDLRKRPHNPPLFDSVSIVGTAATANELTRDPTDFSHGSGADTSFINFNGMVAVKHPD
DPATTSVDKGRPYRLLIRARRMNSNPEFSASYDSWLSPPFQFLD

PONDR:

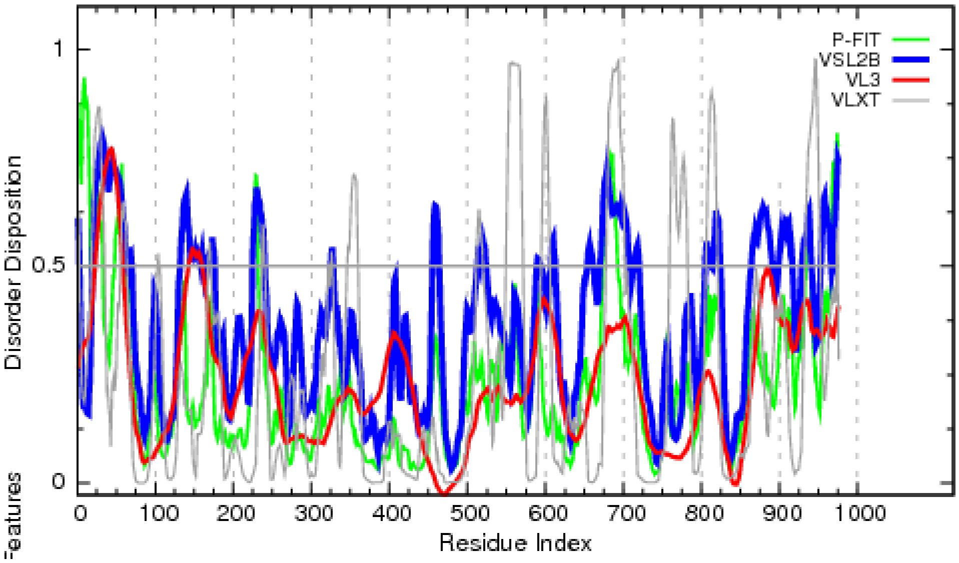

================================PONDR VSL2 STATISTICS================================

Predicted residues: 976	Number Disordered Regions: 23
Number residues disordered: 286	Longest Disordered Region: 44
Overall percent disordered: 29.30	Average Prediction Score: 0.3690
Predicted disorder segment [1]-[2]	Average Strength= 0.5861
Predicted disorder segment [20]-[63]	Average Strength= 0.6953
Predicted disorder segment [66]-[69]	Average Strength= 0.5239
Predicted disorder segment [133]-[162]	Average Strength= 0.5575
Predicted disorder segment [166]-[170]	Average Strength= 0.5125
Predicted disorder segment [172]-[176]	Average Strength= 0.5355
Predicted disorder segment [225]-[240]	Average Strength= 0.6081

Predicted disorder segment [326]-[328]	Average Strength= 0.5265
Predicted disorder segment [456]-[464]	Average Strength= 0.5943
Predicted disorder segment [513]-[523]	Average Strength= 0.5317
Predicted disorder segment [589]-[591]	Average Strength= 0.5126
Predicted disorder segment [608]-[612]	Average Strength= 0.5391
Predicted disorder segment [654]-[660]	Average Strength= 0.5248
Predicted disorder segment [670]-[707]	Average Strength= 0.6281
Predicted disorder segment [714]-[720]	Average Strength= 0.5272
Predicted disorder segment [805]-[812]	Average Strength= 0.5269
Predicted disorder segment [815]-[824]	Average Strength= 0.5722
Predicted disorder segment [865]-[891]	Average Strength= 0.5827
Predicted disorder segment [899]-[916]	Average Strength= 0.5697
Predicted disorder segment [929]-[941]	Average Strength= 0.5631
Predicted disorder segment [954]-[966]	Average Strength= 0.6000
Predicted disorder segment [968]-[968]	Average Strength= 0.5005
Predicted disorder segment [970]-[976]	Average Strength= 0.6613

ANCHOR:

Predicted Disordered Binding Regions			
	From	To	Length
1	889	894	6
2	918	926	9
**Filtered Regions**			
	From	To	Length
1	1	16	16
2	42	43	2
3	216	219	4
4	832	839	8
5	946	949	4
6	974	976	3

ModPred and PROSITE:

ModPred: Amidation (A19, F131, N392, F652, F709, R953), Proteolytic cleavage (R34, R41, D110, D222, Y527, R715, D781, K828, D888, D965), Acetylation (K213), Ubiquitination (K229, K456), Sumoylation (K259), O-linked glycosylation (S694), ADP-ribosylation (R698)

PROSITE: Subtilase_ASP (193-204, PROSITE entry PS00136), Subtilase_HIS (242-252, PROSITE Entry PS00137), Subtilase_SER (566-576, PROSITE PS00138)

Structural modelling:

	Name	Title	Identity	Method	Oligo State	Ligands
	3eif.1.A	C5a peptidase	22.10	X-ray, 1.9Å	monomer	1 x CA, 1 x MLA
	4i0w.1.B	Protease CspB	20.42	X-ray, 1.6Å	hetero-oligomer	None
	3lxu.1.A	Tripeptidyl-peptidase 2	18.67	X-ray, 3.1Å	homo-dimer	None
	1r6v.1.A	subtilisin-like serine protease	22.94	X-ray, 1.7Å	monomer	1 x CA
	1y9z.1.A	alkaline serine protease	20.58	X-ray, 1.4Å	monomer	2 x CA, 1 x PMS
	4mzd.1.A	Nisin leader peptide-processing serine protease NisP	21.30	X-ray, 1.1Å	monomer	None

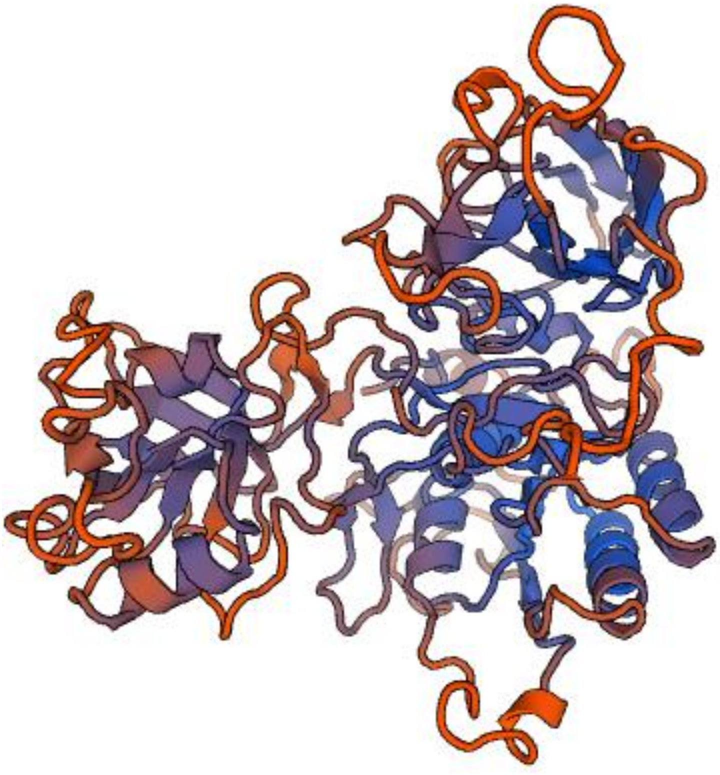

Model #1: Residues 172-780 of MVLG_02763T0 with 3eif.1.A (22.10% sequence identity) as a template

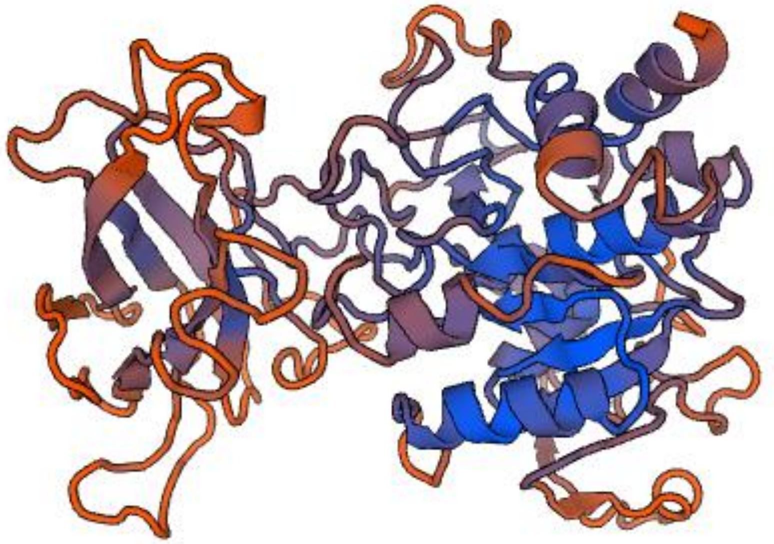

Model #2: Residues 172-639 of MVLG_02763T0 with 4i0w.1.B (20.42% sequence identity) as a template

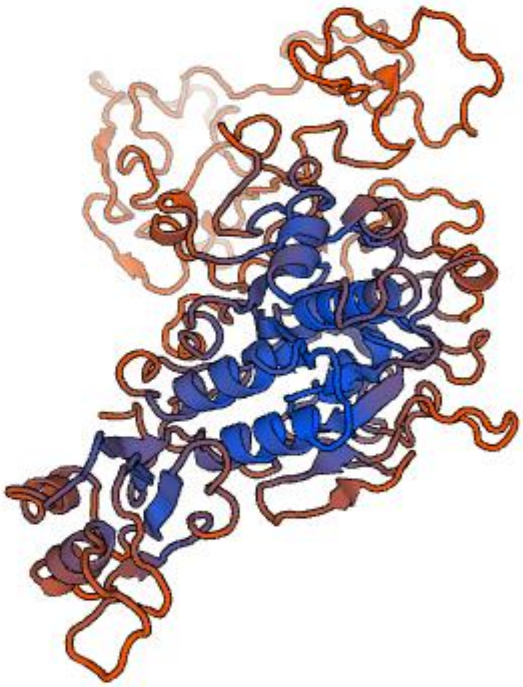

Model #3: Residues 34-637 of MVLG_02763T0 with 1r6v.1.A (22.94% sequence identity) as a template

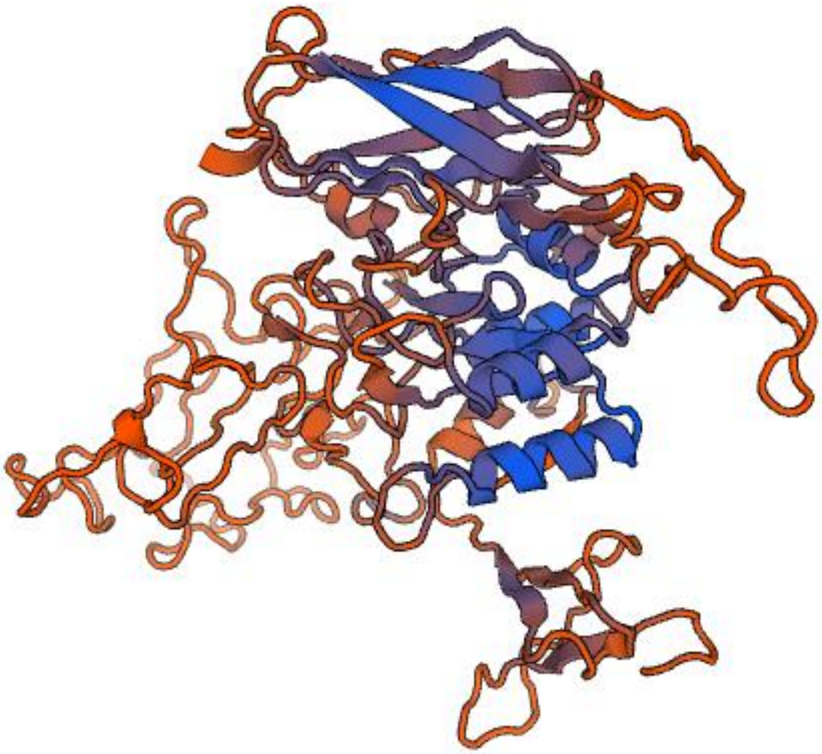

Model #4: Residues 185-770 of MVLG_02763T0 with 3lxu.1.A (18.67% sequence identity) as a template

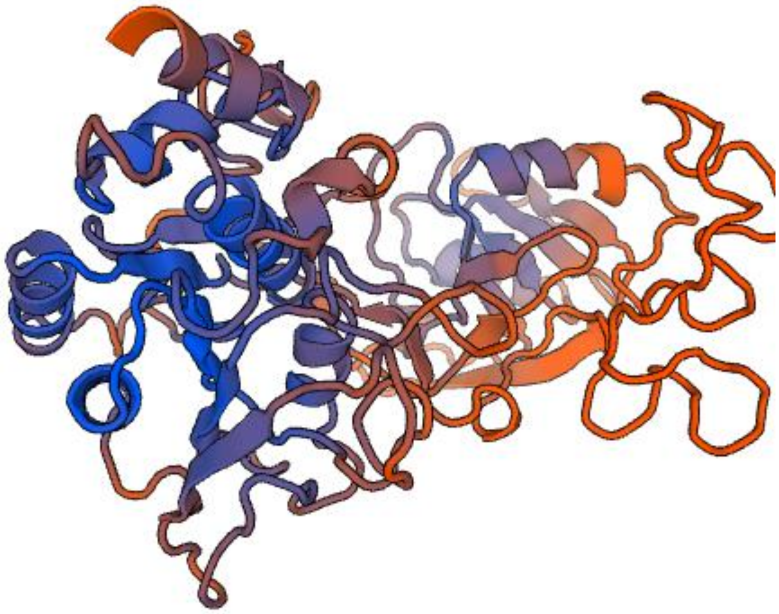

Model #5: Residues 177-639 of MVLG_02763T0 with 1y9z.1.A (20.58% sequence identity) as a template

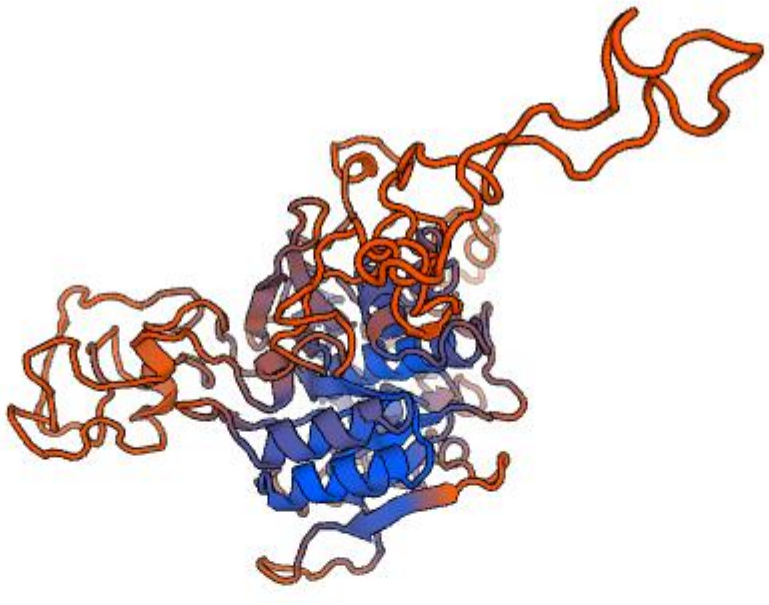

Model #6: Residues 144-638 of MVLG_02763T0 with 4mzd.1.A (21.30% sequence identity) as a template

> MVLG_03994T0 (28.32%)- 618 aa

MRKAFSFFALLYATSWARAQVLRPHPLHDSISSSDQIHPRGLEDYFLDKKRLSAALDLLDEEHIMW
SGDETPAGDFPVATMHFETHSKEKVLNMQRFSRLISGVSCGVGRIYITFKSRVAFDYAAQAWDWVH
LHPEHVFTLLAHWRDCMNPDGHFIPFHFKKAVPEASTLTITLEGIEVAWEEAGHTFTLHVGSGLREG
EELQNTATKELEVPSHFTEVAHPLPAPNAGPLLEERFHIGHLNFKLPDPSVSRSKGYSVHLDHKYNG
EMVSKHHLGQNGYEATSHCINCGSSGRIDISFRLRIKWFDIKEMGIYATAFNVGARLQWDLSLKANT
IASLDFGGNIFEFPLPGLGLEIHKIFKLGLIASVGWGIGCRNYTGHLEMSHGIQFRIQDGAEAHIDLVK
GIGGNGHWRPQVWSAPLHIEGKVKANPAASAGSTVGFEMELFKTTLAAGLRISAPSAVFLVKLNDA
NKGPCGQRLHRRSIQVDAILLAYLGMSGGLNGAFAGSEPIGKRGLIDGGVHTSKNNQTRRERLLDM
SWMEPNFTEEEFREFLESNDVDVSAHAAHLNSSKHSLHPRRLGLFANLPIYHHSWPLIKELCIPIGPH
VPLQKRSHPRDLLIGP

PONDR:

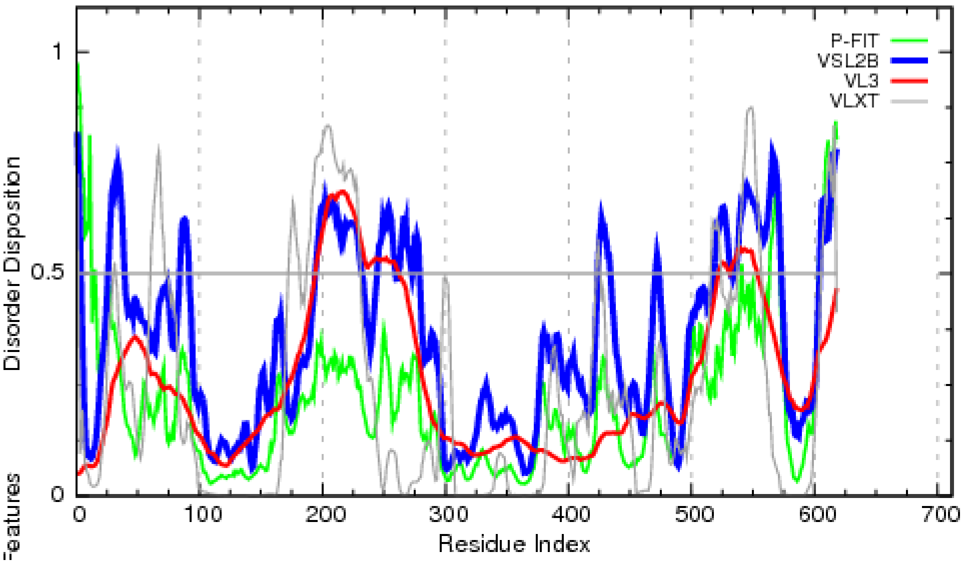

================================PONDR VSL2 STATISTICS================================

Predicted residues: 618
Number residues disordered: 175
Overall percent disordered: 28.32
Number Disordered Regions: 12
Longest Disordered Region: 38
Average Prediction Score: 0.3560

Predicted disorder segment [1]-[3]	Average Strength= 0.7133
Predicted disorder segment [26]-[39]	Average Strength= 0.6514
Predicted disorder segment [85]-[91]	Average Strength= 0.5943
Predicted disorder segment [195]-[232]	Average Strength= 0.6078
Predicted disorder segment [245]-[259]	Average Strength= 0.5904
Predicted disorder segment [262]-[272]	Average Strength= 0.5630
Predicted disorder segment [274]-[279]	Average Strength= 0.5489
Predicted disorder segment [424]-[434]	Average Strength= 0.5695
Predicted disorder segment [471]-[473]	Average Strength= 0.5258
Predicted disorder segment [519]-[533]	Average Strength= 0.5848
Predicted disorder segment [536]-[573]	Average Strength= 0.6425
Predicted disorder segment [605]-[618]	Average Strength= 0.6718

ANCHOR:

Predicted Disordered Binding Regions			
	From	To	Length
None			

Filtered Regions			
	From	To	Length
1	236	240	5
2	486	492	7
3	579	579	1

ModPred and PROSITE:

ModPred: Sumoylation (K3), Amidation (A4, L353, Y376, N407, Y491, Y585), Proteolytic cleavage (D29, R530, R608), Ubiquitination (K263, K271, K426, K568).

PROSITE: No identified domain recognition sites.

Structural modelling:

Name	Title	Identity	Method	Oligo State	Ligands
1p1g.1.A	PROTEIN (MACROPHAGE MIGRATION INHIBITORY FACTOR)	10.29	X-ray, 2.5Å	homo-trimer	None
3iq2.1.A	Sorting nexin-7	28.00	X-ray, 1.7Å	monomer	1 x GOL, 6 x SO4

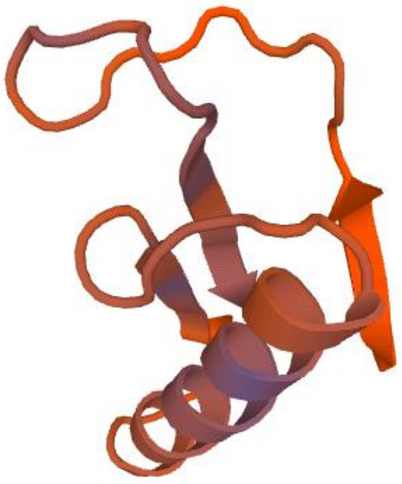

Model #1: Residues 53-121 of MVLG_03994T0 with 1p1g.1.A (10.29% sequence identity) as a template

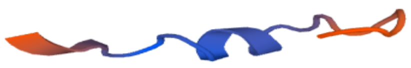

Model #2: Residues 536-556 of MVLG_03994T0 with 3iq2.1.A (28.00% sequence identity) as a template

>MVLG_01159T0 (27.49%)- 502 aa

MHGNYLRLLLGSILICQVIAKWYKYDTGDHRIPRRHRGVDLRGRHHASGVLGGDFSSNIETANQT
IQIDELRQTTDTKGENEDLQVSDQEGNGSSFDESDKASNGRIIISGPPRYVNPAVREKYLRIDMRSKG
FTLDEEPFRVVGINIYWLCNDENVLGVKPGTPTQKRRIREALAAAVAMGANTVRVGSCGISLGYAD
ALQPDQHHRAAPRSPAMDIHDYAIYAAGRYGLKIILPLMDNYDYYHGGKYTVLKWLGISAEHNGA
NFFTDPRAIAFFKSYIEFVLNRKNPYTMRTYGEDPVVSIIEDGNEFGAYKGSEGYPPLAFTDEIAAQV
KKFAPQALFMDGTDGFFNLTAHLQAPGLRSKAVDIVTDHPYPRDIPLLQMQAFLARISGKAFILGEM
DWVPSAPSRNPPSRLVEPSLSAYLNVLDRYPNIGVLAWSLFVHTDDCRDWVRHHDGYEMYYPLPQ
DTAEKQANVLTLVQWFYARTGREVPSLLPYQTCPQEEF

PONDR:

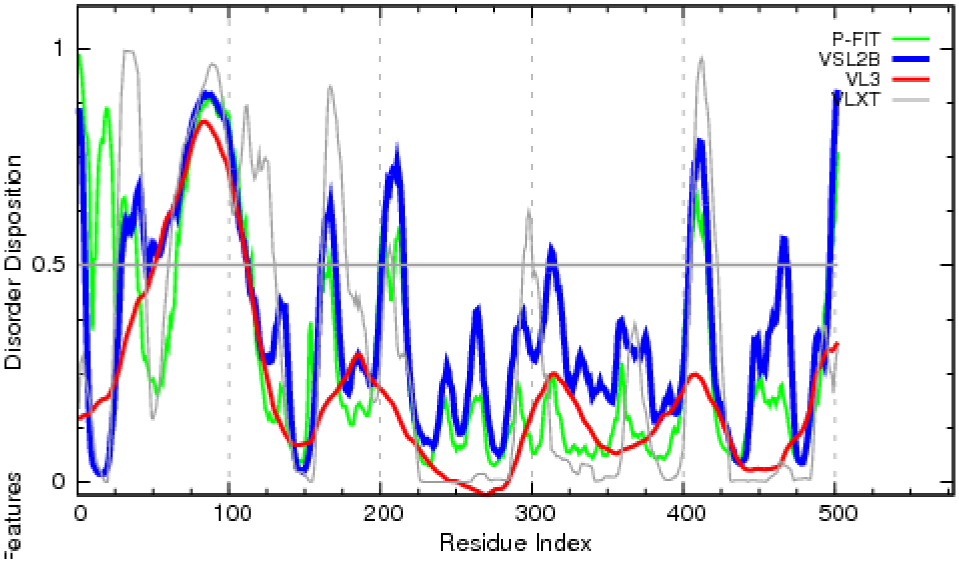

===============================PONDR VSL2 STATISTICS================================

Predicted residues: 502	Number Disordered Regions: 9
Number residues disordered: 138	Longest Disordered Region: 65
Overall percent disordered: 27.49	Average Prediction Score: 0.3579
Predicted disorder segment [1]-[5]	Average Strength= 0.7244
Predicted disorder segment [29]-[45]	Average Strength= 0.5999
Predicted disorder segment [48]-[112]	Average Strength= 0.7189
Predicted disorder segment [161]-[170]	Average Strength= 0.5798
Predicted disorder segment [201]-[215]	Average Strength= 0.6589
Predicted disorder segment [312]-[314]	Average Strength= 0.5251
Predicted disorder segment [404]-[416]	Average Strength= 0.6889
Predicted disorder segment [465]-[468]	Average Strength= 0.5394
Predicted disorder segment [497]-[502]	Average Strength= 0.7601

ANCHOR:

Predicted Disordered Binding Regions			
	From	To	Length
None			

Filtered Regions			
	From	To	Length
1	9	23	15
2	55	55	1
3	107	111	5
4	144	153	10

ModPred and PROSITE:

ModPred: Amidation (A8, V55, L723, Y752, D770, D955, Y1022), Proteolytic cleavage (R62, K160, R283, R286, D322, K326, Y329, S330, D407, R562, R565, D568, T897, P931), Carboxylation (E99, E106, E107, E109, E113, E117, E118, E121, E122), O-linked glycosylation (T169, T170, T171T178, T180, T182, T186, S190, T192, T196, T197, T198, T199, T200, T201, T202, T203, T204, T205, T206), Hydroxylation (P189), Nlinked glycosylation (N397), Phosphorylation (T692), Disulphide linkage (C1025).

PROSITE: No identified domain recognition sites.

Structural modelling:

	Name	Title	Identity	Method	Oligo State	Ligands
☐	1uuq.1.A	MANNOSYL-OLIGOSACCHARIDE GLUCOSIDASE	16.91	X-ray, 1.5Å	monomer	None
☐	4xzw.1.A	endo-glucanase chimera C10	16.92	X-ray, 1.5Å	monomer	2 x O4B, 1 x CA

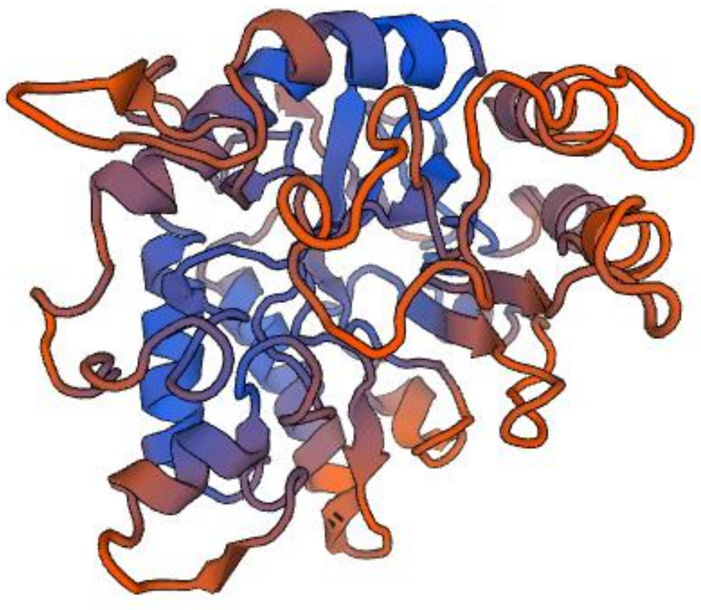

Model #1: Residues 122-485 of MVLG_01159T0 with 1uuq.1.A (16.91% sequence identity) as a template

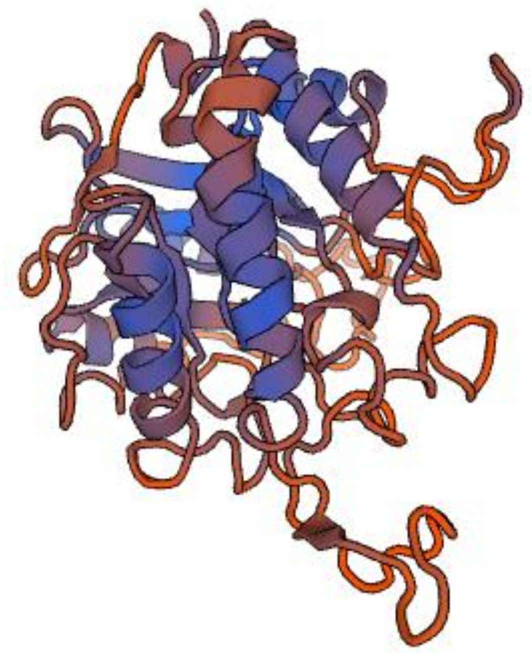

Model #2: Residues 116-445 of MVLG_01159T0 with 4xzw.1.A (16.92% sequence identity) as a template

>MVLG_07010T0 (27.04%)- 159 aa

MLFKLPVVLAMALLTLGASASERFTVTSLRRRDKPGDYPQDRGSVKSPAEGQQLKVGTLFPFRFN
PISVGDLVDTLDVEVFLKIKSLNYSRRLVTNLMSPGGNKPIVQNFIVMHPKGSIVKRGTIMPGTIEVFE
QQNGTKANGNGKYFLNQAVGVTFQF

PONDR:

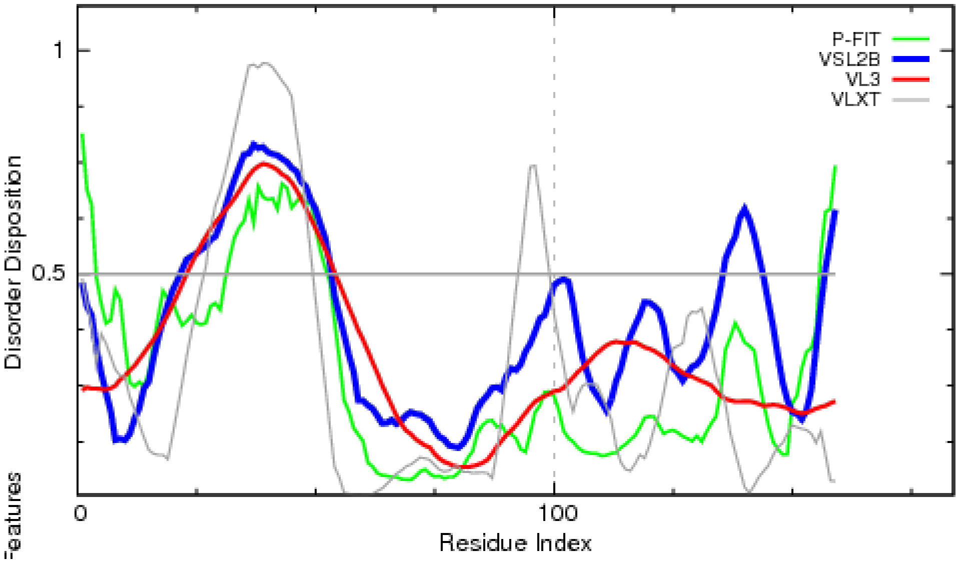

===============================PONDR VSL2 STATISTICS===============================

Predicted residues: 159	Number Disordered Regions: 3
Number residues disordered: 43	Longest Disordered Region: 32
Overall percent disordered: 27.04	Average Prediction Score: 0.3847
Predicted disorder segment [22]-[53]	Average Strength= 0.6694
Predicted disorder segment [136]-[143]	Average Strength= 0.5874
Predicted disorder segment [157]-[159]	Average Strength= 0.5780

ANCHOR:

Predicted Disordered Binding Regions			
	From	To	Length
None			

ModPred and PROSITE:

ModPred: Proteolytic cleavage (R23, T27, S28, R30, R32, R42, K46), ADP-ribosylation (R122), Acetylation (K146), Amidation (Y147).

PROSITE: No identified domain recognition sites.

Structural modelling:

Name	Title	Identity	Method	Oligo State	Ligands
5i4q.1.A	Contact-dependent inhibitor A	22.45	X-ray, 2.3Å	hetero-oligomer	2 x SO4, 2 x CL

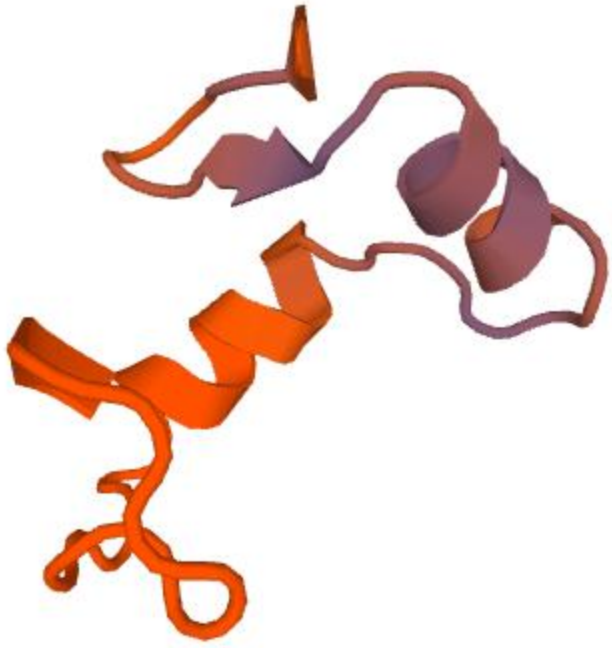

Model #1: Residues 81-129 of MVLG_07010T0 with 5i4q.1.A (22.45% sequence identity) as a template

> MVLG_01192T0 (23.84%)- 495 aa

MLRSYLLLVSILVHDAAASWYEAASRLDDTVQPWNRSCPRRVRAQAELLRGVWRDSNGDRISFD
LRKRNLEQDFDFVEPAPDFLGLPPLGTAVSSARSARNFAVRPRATGGQGTDYLGTAPINSSASAVDT
IQPEASIIIDGPPASFQGSGSYLEVDPSGTGLTLDGEPFRPVGPRLCNDESLSCLPRGYYTDKSRIREAL
AAAVAMGANTIRINSCGISTGFPQAVQPSLHTYGTDEQLDIHDYVIYAAGEYGLKVILPLTDNYDYY
HGGKYTFLRWLNVPTDNAGAQFFTDRQVRRAFKRYIKFLLTRVNQYNGLAYGEDPTIAIIEDGNEF
GAYMGKEGFPPLSFTEDIAKYVKSLAPQALLMDGTDGFYNYTTKAVAPGVTSPYVDIVTDHAYPRN
IALLKRQVDIAHSNGKVFLIGEMDWTPNNGGADFGAYLNLLYNYKSVGVMAWSLFTHDTPCSSYV
IHDDAYSIYYPNGGQHLTLILLQSSGAEGF

PONDR:

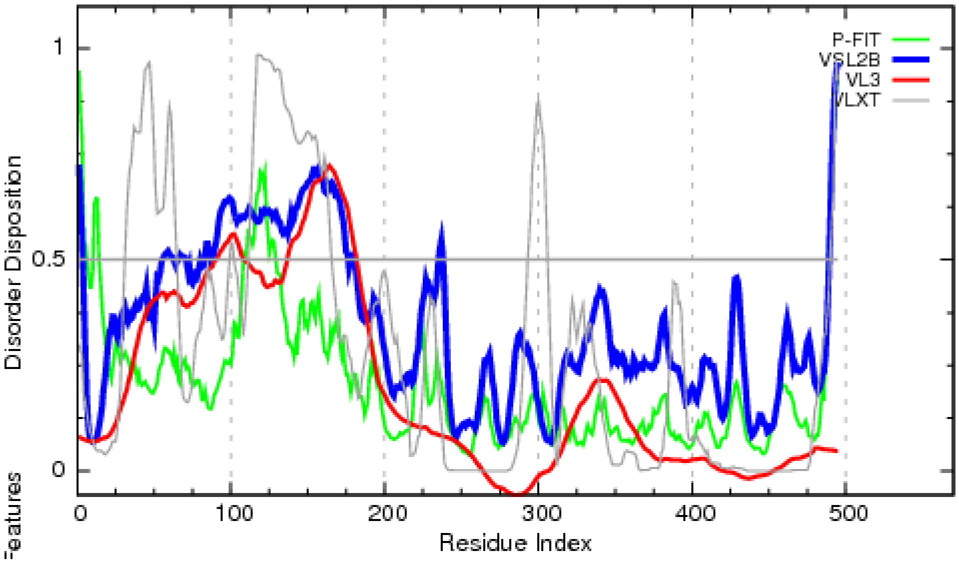

================================PONDR VSL2 STATISTICS================================

Predicted residues: 495	Number Disordered Regions: 8
Number residues disordered: 118	Longest Disordered Region: 88
Overall percent disordered: 23.84	Average Prediction Score: 0.3486
Predicted disorder segment [1]-[3]	Average Strength= 0.6444
Predicted disorder segment [56]-[62]	Average Strength= 0.5107
Predicted disorder segment [66]-[70]	Average Strength= 0.5060
Predicted disorder segment [83]-[86]	Average Strength= 0.5228
Predicted disorder segment [89]-[176]	Average Strength= 0.6229
Predicted disorder segment [179]-[180]	Average Strength= 0.5117
Predicted disorder segment [236]-[238]	Average Strength= 0.5279
Predicted disorder segment [490]-[495]	Average Strength= 0.8299

ANCHOR:

Predicted Disordered Binding Regions			
	From	To	Length
None			

Filtered Regions			
	From	To	Length
1	136	140	5

ModPred and PROSITE:

ModPred: Amidation (A16, M450, Y471, Y475, Q488), Pyrrolidone carboxylic acid (Q45), Proteolytic cleavage (R97, W452), Methylation (K256, K301).

PROSITE: No identified domain recognition sites.

Structural modelling:

	Name	Title	Identity	Method	Oligo State	Ligands
	1rh9.1.A	endo-beta-mannanase	24.92	X-ray, 1.5Å	monomer	None
	2zun.1.A	458aa long hypothetical endo-1,4-beta-glucanase	17.99	X-ray, 2.0Å	monomer	2 x CBI
	4cu6.1.A	BETA-GALACTOSIDASE	19.29	X-ray, 2.7Å	monomer	None

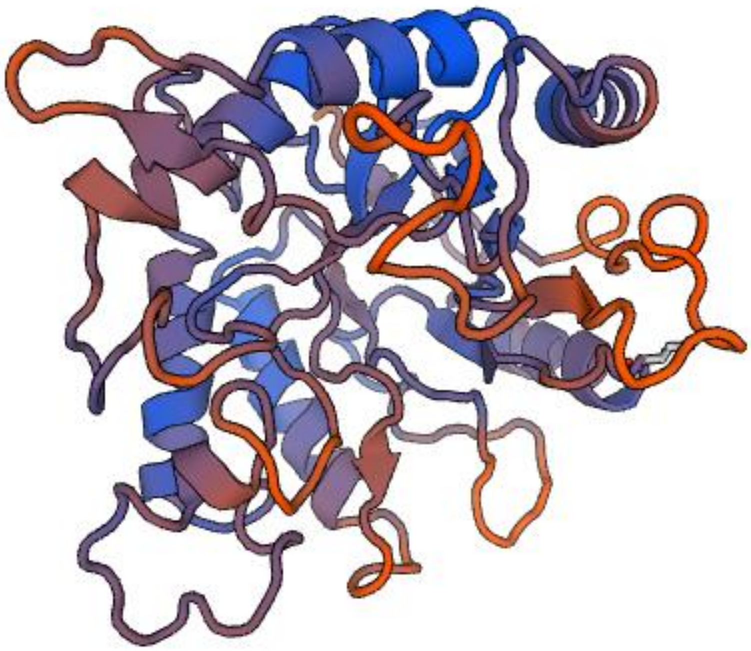

Model #1: Residues 153-491 of MVLG_01192T0 with 1rh9.1.A (24.92% sequence identity) as a template

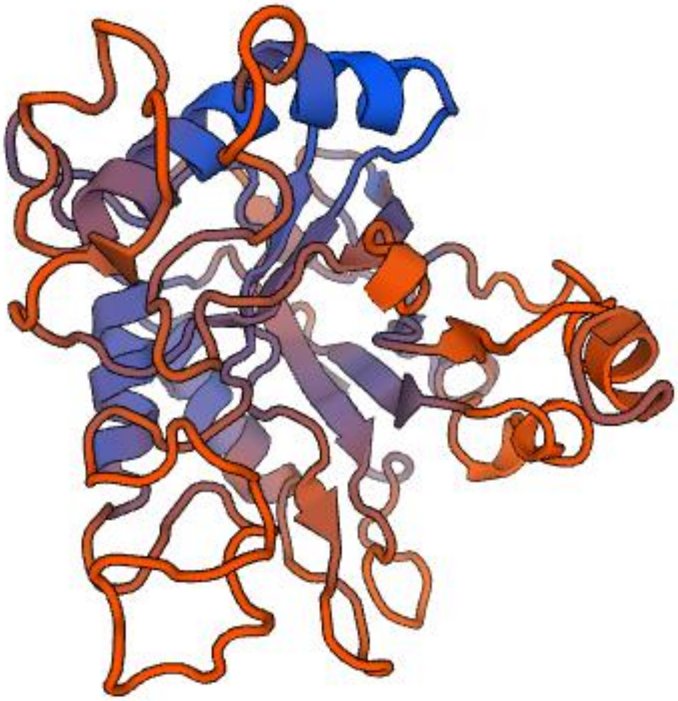

Model #2: Residues 151-457 of MVLG_01192T0 with 2zun.1.A (17.99% sequence identity) as a template

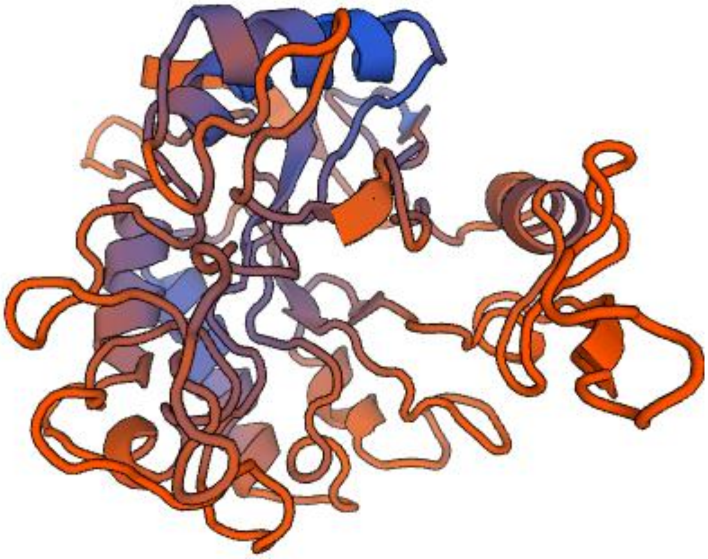

Model #3: Residues 152-457 of MVLG_01192T0 with 4cu6.1.A (19.29% sequence identity) as a template

> MVLG_05108T0 (23.69%)- 363 aa

MLCKLSLVLILASSFWVALATPPAACTIVSSTDIPKVQKCKVITIMAFIMPAGQTLMLDVQAGTTIN
QLGDIIFEHRGPWRGPLMSIYGDSITYNGNNKKLYCNGQMYWDGMGVTGTTKPGPALSLLITGTVS
DLIIHNSPLNAVVVEANGKTLLSNIFVNNTDGDRMGGHNTDGFNVVQKTRDLTISGCTVINQDDCISI
TSGQGITISQNTCKNGHGISIGSIKSNEHVSQVTISQNHVENSQQGYRIKTYSGATRGSVDNITFHGNT
GNGLTHYGVVVEQDYTESGPKGPSFATNGVLISNIRFVGPITTLSMAGDKAQKVYVLCGVNSCIGD
WDWSSLKFTRGGSLGSITRAPIRGLTA

PONDR:

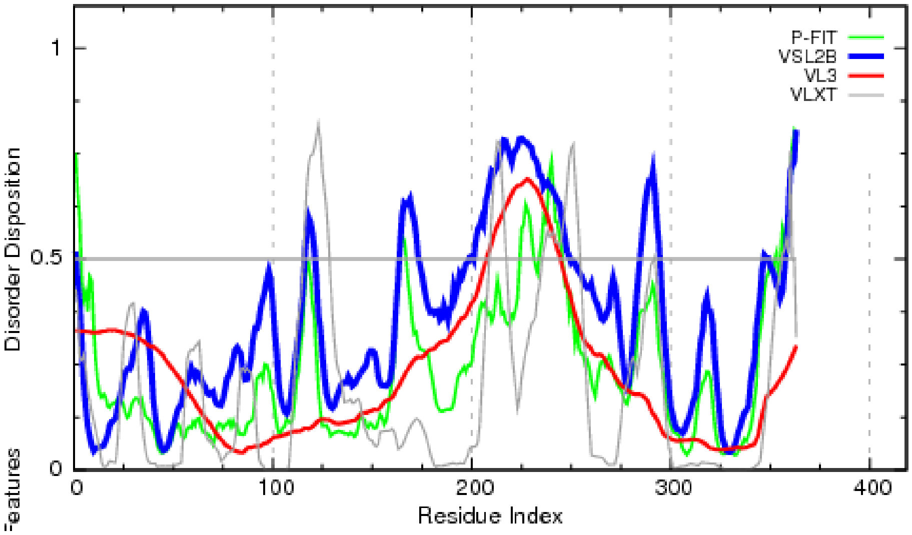

==============================PONDR VSL2 STATISTICS===============================

Predicted residues: 363	Number Disordered Regions: 7
Number residues disordered: 86	Longest Disordered Region: 48
Overall percent disordered: 23.69	Average Prediction Score: 0.3541
Predicted disorder segment [116]-[121]	Average Strength= 0.5558
Predicted disorder segment [164]-[173]	Average Strength= 0.5941
Predicted disorder segment [199]-[200]	Average Strength= 0.5054
Predicted disorder segment [202]-[249]	Average Strength= 0.6874
Predicted disorder segment [285]-[294]	Average Strength= 0.6248
Predicted disorder segment [347]-[349]	Average Strength= 0.5044
Predicted disorder segment [358]-[363]	Average Strength= 0.6849

ANCHOR:

Predicted Disordered Binding Regions			
	From	To	Length
None			

Filtered Regions			
	From	To	Length
1	327	329	3

ModPred and PROSITE:

ModPred: Disulphide linkage (C103), Amidation (Y248, Y325).
PROSITE: No identified domain recognition sites.

Structural modelling:

	Name	Title	Identity	Method	Oligo State	Ligands
☐	1czf.1.A	POLYGALACTURONASE II	40.48	X-ray, 1.7Å	monomer	3 x ZN, 1 x NAG
☐	1k5c.1.A	ENDOPOLYGALACTURONASE	41.72	X-ray, 1.0Å	monomer	2 x NAG
☐	3lmw.1.A	Iota-carrageenase, CgiA	10.14	X-ray, 2.6Å	monomer	1 x NI, 1 x CA
☐	4xqi.1.A	Tail spike protein	13.82	X-ray, 1.8Å	homo-trimer	3 x GLC, 3 x GLA, 3 x RAM, 3 x NAG, 3 x NDG
☐	5gai.1.Y	Tail fiber protein	17.27	EM	hetero-oligomer	None
☐	4xor.1.A	Tail spike protein	12.07	X-ray, 1.5Å	homo-trimer	3 x GLC, 3 x GLA, 3 x RAM, 6 x NAG, 3 x NDG

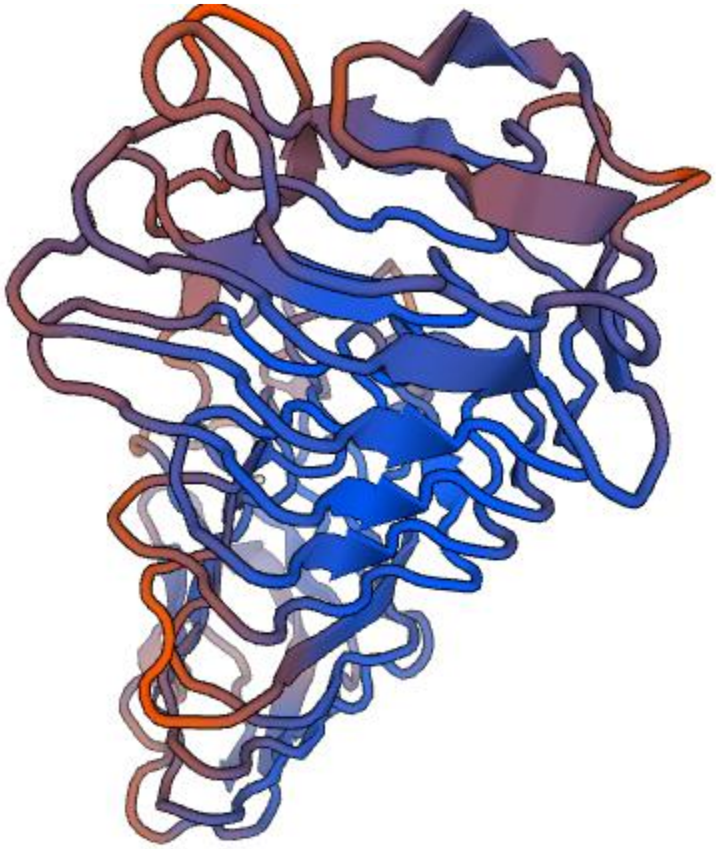

Model #1: Residues 26-360 of MVLG_05108T0 with 1k5c.1.A (41.72% sequence identity) as a template

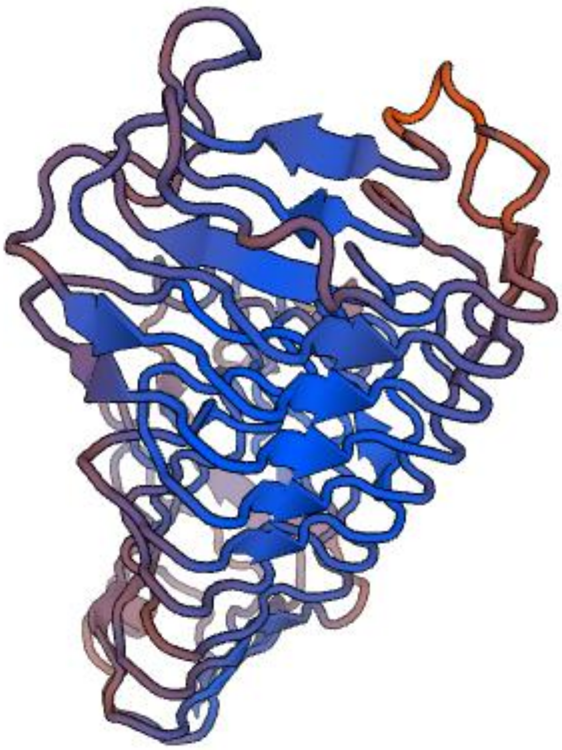

Model #2: Residues 26-347 of MVLG_05108T0 with [1czf.1.A](1czf.1.A) (40.48% sequence identity) as a template

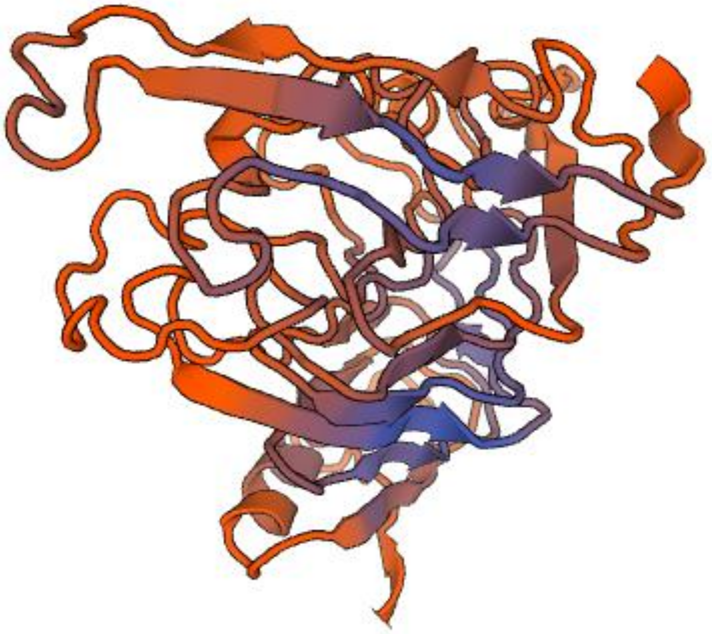

Model #3: Residues 13-314 of MVLG_05108T0 with 4xqi.1.A (13.82% sequence identity) as a template

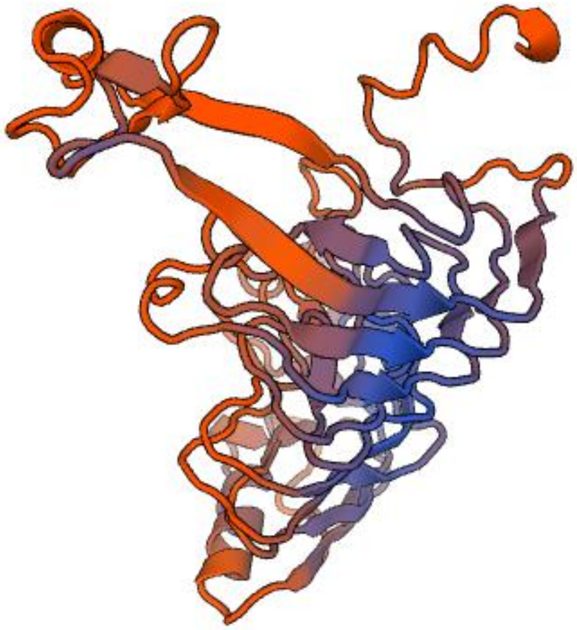

Model #4: Residues 14-330 of MVLG_05108T0 with 3lmw.1.A (10.14% sequence identity) as a template

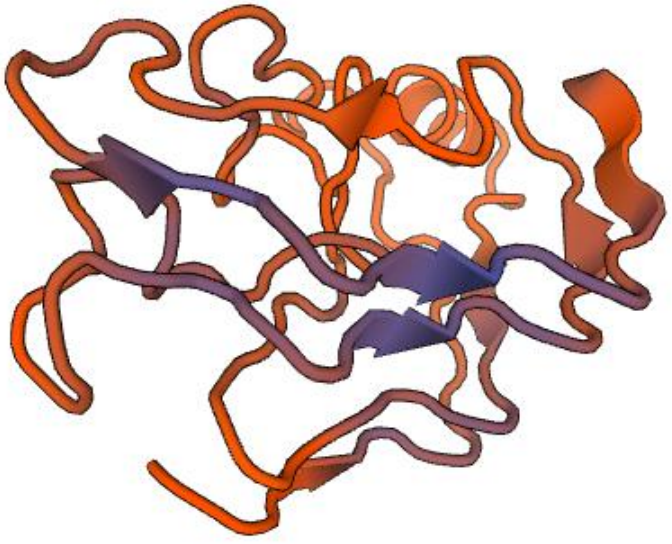

Model #5: Residues 142-274 of MVLG_05108T0 with 4xor.1.A (12.07% sequence identity) as a template

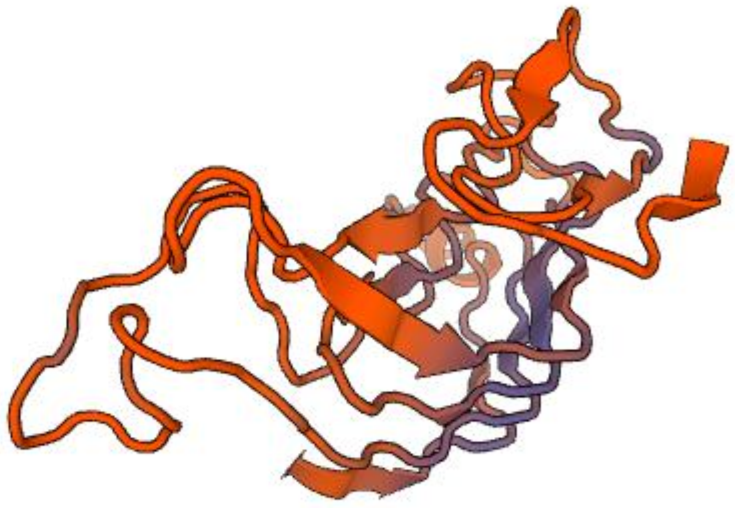

Model #6: Residues 151-313 of MVLG_05108T0 with 5gai.1.Y (17.27% sequence identity) as a template

> MVLG_04797T0 (23.60%)- 428 aa

MPKAAIFNVATVRVLVTFVTLLVFVSPLAAADAVDHPKLRVKGGLGRAGAPAEVIASAAASVTG
SNSKSYPRGYSAVIAFGASYMDNAHKRSKKYATSFRDQQEYPFSDRGRYTNGPVAVEYMVKPSTN
PALRPFQIDPPVLFDFAYGGSVIKNNLTGTAGPHNIPDLGREIKQYLEQLDDEIIDPGRGRVLHVIHTG
TNPISQMWLHALTANITHAKTRRSIGKQVTQMAKYIRYLATHDSLRDNVVAADYLIVGLPPLGIVPN
LYFNYIAAFPNHTAAQRDAALEYAGELVDLFNVELEAFTSSLKAYVKPGSRILYYDLANLFKTIYRF
PRIYGITAPVTQACWSSSTRVLCKDPEHHLYIDTLHPTTSAHKIWASRMNRLVNKVARQADAKTLE
TTVEDDPTTDDHPSSKTDDEPSSSPGDLRC

PONDR:

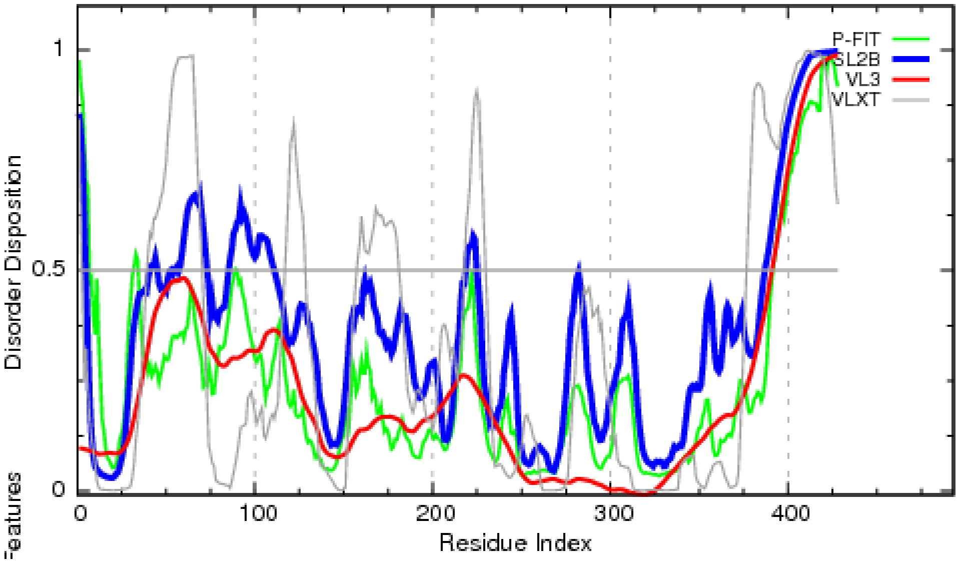

===============================PONDR VSL2 STATISTICS===============================

Predicted residues: 428	Number Disordered Regions: 8
Number residues disordered: 101	Longest Disordered Region: 42
Overall percent disordered: 23.60	Average Prediction Score: 0.3703
Predicted disorder segment [1]-[4]	Average Strength= 0.7574
Predicted disorder segment [41]-[45]	Average Strength= 0.5111
Predicted disorder segment [53]-[53]	Average Strength= 0.5008
Predicted disorder segment [56]-[56]	Average Strength= 0.5064
Predicted disorder segment [58]-[73]	Average Strength= 0.6098
Predicted disorder segment [86]-[110]	Average Strength= 0.5725
Predicted disorder segment [219]-[225]	Average Strength= 0.5442
Predicted disorder segment [387]-[428]	Average Strength= 0.8670

ANCHOR:

Predicted Disordered Binding Regions			
	From	To	Length
1	140	150	11
2	383	389	7

Filtered Regions			
	From	To	Length
1	424	428	5

ModPred and PROSITE:

ModPred: Ubiquitination (K3), Amidation (A4, S26, A373, A389, K414), Methylation (K68), Proteolytic cleavage (R100), Phosphorylation (S422).

PROSITE: No identified domain recognition sites.

Structural modelling:

	Name	Title	Identity	Method	Oligo State	Ligands
☐	3kvn.1.A	Esterase estA	21.58	X-ray, 2.5Å	monomer	None
☐	1zmb.1.A	Acetylxylan esterase related enzyme	9.52	X-ray, 2.6Å	homo-dimer	None

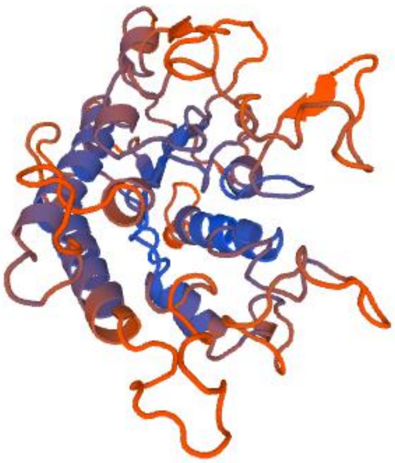

Model #1: Residues 71-399 of MVLG_04797T0 with 3kvn.1.A (21.58% sequence identity) as a template

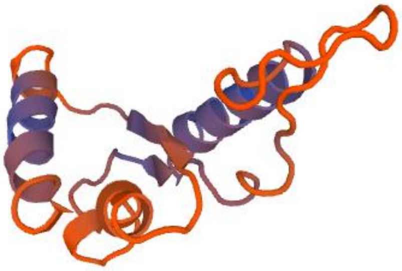

Model #2: Residues 293-389 of MVLG_04797T0 with 1zmb.1.A (9.52% sequence identity) as a template

> MVLG_03707T0 (22.86%)- 433 aa
MLSHSTSRRFLRSWGAFLCLPWNPLALVLAATIPTSHSLLTNSGLDSRTLGKVRERLDLVARDVW
VSGTQTEAYLELDQPQLTVFNPYVFNPFTPKSSRDRVILESSFPNSSNRIVLNWLERLGPDDEQFAVI
KGGAAGDPASLGYAWMIAQATTTDEGTQERLERMIEAEVEWLLEKVPRTMDGAISHRKEVTQLWS
DFIYMVPPFLAARGIATSNHSLLLESYRQIKLYRSHLQDTSTHLWRHVRYGTWEDPSLWATGNAWA
AAGITRVLATLTNSFHTAMYWEEIRDLALWANEIVEAGFARVKKDGLLPNHLDDPYDFSDSASSAL
LASTVFRLHQLGMIRKPSTTLAKAEKIRSKINDKIDPKTGWLRGCVNPLSWYQRTDQSPEAQAFVIL
LEAAWRDSRMISERNSIASKEKVFGGQGTHTRRRDR

PONDR:

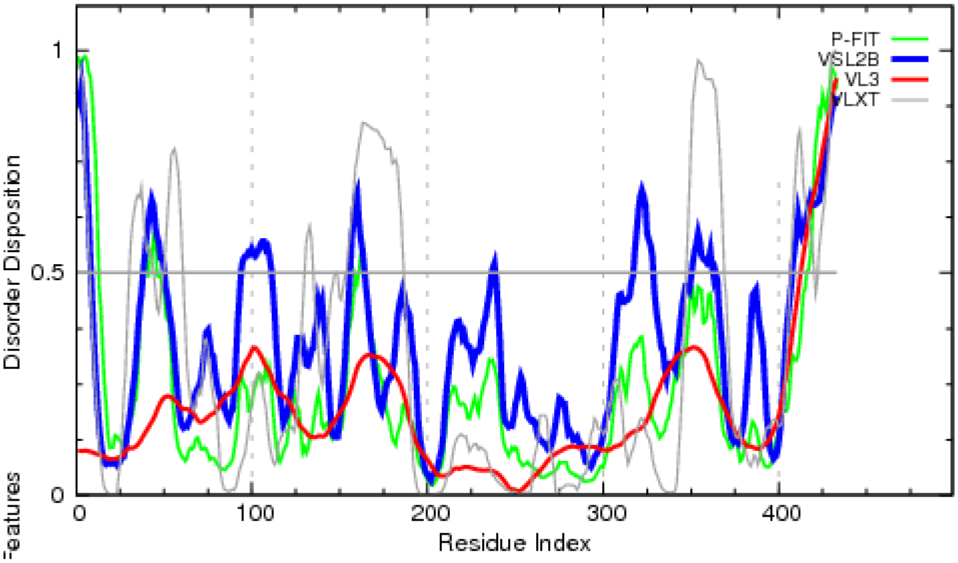

==============================PONDR VSL2 STATISTICS===============================

Predicted residues: 433	Number Disordered Regions: 9
Number residues disordered: 99	Longest Disordered Region: 26
Overall percent disordered: 22.86	Average Prediction Score: 0.3405
Predicted disorder segment [1]-[8]	Average Strength= 0.7855
Predicted disorder segment [39]-[50]	Average Strength= 0.5846
Predicted disorder segment [94]-[111]	Average Strength= 0.5440
Predicted disorder segment [156]-[164]	Average Strength= 0.5907
Predicted disorder segment [237]-[238]	Average Strength= 0.5152
Predicted disorder segment [318]-[328]	Average Strength= 0.6108
Predicted disorder segment [351]-[362]	Average Strength= 0.5418
Predicted disorder segment [364]-[364]	Average Strength= 0.5008
Predicted disorder segment [408]-[433]	Average Strength= 0.6937

ANCHOR:

Predicted Disordered Binding Regions			
	From	To	Length
1	393	401	9

Filtered Regions			
	From	To	Length
1	148	148	1
2	375	377	3

ModPred and PROSITE:

ModPred: Proteolytic cleavage (R9, R12, D321, R431, R433), Sumoylation (K178), Sulfation (Y320).

PROSITE: No identified domain recognition sites.

Structural modelling:

	Name	Title	Identity	Method	Oligo State	Ligands
☐	4mmi.1.A	Heparinase III protein	10.34	X-ray, 2.4Å	monomer	2 x CA
☐	3qwt.1.A	Putative GH105 family protein	24.18	X-ray, 2.2Å	homo-octamer	None

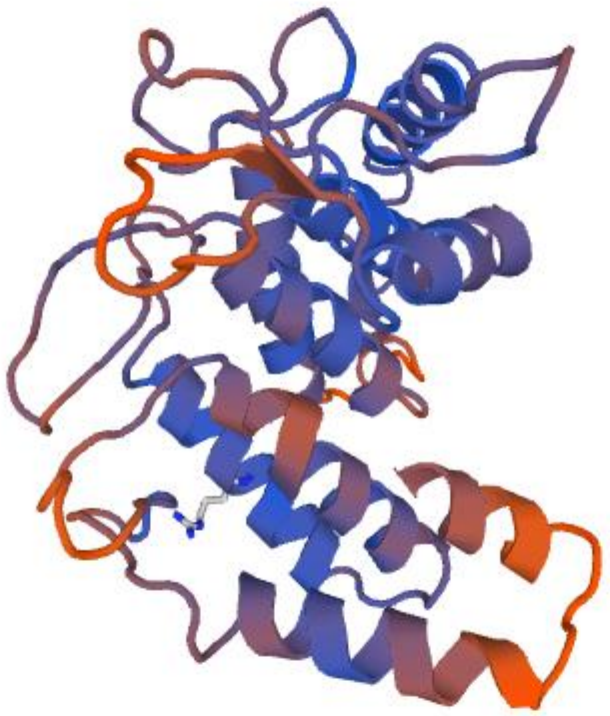

Model #1: Residues 150-402 of MVLG_03707T0 with 3qwt.1.A (24.18% sequence identity) as a template

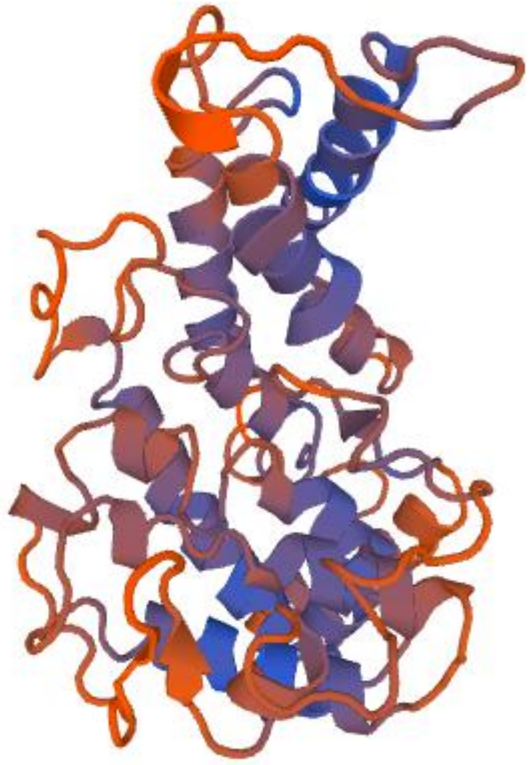

Model #2: Residues 45-378 of MVLG_03707T0 with 4mmi.1.A (10.34% sequence identity) as a template

> MVLG_01732T0 (22.44%)- 156 aa

MLLKLTITLIVALLVLNVSALQEAGDTKAEFRLIKRAAAQKSNLTQPTENASFLLHHPIPFELNYDP
KIVYAVDVELLSEHDKSIPLAVQMAGDHTGAISTTFSTPYFAENSVKYRNVTLRVTEWSLPSHNTAP
KKKSSTIDRKIVCRNFSGKIHA

PONDR:

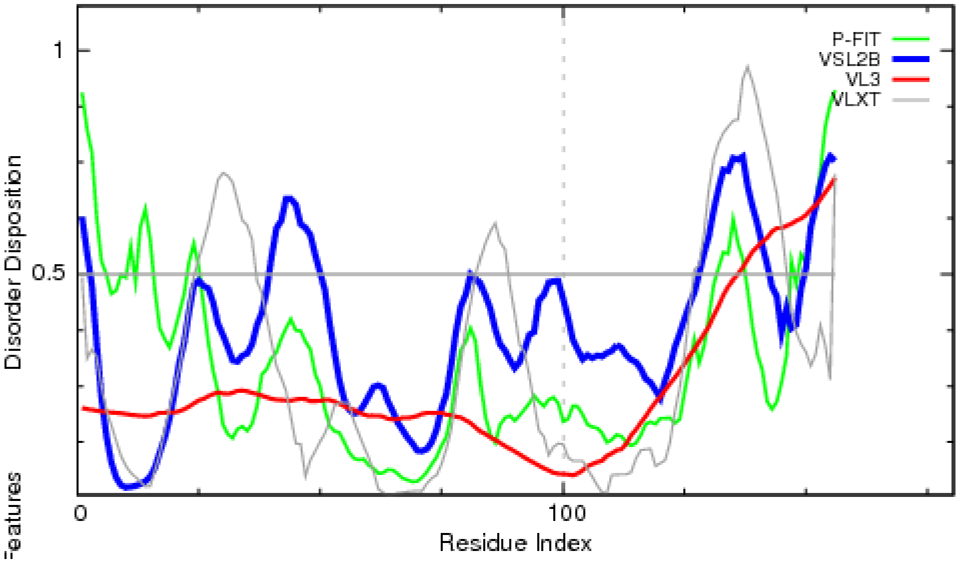

================================PONDR VSL2 STATISTICS================================

Predicted residues: 156	Number Disordered Regions: 4
Number residues disordered: 35	Longest Disordered Region: 15
Overall percent disordered: 22.44	Average Prediction Score: 0.3764
Predicted disorder segment [1]-[2]	Average Strength= 0.5863
Predicted disorder segment [40]-[50]	Average Strength= 0.5999
Predicted disorder segment [128]-[142]	Average Strength= 0.6503
Predicted disorder segment [150]-[156]	Average Strength= 0.6757

ANCHOR:

Predicted Disordered Binding Regions			
	From	To	Length
1	145	153	9

ModPred and PROSITE:

ModPred: Amidation (Y115).

PROSITE: No identified domain recognition sites.

Structural modelling:

	Name	Title	Identity	Method	Oligo State	Ligands
	2lzr.1.A	Sec-independent protein translocase protein TatA	20.59	NMR	monomer	None
	5cwb.1.A	Designed helical repeat protein	28.21	X-ray, 1.5Å	monomer	None
	5bwd.1.A	benzylsuccinate synthase alpha chain	17.65	X-ray, 2.0Å	hetero-oligomer	1 x FUM
	2jn8.1.A	Putative cytoplasmic protein	39.29	NMR	monomer	None

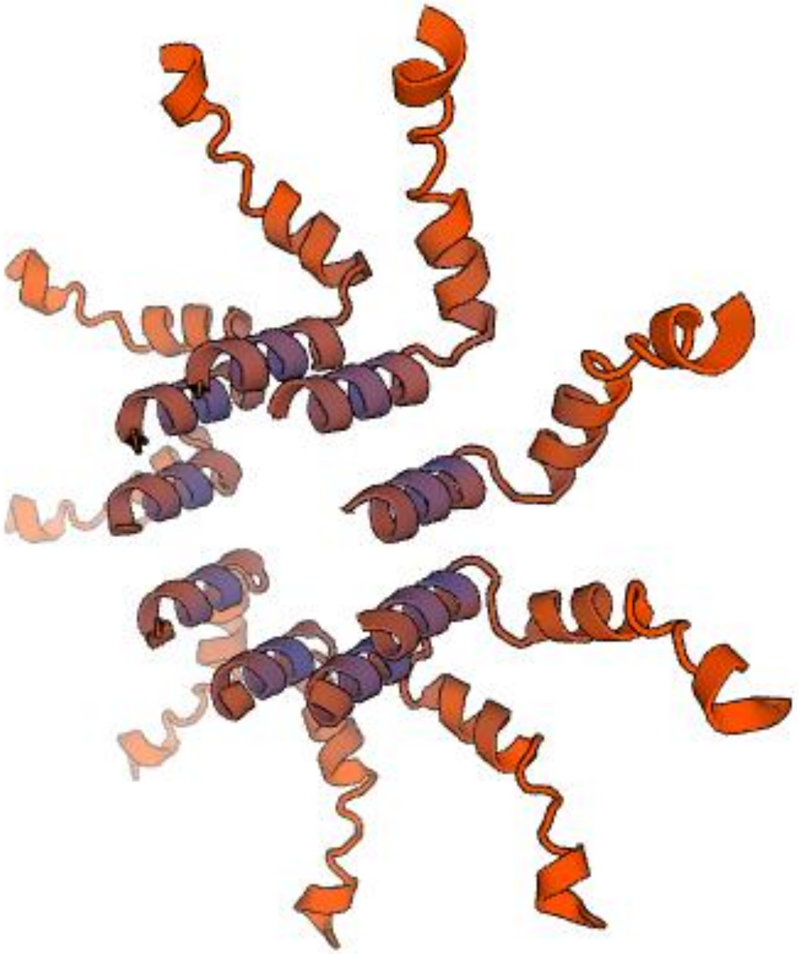

Model #1: Residues 4-37 of MVLG_01732T0 with 2lzs.1.E (20.59 % sequence identity) as a template

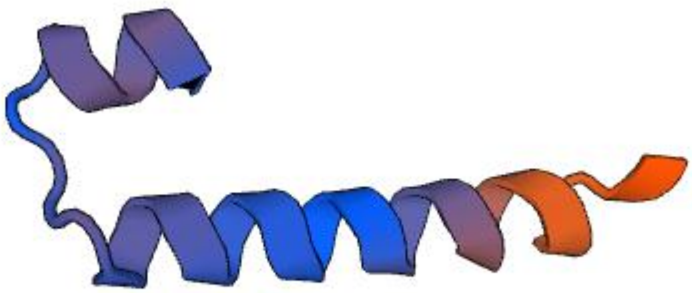

Model #2: Residues 18-51 of MVLG_01732T0 with 5cwb.1.A (28.21 % sequence identity) as a template

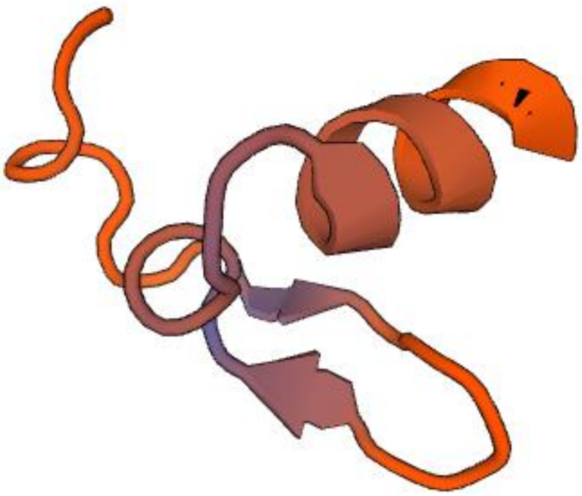

Model #3: Residues 110-143 of MVLG_01732T0 with 5bwd.1.A (17.65 % sequence identity) as a template

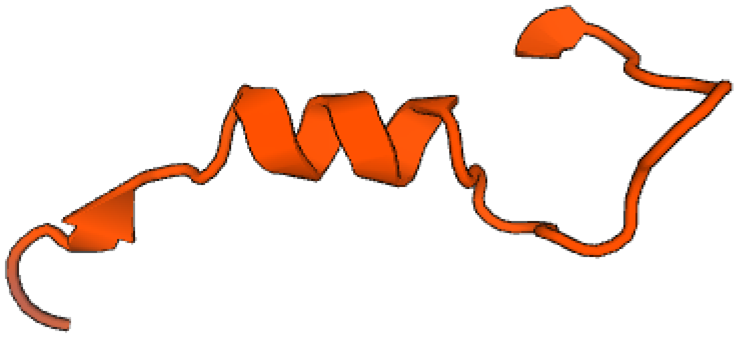

Model #4: Residues 81-110 of MVLG_01732T0 with 2jn8.1.A (36.67 % sequence identity) as a template

> MVLG_01191T0 (21.19%)- 505 aa

MRYSRQLWFGLGLLSQLVTAVTPEVYNNGDCTSSRRFRGLAHTNALMRHSADTFDAPRGVPLLA
LSAPNQMSKIVGLGKRSPAVPAGVSVSVRANVEVGGQSGLQPCADDCDHPQPPPSEEPIVISGPGTLP
RSDSYLTLDDARTGLLLDDEPFRPVGINIYWLCNDENIEGRPKGYPTDKTRVREALAAAVAMGANT
VRIGSCGTSLGFHDAIQPDLHHYADDDGMDIHDYAIWAAGRYDLKVILTLTDNYDYYHGGKYTILR
WLGEPTDDAGARFFADERPIQVYLRYAKWVLGRVNRYNNIAYGEDPTVSIIETGNELGAYMGKEG
YPPLNWTDRVAQRIKQLAPLALVMDGTDGIYNWSTKATAPGLLSPHIDIVTDHPYPRDINLFRTQAQ
LAKSANKVFLLGEMNWLPTGATNANLSDYLEVLDKYPSVGVLVWSLFTHDSQCSEYVLHNDSYSI
YYPDGPNTPEEKQNIWSLVQWFYRVTDRAVPAVLPVQACPQEVF

PONDR:

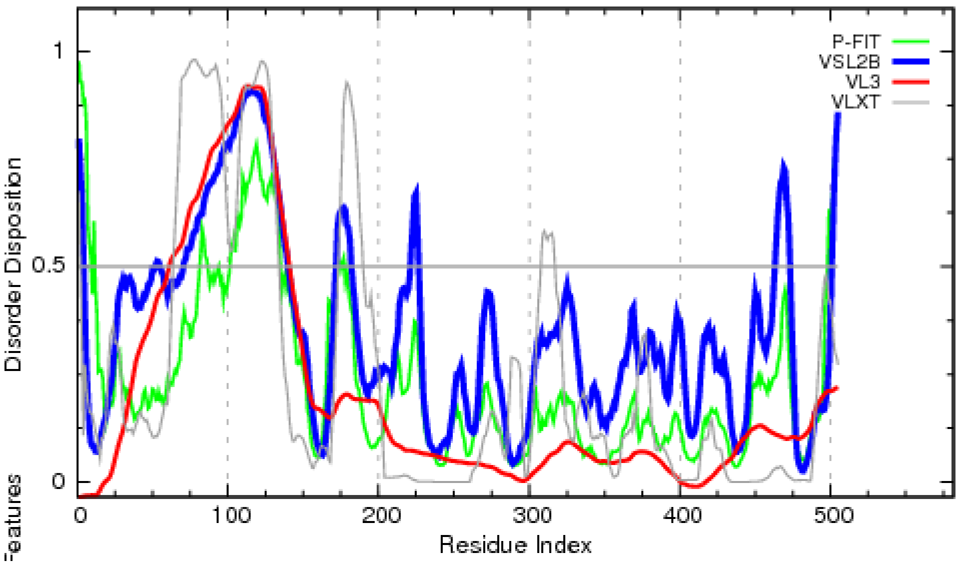

===============================PONDR VSL2 STATISTICS===============================

Predicted residues: 505	Number Disordered Regions: 7
Number residues disordered: 107	Longest Disordered Region: 69
Overall percent disordered: 21.19	Average Prediction Score: 0.3548
Predicted disorder segment [1]-[4]	Average Strength= 0.6816
Predicted disorder segment [52]-[54]	Average Strength= 0.5082
Predicted disorder segment [71]-[139]	Average Strength= 0.7406
Predicted disorder segment [172]-[182]	Average Strength= 0.5923
Predicted disorder segment [222]-[226]	Average Strength= 0.6133
Predicted disorder segment [464]-[473]	Average Strength= 0.6476
Predicted disorder segment [501]-[505]	Average Strength= 0.7122

ANCHOR:

Predicted Disordered Binding Regions			
	From	To	Length
1	88	94	7
2	103	108	6
3	136	144	9
4	157	166	10

Filtered Regions			
	From	To	Length
1	61	65	5
2	146	148	3

ModPred and PROSITE:

ModPred: Amidation (A20, P82, D337, P492), Proteolytic cleavage (R36, R38, R58, R276), ADP-ribosylation (R80, R133), O-linked glycosylation (S126).

PROSITE: No identified domain recognition sites.

Structural modelling:

	Name	Title	Identity	Method	Oligo State	Ligands
	1uuq.1.A	MANNOSYL-OLIGOSACCHARIDE GLUCOSIDASE	19.59	X-ray, 1.5Å	monomer	None
	5byw.1.A	Endoglucanase H	28.21	X-ray, 1.5Å	monomer	None

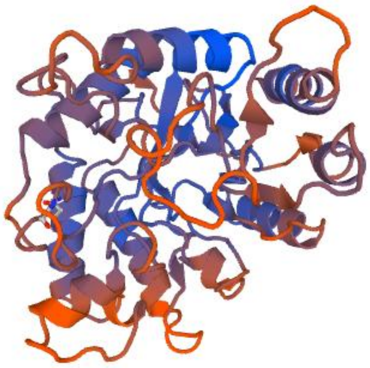

Model #1: Residues 135-488 of MVLG_01191T0 with 1uuq.1.A (19.59 % sequence identity) as a template

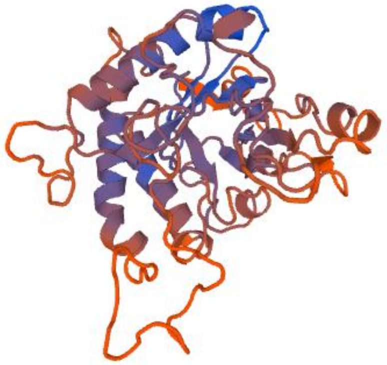

Model #2: Residues 137-467 of MVLG_01191T0 with 5byw.1.A (15.33 % sequence identity) as a template

> MVLG_05525T0 (20.63%)- 126 aa

MCRSSNMARPGMLILALITTVAPIAIALTHVENACAKEAVLHNDLSDGAKCKSITDLGCVCSESTG
DAFLRSLGDYVKDGRGRCREQYFANIQAYACAYCLFKDLVPPQSCGKASVSTVPTESDVQ

PONDR:

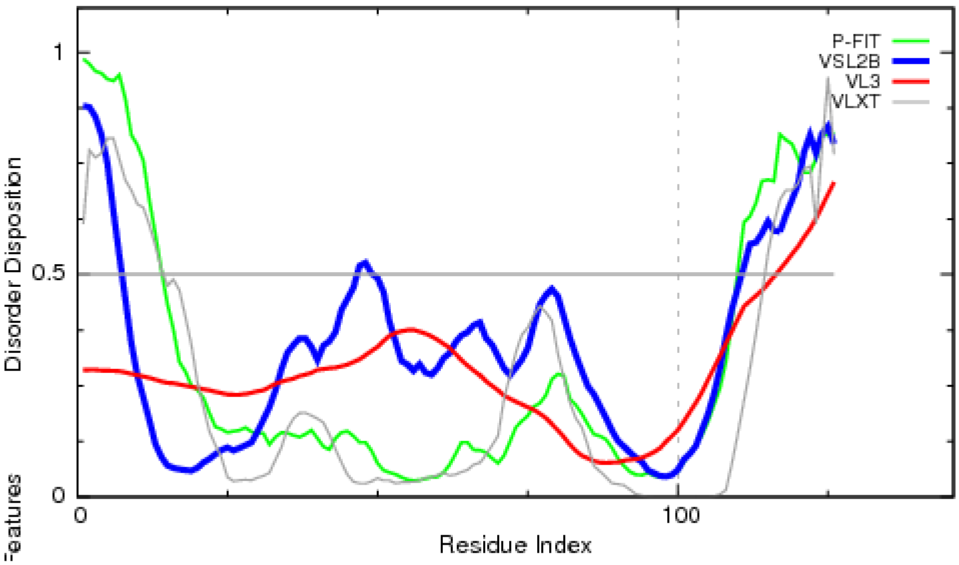

==============================PONDR VSL2 STATISTICS==============================

Predicted residues: 126	Number Disordered Regions: 3
Number residues disordered: 26	Longest Disordered Region: 16
Overall percent disordered: 20.63	Average Prediction Score: 0.3398
Predicted disorder segment [1]-[7]	Average Strength= 0.7662
Predicted disorder segment [47]-[49]	Average Strength= 0.5157
Predicted disorder segment [111]-[126]	Average Strength= 0.6798

ANCHOR:

Predicted Disordered Binding Regions			
	From	To	Length
None			

ModPred and PROSITE:

ModPred: GPI anchor amidation (N6).

PROSITE: No identified domain recognition sites.

Structural modelling:

	Name	Title	Identity	Method	Oligo State	Ligands
	5hiu.1.A	GTPase activator-like protein	10.53	X-ray, 2.5Å	monomer	None

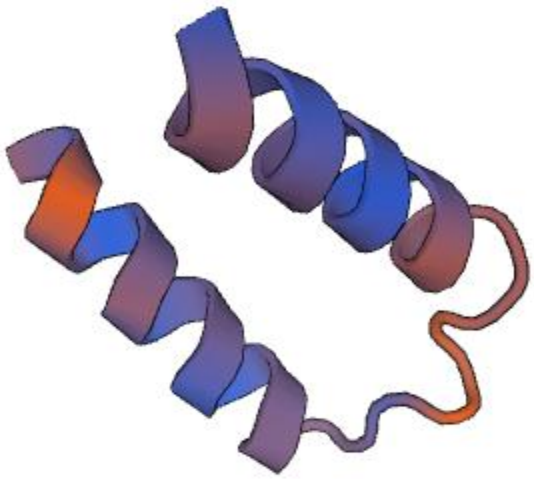

Model #1: Residues 64-101 of MVLG_05525T0 with 5hiu.1.A (10.53 % sequence identity) as a template

> MVLG_02331T0 (19.73%)- 679 aa

MWACFSASLVTVTCAALASAHDPFSRSTIRHSRRYSLLPDTYILETECPDASILAEASPRTSGANMD
SLVTEVLAAFPDARPRHVYDSALFCGLSVELGADSPHTQLLRIQRLKSVSPVRSIQLGVRTAGSSDTP
FQAEASLNVVPRTEARGESDYLSSHVMLEVDKMHEMGLFGSRETLACVIDSGIDLMHPLLGNGCFG
SNCKVVTGYDFVGDDGHSPKRSPQTSCSDHGTHIAGILAADKFKAFGFSGVAPNASLGVYRVFSCK
GAASSDTFLKAMLMAADDGCRVLSLSFGKALGWDQDDGDDPFRKVVSRLATRGVFIAAASGNDA
SQGLMFAQTPADLAGILAVGSVEPVAAPRGFKLSFEHNRYPSMTYLALRPVNHSQTFQIHFHSIRRA
KDTSCDPLLPRSSNFTNSVVVLQKGACGTKLIHFFVRHGARVVIAHDNGDPEQAQNWRRTAYAVH
SQEGLEWLLKWPTSAVHTLLDHYLDSLGDLQVNFRSKDPVPQDELIDRVAGGLVSEYTEFGPAATL
DTLAAHVSAPGSSILSTFPLNKGGYGVASGTSMATPMAAGVATLLISHRKDDHLTPAQIRSLMITTA
GPVATKLNSVHPLTTVMQQGGGLVSAHRAYHAMTLIWPYALALYDTPRHVKDHVVTLTNTHKSV
VTYSFNSVPSQTLAMYNKV

PONDR:

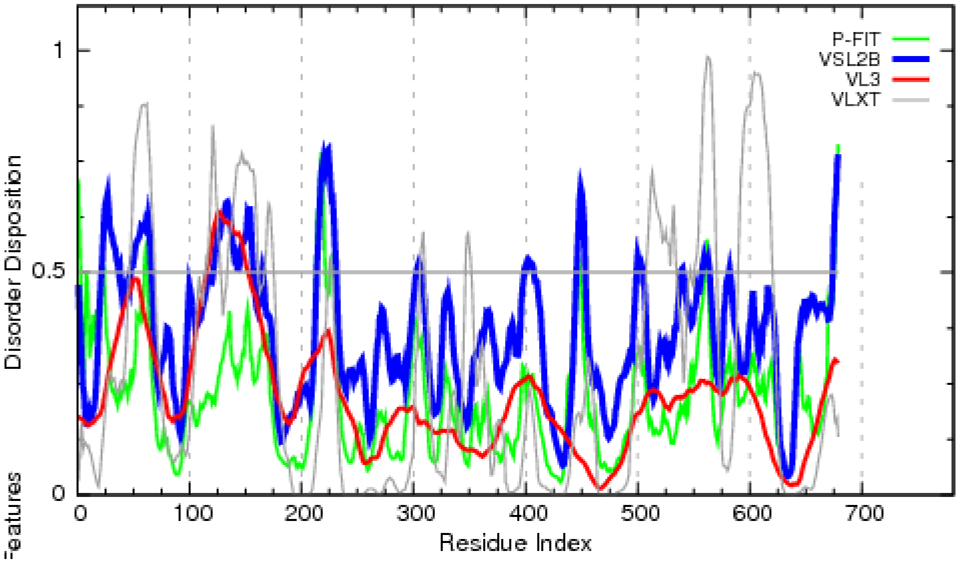

===============================PONDR VSL2 STATISTICS===============================

Predicted residues: 679	Number Disordered Regions: 13
Number residues disordered: 134	Longest Disordered Region: 36
Overall percent disordered: 19.73	Average Prediction Score: 0.3656
Predicted disorder segment [22]-[38]	Average Strength= 0.5801
Predicted disorder segment [48]-[66]	Average Strength= 0.5721
Predicted disorder segment [122]-[157]	Average Strength= 0.5821
Predicted disorder segment [165]-[165]	Average Strength= 0.5088
Predicted disorder segment [214]-[231]	Average Strength= 0.6758
Predicted disorder segment [303]-[307]	Average Strength= 0.5114
Predicted disorder segment [399]-[407]	Average Strength= 0.5154
Predicted disorder segment [446]-[453]	Average Strength= 0.6192
Predicted disorder segment [499]-[504]	Average Strength= 0.5225
Predicted disorder segment [539]-[540]	Average Strength= 0.5076
Predicted disorder segment [558]-[564]	Average Strength= 0.5195
Predicted disorder segment [582]-[582]	Average Strength= 0.5082
Predicted disorder segment [675]-[679]	Average Strength= 0.6825

ANCHOR:

Predicted Disordered Binding Regions			
	From	To	Length
1	88	95	8
**Filtered Regions**			
	From	To	Length
1	429	432	4

ModPred and PROSITE:

ModPred: Proteolytic cleavage (Y35, Y210, D211, R310), O-linked glycosylation (S62, S227), Phosphorylation (S117), N-linked glycosylation (N197), Amidation (S664)

PROSITE: Subtilase_ASP (181-192, PROSITE entry PS00136), Subtilase_HIS (231-241, PROSITE Entry PS00137), Subtilase_SER (559-569, PROSITE entry PS00138)

Subtilases are an extensive family of serine proteases whose catalytic activity is provided by a charge relay system similar to that of the trypsin family of serine proteases but which evolved by independent convergent evolution. Subtilase family currently includes the following proteases in Fungi:

- Alkaline extracellular protease (AEP) from Yarrowia lipolytica (gene xpr2).
- Alkaline proteinase from Cephalosporium acremonium (gene alp).
- Cerevisin (EC 3.4.21.48) (vacuolar protease B) from yeast (gene PRB1).
- Cuticle-degrading protease (pr1) from Metarhizium anisopliae.
- KEX-1 protease from Kluyveromyces lactis.
- Kexin (EC 3.4.21.61) from yeast (gene KEX-2).
- Oryzin (EC 3.4.21.63) (alkaline proteinase) from Aspergillus (gene alp).
- Proteinase K (EC 3.4.21.64) from Tritirachium album (gene proK).
- Proteinase R from Tritirachium album (gene proR).
- Proteinase T from Tritirachium album (gene proT).
- Subtilisin-like protease III from yeast (gene YSP3).
- Thermomycolin (EC 3.4.21.65) from Malbranchea sulfurea.

Structural modelling:

	Name	Title	Identity	Method	Oligo State	Ligands
	3eif.1.A	C5a peptidase	23.48	X-ray, 1.9Å	monomer	1 x CA, 1 x MLA
	4i0w.1.B	Protease CspB	18.69	X-ray, 1.6Å	hetero-oligomer	None
	1r6v.1.A	subtilisin-like serine protease	24.16	X-ray, 1.7Å	monomer	1 x CA
	1y9z.1.A	alkaline serine protease	23.16	X-ray, 1.4Å	monomer	2 x CA, 1 x PMS
	3i74.1.A	Subtilisin-like protease	32.94	X-ray, 2.6Å	homo-dimer	1 x NAG-NAG, 1 x NAG-FUC, 2 x ACE-PHE-GLU-LYS-ALV-0QE

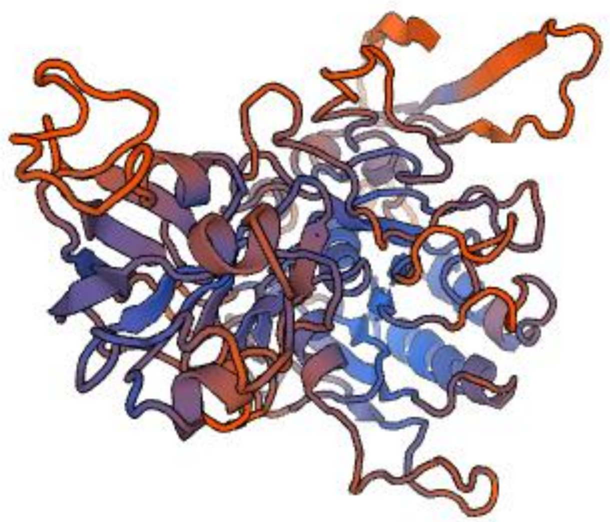

Model #1: Residues 160-667 of MVLG_02331T0 with 3eif.1.A (23.48 % sequence identity) as a template

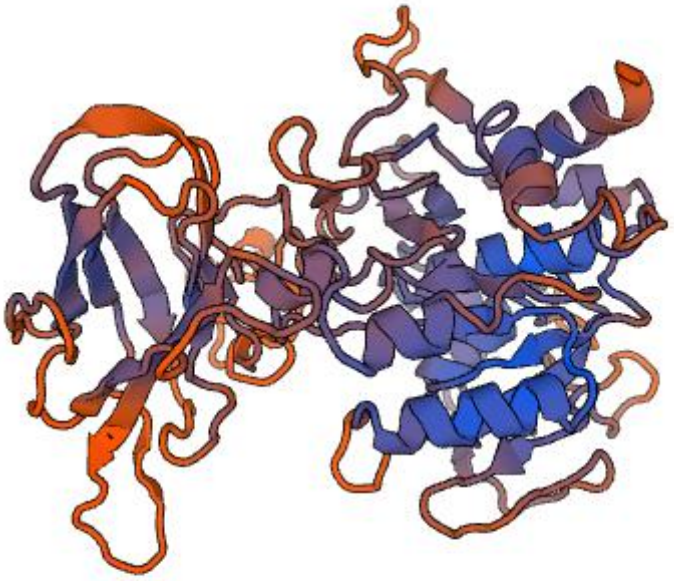

Model #2: Residues 160-632 of MVLG_02331T0 with 4i0w.1.B (18.69% sequence identity) as a template

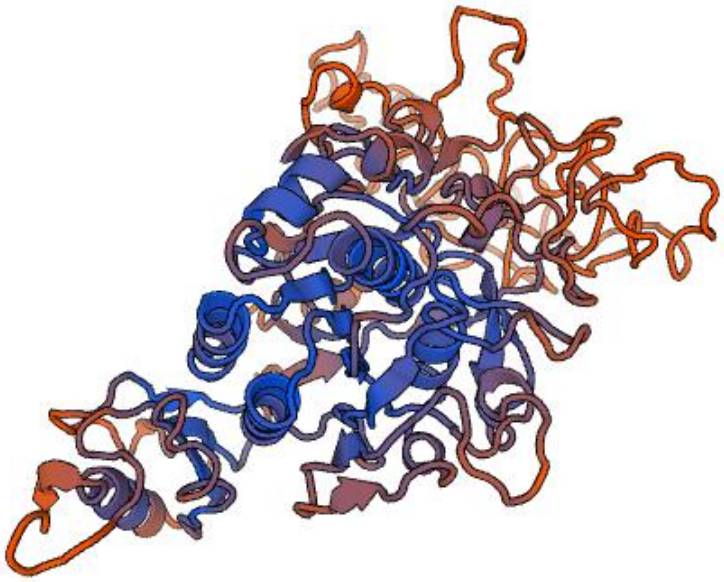

Model #3: Residues 34-630 of MVLG_02331T0 with 1r6v.1.A (24.16% sequence identity) as a template

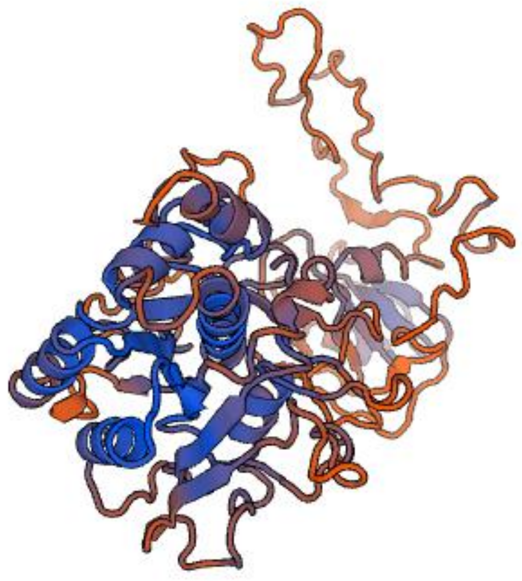

Model #4: Residues 163-633 of MVLG_02331T0 with 1y9z.1.A (23.16% sequence identity) as a template

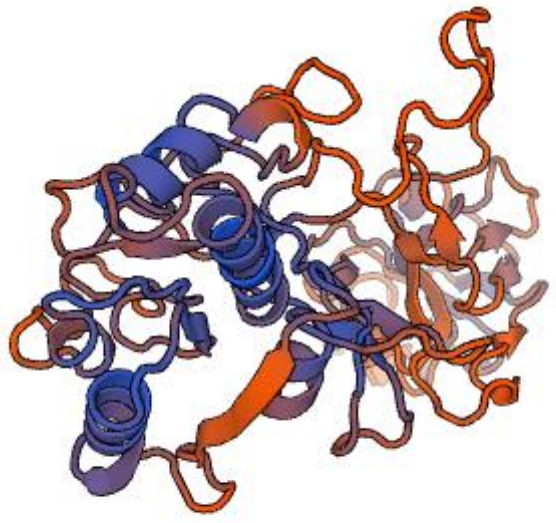

Model #5: Residues 231-625 of MVLG_02331T0 with 3i74.1.A (32.94% sequence identity) as a template

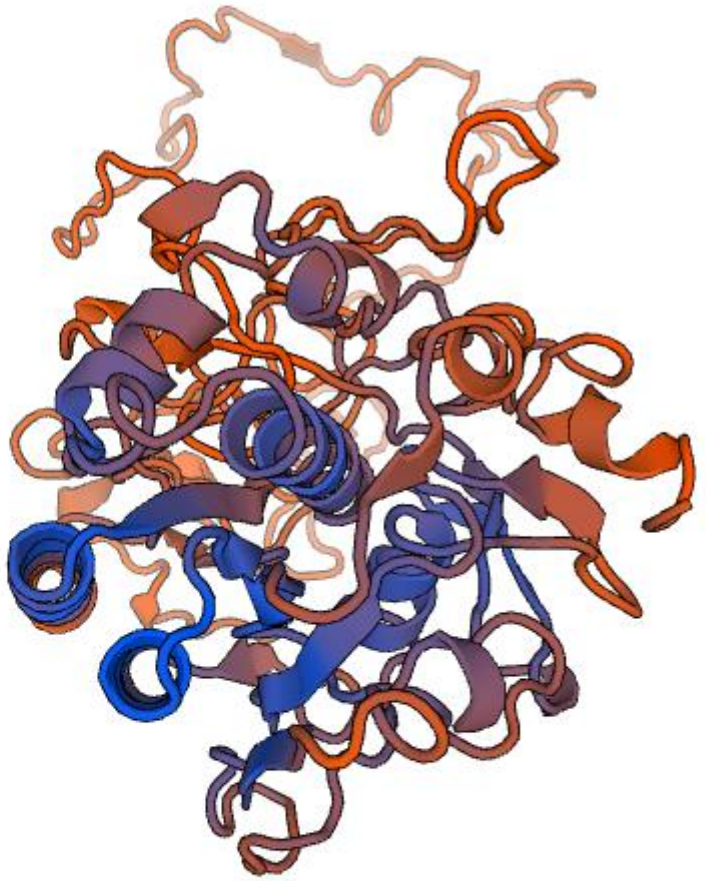

Model #6: Residues 139-631 of MVLG_02331T0 with 4mzd.1.A (20.48% sequence identity) as a template

> MVLG_02184T0 (19.34%)- 641 aa

MLIGDLLLPCAFAALGRLVLASPLPLSEFETELHRHKTSPGHPEKGSVFHSAYKPLWEAHRRGQQL
ETLKKVTYDHEDYRLSSDFEITDIPTERNYYFDVSEVTAAPDGVTRKMFLVNARVNGELIEANEGDT
IKLHVRNWLRVGTGIHFHGIPQAHVNYFDGPVGVVTCPIASKSEFTFSFKLVNVCGTYFWHGHRSTQ
SVDGINGPVVVHCRNDTLKKGADFDREQVVMVTDNYHELSSVIMEKLRSSAGVYGSTSTPTPKSGL
IQGRGDFDCKNRTNILKGHSCKKQSIYSEIAVPAGSLTRLRFINAGMHAFWRISVDEHEMKLIEVDDT
PIDAVGMPRIPINAGQRFSAVLDTRSDKAGSSFWMRSFAATQCFRAPLNGFNPETLAIVRVVDPYAS
TSSSSGQQKFPTSKPFTHDLVELCEDPATSILRPRVAENTADTADQVDYFNATYILAPEGGRFYMNGI
SFEAYAYDPLLFRAIRGESIANRSATVIMSETTGEGGRARVHDIIVNNPGGGAHPFHLHGPRSYIVGG
GDGTISKETWATMTPMTQNPTRRDVFTVPPNSYIVIRIVADLVGVHAFHCHVSPHTSVGMAGALVV
RPDLIRQIQLPQESIDMCKASHYDSGFSEETPESARRR

PONDR:

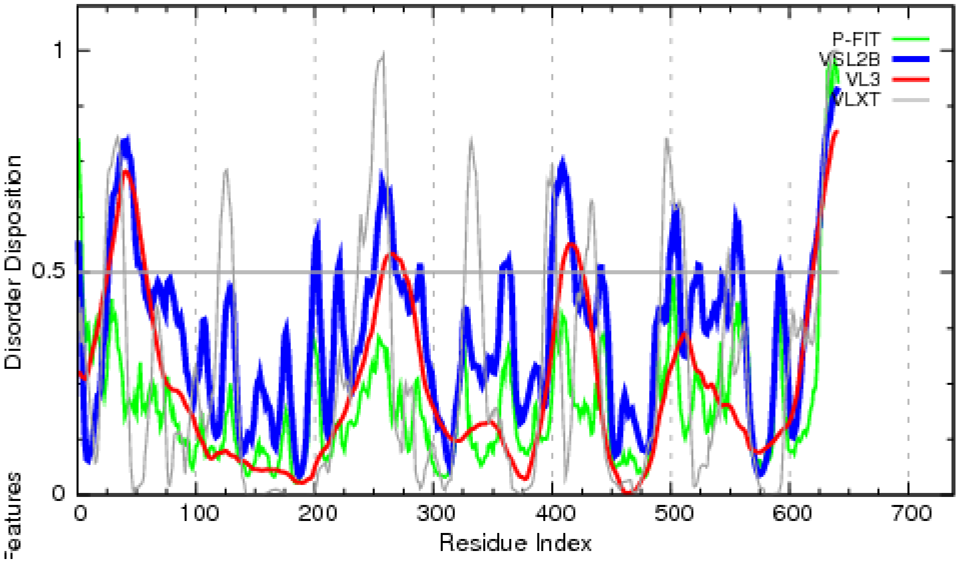

================================PONDR VSL2 STATISTICS================================

Predicted residues: 641	Number Disordered Regions: 13
Number residues disordered: 124	Longest Disordered Region: 28
Overall percent disordered: 19.34	Average Prediction Score: 0.3630
Predicted disorder segment [27]-[54]	Average Strength= 0.6757
Predicted disorder segment [200]-[203]	Average Strength= 0.5562
Predicted disorder segment [220]-[221]	Average Strength= 0.5163
Predicted disorder segment [251]-[268]	Average Strength= 0.6264
Predicted disorder segment [289]-[289]	Average Strength= 0.5192
Predicted disorder segment [358]-[364]	Average Strength= 0.5156
Predicted disorder segment [399]-[418]	Average Strength= 0.6553
Predicted disorder segment [441]-[441]	Average Strength= 0.5179
Predicted disorder segment [499]-[506]	Average Strength= 0.5902
Predicted disorder segment [523]-[524]	Average Strength= 0.5158
Predicted disorder segment [552]-[559]	Average Strength= 0.5641
Predicted disorder segment [592]-[592]	Average Strength= 0.5069
Predicted disorder segment [619]-[641]	Average Strength= 0.7449

ANCHOR:

Predicted Disordered Binding Regions			
	From	To	Length
None			

Filtered Regions			
	From	To	Length
1	5	18	14
2	474	477	4
3	479	479	1
4	570	586	17

ModPred and PROSITE:

ModPred: Amidation (A14, F316, S405, R507, I571), Proteolytic cleavage (Q200, R394, R560, D561, D627, F630, R641), O-linked glycosylation (S256), Phosphorylation (T259), ADP-ribosylation (R270)

PROSITE: Multicopper oxidase2 (586-597, PROSITE entry PS00080)

Multicopper oxidases [1,2] are enzymes that possess three spectroscopically different copper centers. These centers are called: type 1 (or blue), type 2 (or normal) and type 3 (or coupled binuclear). Consensus pattern: H-C-H-x(3)-H-x(3)-[AG]-[LM]

The first 2 H's are copper type 3 binding residues; The C, the third H, and L or M are copper type 1 ligands. The enzymes that belong to this family are:

- Laccase (EC 1.10.3.2) (urishiol oxidase), an enzyme found in fungi and plants, which oxidizes many different types of phenols and diamines.
- In addition to the above enzyme there are a number of proteins which, on the basis of sequence similarities, can be said to belong to this family. These proteins are:
- Copper resistance protein A (copA) from a plasmid in Pseudomonas syringae. This protein seems to be involved in the resistance of the microbial host to copper.
- Yeast FET3, which is required for ferrous iron uptake.
- Yeast hypothetical protein YFL041w and SpAC1F7.08, the fission yeast homolog.

Structural modelling:

	Name	Title	Identity	Method	Oligo State	Ligands
☐	3v9e.1.A	Laccase	27.48	X-ray, 1.7Å	monomer	5 x NAG, 3 x CU, 1 x MAN
☐	1aso.1.A	ASCORBATE OXIDASE	27.23	X-ray, 2.2Å	homo-dimer	2 x NAG, 9 x CU, 2 x OH
☐	5mew.1.A	Laccase 2	28.06	X-ray, 1.3Å	monomer	4 x NAG, 4 x CU, 2 x OXY

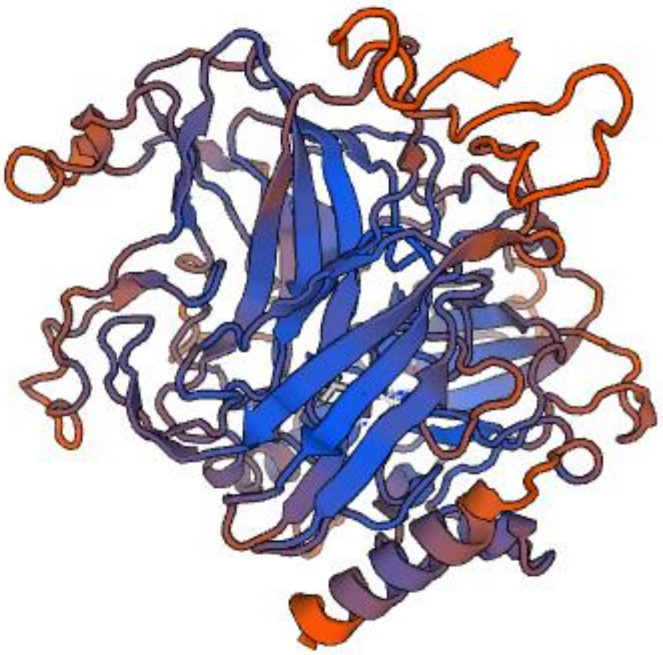

Model #1: Residues 50-629 of MVLG_02184T0 with 3v9e.1.A (27.48% sequence identity) as a template

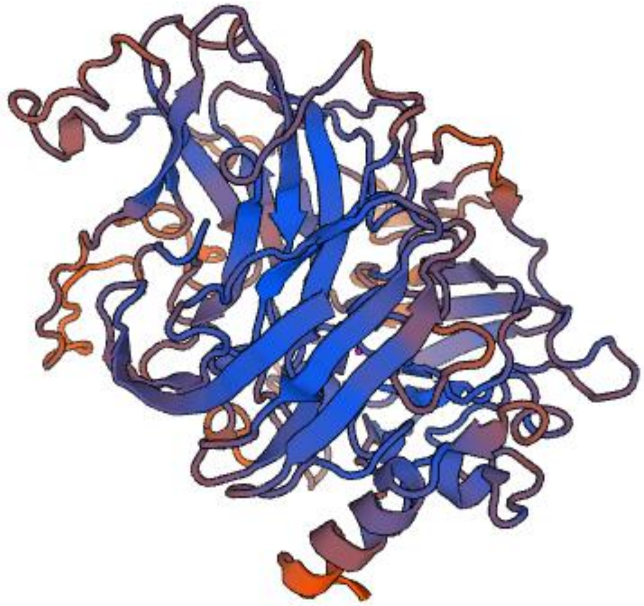

Model #2: Residues 95-631 of MVLG_02184T0 with 5mew.1.A (28.06% sequence identity) as a template

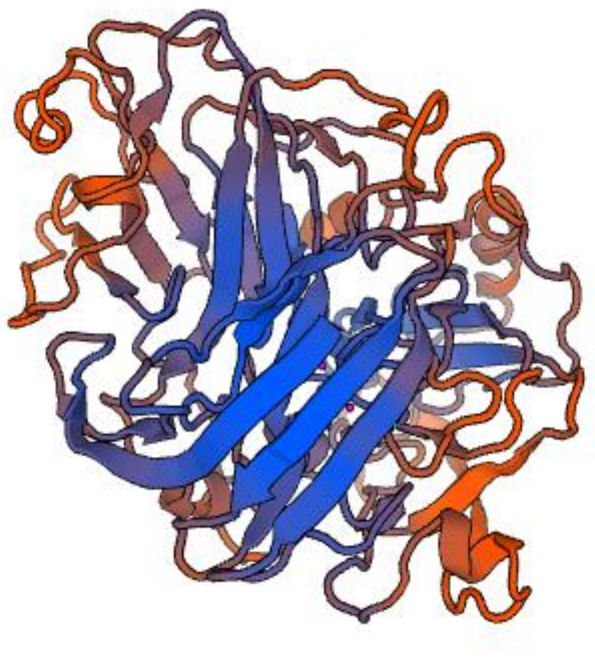

Model #3: Residues 94-598 of MVLG_02184T0 with 1aso.1.A (27.23% sequence identity) as a template

> MVLG_07060T0 (18.95%)- 496 aa

MMWSTLLVPITAALAATAVHAATNHAAVGAHSSLDHNKGSQNVAKGDISKGNTFQVISHPDFPN
HKLRIKESQLCGDKEKIYSGFLDIAEHTHLFFAFAESRDKPDEDSVLLWLNGGPGCSSMAGFLLENG
PCLVTNGGNSSTFNPYSWNSNANMIFLDSPVKVGFSNARKPVDTSRKTAEDIYAFMQLFYQVFPRF
AMLDFILAGESYAGMYIPQVASVIVQKNKLVDGASSNTIYVPLVSMAIGNGFVEIVSALSAEVDFAC
GKGVHKAIYNSSTCDALYPQIPICSRSVATCRQNLTRQNCQQAELDCFVLGAPFDNTGLNPYDVTK
KCDRSPSKDGPLCYKEASWLPIYLNRPDIRAKLGVHAKAKPFEECSDSVHTAFLLSGDWVVNTPAV
LSDLLEAGIKLLLYVGVNDFICNYLGVRNWTTAMKWSGQDQYSKAPFHEFRMPNGTVVGLTKSYG
PLTYLEVKDAGHMVPRDKPDEALEMIKTWIRGDQF

PONDR:

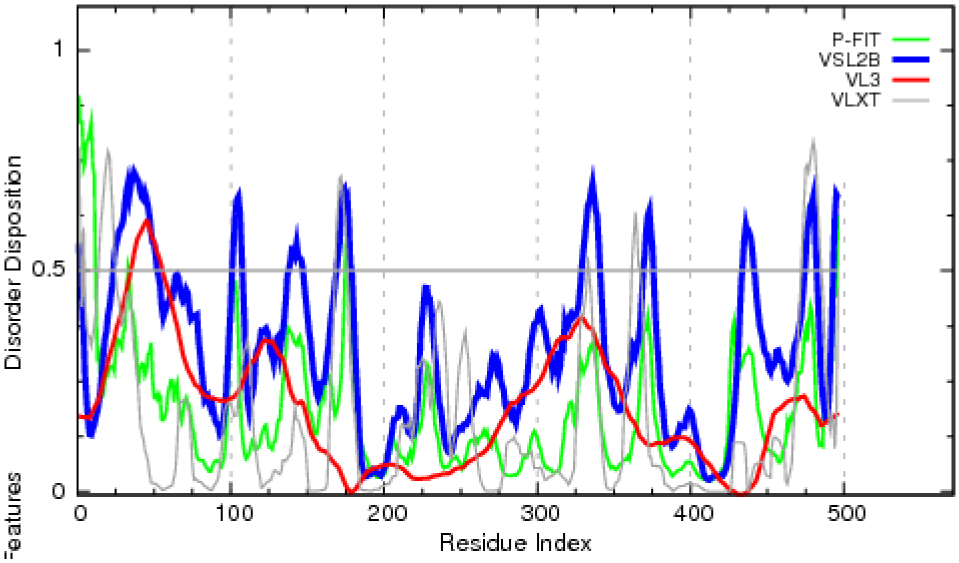

================================PONDR VSL2 STATISTICS================================

Predicted residues: 496	Number Disordered Regions: 9
Number residues disordered: 94	Longest Disordered Region: 29
Overall percent disordered: 18.95	Average Prediction Score: 0.3250
Predicted disorder segment [24]-[52]	Average Strength= 0.6341
Predicted disorder segment [101]-[107]	Average Strength= 0.6034
Predicted disorder segment [138]-[147]	Average Strength= 0.5319
Predicted disorder segment [170]-[178]	Average Strength= 0.6156
Predicted disorder segment [329]-[340]	Average Strength= 0.6186
Predicted disorder segment [370]-[375]	Average Strength= 0.5888
Predicted disorder segment [434]-[441]	Average Strength= 0.5633
Predicted disorder segment [475]-[482]	Average Strength= 0.6040
Predicted disorder segment [493]-[496]	Average Strength= 0.6175

ANCHOR:

Predicted Disordered Binding Regions			
	From	To	Length
None			

Filtered Regions			
	From	To	Length
1	1	3	3
2	6	8	3
3	94	96	3

ModPred and PROSITE:

ModPred: Proteolytic cleavage (H31, D108, S109, L111, W113, K362, H473), Amidation (Y465)

PROSITE: CARBOXYPEPT_SER_SER(Serine carboxypeptidases, serine active site, 204-211, PROSITE entry PS00131), CARBOXYPEPT_SER_HIS (Serine carboxypeptidases, histidine active site, 463-480, PROSITE entry PS00560)
All known carboxypeptidases are either metallo carboxypeptidases or serine carboxypeptidases (EC 3.4.16.5 and EC 3.4.16.6). The catalytic activity of the serine carboxypeptidases, is provided by a charge relay system involving an aspartic acid residue hydrogen-bonded to a histidine, which is itself hydrogen-bonded to a serine. Fungal Proteins known to be serine carboxypeptidases are:

- Yeast carboxypeptidase Y (YSCY) (gene PRC1), a vacuolar protease involved in degrading small peptides.
- Yeast KEX1 protease, involved in killer toxin and α-factor precursor processing.
- Fission yeast sxa2, a probable carboxypeptidase involved in degrading or processing mating pheromones.
- Penicillium janthinellum carboxypeptidase S1
- Aspergullus niger carboxypeptidase pepF.
- Aspergullus satoi carboxypeptidase cpdS.
- Yeast hypothetical protein YBR139w.

Structural modelling:

Name	Title	Identity	Method	Oligo State	Ligands
1ysc.1.A	SERINE CARBOXYPEPTIDASE	37.35	X-ray, 2.8Å	monomer	3 x NDG
1gxs.1.B	P-(S)-HYDROXYMANDELONITRILE LYASE CHAIN B	23.97	X-ray, 2.3Å	hetero-oligomer	2 x BEZ, 2 x NAG, 2 x DKA

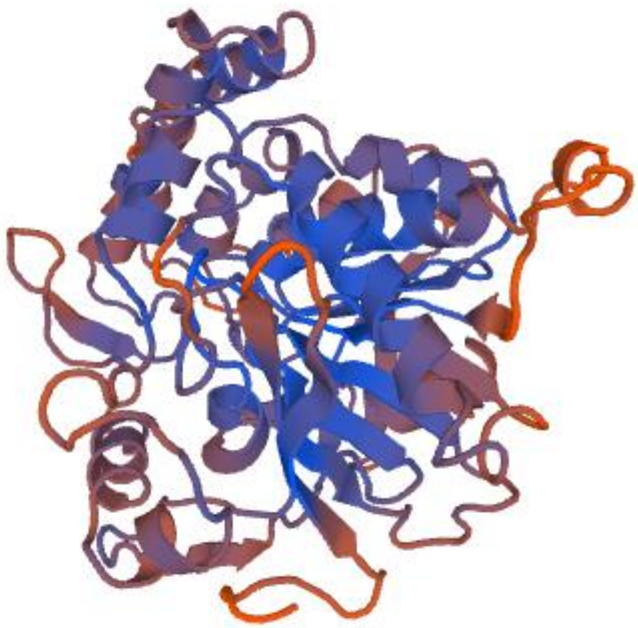

Model #1: Residues 72-495 of MVLG_07060T0 with 1ysc.1.A (37.35% sequence identity) as a template

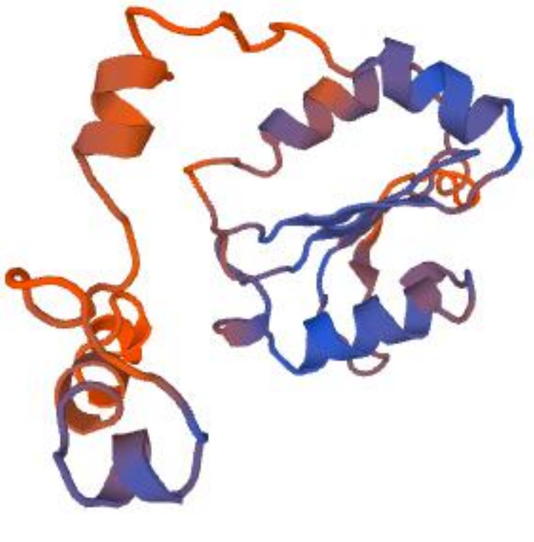

Model #2: Residues 341-496 of MVLG_07060T0 with 1gxs.1.B (23.97% sequence identity) as a template

> MVLG_01474T0 (16.32%)- 380 aa

MLSKLRNVSVAAALLFAGLAIAAPAPVSNSSLEARHGKQNLLTPKVMIISMFAPERAVWIKPMKL
VHNVSVVGLSPLYPYVACNNEYDVCIMTTGEAEINAAASMMALALSPLFCLQHTYFLIAGIGGVNP
YAGTLGSAAFARFAVQVALEYELDARQIPSNWTTGYWMQNTAGPGQLSATKDLYGTELFEVNTNL
LAKAYSAAKGVTLNDSTTAQAYRQKFDYAPANQPPQVILGDVATSDVYYAGTLLSESFGNYTALL
TNGTGKYTTTAQEDNATLESMVRATKAGLLDYARVIILRTCSDFDRAPPGKVTAYDAFFANQGGFE
LALQNLYIAGKPVVDMILKDWSTFKNGVQPQSKGNGSYYGDDLGTLRSGPALA

PONDR:

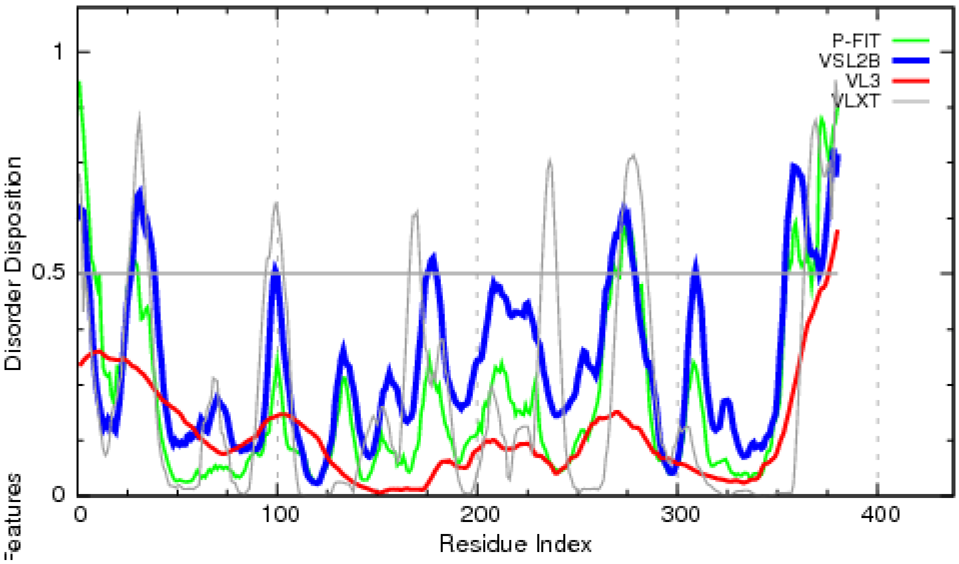

==============================PONDR VSL2 STATISTICS==============================

Predicted residues: 380	Number Disordered Regions: 7
Number residues disordered: 62	Longest Disordered Region: 27
Overall percent disordered: 16.32	Average Prediction Score: 0.2960
Predicted disorder segment [1]-[5]	Average Strength= 0.6035
Predicted disorder segment [27]-[38]	Average Strength= 0.6006
Predicted disorder segment [99]-[99]	Average Strength= 0.5068
Predicted disorder segment [176]-[179]	Average Strength= 0.5212
Predicted disorder segment [267]-[278]	Average Strength= 0.5886
Predicted disorder segment [309]-[309]	Average Strength= 0.5085
Predicted disorder segment [354]-[380]	Average Strength= 0.6394

ANCHOR:

Predicted Disordered Binding Regions			
	From	To	Length
None			

ModPred and PROSITE:

ModPred: GPI anchor amidation (N29), Amidation (A227, Q232, Y366), Proteolytic cleavage (R374).

PROSITE: No identified domain recognition sites.

Structural modelling:

	Name	Title	Identity	Method	Oligo State	Ligands
☐	1zos.1.A	5'-methylthioadenosine / S-adenosylhomocysteine nucleosidase	18.78	X-ray, 1.6Å	homo-dimer	2 x MTM
☐	4g89.1.B	5'-methylthioadenosine/S-adenosylhomocysteine nucleosidase	17.65	X-ray, 2.1Å	homo-dimer	1 x SAH, 1 x ADE

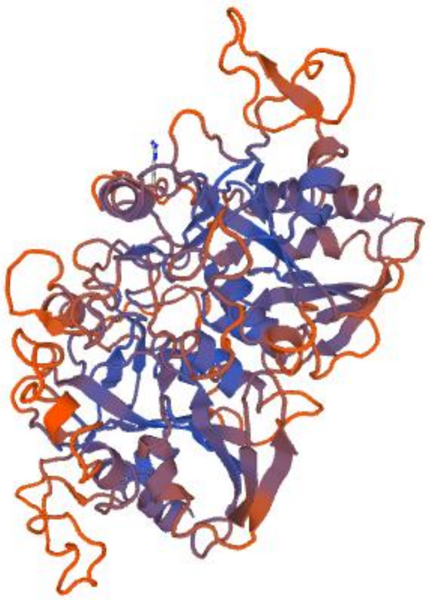

Model #1: Residues 45-349 of MVLG_01474T0 with 1zos.1.A (18.78% sequence identity) as a template

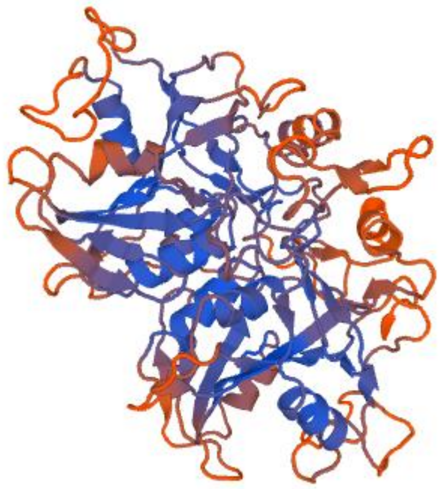

Model #2: Residues 44-311 of MVLG_01474T0 with 4g89.1.B (17.65% sequence identity) as a template

> MVLG_00784T0 (15.81%)- 215 aa

MLVSFKPFGLVWLCMSVFVSATFGRVTKDPKTPIVKGPKAPVVKEPKTPLVFQECKKYTYSKDIG
KLFKRKGVEGKATFFINGYNYGCIYNKENVNALRARYHEGHADLVNLSSVQIVKQVELLETAVERI
LGVRLGMFIAPYDSIDEKAAKVIRDKGYKIVRWSLDSGDTTFYLGRPQSSVNLIRKWIKKASGKSGI
GLFDEVGFIFLTCHGSE

PONDR:

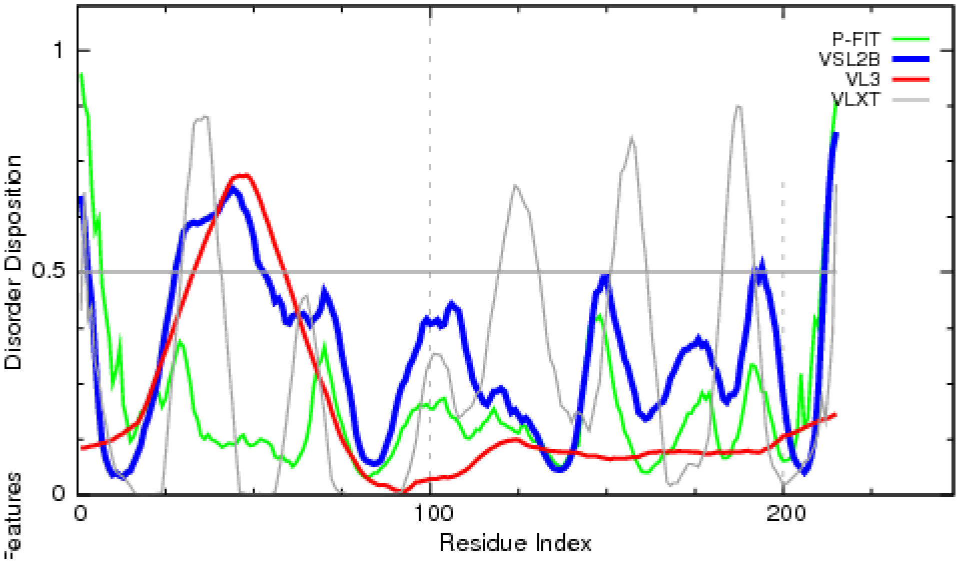

==============================PONDR VSL2 STATISTICS==============================

Predicted residues: 215
Number residues disordered: 34
Overall percent disordered: 15.81
Predicted disorder segment [1]-[3]
Predicted disorder segment [28]-[52]
Predicted disorder segment [192]-[192]
Predicted disorder segment [194]-[194]
Predicted disorder segment [212]-[215]

Number Disordered Regions: 5
Longest Disordered Region: 25
Average Prediction Score: 0.3172
Average Strength= 0.5966
Average Strength= 0.6149
Average Strength= 0.5020
Average Strength= 0.5114
Average Strength= 0.7071

ANCHOR:

Predicted Disordered Binding Regions			
	From	To	Length
None			

ModPred and PROSITE:

ModPred: Sumoylation (K6), Proteolytic cleavage (T22, F23, R25), Acetylation (K62, K66), Methylation (K69), Amidation (I89, V95, Y102), Ubiquitination (K92), Pupylation (K152).

PROSITE: No identified domain recognition sites.

Structural modelling:

	Name	Title	Identity	Method	Oligo State	Ligands
☐	2iw0.1.A	CHITIN DEACETYLASE	26.88	X-ray, 1.8Å	monomer	1 x ZN
☐	3qbu.1.A	Putative uncharacterized protein	16.81	X-ray, 2.6Å	homo-tetramer	4 x ZN
☐	3l6u.1.A	ABC-TYPE SUGAR TRANSPORT SYSTEM PERIPLASMIC COMPONENT	17.39	X-ray, 1.9Å	homo-dimer	None
☐	2eqo.1.A	TNF receptor-associated factor 3-interacting protein 1	13.21	NMR	monomer	None

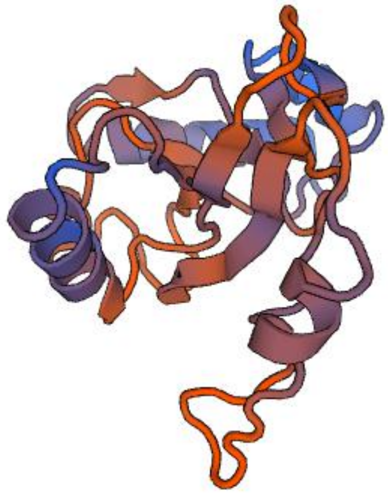

Model #1: Residues 37-203 of MVLG_00784T0 with 2iw0.1.A (26.88% sequence identity) as a template

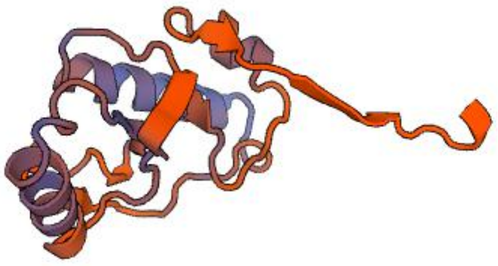

Model #2: Residues 48-173 of MVLG_00784T0 with 3qbu.1.A (16.81% sequence identity) as a template

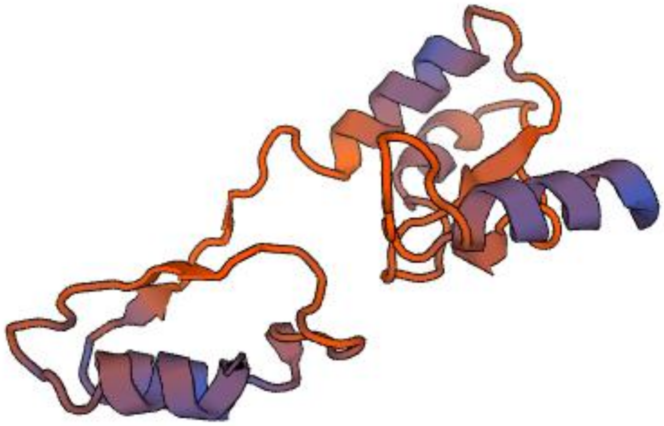

Model #3: Residues 61-191 of MVLG_00784T0 with 3l6u.1.A (17.39% sequence identity) as a template

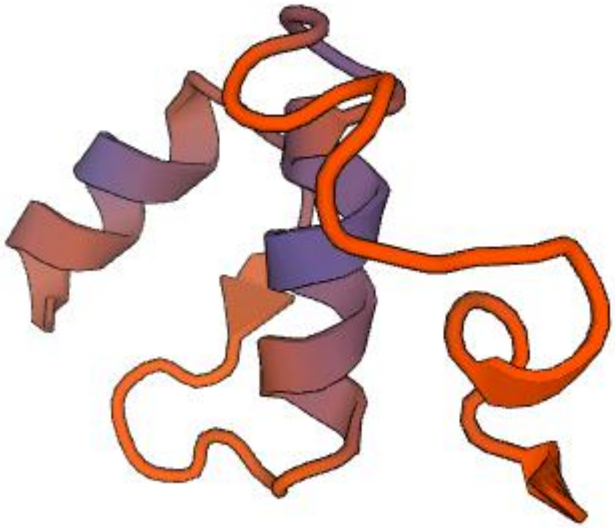

Model #4: Residues 121-173 of MVLG_00784T0 with 2eqo.1.A (13.21% sequence identity) as a template

> MVLG_06541T0 (15.48%)- 84 aa

MRFSLIVLATLLVGFAAAAPVLQSDIFKDTQKADRAQNLVHRLMVRVSTVYSQCTHNCEDEYAR
YKIGPYELIACKKGCQSDAI

PONDR:

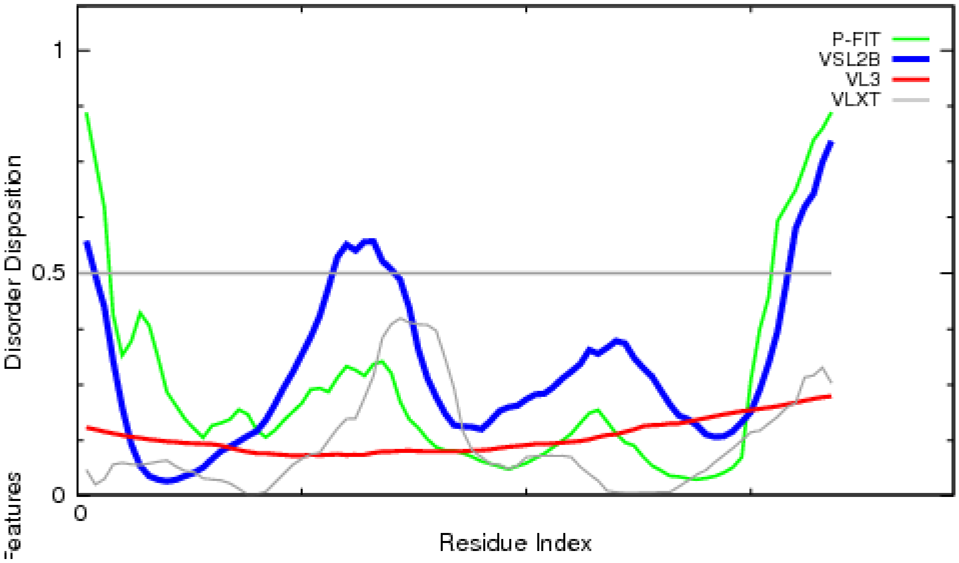

===============================PONDR VSL2 STATISTICS===============================

Predicted residues: 84
Number residues disordered: 13
Overall percent disordered: 15.48
Predicted disorder segment [29]-[35]
Predicted disorder segment [80]-[84]

Number Disordered Regions: 2
Longest Disordered Region: 7
Average Prediction Score: 0.2829
Average Strength= 0.5466
Average Strength= 0.6944

ANCHOR:

Predicted Disordered Binding Regions			
	From	To	Length
None			

ModPred and PROSITE:

ModPred: Amidation (Y70).
PROSITE: No identified domain recognition sites.

Structural modelling:

Name	Title	Identity	Method	Oligo State	Ligands
2m7g.1.A	Geopilin domain 1 protein	21.62	NMR	monomer	None
2lqx.1.A	Trypsin inhibitor BWI-2c	32.35	NMR	monomer	None
2l03.1.A	Ly-6/neurotoxin-like protein 1	22.58	NMR	monomer	None

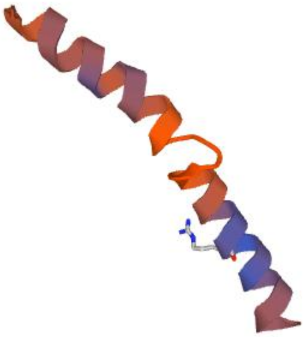

Model #1: Residues 6-44 of MVLG_06541T0 with 2m7g.1.A (21.62% sequence identity) as a template

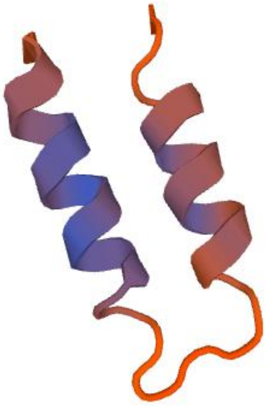

Model #2: Residues 29-64 of MVLG_06541T0 with 2lqx.1.A (32.35% sequence identity) as a template

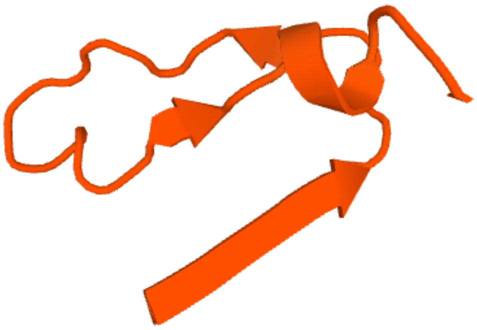

Model #3: Residues 48-82 of MVLG_06541T0 with 2l03.1.A (22.58% sequence identity) as a template

> MVLG_01897T0 (14.81%)- 385 aa

MLLSTTLLASLLVAVSAKKGLLAAYYPASQVDAAIDWNVTDIGYYMAAVTAKNGLAFPAGKPGL
ADFVLRAHAHKKKAVLSIGGPEGSQYFSSLVRTETARAKFVEQILEVGRKYNTDGVDISWQFPTVH
GNPKNEIDPKDSANLLKLLKDLRRSRPKEWLSAAVSPNGIFAPSGTTTLSNYQDFAEVVDAFNVMA
YHYVGAWNEWTGPDSPSHQCGTGRSVTTDIERFIKAGFVANKIMLGIPSFGKAFTLHNNTLRTSVV
YGDQQVPKKYQIRIRQRYETYNGADNTFVKLKAQGILKGEDGLTAGRGYKRHYDHCSRTPFLFNPG
TKSFITYLDARSASYRAQIAVQQEYLGVFVSSIGLDNLAFNAIDKALKTPIDGFATD

PONDR:

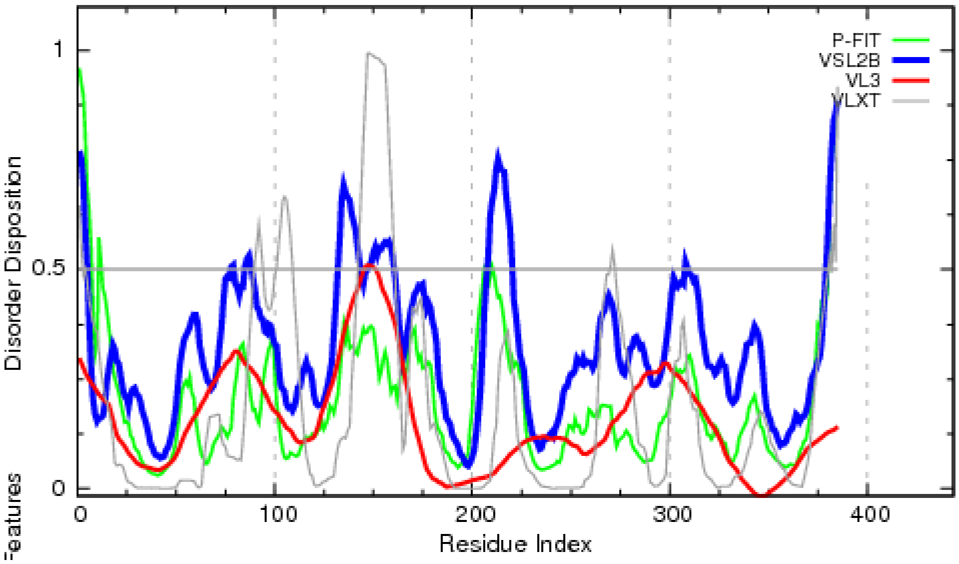

================================PONDR VSL2 STATISTICS================================

Predicted residues: 385
Number residues disordered: 57
Overall percent disordered: 14.81
Predicted disorder segment [1]-[5]
Predicted disorder segment [78]-[79]
Predicted disorder segment [85]-[88]
Predicted disorder segment [132]-[143]
Predicted disorder segment [148]-[160]
Predicted disorder segment [208]-[220]
Predicted disorder segment [308]-[309]
Predicted disorder segment [380]-[385]

Number Disordered Regions: 8
Longest Disordered Region: 13
Average Prediction Score: 0.3237
Average Strength= 0.6648
Average Strength= 0.5072
Average Strength= 0.5170
Average Strength= 0.6103
Average Strength= 0.5425
Average Strength= 0.6601
Average Strength= 0.5091
Average Strength= 0.7677

ANCHOR:

Predicted Disordered Binding Regions			
	From	To	Length
None			

Filtered Regions			
	From	To	Length
1	194	198	5

ModPred and PROSITE:

ModPred: Amidation (A9, P209, Y283, S340, Y353), Ubiquitination (K52), Proteolytic cleavage (D66, R154, R220), Acetylation (K248, K300), Pupylation (K376).

PROSITE: No identified domain recognition sites.

Structural modelling:

	Name	Title	Identity	Method	Oligo State	Ligands
	4txg.1.A	Chitinase	21.02	X-ray, 1.8Å	monomer	11 x CS

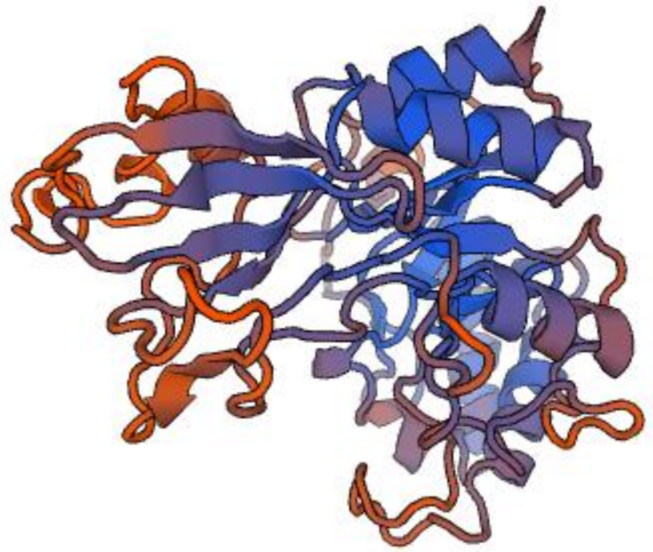

Model #1: Residues 17-377 of MVLG_01897T0 with 4txg.1.A (21.02% sequence identity) as a template

> MVLG_06973T0 (12.58%)- 604 aa

MWALGGIAGSVLWICAVNGQGSSYAPRRVRCPTDGPLVKSTGSPLAGNQFLESREATYQAARWN
KVLEPLYLKYLGNGQDTGYSTAQIATIVKHEPRIGTACSGGGLRASLYCAGTLSALDSRSRSHAAPV
LQLSAYMTGLSGGSWAITSLATSNLGPTSIYDIVLGKNGAPGWKLDLNLNFPSIKHLIPFNANIIRDLH
EKNRAHFSVTLIDYWGRLLGHHFLPGTTRASFFSQLAPNDNGLLFDAINSTSKFKEFEMPYPIVTTTS
RVRPWDQFKVVHDYIPAINTVFEISPYSFGSFDPSLSAHIPTEYMGSYVEQTQTGVARTCVNGFDSAS
FIMGCSAGLFTAIESMLQPDMKTFRRLLSLIHRVSKEEKLDILTSKVPNTFYGYNSGLMGSRRFESAE
NKNLYLTDGGMNGENIPLAPLLVKARRLDTIFAIDASQDTKMSWPNGVSLHRTWERINRTANGYSD
FPPVPSKPYDFLMGGLTRRPVFFGCNVKDARVDKPGNYPILIYLPNAPVPHSGYSTNTKTSQMEYSIS
DTEAFLNTVQANAMKGYPGGDAVVDREYKTALKCATVDRARQRGNMARSAICQIQMQRYCWPPL
KA

PONDR:

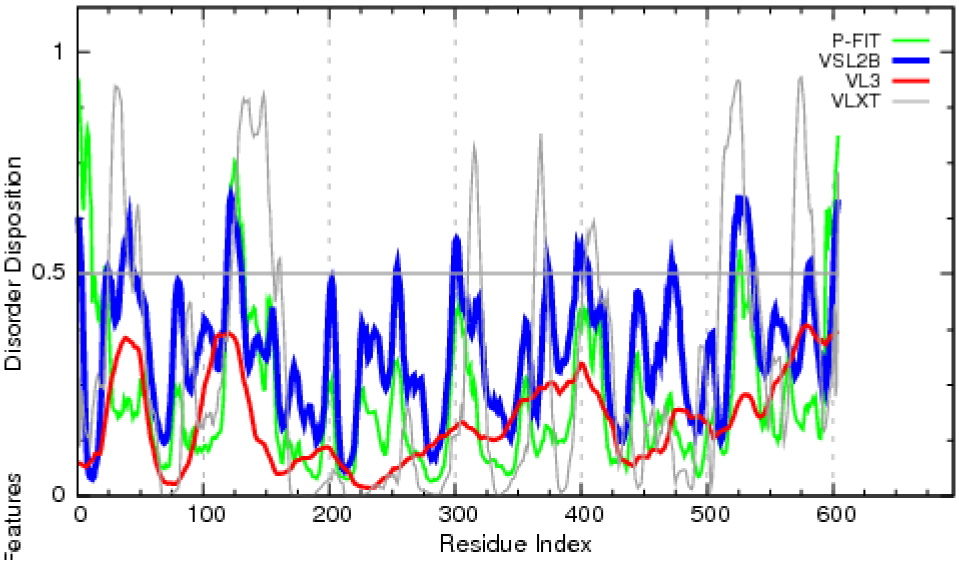

===============================PONDR VSL2 STATISTICS================================

Predicted residues: 604	Number Disordered Regions: 12
Number residues disordered: 76	Longest Disordered Region: 17
Overall percent disordered: 12.58	Average Prediction Score: 0.3269
Predicted disorder segment [1]-[3]	Average Strength= 0.5765
Predicted disorder segment [23]-[25]	Average Strength= 0.5060
Predicted disorder segment [35]-[44]	Average Strength= 0.5536
Predicted disorder segment [119]-[130]	Average Strength= 0.6016
Predicted disorder segment [253]-[254]	Average Strength= 0.5118
Predicted disorder segment [299]-[303]	Average Strength= 0.5561
Predicted disorder segment [373]-[374]	Average Strength= 0.5188
Predicted disorder segment [396]-[406]	Average Strength= 0.5331
Predicted disorder segment [471]-[474]	Average Strength= 0.5104
Predicted disorder segment [520]-[536]	Average Strength= 0.6177
Predicted disorder segment [580]-[582]	Average Strength= 0.5206
Predicted disorder segment [601]-[604]	Average Strength= 0.6225

ANCHOR:

Predicted Disordered Binding Regions			
	From	To	Length
1	491	496	6

Filtered Regions			
	From	To	Length
1	11	15	5
2	70	72	3
3	510	514	5

ModPred and PROSITE:

ModPred: Amidation (K39, E373), Acetylation (K73), Sumoylation (K255)

PROSITE: PLA2C (PLA2c domain profile, 30-604, PROSITE entry PS51210)

The PLA2c domain is the catalytic lipase domain in cytosolic phospholipase A2 (cPLA2) (EC 3.1.1.4) and lysophospholipase or phospholipase B (PLB) (EC 3.1.1.5) of vertebrates and fungi. It catalyzes the carboxylic ester hydrolysis of glycerophospholipids or lysophospholipids. The mammalian cPLA2 group IVA enzymes cleave intracellular phospholipid membranes to produce lipid mediators, which also play a role in inflammatory diseases such as asthma and arthritis. This enzyme contains a N-terminal calcium-binding C2 domain (see <PDOC00380>) that presents the catalytic domain to the membrane. Fungal secreted lysophospholipase/PLB can possess three different enzymatic activities, the hydrolase activity of phospholipase, lysophospholipase and a lysophospholipase transacylase activity.

Some fungal proteins known to contain a PLA2c domain:

- Fungal lysophospholipases/PLB, which are considered to be important for virulence of pathogenic fungi.
- Yeast sporulation-specific protein 1 (SPO1), which is required for meiosis.

Structural modelling:

Name	Title	Identity	Method	Oligo State	Ligands
5iz5.1.A	Cytosolic phospholipase A2 delta	19.26	X-ray, 2.2Å	monomer	None

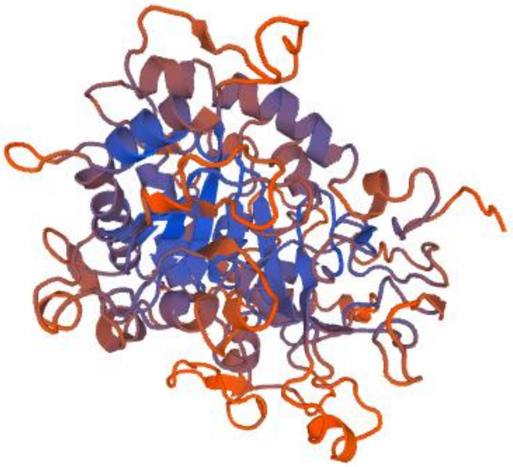

Model #1: Residues 31-577 of MVLG_06973T0 with 5iz5.1.A (19.26% sequence identity) as a template

Mostly ordered proteins (<10% disordered residues by PONDR® VSL2 analysis)

> MVLG_01005T0 (9.67%)- 331 aa

MHYTRLCLLCAALSNAPILLSARPQITMSADLSLECFIATTGLLSSPFARCADASGFLAALDAKIGL
ADALSDWLNNFCKDTCPDDARAKAWSGLEGGCADELAREIALPSVLLGTGHLRSSATAVQCPFRF
DNFVESNPILNARSTAVVANYDVLKRSACTGSVSRQSYCFVEFVKDLEEANKRNFTMSVDLLSPTC
AELNAVPRSKLCTLCNQMLFEMMVKLLSRPIDRVTLTDHARQACGLAFASLSALQADFPLRPVGGQ
FDQAVARYTAAAIKDLDQHNSTPSTPTTSSAYSSRTLSRRSTFALDTVVKAVAPTAAATILLYGWVQ

PONDR:

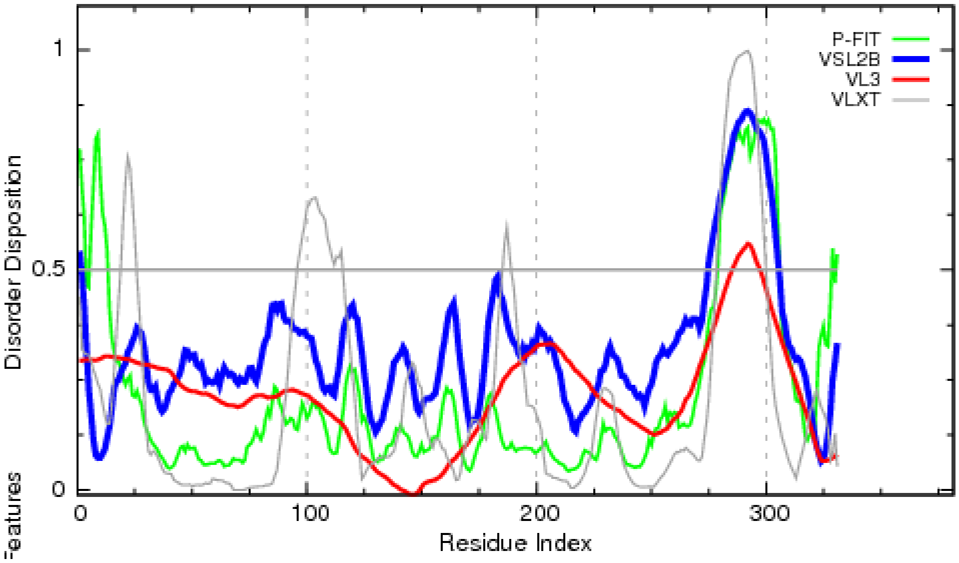

===============================PONDR VSL2 STATISTICS===============================

Predicted residues: 331
Number residues disordered: 32
Overall percent disordered: 9.67
Predicted disorder segment [275]-[305]

Number Disordered Regions: 1
Longest Disordered Region: 31
Average Prediction Score: 0.3224
Average Strength= 0.7347

ANCHOR:

Predicted Disordered Binding Regions			
	From	To	Length
None			
Filtered Regions			
	From	To	Length
1	325	331	7

ModPred and PROSITE:

ModPred: Pupylation (K177, K278), ADP-ribosylation (R206), N-linked glycosylation (N284), O-linked glycosylation (S293), Proteolytic cleavage (R304), Amidation (Y327), Pyrrolidone carboxylic acid (Q331).

PROSITE: No identified domain recognition sites.

Structural modelling:

	Name	Title	Identity	Method	Oligo State	Ligands
	3gxv.1.A	Replicative DNA helicase	22.50	X-ray, 2.2Å	hetero-oligomer	None
	5n9j.1.D	Mediator of RNA polymerase II transcription subunit 21	25.71	X-ray, 3.4Å	hetero-oligomer	None
	3ajb.1.B	Peroxisomal biogenesis factor 19	22.22	X-ray, 2.5Å	hetero-oligomer	None

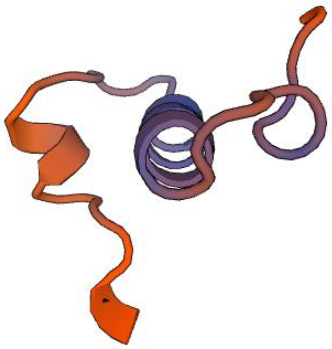

Model #1: Residues 202-241 of MVLG_01005T0 with 3gxv.1.A (22.50% sequence identity) as a template

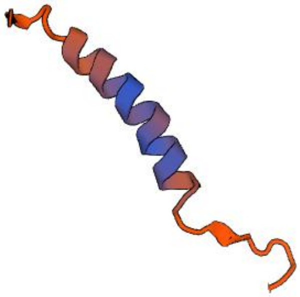

Model #2: Residues 230-264 of MVLG_01005T0 with 5n9j.1.D (25.71% sequence identity) as a template

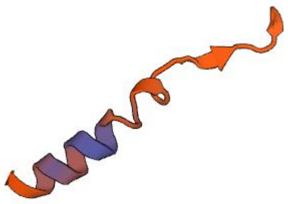

Model #3: Residues 266-291 of MVLG_01005T0 with 3ajb.1.B (22.22% sequence identity) as a template

Table S3: Primers used for PCR and sequencing

Primer	Sequence (5'→3')	Usage
MVLG_04106F	GCC GAA TTC GCT GAC GCG ACC AAA CAG GCC TCC	Yeast two hybrid
MVLG_04106R	GAC GGA TCC TCA ACA ACC TTC GGG CTC GGG TTG	Yeast two hybrid
MVLG_05720F	GCC GAA TTC AAC CCG TGG CCT CCG TCG GTT CAA	Yeast two hybrid
MVLG_05720R	GAC GGA TCC CTA GTA ACC CGA ACG ACG CAT CCT	Yeast two hybrid
MVLG_06175F	GCC GAA TTC TTT TGT CCC TTT GGA AAA ACG GCG	Yeast two hybrid
MVLG_06175R	GAC GGA TCC TTA GAG ATT TAG AGG AAA GAA CCA	Yeast two hybrid
MVLG_01732F	GCC GAA TTC TTG CAA GAA GCG GGC GAT ACC AAG	Yeast two hybrid
MVLG_01732R	GAC GGA TCC CTA GGC GTG GAT TTT GCC GGA GAA	Yeast two hybrid
T7 Sequencing primer	AATACGACTCACTATAGGGCG	Yeast two hybrid
MVLG_04106F	GCC GAA TTC ATG AAG TAC TCG CTC GTC TTT GTC	Yeast secretion trap
MVLG_04106R	GCG GCC GCC GGC GAG AGC CGA GAC GAT GCG CGT G	Yeast secretion trap
MVLG_05720F	GCC GAA TTC ATG ATG CGT TCC CTC ATC AAG TTG	Yeast secretion trap
MVLG_05720R	GCG GCC GCC CGC AAG AGC CAC ACT GAC GGC GGT G	Yeast secretion trap
MVLG_06175F	GCC GAA TTC ATG TGG ACC TCT TCG ATC GTC CAA	Yeast secretion trap
MVLG_06175R	GCG GCC GCC AGC CCA CGC CAC GAC AGG GCT CGA G	Yeast secretion trap
MVLG_01732F	GCC GAA TTC ATG CTG TTA AAG CTT ACC ATC ACC	Yeast secretion trap
MVLG_01732R	GCG GCC GCC TGC CGA AAC ATT GAG GAC GAG TAA G	Yeast secretion trap
Sequencing primer	TCCTCGTCATTGTTCTCGTTCC	Yeast secretion trap

Figure S1: Structure of Protein: Domain structures of AtCLB protein from *Silene latifolia* drawn based on SMART searches (http://smart.embl-heidelberg.de/). The length of the protein and the positions of TM, C_2 domains are scaled below.

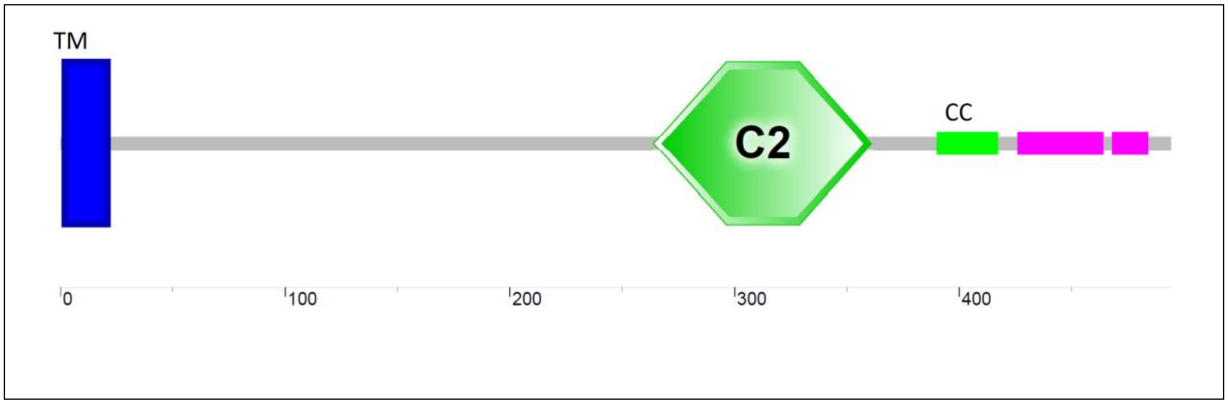

TM- Transmembrane region (amino acids 2-21), C2 domain (amino acids 264-361), CC-Coiled coil (amino acids 390-417).

Figure S2: Structure of Protein: Domain structures of the Cellulose Synthase Interactive 1 protein from *Silene latifolia* drawn based on SMART searches (http://smart.embl-heidelberg.de/). The length of the protein and the positions of ARM and C_2 domains are scaled below.

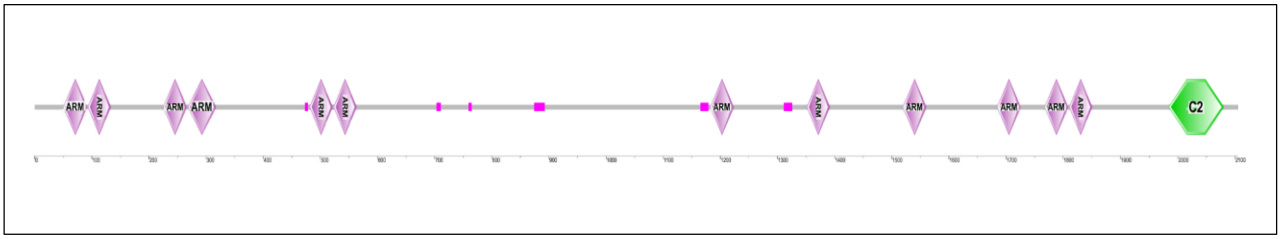

Arm repeats (amino acids 50-92, 93-133, 225-266, 268-317, 481-521, 523-563, 1182-1223, 1351-1391, 1519-1560, 1684-1725, 1767-1808, 1810-1850), C2 domain (amino acids 1984-2080)

Figure S3: Predicted functional partners using STRING for the predicted MVLG_01732 host target, AtCLB, *Arabidopsis thaliana* gene At3G61050.1, network and interactions with other proteins.

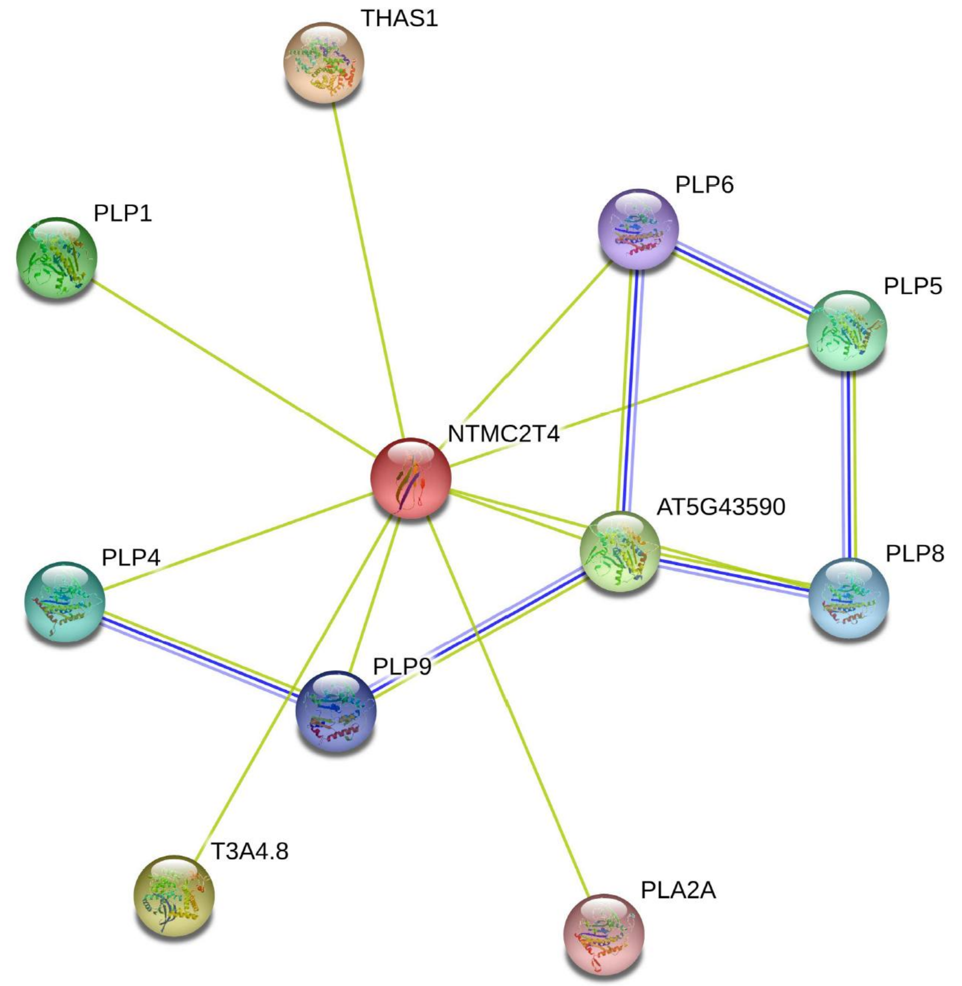

Edges:

Edges represent protein-protein associations

associations are meant to be specific and meaningful, i.e. proteins jointly contribute to a shared function; this does not necessarily mean they are physically binding each other.

Known Interactions

from curated databases

experimentally determined

Predicted Interactions

gene neighborhood

gene fusions

gene co-occurrence

Others

textmining

co-expression

protein homology

Your Input:

NTMC2T4 *calcium-dependent lipid-binding domain-containing protein (510 aa)*

Predicted Functional Partners:

		Neighborhood	Gene Fusion	Cooccurence	Coexpression	Experiments	Databases	Textmining	[Homology]	Score
THAS1	*thalianol synthase 1; Converts oxidosqualene to thalianol (766 aa)*							•		0.829
T3A4.8	*CDPK-related kinase 3; May play a role in signal transduction pathways that involve calcium as a second messenger (By si...*							•		0.596
AT5G43590	*acyl transferase/acyl hydrolase/lysophospholipase-like protein (401 aa)*							•		0.569
PLP1	*patatin-like protein (414 aa)*							•		0.569
PLP5	*PATATIN-like protein 5 (435 aa)*							•		0.569
PLP4	*PATATIN-like protein 4 (428 aa)*							•		0.569
PLP8	*PATATIN-like protein 8 (526 aa)*							•		0.569
PLP9	*PATATIN-like protein 9 (384 aa)*							•		0.569
PLP6	*PATATIN-like protein 6 (499 aa)*							•		0.569
PLA2A	*phospholipase A 2A (407 aa)*							•		0.569

Functional enrichments in the network

Biological Process (GO)			
pathway ID	*pathway description*	*count in gene set*	*false discovery rate*
GO:0016042	lipid catabolic process	8	5.46e-12
GO:0006629	lipid metabolic process	9	1.22e-09
GO:0006952	defense response	7	3.16e-05
GO:0044238	primary metabolic process	10	0.00354
GO:0044699	single-organism process	10	0.0037
GO:0071704	organic substance metabolic process	10	0.00408
GO:0050896	response to stimulus	8	0.0107
GO:0010311	lateral root formation	2	0.0249
GO:0048513	organ development	4	0.0306
GO:0010102	lateral root morphogenesis	2	0.0373

(less ...)

Molecular Function (GO)			
pathway ID	*pathway description*	*count in gene set*	*false discovery rate*
GO:0047372	acylglycerol lipase activity	3	1.21e-06
GO:0016298	lipase activity	4	3.51e-05
GO:0016787	hydrolase activity	8	8.82e-05
GO:0004620	phospholipase activity	3	0.000517
GO:0003824	catalytic activity	10	0.000652

node1	node2	node1 accession	node2 accession	node1 annotation	node2 annotation	score
AT5G43590	NTMC2T4	AT5G43590.1	AT3G61050.1	*acyl transferase/acyl hydrolase...*	*calcium-dependent lipid-bindin...*	0.569
AT5G43590	PLP6	AT5G43590.1	AT2G39220.1	*acyl transferase/acyl hydrolase...*	*PATATIN-like protein 6*	0.407
AT5G43590	PLP8	AT5G43590.1	AT4G29800.2	*acyl transferase/acyl hydrolase...*	*PATATIN-like protein 8*	0.430
AT5G43590	PLP9	AT5G43590.1	AT3G63200.1	*acyl transferase/acyl hydrolase...*	*PATATIN-like protein 9*	0.421
NTMC2T4	AT5G43590	AT3G61050.1	AT5G43590.1	*calcium-dependent lipid-bindin...*	*acyl transferase/acyl hydrolase...*	0.569
NTMC2T4	PLA2A	AT3G61050.1	AT2G26560.1	*calcium-dependent lipid-bindin...*	*phospholipase A 2A*	0.569
NTMC2T4	PLP1	AT3G61050.1	AT4G37070.2	*calcium-dependent lipid-bindin...*	*patatin-like protein*	0.569
NTMC2T4	PLP4	AT3G61050.1	AT4G37050.1	*calcium-dependent lipid-bindin...*	*PATATIN-like protein 4*	0.569
NTMC2T4	PLP5	AT3G61050.1	AT4G37060.2	*calcium-dependent lipid-bindin...*	*PATATIN-like protein 5*	0.569
NTMC2T4	PLP6	AT3G61050.1	AT2G39220.1	*calcium-dependent lipid-bindin...*	*PATATIN-like protein 6*	0.569
NTMC2T4	PLP8	AT3G61050.1	AT4G29800.2	*calcium-dependent lipid-bindin...*	*PATATIN-like protein 8*	0.569
NTMC2T4	PLP9	AT3G61050.1	AT3G63200.1	*calcium-dependent lipid-bindin...*	*PATATIN-like protein 9*	0.569
NTMC2T4	T3A4.8	AT3G61050.1	AT2G46700.1	*calcium-dependent lipid-bindin...*	*CDPK-related kinase 3; May pla...*	0.596
NTMC2T4	THAS1	AT3G61050.1	AT5G48010.2	*calcium-dependent lipid-bindin...*	*thalianol synthase 1; Converts ...*	0.829
PLA2A	NTMC2T4	AT2G26560.1	AT3G61050.1	*phospholipase A 2A*	*calcium-dependent lipid-bindin...*	0.569
PLP1	NTMC2T4	AT4G37070.2	AT3G61050.1	*patatin-like protein*	*calcium-dependent lipid-bindin...*	0.569
PLP4	NTMC2T4	AT4G37050.1	AT3G61050.1	*PATATIN-like protein 4*	*calcium-dependent lipid-bindin...*	0.569
PLP4	PLP9	AT4G37050.1	AT3G63200.1	*PATATIN-like protein 4*	*PATATIN-like protein 9*	0.434
PLP5	NTMC2T4	AT4G37060.2	AT3G61050.1	*PATATIN-like protein 5*	*calcium-dependent lipid-bindin...*	0.569
PLP5	PLP6	AT4G37060.2	AT2G39220.1	*PATATIN-like protein 5*	*PATATIN-like protein 6*	0.418

node1	node2	node1 accession	node2 accession	node1 annotation	node2 annotation	score
PLP5	PLP8	AT4G37060.2	AT4G29800.2	*PATATIN-like protein 5*	*PATATIN-like protein 8*	0.426
PLP6	AT5G43590	AT2G39220.1	AT5G43590.1	*PATATIN-like protein 6*	*acyl transferase/acyl hydrolase/ly...*	0.407
PLP6	NTMC2T4	AT2G39220.1	AT3G61050.1	*PATATIN-like protein 6*	*calcium-dependent lipid-binding d...*	0.569
PLP6	PLP5	AT2G39220.1	AT4G37060.2	*PATATIN-like protein 6*	*PATATIN-like protein 5*	0.418
PLP8	AT5G43590	AT4G29800.2	AT5G43590.1	*PATATIN-like protein 8*	*acyl transferase/acyl hydrolase/ly...*	0.430
PLP8	NTMC2T4	AT4G29800.2	AT3G61050.1	*PATATIN-like protein 8*	*calcium-dependent lipid-binding d...*	0.569
PLP8	PLP5	AT4G29800.2	AT4G37060.2	*PATATIN-like protein 8*	*PATATIN-like protein 5*	0.426
PLP9	AT5G43590	AT3G63200.1	AT5G43590.1	*PATATIN-like protein 9*	*acyl transferase/acyl hydrolase/ly...*	0.421
PLP9	NTMC2T4	AT3G63200.1	AT3G61050.1	*PATATIN-like protein 9*	*calcium-dependent lipid-binding d...*	0.569
PLP9	PLP4	AT3G63200.1	AT4G37050.1	*PATATIN-like protein 9*	*PATATIN-like protein 4*	0.434
T3A4.8	NTMC2T4	AT2G46700.1	AT3G61050.1	*CDPK-related kinase 3; May play a...*	*calcium-dependent lipid-binding d...*	0.596
THAS1	NTMC2T4	AT5G48010.2	AT3G61050.1	*thalianol synthase 1; Converts oxi...*	*calcium-dependent lipid-binding d...*	0.829

NTMC2T4- FUNCTIONS IN: lipid binding; INVOLVED IN: biological_process unknown; LOCATED IN: plasma membrane; EXPRESSED IN: male gametophyte, cultured cell, callus, pollen tube; EXPRESSED DURING: M germinated pollen stage; CONTAINS InterPro DOMAIN/s: C2 membrane targeting protein (InterPro:IPR018029), C2 calcium/lipid-binding domain, CaLB (InterPro:IPR008973), C2 region (InterPro:IPR020477), C2 calcium-dependent membrane targeting (InterPro:IPR000008); BEST Arabidopsis thaliana protein match is: Calcium-dependent lipid-binding (CaLB domain) family protein (TAIR:AT3G61030.1). /db_xref="Araport:AT3G61050" , /db_xref="GeneID:825277", /db_xref="TAIR:AT3G61050" [77].
